# Novel Pyrazolo [1,5‐a]−1,3,5‐Triazine Derivatives as CDK7 Inhibitors: Synthesis and Biological Insights in Pancreatic Ductal Adenocarcinoma Models

**DOI:** 10.1002/cmdc.202500448

**Published:** 2025-08-25

**Authors:** Daniela Carbone, Francesca Terrana, Ludovica Sciuto, Camilla Pecoraro, Geng Xu, Stella Cascioferro, Girolamo Cirrincione, Godefridus J. Peters, Elisa Giovannetti, Barbara Parrino, Patrizia Diana

**Affiliations:** ^1^ Department of Biological, Chemical, and Pharmaceutical Sciences and Technologies (STEBICEF) University of Palermo Viale delle Scienze Ed.16 90128 Palermo Italy; ^2^ Department of Medical Oncology Cancer Center Amsterdam Amsterdam UMC, VU University 1081 HV Amsterdam the Netherlands; ^3^ Cancer Pharmacology Laboratory Fondazione Pisana per la Scienza Via Ferruccio Giovannini 13 56017 Pisa Italy

**Keywords:** antiproliferative activity, CDK7 inhibition, inhibition of cells migration, pancreatic ductal adenocarcinoma, pyrazolo[1,5‐a]−1,3,5‐triazine derivatives, spheroids shrinkage

## Abstract

Pancreatic ductal adenocarcinoma (PDAC), the most prevalent form of pancreatic tumor, is one of the most aggressive and lethal tumor types. Cyclin‐dependent kinase 7 (CDK7) has been recently identified as a promising target in multiple human and mouse PDAC preclinical tumor models, due to significant downregulation of gene transcription and preferential inhibition of the mitotic cell cycle. With the aim of finding new CDK7 inhibitors, new indolyl and 7‐aza‐indolyl pyrazolo [1,5‐a]−1,3,5‐triazine derivatives are efficiently synthesized and screened for antiproliferative activity against three immortalized cell lines (SUIT 2.28, PATU‐T, PANC‐1) of PDAC. 8 out of 33 derivatives show remarkable cytotoxicity with IC_50_ values ranging from 0.19 to 1.58 µM and remarkable inhibition of cell migration from the earliest time point of 4 h, persisting until 24 h. The two most active compounds are further evaluated in clinically relevant models,including gemcitabine‐resistant and primary cells (PATU‐T GR, PDAC3), confirming their potent activity. They induce apoptosis, upregulate apoptotic gene expression, and disrupt the cell cycle, significantly reducing the viability of spheroidal PATU‐T cultures. Additionally, both compounds effectively inhibit CDK7, as demonstrated by an enzyme‐linked immunosorbent assay in cell extracts and by a specific enzymatic activity assay.

## Graphical abstract

Pyrazolo [1,5‐a]−1,3,5‐triazine derivatives efficiently synthesized showed remarkable cytotoxicity and inhibition of cell migration in PDAC models. They induced apoptosis, upregulated apoptotic gene expression, and disrupted the cell cycle, significantly reducing the viability of spheroidal PATU‐T cultures. Pyrazolo [1,5‐a]−1,3,5‐triazine derivatives efficiently synthesized showed remarkable cytotoxicity and inhibition of cell migration in PDAC models. They induced apoptosis, upregulated apoptotic gene expression, and disrupted the cell cycle, significantly reducing the viability of spheroidal PATU‐T cultures.

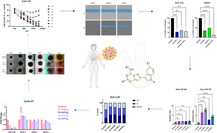

## 1 Introduction

Pancreatic cancer is currently the fourth leading cause of cancer‐related deaths globally and is expected to become the second leading cause of cancer‐related deaths by 2030.^[^ ^1^ ^,^ ^2^ ^]^ The most common form of pancreatic tumor is pancreatic ductal adenocarcinoma (PDAC), which accounts for over 80% of all pancreatic cancers.^[^ ^3^ ^]^ PDAC has an extremely poor prognosis, with a 5‐year survival rate of less than 13%.^[^ ^4^ ^]^ According to the Global Cancer Statistics 2020, the incidence and mortality rates of PDAC patients are nearly identical, highlighting it as one of the most aggressive and deadly tumor types.^[^ ^5^ ^,^ ^6^ ^]^ Though extensive research on PDAC has been conducted, only modest improvements in survival rates have been achieved in recent decades. These severely low survival rates can be attributed to the absence of specific symptoms and rapid cancer progression, which often result in very late diagnoses and therapeutic failure.^[^ ^7^ ^]^ At present, the standard chemotherapy treatment for patients with advanced or metastatic pancreatic cancer involves combinations of 5‐fuorouracil/leucovorin with irinotecan and oxaliplatin (FOLFIRINOX) or gemcitabine plus nanoparticle albumin‐bound paclitaxel.^[^ ^8^ ^]^ Retrospective studies have shown that younger patients treated with FOLFIRINOX have a better overall survival rate compared to those receiving gemcitabine plus nab‐paclitaxel.^[^ ^8^ ^,^ ^9^ ^]^ However, both therapeutic options are limited by a proportion of patients who do not respond to therapy.^[^ ^10^ ^,^ ^11^ ^]^ Thus, new drugs and new strategies that increase the efficacy of chemotherapy in PDAC are urgently needed.

Cyclin‐dependent kinases (CDKs), a family of Ser/Thr kinases that require a specific regulatory subunit, known as a cyclin, for activation, were recently identified as promising targets for PDAC treatment.^[^ ^12^ ^]^ Tumor progression is linked to genetic or epigenetic events that cause an over‐expression of cyclins, constitutive activation of CDKs, loss of CDK inhibitors (such as p27 and p16), and mutations of the retinoblastoma gene. These alterations disrupt the normal regulation of the cell cycle, giving cancer cells a growth advantage.

In particular, CDK7 has emerged as a promising cancer therapeutic target due to its dual role in cell cycle and transcription mechanisms.^[^ ^13^ ^]^ This protein is activated by the phosphorylation of T‐loop residues Thr170 and Ser164 by CDK1 and CDK2. Its influence on cell cycle progression is mediated through cooperation with cyclin H and MAT1, forming the ternary activating kinase complex (CAK). This complex is responsible for activating CDKs such as CDK1, CDK2, CDK4, and CDK6 by phosphorylating their key threonine residues in a process known as “T‐loop activation.”^[^ ^14^, ^15^ ^–^ ^16^ ^]^

On the other hand, the CDK7‐cyclin H‐MAT1 complex also plays an important role in the transcription process by influencing the function of RNA polymerase II. This ternary complex is actually one of the eight subunits of the TFIIH transcription factor complex, which phosphorylates a serine residue in the C‐terminal domain of RNA polymerase II. This phosphorylation promotes the initiation of transcription and allows the elongation complex to progress downstream from the transcription start site.^[^ ^17^ ^]^

Recent studies have demonstrated that inhibiting CDK7 has therapeutical potential in multiple human and mouse PDAC preclinical tumor models. This effect is due to a significant downregulation of gene transcription and preferential inhibition of the mitotic cell cycle, suggesting that targeting CDK7 could be a new strategy to counteract PDAC progression.^[^ ^18^ ^,^ ^19^ ^]^ Moreover, CDK7 inhibition was found to enhance cell apoptosis and DNA damage after chemotherapy improving the response to gemcitabine and paclitaxel in pancreatic cancer in vitro and acting synergistically with standard chemotherapy, suppressing PDAC tumor growth in vivo.^[^ ^20^ ^]^

**Figure 1:**
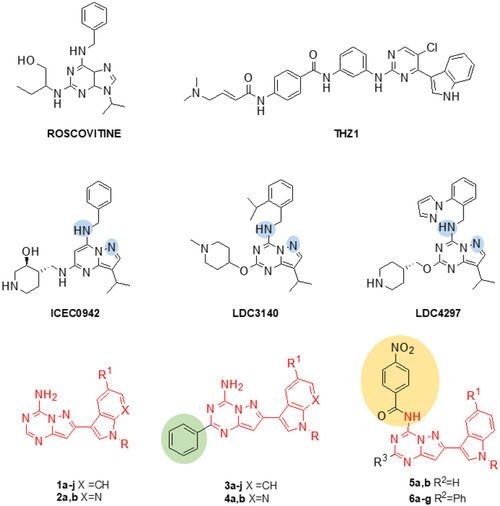
Chemical structures of CDK7 inhibitors and the new pyrazolo[1,5‐a]−1,3,5‐triazine derivatives **1–6**.

Over the last decades, different CDK7 inhibitors were reported belonging to the main categories of the ATP‐competitive inhibitors based on the primary scaffold roscovitine and the covalent‐binding inhibitors based on THZ1 scaffold (**Figure** **1**).^[^ ^17^ ^]^ Among roscovitine analogs, pyrazolo [1,5‐a] pyrimidine and pyrazolo[1,5‐a]−1,3,5‐triazine derivatives showed potent CDK7 inhibition. In particular, the pyrazolo[1,5‐a] pyrimidine samuraciclib (ICEC0942) (Figure 1) showed high affinity and selectivity for CDK7 over other CDKs and is currently undergoing clinical trials.^[^ ^13^ ^,^ ^17^ ^]^ Pyrazolo[1,5‐a]−1,3,5‐triazine derivatives LDC3140 and LDC4297 (Figure 1) have shown selective inhibition of CDK7 with IC_50_ values less than 5 nM. The X‐ray crystallographic structures of CDK7 in complex with ICEC0942 and LDC4297^[^ ^21^ ^,^ ^22^ ^]^ evidenced the importance of both exocyclic nitrogen and N6 of the two pyrazolo[1,5‐a]pyrimidine and pyrazolo[1,5‐a]−1,3,5‐triazine systems, for specific H‐bond interactions with CDK7 ATP‐binding site. Recent studies have highlighted that while most inhibitors of this class maintain substitution at the C8 position, substituents at the C2 position and on the exocyclic nitrogen are predominantly responsible for enhancing the selectivity of these inhibitors toward this kinase over other CDKs.^[^ ^23^ ^]^

Indole and 7‐aza‐indole rings have emerged as privileged pharmacophores for the development of new drugs with antitumor activity,^[^ ^24^ ^,^ ^25^ ^]^ particularly as kinases inhibitors.^[^ ^26^, ^27^ ^–^ ^28^ ^]^ Thus, continuing our ongoing studies on indolyl and 7‐aza‐indolyl CDK inhibitors,^[^ ^29^ ^,^ ^30^ ^]^ we report the synthesis of new pyrazolo[1,5‐a]−1,3,5‐triazine derivatives. In these compounds, the C7 position of the bicyclic scaffold was functionalized with either an indole or 7‐aza‐indole moiety, affording two novel scaffolds, designated as types **1** and **2**. Furthermore, given the critical role of substitutions at the C2 position and on the exocyclic nitrogen in conferring CDK7 selectivity, we investigated the functionalization of these sites, leading to the generation of compounds of types **3, 4** and **5, 6**, respectively (Figure 1). Their biological properties were investigated in representative PDAC models.

## 2 Results and Discussion

### 2.1 Chemistry

**Scheme 1:**
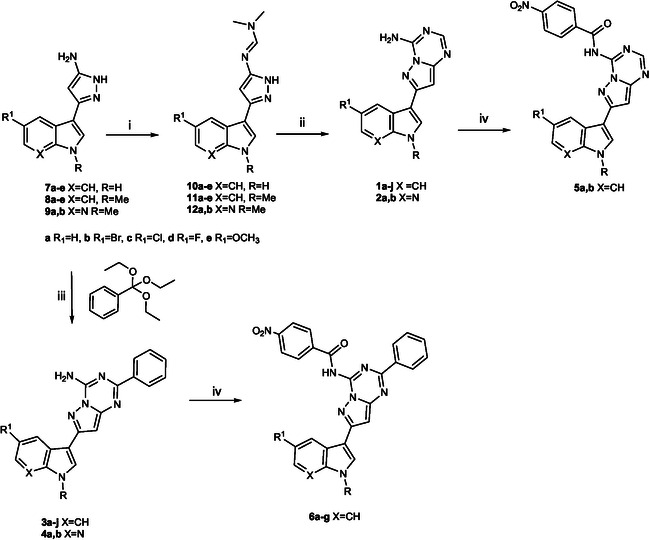
General synthetic pathway for the preparation of the new pyrazolo [1,5‐a]−1,3,5‐triazine derivatives. Reagents and conditions*:* i) DMF‐DMA in 1,4‐dioxane, 1–6 h, reflux, 53–99%; ii) cyanamide, NaH in DMSO, 1–2.5 h, reflux, 40–93%; iii) cyanamide in DMSO, 2 h at 165 °C, then NaH, 2.5 h at 180 °C, 53–97%; iv) NaH, in tetrahydrofuran, 1 h at 0 °C, then‐nitrobenzoyl chloride, 30 min, 0 °C, 54–99%.

**Scheme 2:**
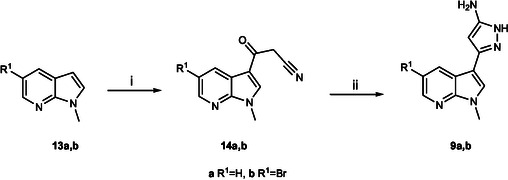
Synthesis of pyrazolo‐5‐amines **9a,b**. Reagents and conditions*:* i) cyanoacetic acid, in acetic anhydride, 5–24 h, 85 °C, 45–48%; ii) acetyl hydrazine, PTSA·H_2_O, in *n*‐butanol 45 min‐1 h, reflux, 49–55%.

The new pyrazolo[1,5‐a]−1,3,5‐triazine derivatives **1–6** were synthesized from pyrazolo‐5‐amine intermediates **7–9** (**Scheme** **1**). Derivatives **7** and **8** were prepared as reported in literature;^[^ ^31^ ^,^ ^32^ ^]^ in case of intermediates **9a, b** the synthesis was carried out according to **Scheme** **2**. The methylated 7‐azaindoles **13a, b**, prepared as previously reported,^[^ ^33^ ^]^ were converted to their corresponding 3‐oxopropanitriles **14a, b** using a mixture of cyanoacetic acid and acetic anhydride at 85 °C. Compounds **14** were then reacted with acetylhydrazine in the presence of *p*‐toluenesulfonic acid monohydrate (PTSA.H_2_O) in *n*‐butanol under reflux, to obtain the desired intermediates **9a, b** (Scheme 2).

Once obtained, the amines **7–9** were converted to their corresponding *N, N*‐dimethylmethanimidamides **10–12** using *N,N*‐dimethylformamide dimethyl acetal (DMF DMA) in 1,4‐dioxane under reflux (Scheme 1, **Table** **1**). Thus, indolyl derivatives **10,11** and 7‐aza‐indolyl derivatives **12** were subjected to cyclization reaction using sodium hydride in dimethylsulfoxide (DMSO) to afford the new derivatives **1a‐j** and **2a, b**, respectively.

**Table 1:** Pyrazolo [1,5‐a]−1,3,5‐triazine derivatives 1–6.

Cmp		R	R^1^	X	Yield [%]	Cmp	R	R^1^	X	Yield [%]
**1a**	H		H	CH	45	**3f**	Me	H	CH	77
**1b**	H		Br	CH	80	**3g**	Me	Br	CH	78
**1c**	H		Cl	CH	60	**3hr**	Me	Cl	CH	75
**1d**	H		F	CH	70	**3i**	Me	F	CH	60
**1e**	H		OMe	CH	71	**3j**	Me	OMe	CH	90
**1f**	Me		H	CH	93	**4a**	Me	H	N	87
**1g**	Me		Br	CH	70	**4b**	Me	Br	N	89
**1h**	Me		Cl	CH	50	**5a**	Me	H	CH	58
**1i**	Me		F	CH	80	**5b**	Me	F	CH	55
**1j**	Me		OMe	CH	40	**6a**	Hl	H	CH	54
**2a**	Me		H	N	65	**6b**	H	OMe	CH	65
**2b**	Me		Br	N	55	**6c**	Me	H	CH	68
**3a**	H		H	CH	97	**6d**	Me	Bt	CH	85
**3b**	H		Br	CH	53	**6e**	Me	Cl	CH	77
**3c**	H		Cl	CH	64	**6f**	Me	F	CH	99
**3d**	H		F	CH	71	**6g**	Me	OMe	CH	89
**3e**	H		OMe	CH	64	–	–	–	–	–

The amine intermediates **7–9** were also reacted with commercial available triethyl orthobenzoate and cyanamide in dimethylsulfoxide (DMSO) at 165 °C, to afford 2‐substituted indolyl pyrazolo [1,5‐a]−1,3,5‐triazines of type **3** and 7‐aza‐indolyl pyrazolo [1,5‐a]−1,3,5‐triazines of type **4** (Scheme 1, Table 1).

Finally, derivatives **2a, b** and **3a, e‐j** were further functionalized by *N*‐acylation reaction with 4‐nitro‐benzoyl chloride, carried out in the presence of sodium hydride, to afford derivatives **5a, b** and **6a‐g**, respectively (Table 1).

### 2.2 Antiproliferative Activity

**Figure 2:**
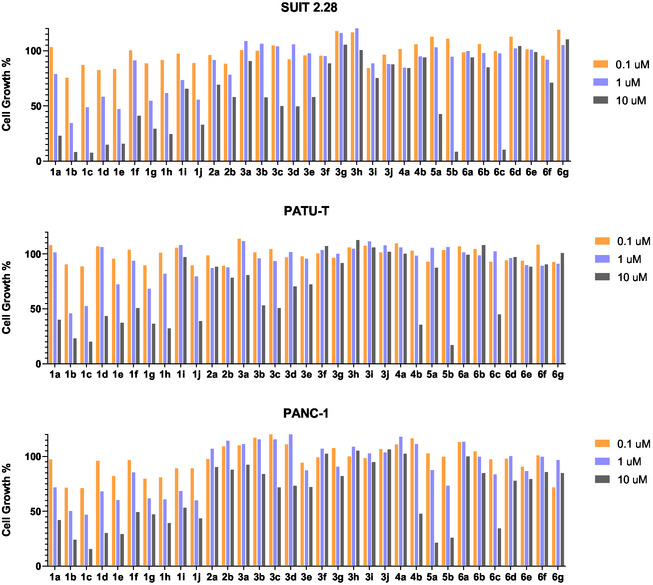
Screening at three concentrations (0.1, 1 and 10 µM) of newly synthesized compounds in immortalized SUIT 2.28, PATU‐T, and PANC‐1 cell lines.

The newly synthesized pyrazolo[1,5‐a]−1,3,5‐triazine derivatives **1–6** were preliminary screened against three immortalized PDAC cell lines (SUIT 2.28, PATU‐T, PANC‐1) at three different concentrations: 0.1, 1 and 10 µM, with 72 h of exposure. On the basis of the obtained results (**Figure** **2**), the most effective compounds, **1b, c–e, g,h‐j**, were selected for the calculation of the IC_50_ values, (**Table** **2**).The IC_50_ measurements were performed after 72 h of treatment. All compounds reduced cell viability in all the immortalized cell lines. The SUIT 2.28 cell line was the most sensitive, with IC_50_ values ranging from 0.2 to 1.6 µM. The most effective compound for these cell lines was **1g**, which exhibited the lowest IC_50_ of all the tested compounds. The next most sensitive line was PATU‐T, with IC_50_ values ranging from 1.4 to 5.5 µM, where compounds **1b** and **1g** showed the highest effectiveness, similar to their performance in SUIT 2.28. PANC‐1 was the least sensitive; however, cell viability was still inhibited, with IC_50_ values ranging from 2.2 to 8.8 µM. In this line, while compounds **1b** and **1g** did not have the lowest IC_50_, they remained among the most effective compared to the other compounds.

**Table 2:** IC_50_ values of the selected compounds for PATU‐T, PANC‐1 and SUIT 2.28.

Compound	IC_50_ [μM]PATU‐Ta)	IC_50_ [μM]PANC‐1a)	IC_**50**_ [μM]SUIT 2.28a)
**1b**	1.4 ± 0.2	4.3 ± 0.8	0.6 ± 0.1
**1c**	1.9 ± 0.6	8.7 ± 1.5	0.4 ± 0.1
**1d**	3.5 ± 0.7	9.6 ± 0.8	0.6 ± 0.1
**1e**	4.7 ± 0.9	8.8 ± 1.2	0.5 ± 0.1
**1g**	1.3 ± 0.1	2.9 ± 0.8	0.2 ± 0.1
**1hr**	2.0 ± 0.3	3.1 ± 0.7	1.6 ± 0.3
**1i**	5.5 ± 1.1	2.2 ± 0.4	0.5 ± 0.1
**1j**	4.7 ± 0.8	2.4 ± 0.5	0.5 ± 0.1

a) Each value (average ± SEM) was obtained from triplicate experiments.

### 2.3 Reduction of Cell Migration

**Figure 3:**
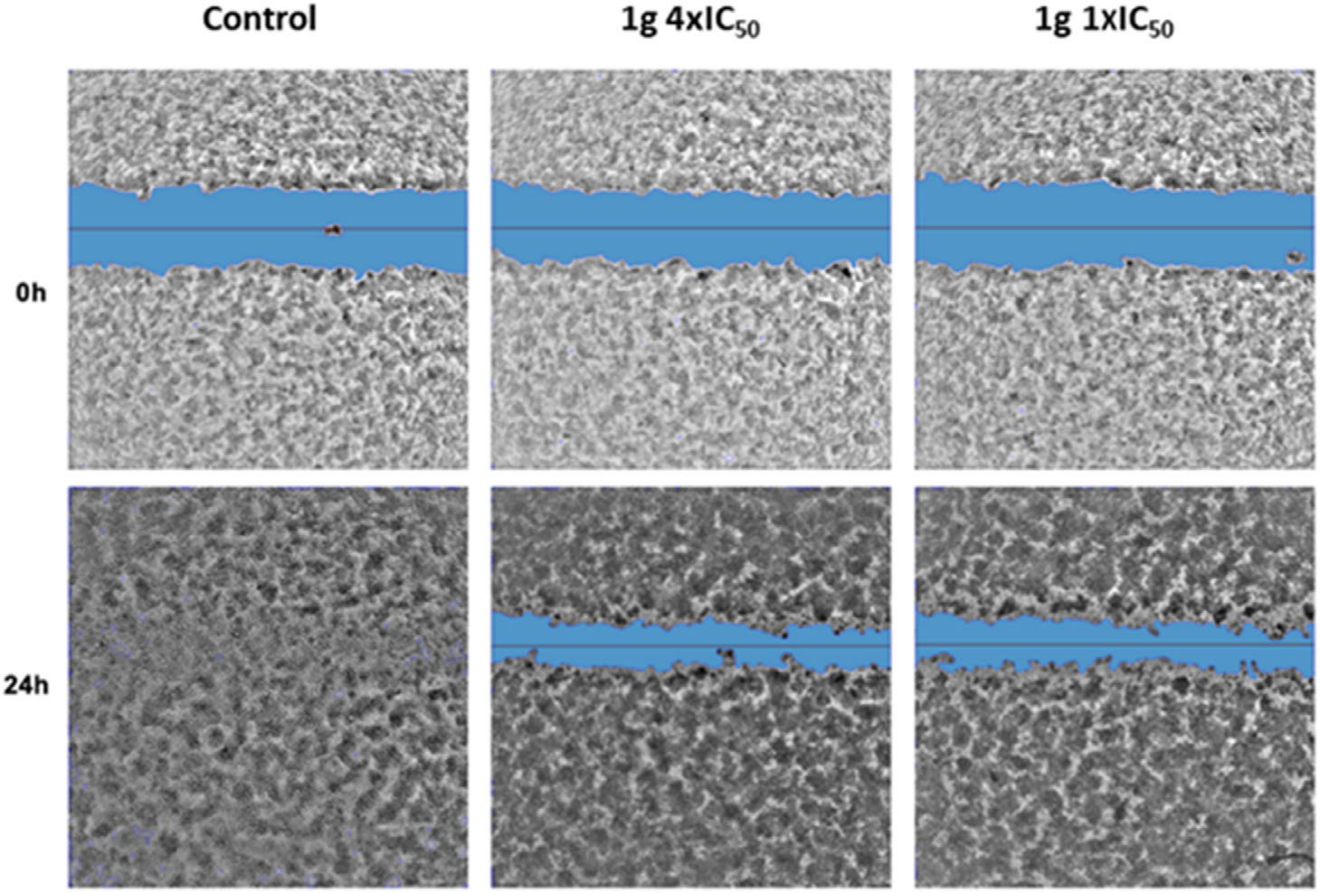
Representative images of migratory capacity of PATU‐T post‐treatment. Artificial wound area at 0 h compared to 24 h. The treated conditions with compound **1g** at both concentrations displayed impaired migration.

**Figure 4:**
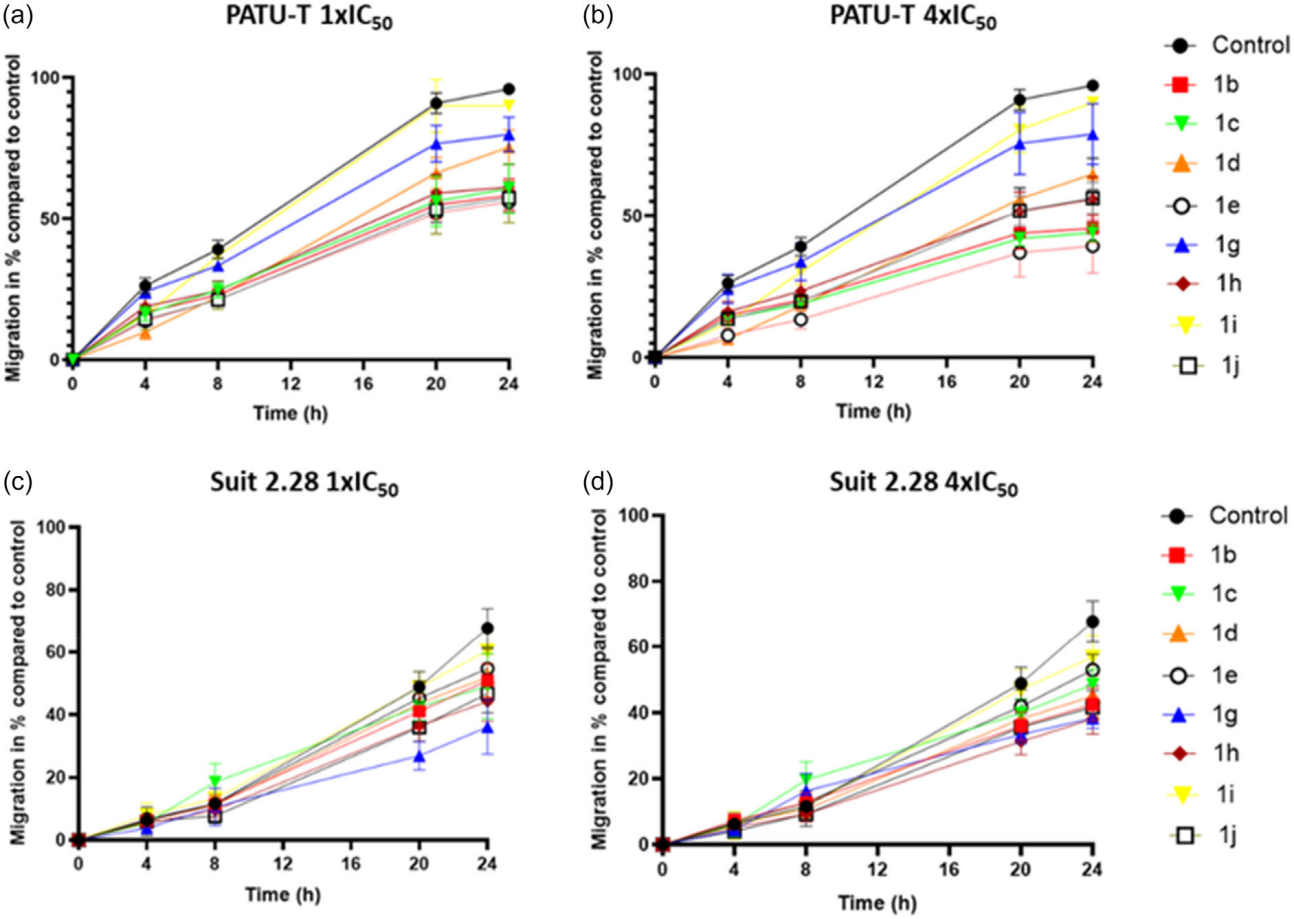
Migratory capacity of PATU‐T and SUIT 2.28 post‐treatment. Percentage of cell migration monitored over time (0, 4, 8, 20, and 24 h). The eight selected compounds were tested for migration inhibition in the PATU‐T and SUIT 2.28 cell line. a) Percent of migration of PATU‐T was significantly inhibited at 1× IC50 concentration and b) at 4× IC50 for all compounds as early as 4 h timepoint. Migration inhibition in SUIT 2.28 was only noticeable from the 20 h time point and was only significant at 24 h for both c) 1× IC50 and d) 4× IC50. The experiment was conducted in triplicates. Significance table is reported in Table S1 and S2, Supporting Information (****p < 0.0001).

A wound healing assay was conducted to investigate the effect of the selected compounds **1b, c–e, g,h‐j** on cell migration in the more mesenchymal and epithelial cells, i.e., PATU‐T and SUIT 2.28 cell lines. The compounds were tested at two different concentrations: 1 × IC_50_ and 4 × IC_50_. In the untreated PATU‐T cell line, the wound was completely closed in the control at 24 h compared to the treated conditions (**Figure** **3**). Results showed that both concentrations of the compounds significantly suppressed cell migration from the earliest time point of 4 h, with the effect persisting until 24 h (**Figure** **4**a,b). In PATU‐T, compounds **1b** and **1e** had the greatest inhibitory effect at both concentrations. In contrast, the SUIT 2.28 cell line did not show significant inhibitory effects at the earliest time points but exhibited a significant inhibition at 24 hr for the treated conditions at both concentrations compared to the control (Figure 4c,d). At the 1 × IC_50_ concentration, the greatest inhibition for SUIT 2.28 was observed with compound **1g**, aligning with previous IC_50_ measurement findings. Additionally, compound **1b**, which emerged as one of the most active compounds in PATU‐T, also showed strong inhibitory effects in SUIT 2.28. Based on the obtained IC_50_ values for all the different immortalized cell lines and the migration inhibition capacity of the compounds, the compounds **1b** and **1g** were selected for further analysis and testing in additional models.

### 2.4 Cell Viability and Migration Impact in Resistant and Primary Cell Lines

**Figure 5:**
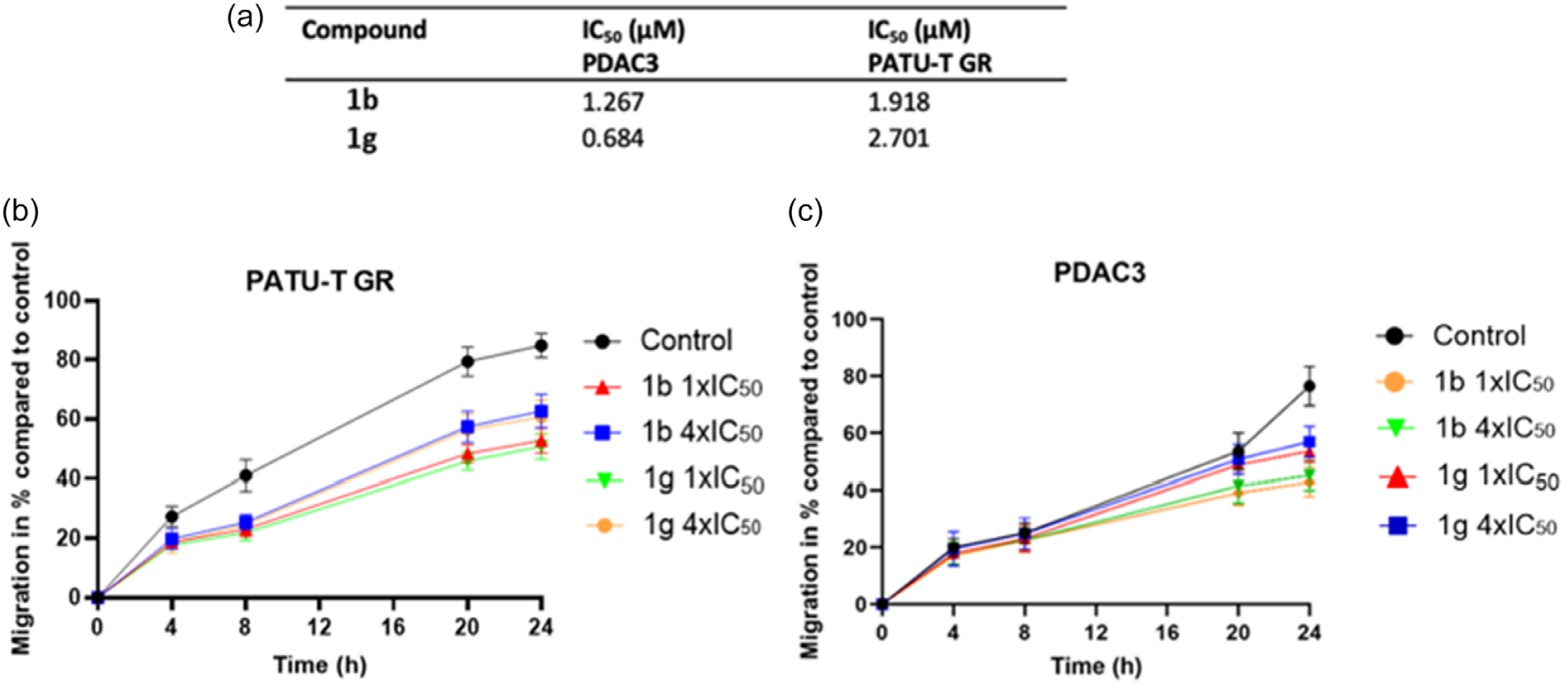
Evaluation of new models. IC_50_ measurement and percentage of migration overtime in PDAC3 and PATU‐T GR. a) IC_50_ values for compounds **1b** and **1g**. b) Migration inhibition in PATU‐T GR cell line. c) Migration inhibition in PDAC3 cell line. Significance table is reported in Table S3 and S4, Supporting Information (****p < 0.0001).

Further testing of the compounds **1b** and **1g** included two clinically relevant models, a gemcitabine resistant cell line (PATU‐T GR), and a primary cell line (PDAC3). SRB assays were performed in triplicate to calculate the IC_50_ values for both lines. The PDAC3 cell line showed remarkable sensitivity to these compounds, with IC_50_ values of 1.3 ± 0.2 µM for compound **1b** and 0.7 ± 0.1 µM for compound **1g** (**Figure** **5**a). The gemcitabine‐resistant PATU‐T GR line exhibited higher IC_50_ values for both compounds, compared to the wild‐type PATU‐T cell line. However, the cells still displayed good sensitivity, with IC_50_ values ranging from 1.9 ± 0.2 to 2.7 ± 0.5 µM. In addition, a wound healing assay was performed to evaluate migration capacity after treatment of these models. PATU‐T GR displayed inhibited migration abilities post‐treatment with both compounds at both 1× and 4 × IC_50_ concentrations. Similar to the wild‐type PATU‐T, PATU‐T GR migration was significantly inhibited as early as 4 h and continued to be inhibited throughout the 24‐hour period (Figure 5b). In contrast, the primary PDAC3 line showed significant migration inhibition only at the 24‐hour time point for both **1b** and **1g** inhibitors, with compound **1b** demonstrating a slightly better performance. (Figure 5c).

To better understand the performance of compounds **1b** and **1g** in PDAC, a commercially available CDK7 inhibitor, LDC4297, was purchased for comparison with these newly synthesized compounds. The IC_50_ value for LDC4297 was calculated for all PDAC cell lines used, as well as in pancreatic stellate cells (PSCs), which are nontumorous immortalized pancreatic fibroblasts. This model was employed to assess the cytotoxicity of our compounds, as well as LDC4297, on “healthy” cells. Compared to compounds **1b** and **1g**, LDC4297 was more effective on PDAC cell lines, with IC_50_ values ranging from 0.02 to 0.4 µM across the different cell lines (**Table** **3**). However, the low IC_50_ value for the PSC "healthy" line indicates that even small concentrations of LDC4297 can harm healthy tissue, not just tumorous cells. In contrast, compounds **1b** and **1g** exhibited higher IC_50_ values in the PSC cell line compared to tumor cells. This is favorable, as it suggests that higher doses of these compounds are required to damage healthy tissue.

**Table 3:** IC_50_ values of the inhibitor LDC4297 across all cell lines.

Compound	IC_50_ [μM]PDAC3a)	IC_50_ [μM]PATU‐GRa)	IC_50_ [μM]PSCa)	IC_50_ [μM]PATU‐Ta)	IC_50_ [μM]SUIT 2.28a)	IC_50_ [μM]PANC‐1a)
**1b**	1.3 ± 0.2	1.9 ± 0.2	6.6 ± 0.7	1.4 ± 0.2	0.6 ± 0.1	4.3 ± 0.8
**1g**	0.7 ± 0.1	2.7 ± 0.5	5.3 ± 1.1	1.3 ± 0.1	0.2 ± 0.1	2.9 ± 0.8
**LDC4297**	0.10 ± 0.02	0.45 ± 0.09	0.11 ± 0.02	0.03 ± 0.01	0.03 ± 0.01	0.02 ± 0.01

a) All values (average ± SEM) are reported in μM. Experiment conducted in triplicates.

### 2.5 Effects on Apoptosis

**Figure 6:**
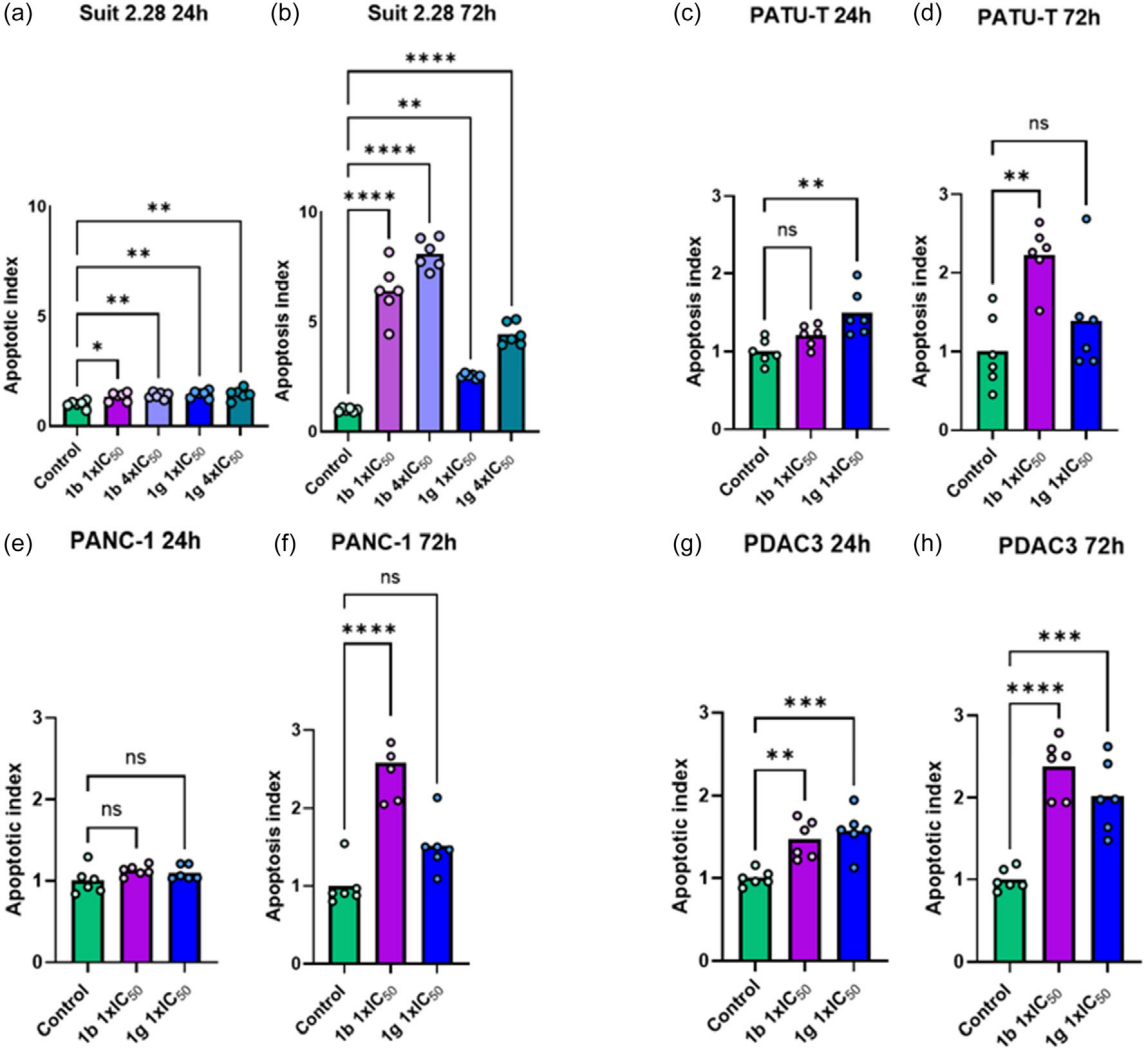
Effect of compounds **1b** and **1g** on apoptosis. Cell lines were exposed to treatment for either 24 or 72 h and their apoptotic index was assessed via Annexin V assay. a) SUIT 2.28 24 h post‐treatment; b) SUIT 2.28 72 h post‐treatment; c) PATU‐T 24 h post‐treatment; d) PATU‐T 72 h post‐treatment; e) PANC‐1 24 h post‐treatment; f) PANC‐1 72 h post‐treatment; g) PDAC3 24 h post‐treatment; h) PDAC3 72 h post‐treatment. ****p < 0.0001. ***p < 0.001. **p < 0.01. *p < 0.05.

To evaluate the apoptotic properties of compounds **1b** and **1g**, an Annexin V apoptosis assay was conducted for each cell line at two different time points: 24‐ and 72‐hours post‐treatment. The SUIT 2.28 cell line was the first to be tested, using both concentrations of 1× and 4 × IC_50_. In the 24 h experiment, both concentrations of both compounds showed significantly higher apoptotic index compared to untreated controls, indicating increased apoptosis in the treated cells, but the values were below 1.5, suggesting a minor biological impact (**Figure** **6**a). However, the 72 h exposure revealed an higher apoptotic index, especially for compound 1b at both concentrations, but also for compound 1g (Figure 6b). However, the 72 h exposure revealed an higher apoptotic index, especially for compound 1b at both concentrations, but also for compound 1g (Figure 6b). Given these findings, apoptosis analysis in the other cell lines was conducted at 1 × IC_50_, as it already showed significant differences from the control. However, the PATU‐T cell line showed a slightly different pattern for apoptosis. At the 24 h’ time point, compound **1b** did not show a significant apoptotic index compared to the control, while compound **1g** demonstrated significantly increased apoptosis in the treated versus control (Figure 6c). In contrast, at the 72 h’ time point, compound **1b** exhibited a significant increase in apoptotic index compared to control, suggesting that in the cells **1b** might require more time to induce apoptosis. Conversely, compound **1g**, which had a significant effect at 24 h, was not significant compared to control at this time at 72 h (Figure 6d). In PANC‐1, the conditions treated at 24 h did not show any significant increase in apoptotic index compared to the control (Figure 6e). However, at the 72‐hours’ time point, compound **1b** exhibited a significant increase in apoptotic index, while compound **1g** showed a slight increase that was not statistically significant (Figure 6f). Finally, the PDAC3 cell line appeared to be the most sensitive in this assay, showing a significant increase in apoptotic index at the earliest time point for both compounds (Figure 6g). This increase was further supported at the latest time point, where the apoptotic index continued to show a significant rise (Figure 6h).

**Figure 7:**
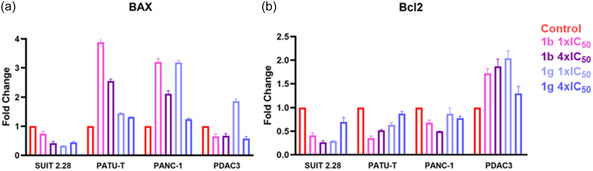
Apoptotic gene expression in PDAC cell lines. Gene expression was assessed 24 h after treatment with compounds **1b** and **1g**. a) Analysis of the proapoptotic gene BAX for each cell line. Gene expression was assessed 24 h after treatment with compounds **1b** and **1g**. b) Analysis of the antiapoptotic gene BCL‐2 for each cell line. All data are normalized to the GusB housekeeping gene and to their respective controls.

To validate the findings obtained from the Annexin V assay for each cell line, gene analysis was performed for proapoptotic and antiapoptotic genes. The pro‐apoptotic gene Bax was found to be upregulated in PATU‐T and PANC‐1 after treatment, especially with compound **1b** at 1 × IC_50_ concentration (**Figure** **7**a). Conversely, Bax was downregulated in the SUIT 2.28 and PDAC3 cell lines, except for PDAC3 treated with 1 × IC_50_ of compound **1g**. Additionally, the antiapoptotic gene Bcl‐2 was downregulated in all immortalized cell lines except the primary PDAC3 line, where Bcl‐2 was upregulated in all treated conditions compared to the control (Figure 7b). The Bax/Bcl‐2 ratio provides a way to assess cellular response to apoptotic stimuli, an increased Bax/Bcl‐2 ratio decreases cellular resistance to apoptotic stimuli, leading to increased cell death.^[^ ^34^ ^]^ Therefore, the ratio was calculated for all cell lines, as presented in **Table** **4**. The ratio values for all immortalized cell lines were above 1, indicating a positive Bax impact, leading to apoptosis. In contrast, PDAC3 showed ratio values below 1, suggesting that Bax does not have the same effect, and apoptosis might be regulated by other genes in these cells.

**Table 4:** Bax/Bcl‐2 ratio per cell line, used to estimate cell's life or death in response to an apoptotic stimulus.

Bax/Bcl‐2 ratio	SUIT 2.28	PATU‐T	PANC‐1	PDAC3
**1b 1×**	1.07	4.43	4.12	0.44
**1b 4×**	1.45	4.03	2.44	0.01
**1g 1×**	1.21	2.82	6.41	0.36
**1g 4×**	2.06	3.74	1.83	0.38

### 2.6 Gene Expression Analysis

**Figure 8:**
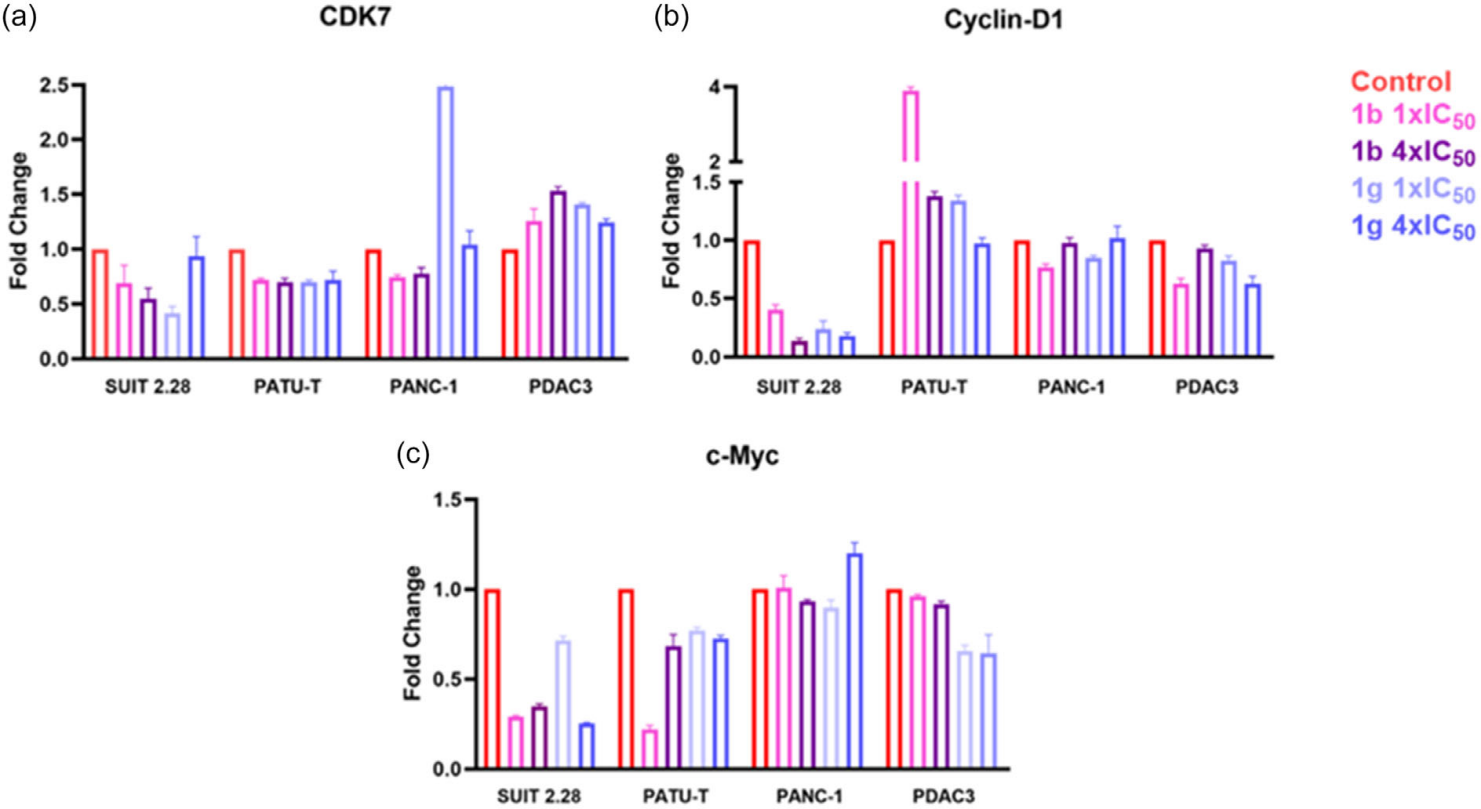
Gene expression analysis of CDK7 and two downstream genes. Gene expression was assessed 24 h after treatment with compounds **1b** and **1g**. a) Gene expression of CDK7 in all cell lines. b) Cyclin‐D1 gene expression analysis for all cell lines. c) c‐Myc gene expression analysis. All data are normalized to the GusB housekeeping gene and to their respective controls.

To further explore the effects of these novel compounds, gene expression of CDK7 was investigated. Gene expression was assessed 24 h after treatment with the compounds **1b** and **1g** at both 1× and 4 × IC_50_ concentrations. CDK7 expression was slightly lower for all the immortalized cell lines, excluding one treated condition with **1g** for the PANC‐1 cell line (Figure 7a). In contrast, CDK7 seems to be upregulated in PDAC3 (**Figure** **8**a). Cyclin‐D1, the first cyclin to be phosphorylated by the CAK complex, and a promoter of the G1 phase, was notably downregulated in SUIT 2.28, aligning with this cell line's high sensitivity to compounds 1b and 1g (Figure 8b). PANC‐1 and PDAC3 showed a comparable trend, with neither up‐ nor downregulation of the gene. However, PATU‐T displayed upregulation of Cyclin‐D1, suggesting that a 24 h treatment may not be sufficient to observe an effect on CDK7 inhibition (Figure 7b). Lastly, c‐Myc, a potent oncogene, exhibited downregulation in SUIT 2.28 and PATU‐T after treatment with either compound (Figure 7c). PDAC3 showed a downregulation only with compound **1g**, while PANC‐1 does not present any modulation of c‐Myc expression post‐treatment (Figure 8c).

### 2.7 Effect of Compounds 1b and 1g in Spheroidal Cultures

**Figure 9:**
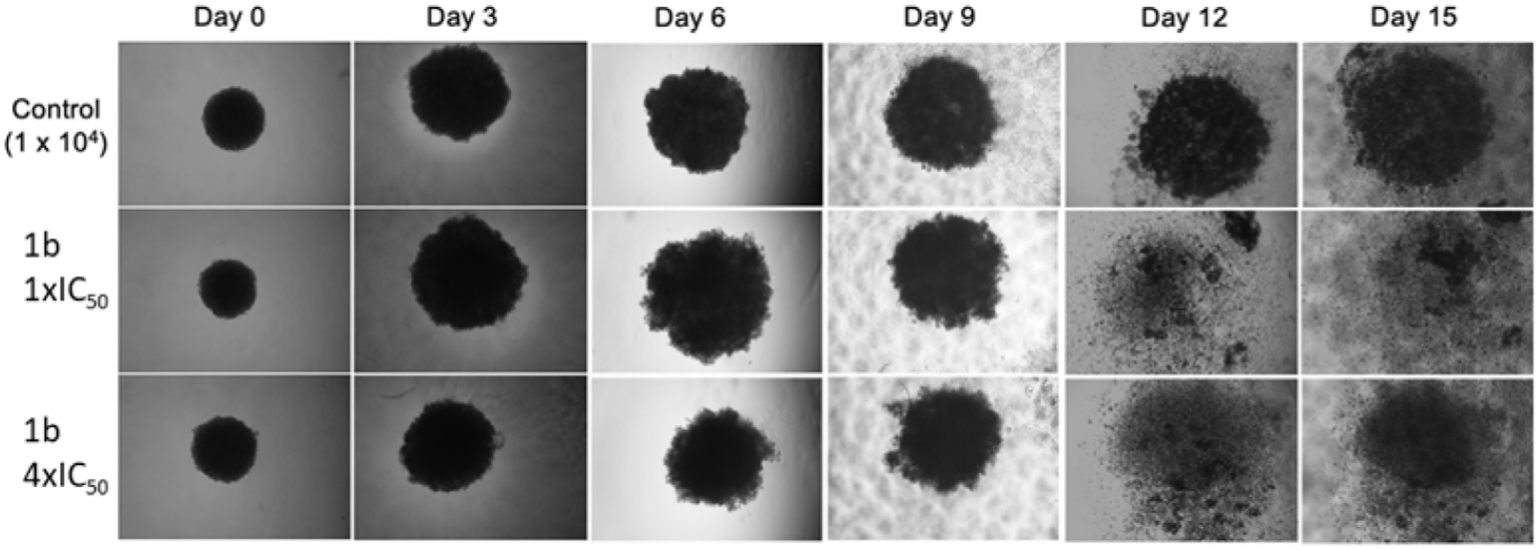
Representative images of impact of compound 1D in PATU‐T spheroids (1 × 10^3^). Compound **1g** at 1 × IC^50^ and 4 × IC^50^ was administered to the cells. These were monitored over 15 days, with images captured and media + treatment refreshed every 3 days. Objective: 4×; Calibration: 1.20 µm/px.

We evaluated the most promising compounds **1b** and **1g** on 3D spheroids of PATU‐T PDAC cells. To optimize the formation protocol, three different seeding densities (7 × 10^3^, 1 × 10^4^, and 2 × 10^4^ cells) were tested in agarose‐coated 96‐well plates. Spheroids were monitored over 15 days with imaging every 3 days, and the medium was refreshed periodically to prevent nutrient depletion and cell death. To evaluate the impact of compounds **1b** and **1g**, PATU‐T spheroids of varying densities were exposed to both compounds at concentrations of 1 × IC_50_ and 4 × IC_50_. **Figure** **9** illustrates the effect of compound **1b** on spheroids seeded at 1 × 10^4^ density, where both drug concentrations caused the spheroids to begin flaking by day 12.

**Figure 10:**
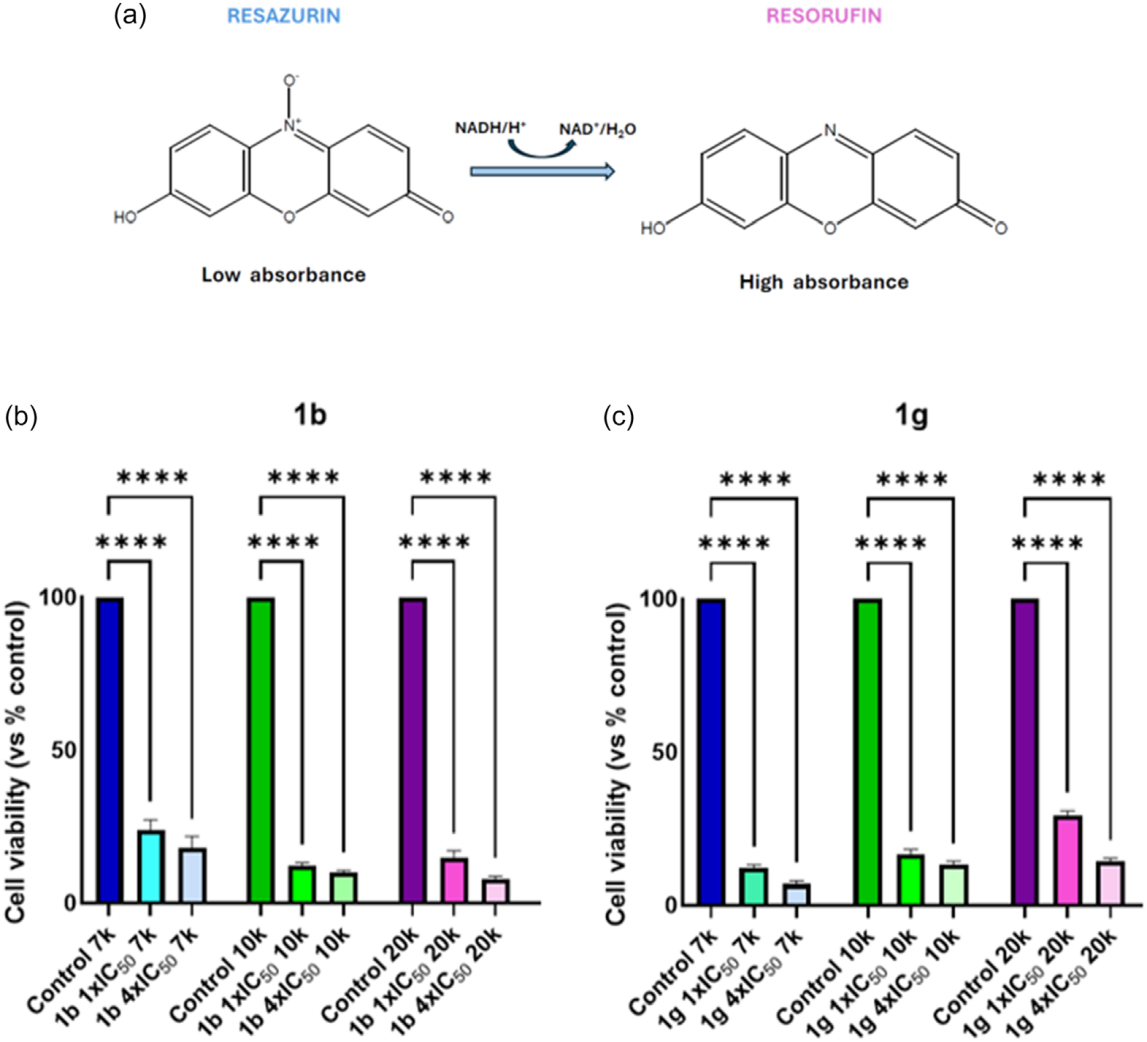
Resazurin assay of PATU‐T cells. a) Resazurin assay principle. b) Percent of cell viability compared to control in three different cell densities treated with compound **1b** at 1× and 4 × IC_50_ concentrations. c) % of cell viability compared to control in three different cell densities treated with compound **1g** at 1× and 4 × IC_50_ concentrations. ****p < 0.0001. ***p < 0.001. **p < 0.01. *p < 0.05.

To quantitatively assess cellular viability post‐treatment, cells were subsequently reseeded into standard 96‐well plates and stained with resazurin on day 7. The resazurin assay relies on the principle that ongoing cell proliferation maintains a reduced environment, while growth inhibition results in an oxidized environment.^[^ ^35^ ^]^ This reduction triggers the transformation of the redox indicator from its nonfluorescent, blue oxidized form to its fluorescent, red reduced form (**Figure** **10**a). Notably, cells treated with either compound **1b** or **1g** exhibited significantly reduced viability compared to their untreated controls across all cell densities (Figure 10b,c). This indicates that compounds **1b** and **1g** effectively inhibited cell proliferation in the spheroids.

### 2.8 Modulation of the Cell Cycle

The effects of the most effective compounds, **1b** and **1g,** on cell cycle progression were investigated in both immortalized and primary cell lines using cytofluorimetric analysis with propidium iodide (PI) staining. The results revealed that compounds **1b** and **1g** induced changes in the distribution of cells across the cell‐cycle phases.

**Figure 11:**
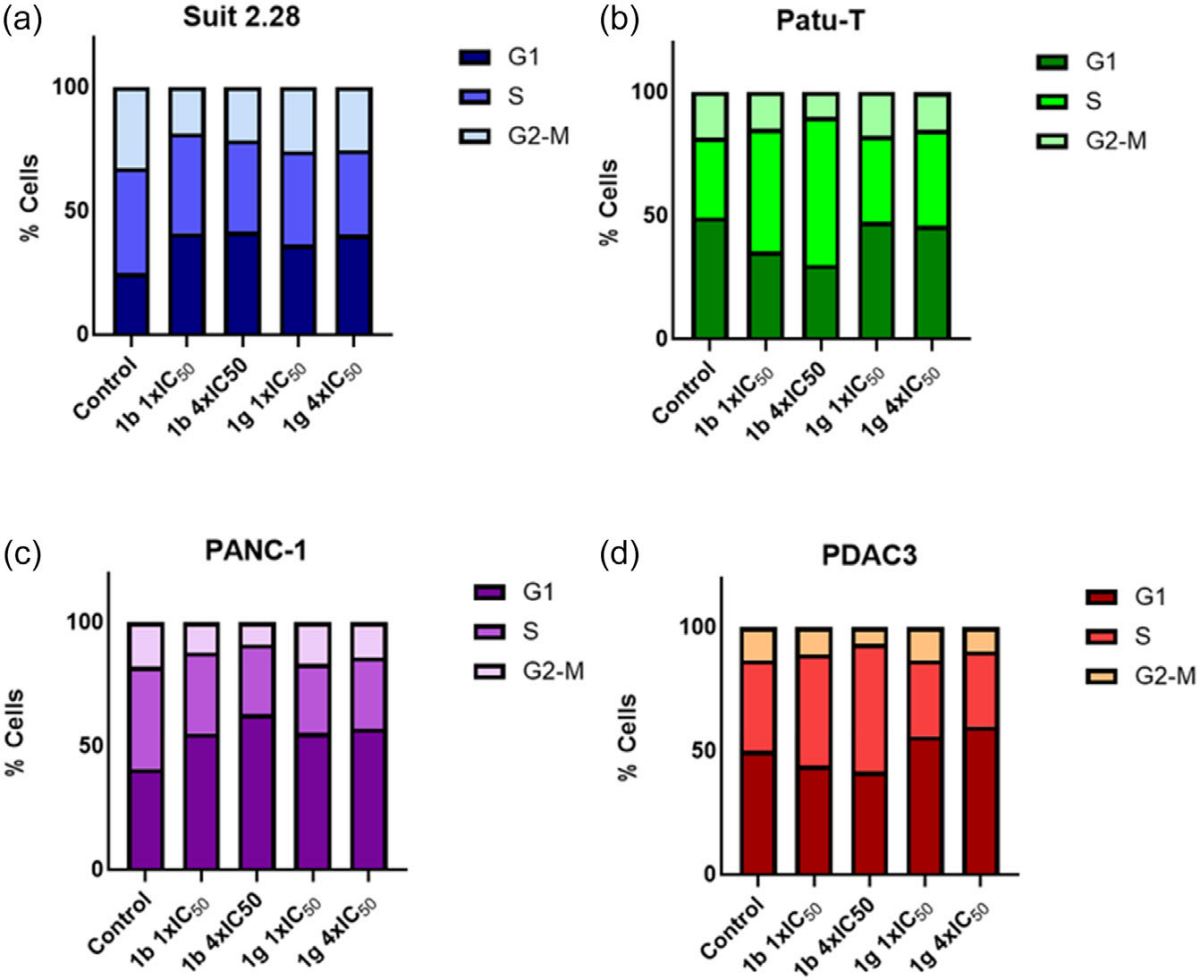
Modulation of the cell cycle in PDAC cell lines post treatment with **1b** and **1g** compounds. Cells were exposed to treatment for 24 h and columns show the mean percentages of cells at various stages of the cell cycle. a) Cell cycle effects induced by compounds **1b** and **1g** in SUIT 2.28 cells. b) Cell cycle effects induced by compounds **1b** and **1g** PATU‐T cells. c) Cell cycle effects induced by compounds **1b** and **1g** in PANC‐1 cells. d) Cell cycle effects induced by compounds **1b** and **1g** in PDAC3 cells.

SUIT 2.28 treated with compound **1b** at 1× and 4 × IC_50_ concentrations exhibited an increase in the percentage of cells in the G0‐G1 phase from 24,6% to 41.0% and 41.6%, respectively. In contrast the percentage of cells in the S phase decreased from 42.7% to 40.3% and 34.9%. Similarly, there was a decrease in the percentage of cells in the G2‐M phase from 32.7% to 18.7% and 21.6%. SUIT 2.3 cells treated with compound **1g** at 1× and 4 × IC_50_ concentrations exhibited an increase in the percentage of cells in the G0‐G1 phase from 24.6% to 36.2% and 40.3%, respectively. Conversely, the percent of cells in the S phase decreased from 42.7% to 37.8% and 34.2%. Similarly, there was a decrease in the percentage of cells in the G2‐M phase from 32.7% to 25.9% and 25.4% (**Figure** **11**a).

In PATU‐T cells treated with 1× and 4 × IC_50_ concentrations of **1b**, the G0‐G1 phase decreased from 49.1% to 35.5% and 30.0%, respectively, while the S phase increased from 32.5% to 49.6% and 60.0%. There was a decrease in the percent of cells in the G2‐M phase from 18.4% to 14.9% and 10.1%. PATU‐T cells treated with 1 × IC_50_ and 4 × IC_50_ concentrations of **1g** exhibited a decrease in the percentage of cells in the G0‐G1 phase from 49.1% to 47.4% and 45.7%, respectively. In contrast, the number cells in the S phase increase from 32.5% to 35.0% and 39.0%. There was a decrease in the percentage of cells in the G2‐M phase from 18.4% to 17.7% and 15.2% (Figure 11b).

PANC‐1 cells treated with 1× and 4 × IC_50_ concentrations of **1b** displayed an increase in the amount of cells in the G0‐G1 phase from 40.7% to 55.0% and 62.8%, respectively. Conversely, the percent of cells in the S phase decreased from 41.3% to 32.7% and 28.3%. Similarly, the percent of cells in the G2‐M phase decreased from 18.09% to 12.2% and 10.0%. PANC‐1 cells treated with 1× and 4 × IC_50_ concentrations of **1g** exhibited an increase in the percentage of cells in the G0‐G1 phase from 40.7% to 55.2% and 56.9%, respectively. Whereas, the percent of cells in the S phase decrease from 41.3% to 27.9% and 29.0%. There was a decrease in the number of cells in the G2‐M phase from 18.1% to 16.9% and 14.1% (Figure 11c).

Finally, PDAC3 cells treated with 1 × IC_50_ and 4 × IC_50_ concentrations of **1b** exhibited a decrease in the amount of cells in the G0‐G1 phase from 50.0% to 44.0% and 41.6%, respectively. In contrast, the percent of cells in the S phase increased from 36.7% to 44. 9% and 51.6%. In contrast, there was a decrease in the percentage of cells in the G2‐M phase from 13.3% to 11.1% and 6.9%. PDAC3 cells treated with 1‐fold IC_50_ and 4‐fold IC_50_ concentrations of **1g** exhibited an increase in the percentage of cells in the G0‐G1 phase from 50.0% to 55.9% and 59.8%, respectively. In contrast the percentage of cells in the S phase decreased from 36.7% to 30.8% and 30.4%. Moreover, there was a decrease in the percentage of cells in the G2‐M phase from 13.3% to 13.3% and 9.8% (Figure 11d).

These findings suggest that compounds **1b** and **1g** may exert its effect by inhibiting protein kinases involved in the cell cycle process.

### 2.9 Profiling of Kinase Inhibition as Assessed by Pamgene Array

**Figure 12:**
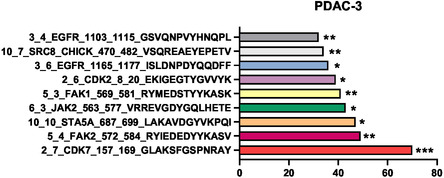
Average kinase inhibition in untread cells compared to cells treated with IC50 of compound **1b**. *P* values were calculated with student's *t*‐test. ***p < 0.001, **p < 0.01, *p < 0.05.

PDAC‐3 primary cells were selected for kinase activity profiling, as they represent a clinically relevant model of PDAC. These cells were exposed to compound **1b** at its IC50_50_ concentration for 2 h. Following treatment, lysates from both treated and control cells were applied to porous kinase arrays in the presence of ATP to facilitate kinase‐driven phosphorylation of peptide substrates. The assay included a fluorescein isothiocyanate‐labeled antibody to enable detection of phosphorylation events, captured using a charge‐coupled device (CCD) camera. Compound **1b** demonstrated significant inhibition of phosphorylation across 9 peptide substrates, with reduction levels ranging from 70% to 32% compared to untreated controls (**Figure** **12**). Notably, the most pronounced inhibition was observed for CDK7, with a 70% decrease, markedly exceeding that of the other kinases, all of which exhibited inhibition below 50%. Of note, several of these affected kinases, such as EGFR, JAK2, and SRC, are functionally associated with CDK7 through transcriptional regulation, signaling crosstalk, or oncogenic codependencies ref. [12], and further investigation into these relationships is warranted.

These findings strongly support the hypothesis that CDK7 inhibition represents a key mechanism of action for compound 1b. To confirm this and to evaluate whether a similar effect is observed with compound 1g and in an additional cellular model, a targeted ELISA assay was performed, as detailed in the following section.

### 2.10 Inhibition of CDK7 as Assessed by ELISA

**Figure 13:**
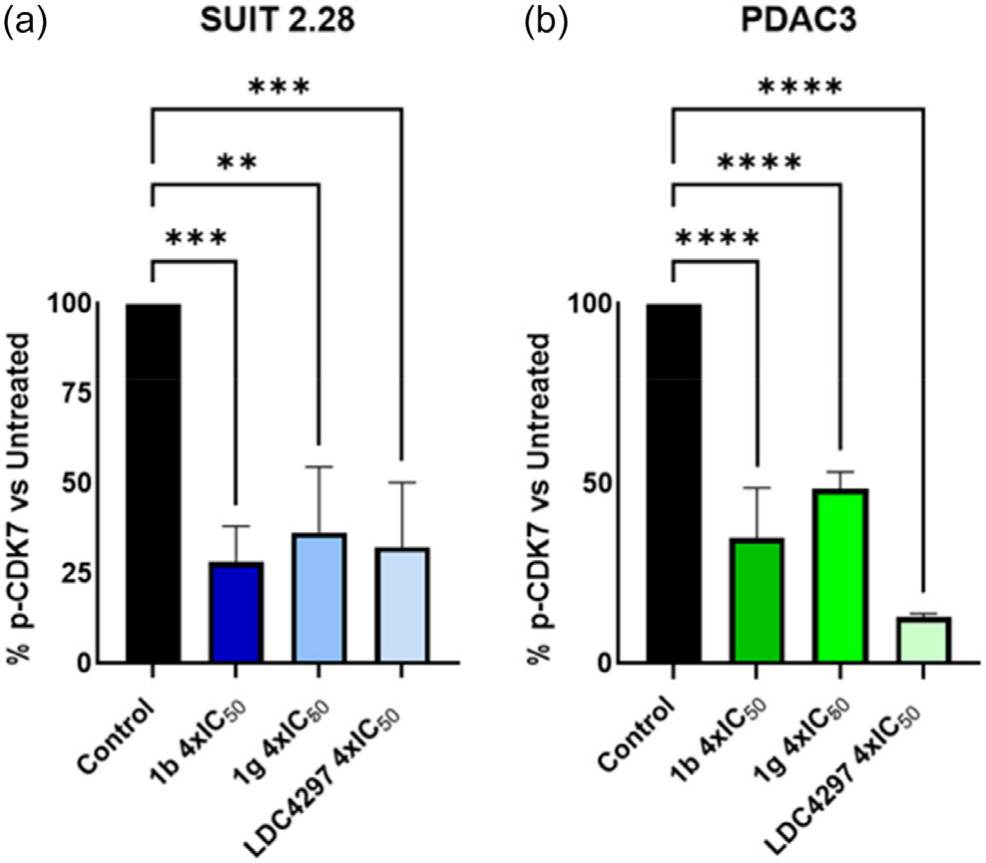
Modulation of p‐CDK7 expression by ELISA in SUIT 2.28 and PDAC3. a) % of p‐CDK7 in treated versus untreated condition in SUIT 2.28. b) % of p‐CDK7 in treated versus untreated condition in PDAC3. ****p < 0.0001. ***p < 0.001. **p < 0.01. *p < 0.05.

A specific ELISA assay was conducted to determine phosphorylated CDK7 (p‐CDK7) expression in two PDAC preclinical models (SUIT 2.28 and PDAC3) treated with 4 × IC_50_ of compounds **1b**, **1g** and the known CDK7 inhibitor LDC4297. The findings revealed a decrease in p‐CDK7 expression in both models following treatment with either **1b** or **1g** (**Figure** **13**). Compound LDC4297 also exhibited a significant decrease in p‐CDK7 levels compared to control. Particularly, in SUIT 2.28 its effect was comparable to that of both compounds **1b** and **1g**, with **1b** demonstrating a more pronounced reduction of p‐CDK7 levels (Figure 13a). Whereas, in PDAC3, LDC4297 appeared more effective in inhibiting CDK7, as its levels were lower when compared to treatment with **1b** and **1g** (Figure 13b). These results suggest that compounds **1b** and **1g** exert their anticancer effects by targeting CDK7 and inhibiting its activation.

### 2.11 In Vitro CDK7 Enzyme Inhibition Assay

**Figure 14:**
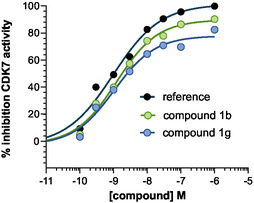
CDK7 activity inhibition assessed by an in vitro enzymatic assay. Data points represent the mean values from triplicate experiments.

The candidate compounds **1b** and **1g** were evaluated for their in vitro CDK7 enzyme inhibition activity using the ADP‐Glo assay, as described previously.^[^ ^36^ ^]^ Dose–response curves were generated to determine mean IC_50_ values, as reported in **Figure** **14**.

The results showed that compound **1b** exhibited the highest CDK7 inhibition activity (IC_50_ = 25 nM), which was not significantly different from the reference inhibitor (LDC4297, IC_50_ = 7.5 nM). Similarly, compound **1g** demonstrated statistically nonsignificant inhibition (IC_50_ = 28 nM) compared to the reference compound.

Considering the structural similarity of our compounds to LDC4297 and ICEC0942, ATP‐competitive CDK7 inhibitors,^[^ ^37^ ^]^ we hypothesize that our compounds engage CDK7 through a similar ATP‐competitive binding mode. Further structural or biochemical studies will be necessary to confirm this hypothesis.

## 3 Conclusions

A new series of indolyl and 7‐aza‐indolyl pyrazolo [1,5‐a]−1,3,5‐triazine derivatives was efficiently synthesized. 8 out of 33 derivatives showed remarkable cytotoxicity against a panel of PDAC cells, namely SUIT 2.28, PATU‐T, PANC‐1, with IC_50_ values ranging from 0.19 to 1.58 µM and remarkable inhibition of cell migration from the earliest time point of 4 h, persisting until 24 h. The two most active compounds were further investigated in additional clinically relevant models (PATU‐T GR, PDAC3) confirming their significant activity with IC_50_ values ranging from 0.68 to 2.70 µM. Annexin apoptosis assay demonstrated the apoptosis induction properties of the compounds with upregulation of apoptotic genes Bax. They showed significant reduced viability of spheroidal PATU‐T cultures, inhibiting their proliferation at 1× and 4 × IC_50_ concentrations. Cytofluorimetric analysis revealed that the compounds induced changes in the distribution of cells across the cell cycle phases; meanwhile, Pamgene and ELISA assays showed a decrease in phosphorylated CDK7 expression. CDK7 inhibition was further confirmed through a specific in vitro enzymatic assay, where the newly synthesized compounds elicited IC_50_ values in the nanomolar range.

## 4 Experimental Section

4.1

4.1.1

##### Chemistry

All materials and solvents were purchased from commercial sources and used without further purification. All melting points were obtained on a Büchi‐Tottoly capillary apparatus (Büchi, Cornaredo, Italy) and have not been corrected. IR spectra were determined in bromoform with a Shimadzu FT/IR 8400S spectrophotometer (Shimadzu Corporation, Milan, Italy). ^1^H and ^13^C spectra were measured at 200 and 50 MHz, respectively in DMSO‐*d*_6_ solution, using a Bruker Avance II series 200 MHz spectrometer (Bruker, Milan, Italy). Column chromatography was performed with Merck silica gel 230–400 mesh ASTM or with a Büchi Sepacor chromatography module (prepacked cartridge system). Elemental analyses (C, H, N) were within ±0.4% of theoretical values and were performed with a VARIO EL III elemental analyzer. The HRMS of the final compounds **1b** and **1g** acquired using a Thermo Q‐Exactive system revealed a purity exceeding 95% and were reported in SI.

##### General Procedure for the Synthesis of 3‐(1‐methyl‐7‐azaindol‐3‐yl)‐3‐oxopropanenitriles (14a,b)

A solution of cyanoacetic acid (7 mmol) and acetic anhydride (Ac_2_O) (7 mL) was heated at 85 °C for 10 min. Then appropriate 1‐methyl‐7‐azaindole (**13a, b**) (7 mmol) was added to this solution and heating was continuing at 85 °C for 5–24 h. After completion the reaction mixture was left to cool and was poured into water and ice. The obtained precipitate was filtered off, dried and purified by column chromatography, using cyclohexane/ethyl acetate as eluent to obtain the desired products (**14a, b**).

##### 3‐(1‐Methyl‐7‐azaindol‐3‐yl)‐3‐oxopropanenitrile (14a)

Condition: 5 h 85 °C. Yield: 48%; yellow solid; m.p.: 209.6–210.3 °C; IR (cm^−1^): 2255 (CN), 1653 (CO); ^1^H NMR (200 MHz, DMSO*‐d*_6_) *δ*: 3.90 (s, 3H, CH_3_). 4.50 (s, 2H, CH_2_), 7.34 (dd, *J =* 7.7, 4.8 Hz, 1H, H‐5), 8.43 (t, *J =* 6.0 Hz, 2H, H‐4, H‐6), 8.58 (s, 1H, H‐2); ^13^C NMR (50 MHz, DMSO*‐d*_6_) *δ*: 29.3 (t), 31.7 (q), 111.7 (s), 116.1 (s), 117.8 (s), 118.8 (d), 129.6 (d), 138.8 (d), 144.5 (d), 148.0 (s), 182.7 (s); *Anal.* Calculated for C_11_H_9_N_3_O (MW: 199,21): C, 66.32; H, 4.55; N, 21.09%. Found: C, 66.41; H, 4.67; N, 21.20%.

##### 3‐(5‐Bromo‐1‐methyl‐7‐azaindol‐3‐yl)‐3‐oxopropanenitrile (14b)

Condition: overnight 85 °C. Yield: 45%; light yellow solid; m.p.: 238.6–239.6 °C; IR (cm^−1^): 2257 (CN), 1653 (CO); ^1^H NMR (200 MHz, DMSO*‐d*_6_) *δ*: 3.87 (s, 3H, CH_3_). 4.51 (s, 2H, CH_2_), 8.47 (d, *J* = 2.1 Hz, 1H, H‐4), 8.49 (d, *J* = 2.1 Hz, 1H, H‐6), 8.62 (s, 1H, H‐2); ^13^C NMR (50 MHz, DMSO*‐d*_6_) *δ*: 29.97 (t) 32.4 (q), 111.5 (s), 114.7 (s), 116.3 (s), 119.7 (s), 131.6 (d), 140.4 (d), 145.1 (d), 146.9 (s), 183.28 (s); *Anal.* Calculated for C_11_H_8_BrN_3_O (MW: 278,11): C, 47.51; H, 2.90; N, 15.11%. Found: C, 47.67; H, 2.99; N, 15.25%.

##### General Procedure for the Synthesis of 3‐(1H‐indol‐3‐yl)‐1H‐pyrazol‐5‐amines (9a, b)

To a mixture of appropriate 3‐(1‐methyl‐7‐azaindol‐3‐yl)‐3‐oxopropanenitriles (**14a, b**) (5.45 mmol) and acethyl hydrazide (AcNHNH_2_) (6 mmol) in anhydrous *n*‐butanol (*n*‐BuOH) (30 mL), *p*‐toluenesulfonic acid monohydrate (PTSA·H_2_O) (6 mmol) was added. The reaction mixture was heated vigorously under reflux for 45 min^−^^1^ h by using the Marcusson apparatus. After completion the reaction mixture was cooled. The solvent was removed in vacuo. The resulting residue was neutralized by adding a few drops of saturated aqueous solution of sodium hydrogencarbonate (NaHCO_3_), extracted with water (20 mL) and ethyl acetate (3 × 20 mL), dried (Na_2_SO_4_) and evaporated under reduced pressure. The product obtained was further purified by column chromatography, using ethyl acetate as eluent.

##### 3‐(1‐Methyl‐1H‐pyrrolo[2,3‐b]pyridin‐3‐yl)‐1H‐pyrazol‐5‐amine (9a)

Condition: 45 min reflux. Yield: 49%; brown solid; m.p: 181.4–182 °C; IR (cm^−1^): 3468 (NH_2_), 3365 (NH); ^1^H NMR (200 MHz, DMSO‐*d*_6_) *δ*: 3.80 (s, 3H, CH_3_), 4.87 (br s, 2H, NH_2_), 5.79 (br s, 1H, pyrazole‐CH), 7.10 (t, *J* = 7.4 Hz, 1H), 7.19 (d, *J* = 8.5 Hz, 1H), 7.45 (d, *J* = 8.2 Hz, 1H), 7.88 (br s, 1H), 11.49 (br s, 1H, NH); ^13^C NMR (50 MHz, DMSO‐*d*_6_) *δ*: 33.0 (q), 110.4 (d), 119.9 (d), 120.5 (d), 121.9 (d), 124.4 (s), 125.5 (s), 127.3 (d), 133.4 (s), 135, 5 (s), 137.2 (s); *Anal.* Calculated for C_11_H_11_N_5_ (MW: 213,24): C, 61.96; H, 5.20; N, 32.84%. Found: C, 61.82; H, 5.36; N, 32.68%.

##### 3‐(5‐Bromo‐1‐methyl‐1H‐pyrrolo[2,3‐b]pyridin‐3‐yl)‐1H‐pyrazol‐5‐amine (9b)

Condition: 1 h reflux. Yield: 55%; brown solid; m.p: 191–195.6 °C; IR (cm^−1^): 3456 (NH_2_), 3298 (NH); ^1^H NMR (200 MHz, DMSO‐*d*_6_) *δ*: 3.80 (s, 3H, CH_3_), 4.83 (br s, 2H, NH_2_), 5.63 (br s, 1H, pyrazole‐CH), 7.44 (s, 1H,), 7.63 (s, 1H), 8.14 (br s, 1H,), 11.67 (br s, 1H, NH); ^13^C NMR (50 MHz, DMSO‐*d*_6_) *δ*: 33.1 (q), 112.4 (d), 112.6 (s), 119.4 (s), 120.5 (d), 124.3 (d), 126.3 (s) 127.2 (s), 128.8 (d), 135.9 (s), 136.9 (s); Anal. Calculated for C_11_H_10_BrN_5_ (MW: 292,13): C, 45.22; H, 3.45; N, 23.97%. Found: C, 45.08; H, 3.31; N, 23.83%.

##### General Procedure for the Synthesis of N′‐[3‐(1H‐indol‐3‐yl)‐1H‐pyrazol‐5‐yl]‐N,N‐dimethylmethanimidamides (10a‐e)(11a‐e)(12a,b)

To a solution of the opportune amine **7–9**, (5.43 mmol) in anhydrous 1,4 dioxane, DMF‐DMA (0.868 mL) was added. The reaction mixture was heated vigorously under reflux for 1–6 h (the progress of the reaction was monitored by TLC). After completion, the reaction mixture was left to cool, and the obtained precipitate was filtered off, without further purification.

##### N′‐[3‐(1H‐indol‐3‐yl)‐1H‐pyrazol‐5‐yl]‐N,N‐dimethylmethanimidamide (10a)

Condition: 2.5 h reflux. Yield: 53%; white solid; m.p.: 177–179 °C. IR (cm^−1^): 3266 (NH), 3167 (NH); ^1^H NMR (200 MHz, DMSO‐*d*_6_) *δ*: 2.92 (s, 3H, CH_3_), 3.02 (s, 3H, CH_3_), 6.16 (s, 1H, pyrazole‐CH), 7.05 (t, *J* = 7.3 Hz, 1H), 7.11 (t, *J* = 7.4 Hz, 1H), 7.39 (d, *J* = 8.0 Hz, 1H), 7.60 (s, 1H), 8.01 (s, 1H), 8.05 (s, 1H), 11.15 (br s, 1H, NH), 11.93 (br s, 1H, NH). ^13^C NMR (50 MHz, DMSO‐*d*_6_) *δ*: 21.2 (qx2), 112.0 (d), 119.6 (d), 120.8 (s), 121.8 (d), 122.9 (d), 125.3 (s), 126.0 (d), 128.5 (d), 136.8 (s), 138.1 (s), 146.1 (s), 155.3 (d); *Anal.* Calculated for C_14_H_15_N_5_ (MW: 253.30): C, 66.38; H, 5.97; N, 27.65%. Found: C 66.24; H, 5.83; N, 27.51%.

##### N′‐[3‐(5‐bromo‐1H‐indol‐3‐yl)‐1H‐pyrazol‐5‐yl]‐N,N‐dimethylmethanimidamide (10b)

Condition: 2 h reflux. Yield: 77%; white solid; m.p.: 220–221.9 °C. IR (cm^−1^): 3399 (NH), 3254 (NH); ^1^H NMR (200 MHz, DMSO‐*d*_6_) *δ*: 2.92 (s. 3H, CH_3_), 3.02 (s, 3H, CH_3_), 6.10 (s, 1H, pyrazole‐CH), 7.22 (d, *J* = 7.6 Hz, 1H), 7.36 (d, *J* = 8.1 Hz, 1H), 7.63 (s, 1H), 8.04 (s, 1H), 8.32 (s, 1H), 11.29 (br s, 1H, NH), 11.94 (br s, 1H, NH). ^13^C NMR (50 MHz, DMSO‐*d*_6_) *δ*: 34.3 (qx2), 86.4 (d), 111.1 (s), 112.1 (s), 113.9 (d), 123.8 (d), 124.1 (d), 124.4 (d), 127.2 (s), 135.5 (s), 135.6 (s), 153.0 (s), 155.7 (d); *Anal.* Calculated for C_14_H_14_BrN_5_ (MW: 332.20): C, 50.62; H, 4.25; N, 21.08%. Found: C, 50.58; H, 4.41; N, 21.24%.

##### N′‐[3‐(5‐chloro‐1H‐indol‐3‐yl)‐1H‐pyrazol‐5‐yl]‐N,N‐dimethylmethanimidamide (10c)

Condition: 1 h reflux. Yield: 89%; white solid; m.p.: 183.2–186 °C. IR (cm^−1^): 3402 (NH), 3153 (NH); ^1^H NMR (200 MHz, DMSO‐*d*_6_) *δ*: 2.92 (s, 3H, CH_3_), 3.02 (s, 3H, CH_3_), 6.13 (s, 1H. pyrazole‐CH), 7.11 (d, *J* = 8.4 Hz, 1H), 7.41 (d, *J* = 8.6 Hz, 1H), 7.67 (s, 1H), 8.05 (s, 1H), 8.13 (s, 1H), 11.31 (br s, 1H, NH), 11.96 (br s, 1H, NH). ^13^C NMR (50 MHz, DMSO‐*d*_6_) *δ*: 34.3 (qx2), 113.4 (d), 113.5 (d), 121.7 (d), 124.3 (s), 124.6 (d), 124.7 (d), 126.4 (s), 135.3 (3xs), 155.7 (d); *Anal.* Calculated for C_14_H_14_ClN_5_ (MW: 287.75): C, 58.44; H, 4.90; N, 24.34%. Found: C, 58.24; H, 4.76; N, 24.12%.

##### N′‐[3‐(5‐fluoro‐1H‐indol‐3‐yl)‐1H‐pyrazol‐5‐yl]‐N,N‐dimethylmethanimidamide (10d)

Condition: 3 h reflux. Yield: 62%; white solid; m.p.: 215.6 °C. IR (cm^−1^): 3338 (NH), 3184 (NH); ^1^H NMR (200 MHz, DMSO‐*d*_6_) *δ*: 2.92 (s, 3H, CH_3_), 3.02 (s, 3H, CH_3_) 6.11 (s, 1H, pyrazole‐CH), 6.95 (s, 1H), 7.38 (s, 1H), 7.65 (s, 1H), 8.05 (s, 1H), 11.30 (br s, 1H, NH), 11.96 (br s, 1H, NH). *Anal.* Calculated for C_14_H_14_FN_5_ (MW: 271.29): C, 61.98; H, 5.20; N, 25.81%. Found: C, 61.84; H, 5.06; N, 25.67%.

##### N′‐[3‐(5‐methoxy‐1H‐indol‐3‐yl)‐1H‐pyrazol‐5‐yl]‐N,N‐dimethylmethanimidamide (10e)

Condition: 6 h reflux. Yield: 73%; white solid; m.p.: 199.5–200 °C. IR (cm^−1^): 3375 (NH), 3207 (NH); ^1^H NMR (200 MHz, DMSO‐*d*_6_) *δ*: 2.92 (s, 3H, CH_3_), 3.01 (s, 3H, CH_3_), 3.79 (s, 3H, OCH_3_), 6.08 (s, 1H, pyrazole‐CH), 6.76 (d, *J* = 8.2 Hz, 1H), 7.28 (d, *J* = 8.3 Hz, 1H), 7.63 (s, 1H), 7.54 (s, 1H), 8.03 (s, 1H), 11.02 (br s, 1H, NH), 11.90 (br s, 1H, NH). ^13^C NMR (50 MHz, DMSO‐*d*_6_) *δ*: 34.3 (qx2), 55.8 (q), 86.4 (d), 103.1 (s), 112.0 (d), 112.6 (d), 112.6 (d), 112.8 (s), 123.6 (d), 125.7 (s), 132.0 (s), 132.1 (s), 154.1 (s), 155.6 (d); *Anal.* Calculated for C_15_H_17_N_5_O (MW: 283.33): C, 63.59; H, 6.05; N, 24.72; Found: C, 63.45; H, 6.21; N, 24.58; %.

##### N,N‐dimethyl‐N′‐[3‐(1‐methyl‐1H‐indol‐3‐yl)‐1H‐pyrazol‐5‐yl]methanimidamide (11a)

Condition: 1 h reflux. Yield: 50%; white solid; m.p.: 195 °C. IR (cm^−1^): 3177 (NH); ^1^H NMR (200 MHz, DMSO‐*d*_6_) *δ*: 2.92 (s, 3H, CH_3_), 3.01 (s, 3H, CH_3_), 3.80 (s, 3H, CH_3_), 6.13 (s, 1H, pyrazole‐CH), 7.12–7.09 (m, 1H), 7.20–7.16 (m, 1H), 7.43 (d, *J* = 8.2 Hz, 1H), 7.57 (s, 1H), 8.03 (s, 1H), 8.05 (s, 1H), 11.91 (br s, 1H, NH). ^13^C NMR (50 MHz, DMSO‐*d*_6_) *δ*: 32.9 (qx2), 34.3 (q), 110.2 (d), 119.7 (d), 121.1 (d), 121.8 (d), 125.7 (2xs), 127.0 (2xd), 137.25 (3xs), 155.4 (d); *Anal.* Calculated for C_15_H_17_N_5_ (MW: 267.33): C, 67.39; H, 6.41; N, 26.20%. Found: C, 67.55; H, 6.27; N, 26.06%.

##### N′‐[3‐(5‐bromo‐1‐methyl‐1H‐indol‐3‐yl)‐1H‐pyrazol‐5‐yl]‐N,N‐dimethylmethanimidamide (11b)

Condition: 2.5 h reflux. Yield: 88%; white solid; m.p.: 215 °C. IR (cm^−1^): 3177 (NH); ^1^H NMR (200 MHz, DMSO‐*d*_6_) *δ*: 2.92 (s, 3H, CH_3_), 3.02 (s, 3H, CH_3_), 3.79 (s, 3H, CH_3_), 6.08 (s, 1H, pyrazole‐CH), 7.30–7.26 (m, 1H), 7.42 (d, *J* = 8.7 Hz, 1H), 7.61 (s, 1H), 8.03 (s, 1H), 8.28 (s, 1H), 11.96 (br s, 1H, NH). ^13^C NMR (50 MHz, DMSO‐*d*_6_) *δ*: 33.1 (qx2), 34.3 (q), 110.2 (s), 112.3 (d), 112.5 (s), 123.8 (d), 124.0 (d), 124.2 (d), 127.3 (s), 128.5 (d), 136.0 (2xs), 153.1 (s), 155.7 (d); *Anal.* Calculated for C_15_H_16_BrN_5_ (MW: 346.23): C, 52.04; H, 4.66; N, 20.23%. Found: C, 52.22; H, 4.72; N, 20.09%.

##### N′‐[3‐(5‐chloro‐1‐methyl‐1H‐indol‐3‐yl)‐1H‐pyrazol‐5‐yl]‐N,N‐dimethylmethanimidamide (11c)

Condition: 3 h reflux. Yield: 82%; white solid; m.p.: 213.7–213.9 °C. IR (cm^−1^): 3179 (NH); ^1^H NMR (200 MHz, DMSO‐*d*_6_) *δ*: 2.92 (s, 3H, CH_3_), 3.03 (s, 3H, CH_3_), 3.80 (s, 3H, CH_3_), 6.10 (s, 1H, pyrazole‐CH), 7.18 (d, *J* = 8.3 Hz, 1H), 7.47 (d, *J* = 8.6 Hz, 1H), 7.64 (s, 1H), 8.05 (s, 1H), 8.11 (s, 1H), 11.98 (br s, 1H, NH). ^13^C NMR (50 MHz, DMSO‐*d*_6_) *δ*: 33.1 (qx2), 34.3 (q), 111.9 (2xd), 120.5 (s), 121.7 (d), 124.5 (s), 126.6 (s), 126.7 (s),128.7 (2xd), 135.8 (2xs), 155.6 (d); *Anal.* Calculated for C_15_H_16_ClN_5_ (MW: 301.77): C, 59.70; H, 5.34; N, 23.21%. Found: C, 59.56; H, 5.12; N, 23.07%.

##### N′‐[3‐(5‐fluoro‐1‐methyl‐1H‐indol‐3‐yl)‐1H‐pyrazol‐5‐yl]‐N,N‐dimethylmethanimidamide (11d)

Condition: 3 h reflux. Yield: 86%; white solid; m.p.: 222.3–222.5 °C. IR (cm^−1^): 3179 (NH); ^1^H NMR (200 MHz, DMSO‐*d*_6_) *δ*: 2.92 (s, 3H, CH_3_), 3.02 (s, 3H, CH_3_), 6.09 (s, 1H, pyrazole‐CH), 7.02 (t, *J* = 8.2 Hz, 1H), 7.44 (s, 1H), 7.64 (s, 1H), 7.80 (s, 1H), 8.05 (s, 1H), 11.94 (br s, 1H, NH). ^13^C NMR (50 MHz, DMSO‐*d*_6_) *δ*: 33.2 (qx2), 34.3 (q), 86.3 (d), 106.2 (d, *J*_C4‐F_ = 17.4 Hz), 109.9 (d, *J*_C6*‐*F_ = 24.0 Hz), 111.3 (d, *J*_C7‐F_ = 5.9 Hz), 125.9 (d, *J*_C3a‐F_ = 13.1 Hz), 128.9 (d), 134.0 (s), 146.6 (s), 153.1 (d, *J*_C7a‐F_ = 3.0 Hz), 153.2 (s), 155.7 (d), 157.8 (d, *J*_C5‐F_ = 230.01 Hz); *Anal.* Calculated for C_15_H_16_FN_5_ (MW: 285.32): C, 63.14; H, 5.65; N, 24.55%. Found: C, 63.00; H, 5.41; N, 24.41%.

##### N′‐[3‐(5‐methoxy‐1‐methyl‐1H‐indol‐3‐yl)‐1H‐pyrazol‐5‐yl]‐N,N‐dimethylmethanimidamide (11e)

Condition: 1 h reflux. Yield: 99%; white solid; m.p.: 210 °C. IR (cm^−1^): 3178 (NH); ^1^H NMR (200 MHz, DMSO‐*d*_6_) *δ*: 2.90 (s, 3H, CH_3_), 3.01 (s, 3H, CH_3_), 3.79 (s, 3H, OCH_3_), 6.07 (s, 1H, pyrazole‐CH), 6.82 (m, 1H), 7.33 (d, *J* = 8.8 Hz, 1H), 7.51 (s, 1H), 7.61 (s, 1H), 8.03 (s, 1H), 11.90 (br s, 1H, NH). ^13^C NMR (50 MHz, DMSO‐*d*_6_) *δ*: 33.1 (qx2), 34.3 (q), 55.8 (q), 66.8 (s), 111.0 (d), 111.9 (2xd), 125.9 (2xs), 127.7 (2xd), 132.6 (2xs), 154.2 (s), 155.5 (d); *Anal.* Calculated for C_16_H_19_N_5_O (MW: 297.35): C, 64.63; H, 6.44; N, 23.55;. Found: C, 64.59; H, 6.31; N, 23.41%.

##### N,N‐Dimethyl‐N′‐[5‐(1‐methyl‐1H‐pyrrolo[2,3‐b]pyridin‐3‐yl)‐2H‐pyrazol‐3‐yl]‐formamidine (12a)

Condition: 1.5 h reflux. Yield: 66%; beige solid; m.p.: 218.6–220 °C.; IR (cm^−1^): 3356 (NH); ^1^H NMR (200 MHz, DMSO‐*d*_6_) *δ*: 2.87 (s, 3H, CH_3_), 2.99 (s, 3H, CH_3_), 3.85 (s, 3H, CH_3_), 6.15 (s, 1H, pyrazole‐CH), 7.34 (dd, *J =* 7.7, 4.8 Hz, 1H), 8.43 (t, *J =* 6.0 Hz, 1H), 8.04 (s, 1H), 8.34 (d, *J* = 2.2 Hz, 1H), 12.06 (s, 1H, NH). ^13^C NMR (50 MHz, DMSO‐*d*_6_) *δ*: 33.5 (qx2), 34.6 (q), 103.1 (d), 114.13 (s), 121.1 (s), 128.6 (d), 129.7 (d), 131.5 (s), 134.7 (d), 136.3 (s), 143.2 (d), 150.6 (d), 155.6 (s); *Anal.* Calculated for C_14_H_15_BrN_6_ (MW: 347.21): C, 48.43; H, 4.35; N, 24.20%. Found: C, 48.29; H, 4.21; N, 24.06%.

##### N′‐[5‐(5‐Bromo‐1‐methyl‐1H‐pyrrolo[2,3‐b]pyridin‐3‐yl)‐2H‐pyrazol‐3‐yl]‐N,N‐dimethyl‐formamidine (12b)

Condition: 2 h reflux. Yield: 56%; white solid; m.p.: 210 °C. ^1^H NMR (200 MHz, DMSO‐*d*_6_) *δ*: 2.97 (d, *J* = 44.2 Hz, 6H, CH_3_), 3.83 (s, 3H, CH_3_), 6.15 (s, 1H, pyrazole‐CH), 7.82 (s, 1H), 8.04 (s, 1H), 8.34 (d, *J* = 2.2 Hz, 1H), 8.58 (d, *J* = 2.0 Hz, 1H), 12.06 (s, 1H, NH). ^13^C NMR (50 MHz, DMSO‐*d*_6_) *δ*: 33.6 (qx2), 34.4 (q), 103.1 (d), 114.13 (s), 121.1 (s), 128.6 (d), 129.7 (d), 131.5 (s), 134.7 (d), 136.3 (s), 143.2 (s), 150.6 (d), 155.6 (s); *Anal.* Calculated for C_14_H_15_BrN_6_ (MW: 347.21): C, 48.43; H, 4.35; N, 24.20%. Found: C, 48.29; H, 4.21; N, 24.36%.

##### General Procedure for the Synthesis of 7‐(1H‐indol‐3‐yl)‐pyrazolo[1,5‐a][1,3,5]triazin‐4‐ylamines (1a‐j) and 7‐(1‐methyl‐1H‐pyrrolo[2,3‐b]pyridin‐3‐yl)pyrazolo[1,5‐a][1,3,5]triazin‐4‐amines (2a,b)

To a solution of *N*’‐[3‐(*1H*‐indol‐3‐yl)‐*1H*‐pyrazol‐5‐yl]*‐N, N*‐dimethylmethanimidamides **10–12** (3.95 mmol) in anhydrous dimethyl sulfoxide, sodium hydrade 60% dispersion in mineral oil (9.88 mmol) and cyanamide (9.88 mmol), were added under nitrogen atmosphere. The reaction mixture was heated vigorously under reflux for 1–2.5 h (the progress of the reaction was monitored by TLC). After completion, the reaction mixture was left to cool, water/ice were added and the formed precipitate was filtered off and further purified by column chromatography, using dichloromethane/ methanol as eluent 97:3.

##### 7‐(1H‐indol‐3‐yl)pyrazolo[1,5‐a][1,3,5]triazin‐4‐amine (1a)

Condition: 2 h reflux. Yield: 45%; white solid; m.p.: 274.2–279.9 °C. IR (cm^−1^): 3422 (NH_2_), 3308 (NH); ^1^H NMR (200 MHz, DMSO‐*d*_6_) *δ*: 6.76 (s, 1H), 7.15–7.10 (m, 1H), 7.20–7.16 (m, 1H), 7.44 ( d, *J* = 7.7 Hz, 1H), 8.01 (s, 1H), 8.05 (d, *J* = 1.6 Hz, 1H), 8.62–8.10 (m, 3H, NH_2_), 11.51 (s, 1H, NH). ^13^C NMR (50 MHz, DMSO‐*d*_6_) *δ*: 91.8 (d), 108.8 (s), 112.0 (d), 111.1 (s), 120.4 (d), 122.3 (d), 122.6 (d), 125.3 (s), 127.0 (d), 137.1 (s), 149.5 (s), 150.9 (s), 153.7 (s), 154.2 (d); *Anal.* Calculated for C_13_H_10_N_6_ (MW: 250.26): C, 62.39; H, 4.03; N, 33.58%. Found: C, 62.25; H, 3.89; N, 33.44%.

##### 7‐(5‐Bromo‐1H‐indol‐3‐yl)pyrazolo[1,5‐a][1,3,5]triazin‐4‐amine (1b)

Condition: 1 h reflux. Yield: 80%; white solid; m.p.: 312.1–316.5 °C. IR (cm^−1^): 3355 (NH_2_), 3183 (NH); 1H NMR (200 MHz, DMSO‐*d*_6_) *δ*: 6.76 (s, 1H), 7.29 (dd, *J* = 8.6, 2.0 Hz, 1H), 7.41 (d, *J* = 8.6 Hz, 1H), 8.01 (s, 1H), 8.12 (d, *J* = 2.0 Hz, 1H), 8.48 (br s, 2H, NH_2_), 8.72 (d, *J* = 1.9 Hz, 1H), 11.71 (s, 1H, NH). ^13^C NMR (50 MHz, DMSO‐*d*_6_) *δ*: 91.9 (d), 108.7 (s), 113.5 (s), 114.0 (d), 124.5 (d), 125.1 (d), 126.8 (s), 128.5 (d), 135.8 (s), 149.6 (s), 151.0 (s), 153.1 (s), 154.3 (d); *Anal.* Calculated for C_13_H_9_BrN_6_ (MW: 329.15): C, 47.44; H, 2.76; N, 25.53%. Found: C, 47.30; H, 2.62; N, 25.40%.

##### 7‐(5‐Chloro‐1H‐indol‐3‐yl)pyrazolo[1,5‐a][1,3,5]triazin‐4‐amine (1c)

Condition: 1 h reflux. Yield: 60%; white solid; m.p.:318.2‐322.4. IR (cm^−1^): 3406 (NH_2_), 3298 (NH); ^1^H NMR (200 MHz, DMSO‐*d*_6_) *δ*: 6.76 (s, 1H), 7.17 (dd, *J* = 8.6, 2.5 Hz, 1H), 7.45 (d, *J* = 8.7 Hz, 1H), 8.01 (s, 1H), 8.14 (d, *J* = 2.5 Hz, 1H), 8.49 (br s, 2H, NH_2_), 8.62 (d, *J* = 1.9 Hz, 1H), 11.69 (s, 1H, NH). ^13^C NMR (50 MHz, DMSO‐*d*_6_) *δ*: 91.8 (d), 108.8 (s), 113.6 (d), 121.6 (d), 122.5 (d), 125.4 (s), 126.2 (s), 128.7 (d), 135.6 (s), 149.6 (s), 151.0 (s), 153.1 (s), 154.3 (d); *Anal.* Calculated for C_13_H_9_ClN_6_ (MW: 284.70): C, 54.84; H, 3.19; N, 29.52%. Found: C, 54.90; H, 3.05; N, 29.28%.

##### 7‐(5‐Fluoro‐1H‐indol‐3‐yl)pyrazolo[1,5‐a][1,3,5]triazin‐4‐amine (1d)

Condition: 2.5 h reflux. Yield: 70%; white solid; m.p.: 336.8–340.9. IR (cm^−1^): 3414 (NH_2_), 3228 (NH); ^1^H NMR (200 MHz, DMSO‐*d*_6_) *δ*: 6.75 (s, 1H), 7.01 (td, *J* = 9.2, 2.6 Hz, 1H), 7.40–7.45 (m, 1H), 8.00 (s, 1H), 8.13 (s, 1H), 8.62–8.25 (m, 3H, NH_2_), 11.60 (s, 1H, NH). ^13^C NMR (50 MHz, DMSO‐*d*_6_) *δ*: 91.6 (d), 107.5 (d, *J*_C4‐F_ = 24.3 Hz), 109.1 (s), 109.2 (s), 110.6 (d, *J*_C6‐F_ = 26.6 Hz), 113.0 (d, *J*_C7‐F_ = 10.00 Hz), 125.5 (s), 125.6 (s), 128.9 (d), 133.8 (s), 150.3 (d, *J*_C5‐F_ = 137.1 Hz), 150.9 (s), 153.3 (s), 154.3 (d); *Anal.* Calculated for C_13_H_9_FN_6_ (MW: 268.25): C, 58.21; H, 3.38; N, 31.33%. Found: C, 58.07; H, 3.24; N, 31.19%.

##### 7‐(5‐Methoxy‐1H‐indol‐3‐yl)pyrazolo[1,5‐a][1,3,5]triazin‐4‐amine (1e)

Condition: 2 h reflux. Yield:71%; white solid; m.p.: 270.7–272.8. IR (cm^−1^): 3368 (NH_2_), 3311 (NH); ^1^H NMR (200 MHz, DMSO‐*d*_6_) *δ*: 3.87 (s, 3H, OCH_3_), 6.74 (s, 1H), 6.82 (dd, *J* = 8.8, 2.5 Hz, 1H), 7.33 (d, *J* = 8.8 Hz, 1H), 7.93 (d, *J* = 2.4 Hz, 1H), 7.99 (d, *J* = 2.7 Hz, 1H), 8.01 (s, 1H), 8.33 (br s, 2H, NH_2_), 11.38 (s, 1H, NH). ^13^C NMR (50 MHz, DMSO‐*d*_6_) *δ*: 56.2 (q), 91.9 (d), 104.3 (d), 108.6 (s), 112.4 (d), 112.7 (d), 125.7 (s), 127.6 (d), 132.2 (s), 149.6 (s), 150.9 (s), 153.8 (s), 154.2 (d), 154.8 (s); *Anal.* Calculated for C_14_H_12_N_6_O (MW: 280.28): C, 59.99; H, 4.32; N, 29.98%. Found: C, 59.85; H, 4.18; N, 29.74%.

##### 7‐(1‐Methyl‐1H‐indol‐3‐yl)pyrazolo[1,5‐a][1,3,5]triazin‐4‐amine (1f)

Condition: 1.5 h reflux. Yield: 93%; white solid; m.p.: 276.1–278.5 °C. IR (cm^−1^): 3552 (NH_2_); ^1^H NMR (200 MHz, DMSO‐*d*_6_) *δ*: 3.86 (s, 3H, CH_3_), 6.70 (s, 1H), 7.17 (t, *J* = 7.4 Hz, 1H), 7.25 (t, *J* = 7.4 Hz, 1H), 7.49 (d, *J* = 8.1 Hz, 1H), 8.01 (s, 1H), 8.04 (s, 1H), 8.61–8.04 (m, 3H, NH_2_). ^13^C NMR (50 MHz, DMSO‐*d*_6_) *δ*: 33.2 (q), 91.6 (d), 107.9 (s), 110.4 (d), 120.6 (d), 122.4 (d), 122.8 (d), 125.7 (s), 130.9 (d), 137.6 (s), 149.6 (s), 150.9 (s), 153.3 (s), 154.3 (d); *Anal.* Calculated for C_14_H_12_N_6_ (MW: 264.29): C, 63.62; H, 4.58; N, 31.80%. Found: C, 63.48; H, 4.74; N, 31.66%.

##### 7‐(5‐Bromo‐1‐methyl‐1H‐indol‐3‐yl)pyrazolo[1,5‐a][1,3,5]triazin‐4‐amine (1g)

Condition: 1 h reflux. Yield: 70%; white‐beige solid; m.p.: 320.3–323.2. IR (cm^−1^): 3447 (NH_2_); ^1^H NMR (200 MHz, DMSO‐*d*_6_) *δ*: 3.85 (s, 3H, CH_3_), 6.69 (s, 1H), 7.35 (dd, *J* = 8.7, 1.9 Hz, 1H), 7.48 (d, *J* = 8.7 Hz, 1H), 8.01 (s, 1H), 8.10 (s, 1H), 8.49 (br s, 2H, NH_2_), 8.74 (d, *J* = 1.8 Hz, 1H). ^13^C NMR (50 MHz, DMSO‐*d*_6_) *δ*: 33.5 (q), 91.7 (d), 107.7 (s), 112.5 (d), 113.9 (s), 124.6 (d), 125.1 (d), 127.1 (s), 132.4 (d), 136.4 (s), 149.7 (s), 151.0 (s), 152.7 (s), 154.4 (d); *Anal.* Calculated for C_14_H_11_BrN_6_ (MW: 343.18): C, 49.00; H, 3.23; N, 24.49%. Found: C, 48.96; H, 3.19; N, 24.45%.

##### 7‐(5‐Chloro‐1‐methyl‐1H‐indol‐3‐yl)pyrazolo[1,5‐a][1,3,5]triazin‐4‐amine (1h)

Condition: 2 h reflux. Yield: 50%; white solid; m.p.:306.7–308.8. IR (cm^−1^): 3502 (NH_2_); ^1^H NMR (200 MHz, DMSO‐*d*_6_) *δ*: 3.86 (s, 3H, CH_3_), 6.69 (s, 1H), 7.24 (dd, *J* = 8.7, 2.1 Hz, 1H), 7.53 (d, *J* = 8.7 Hz, 1H), 8.01 (s, 1H), 8.12 (s, 1H), 8.49 (br s 2H, NH_2_), 8.63 (d, *J* = 2.0 Hz, 1H). ^13^C NMR (50 MHz, DMSO‐*d*_6_) *δ*: 33.5 (q), 91.6 (d), 107.8 (s), 112.1 (d), 121.8 (d), 122.5 (d), 125.8 (s), 126.4 (s), 132.5 (d), 136.1 (s), 149.7 (s), 151.0 (s), 152.7 (s), 154.4 (d); *Anal.* Calculated for C_14_H_11_ClN_6_ (MW: 298.73): C, 56.29; H, 3.71;; N, 28.13%. Found: C, 56.15; H, 3.57; N, 28.02%.

##### 7‐(5‐Fluoro‐1‐methyl‐1H‐indol‐3‐yl)pyrazolo[1,5‐a][1,3,5]triazin‐4‐amine (1i)

Condition: 2 h reflux. Yield: 80%; white solid; m.p.: 299.7–300.9. IR (cm^−1^): 3447 (NH_2_); ^1^H NMR (200 MHz, DMSO‐*d*_6_) *δ*: 3.86 (s, 3H, CH_3_), 6.68 (s, 1H), 7.08 (td, *J* = 9.2, 2.6 Hz, 1H), 7.48–7.52 (m, 1H), 8.01 (s, 1H), 8.11 (s, 1H), 8.58–8.30 (m, 3H, NH_2_). ^13^C NMR (50 MHz, DMSO‐*d*_6_) *δ*: 33.5 (q), 91.4 (d), 107.8 (d, *J*_C4‐F_ = 24.1 Hz), 108.1 (d, *J*_C7a‐F_ = 4.3 Hz), 110.6 (d, *J*_C6‐F_ = 26.4 Hz), 111.6 (d, *J*_C7‐F_ = 5.9 Hz), 125.8 (d, *J*_C4‐F_ = 10.8 Hz), 132.6 (d), 134.4 (s), 149.6 (s), 150.9 (s), 152.9 (s), 154.3 (d), 158.5 (d, *J*_C5‐F_ = 232.5 Hz); *Anal.* Calculated for C_14_H_11_FN_6_ (MW: 282.28): C, 59.57; H, 3.93; N, 29.77%. Found: C, 59.43; H, 3.82; F, 6.69; N, 29.63%.

##### 7‐(5‐Methoxy‐1‐methyl‐1H‐indol‐3‐yl)pyrazolo[1,5‐a][1,3,5]triazin‐4‐amine (1j)

Condition: 2.5 h reflux. Yield: 40%; yellow‐beige solid; m.p.: 298.7–303.1 °C. IR (cm^−1^): 3447 (NH_2_); ^1^H NMR (200 MHz, DMSO‐*d*_6_) *δ*: 3.82 (s, 3H, CH_3_), 3.87 (s, 3H, OCH_3_), 6.68 (s, 1H), 6.88 (dd, *J* = 8.9, 2.5 Hz, 1H), 7.39 (d, *J* = 8.9 Hz, 1H), 7.94 (d, *J* = 2.4 Hz, 1H), 7.97 (s, 1H), 8.01 (s, 1H), 8.56–8.12 (br s, 2H, NH_2_). ^13^C NMR (50 MHz, DMSO‐*d*_6_) *δ*: 33.4 (q), 56.2 (q), 91.7 (d), 104.6 (d), 107.5 (s), 111.2 (d), 112.4 (d), 126.1 (s), 131.4 (d), 132.9 (s), 149.6 (s), 150.9 (s), 153.4 (s), 154.2 (d), 155.0 (s); *Anal.* Calculated for C_15_H_14_N_6_O (MW: 294.31): C, 61.21; H, 4.79; N, 28.55%. Found: C, 61.07; H, 4.65; N, 28.71%.

##### 7‐(1‐Methyl‐1H‐pyrrolo[2,3‐b]pyridin‐3‐yl)pyrazolo[1,5‐a][1,3,5]triazin‐4‐amine (2a)

Condition: 1.5 h reflux. Yield: 65%; white solid; m.p.: 279.1–280.8. IR (cm^−1^): 3419 (NH_2_); ^1^H NMR (200 MHz, DMSO‐*d*_6_) *δ*: 3.89 (s, 3H), 6.74 (s, 1H), 7.22 (dd, *J* = 7.9, 4.7 Hz, 1H), 8.02 (s, 1H), 8.23 (s, 1H), 8.66–8.25 (m, 3H, NH_2_), 8.98 (dd, *J* = 7.9, 1.6 Hz, 1H). ^13^C NMR (50 MHz, DMSO‐*d*_6_) *δ*: 31.5 (q), 91.5 (d), 106.6 (s), 117.0 (d), 118.0 (s), 130.7 (d), 131.2 (d), 143.8 (d), 148.3 (s), 149.7 (s), 150.9 (s), 152.6 (s), 154.4 (d); *Anal.* Calculated for C_13_H_11_N_7_ (MW: 265.27): C, 58.86; H, 4.18; N, 36.96%. Found: C, 58.62; H, 4.34; N, 36.82%.

##### 7‐(5‐bromo‐1‐methyl‐1H‐pyrrolo[2,3‐b]pyridin‐3‐yl)pyrazolo[1,5‐a][1,3,5]triazin‐4‐amine (2b)

Condition: 1 h reflux. Yield: 55%; white solid; m.p.: 354.5–357.8. IR (cm^−1^): 3420 (NH_2_); ^1^H NMR (200 MHz, DMSO‐*d*_6_) *δ*: 3.87 (s, 3H, CH_3_), 6.74 (s, 1H), 8.02 (s, 1H), 8.32 (s, 1H), 8.41 (d, *J* = 2.2 Hz, 1H), 8.62–8.55 (br s, 2H, NH_2_), 9.18 (d, *J* = 2.2 Hz, 1H). ^13^C NMR (50 MHz, DMSO‐*d*_6_) *δ*: 31.8 (q), 91.4 (d), 106.4 (s), 112.9 (s), 119.4 (s), 125.4 (s), 132.5 (d), 132.8 (d), 144.0 (d), 151.0 (s), 154.1 (s), 154.5 (d), 156.0 (s). *Anal.* Calculated for C_13_H_10_BrN_7_ (MW: 344.17): C, 45.37; H, 2.93; N, 28.49%. Found: C, 45.33; H, 2.89; Br, 23.18; N, 28.45%.

##### General Procedure for the Synthesis of 7‐(1H‐Indol‐3‐yl)‐2‐phenyl‐pyrazolo[1,5‐a][1,3,5]triazin‐4‐ylamines (3a‐j) and 7‐(1‐methyl‐1H‐pyrrolo[2,3‐b]pyridin‐3‐yl)‐2‐phenylpyrazolo[1,5‐a][1,3,5]triazin‐4‐amines (4a,b)

To a solution of the proper 3‐(1*H*‐indol‐3‐yl)‐1*H*‐pyrazol‐5‐amine **7–9** (5.04 mmol) in anhydrous dimethyl sulfoxide, triethyl orthobenzoate (2.85 mL) and cyanamide (12.60 mmol), were added under nitrogen atmosphere. The reaction mixture was heated at 165 °C for 2 h. Then sodium hydride 60% dispersion in mineral oil (12.60 mmol) was added to the reaction mixture that was heated at 180 °C for 2.5 h. After completion (monitoring via TLC), the reaction mixture was left to cool, then added ice/cold water to obtain a precipitate that was filtered off. The resulting crude material was purified in column using dichloromethane/EtOAc 85:15.

##### 7‐(1H‐indol‐3‐yl)‐2‐phenylpyrazolo[1,5‐a][1,3,5]triazin‐4‐amine (3a)

Yield: 97%; white solid; m.p.: 290.8–291.5 °C. IR (cm^−1^): 3447 (NH_2_), 3275 (NH); ^1^H NMR (200 MHz, DMSO‐*d*_6_) *δ*: 6.81 (s, 1H), 7.13 (dd, *J* = 7.0, 1.2 Hz, 1H), 7.17 (dd, *J* = 2.7, 1.6 Hz, 1H), 7.20 (dd, *J* = 7.0, 1.3 Hz, 1H), 7.47–7.43 (m, 1H), 7.53–7.49 (m, 3H), 8.07 (d, *J* = 2.7 Hz, 1H), 8.39–8.21 (m, 3H, NH_2_), 8.61 (d, *J* = 7.2 Hz, 2H), 11.52 (br s, 1H, NH). ^13^C NMR (50 MHz, DMSO‐*d*_6_) *δ*: 92.0 (d), 109.0 (s), 112.1 (d), 120.4 (d), 122.3 (d), 122.7 (d), 125.3 (s), 126.9 (d), 128.3 (2xd), 128.8 (2xd), 131.1(d), 137.1 (s), 137.5 (s), 150.2 (s), 150.7 (s), 154.2 (s), 158.8 (s); *Anal.* Calculated for C_19_H_14_N_6_ (MW: 326.35): C, 69.92; H, 4.32; N, 25.75%. Found: C, 69.86; H, 4.42; N, 25.61%.

##### 7‐(5‐Bromo‐1H‐indol‐3‐yl)‐2‐phenylpyrazolo[1,5‐a][1,3,5]triazin‐4‐amine (3b)

Yield: 53%; white solid; m.p.:327.3‐329.2 °C. IR (cm^−1^): 3445 (NH_2_), 3213 (NH); ^1^H NMR (200 MHz, DMSO‐*d*_6_) *δ*: 6.82 (s, 1H), 7.30 (dd, *J* = 8.6, 2.0 Hz, 1H), 7.42 (d, *J* = 8.6 Hz, 1H), 7.54–7.47 (m, 3H), 8.15 (d, *J* = 2.7 Hz, 1H), 8.41–8.36 (m, 2H), 8.54 (br s, 2H, NH_2_), 8.76 (d, *J* = 1.9 Hz, 1H), 11.74 (br s, 1H, NH). ^13^C NMR (50 MHz, DMSO‐*d*_6_) *δ*: 92.1 (d), 108.8 (s), 113.5 (s), 114.1 (d), 124.5 (d), 125.1 (d), 126.8 (s), 128.3 (2xd), 128.5 (d), 128.8 (2xd) 131.1 (d), 135.8 (s), 137.4 (s), 150.3 (s), 150.8 (s), 153.6 (s), 159.0 (s). *Anal.* Calculated for C_19_H_13_BrN_6_ (MW: 405.25): C, 56.31; H, 3.23; N, 20.74%; Found: C, 56.44; H, 3.39; N, 20.79%.

##### 7‐(5‐Chloro‐1H‐indol‐3‐yl)‐2‐phenylpyrazolo[1,5‐a][1,3,5]triazin‐4‐amine (3c)

Yield: 64%; white solid; m.p.: 308.2–309.2 °C. IR (cm^−1^): 3449 (NH_2_), 3213 (NH); ^1^H NMR (200 MHz, DMSO‐*d*_6_) *δ*: 6.82 (s, 1H), 7.18 (dd, *J* = 8.6, 2.1 Hz, 1H), 7.46 (d, *J* = 8.6 Hz, 1H), 7.54–7.49 (m, 3H), 8.16 (d, *J* = 2.7 Hz, 1H), 8.41–8.36 (m, 2H), 8.53 (br s, 2H, NH_2_), 8.66 (d, *J* = 2.1 Hz, 1H), 11.72 (br s, 1H, NH). ^13^C NMR (50 MHz, DMSO‐*d*_6_) *δ*: 92.0 (d), 108.9 (s), 113.6 (d), 121.7 (d), 122.5 (d), 125.4 (s), 126.2 (s), 128.3 (2xd), 128.7 (d), 128.8 (2xd), 131.1 (d), 135.6 (s), 137.4 (s), 150.3 (s), 150.8 (s), 153.6 (s), 159.0 (s). *Anal.* Calculated for C_19_H_13_ClN_6_ (MW: 360.80): C, 63.25; H, 3.63; N, 23.29%. Found: C, 63.31; H, 3.45; N, 23.55%.

##### 7‐(5‐Fluoro‐1H‐indol‐3‐yl)‐2‐phenylpyrazolo[1,5‐a][1,3,5]triazin‐4‐amine (3d)

Yield: 71%; white solid; m.p.: 306.8–309.2 °C. IR (cm^−1^): 3447 (NH_2_), 3198 (NH); ^1^H NMR (200 MHz, DMSO‐*d*_6_) *δ*: 6.81 (s, 1H), 7.02 (td, *J* = 9.2, 2.6 Hz, 1H), 7.44 (dd, *J* = 8.8, 4.6 Hz, 1H), 7.53–7.48 (m, 3H), 8.16 (d, *J* = 2.7 Hz, 1H), 8.65–8.32 (m, 5H, NH_2_), 11.63 (br s, 1H, NH). ^13^C NMR (50 MHz, DMSO‐*d*_6_) *δ*: 91.8 (d), 107.5 (d, *J*_*C4‐F*_ = 23.7 Hz), 109.3 (d, *J*_C7a‐F_ = 4.2 Hz), 110.6 (d, *J*_C6‐F_ = 25.8 Hz), 113.0 (d, *J*_C7‐F_ = 9.0 Hz), 125.6 (s), 128.3 (2xd), 128.8 (2xd), 128.8 (d), 131.1 (d), 133.8 (s), 137.5 (s), 150.2 (s), 150.7 (s), 153.8 (s), 158.9 (s), 159.4 (s). *Anal.* Calculated for C_19_H_13_FN_6_ (MW: 344.35): C, 66.27; H, 3.81; N, 24.41%. Found: C, 66.21; H, 3.67; N, 24.23%.

##### 7‐(5‐Methoxy‐1H‐indol‐3‐yl)‐2‐phenylpyrazolo[1,5‐a][1,3,5]triazin‐4‐amine (3e)

Yield: 64%; white solid; m.p.: 308.2–309.4 °C. IR (cm^−1^): 3447 (NH_2_), 3253 (NH); ^1^H NMR (200 MHz, DMSO‐*d*_6_) *δ*: 3.89 (s, 3H, OCH_3_), 6.81 (s, 1H), 6.83 (dd, *J* = 8.8, 2.5 Hz, 1H), 7.35 (d, *J* = 8.8 Hz, 1H), 7.55–7.47 (m, 3H), 7.96 (d, *J* = 2.4 Hz, 1H), 8.02 (d, *J* = 2.7 Hz, 1H), 8.60–8.17 (m, 4H, NH_2_), 11.40 (br s, 1H, NH). ^13^C NMR (50 MHz, DMSO‐*d*_6_) *δ*: 56.19 (q), 92.1 (d), 104.3 (d), 108.7 (s), 112.5 (d), 112.7 (d), 125.7 (s), 127.5 (d), 128.3 (2xd), 128.8 (2xd), 131.1 (d), 132.2 (s), 137.5 (s), 150.2 (s), 150.7 (s), 154.3 (s), 154.8(s), 158.8 (s). *Anal.* Calculated for C_20_H_16_N_6_O (MW: 356.38): C, 67.40; H, 4.53; N, 23.58%. Found: C, 67.33; H, 4.41; N, 23.78%.

##### 7‐(1‐Methyl‐1H‐indol‐3‐yl)‐2‐phenylpyrazolo[1,5‐a][1,3,5]triazin‐4‐amine (3f)

Yield: 77%; white solid; m.p.:287.2‐290.1 °C. IR (cm^−1^): 3439 (NH_2_); ^1^H NMR (200 MHz, DMSO‐*d*_6_) *δ*: 3.87 (s, 3H, OCH_3_), 6.75 (s, 1H), 7.21–7.17 (m, 1H), 7.27–7.23 (m, 1H), 7.52–7.49 (m, 4H), 8.05 (s, 1H), 8.40–8.23 (m, 3H. NH_2_), 8.63 (d, *J* = 7.9 Hz, 2H). ^13^C NMR (50 MHz, DMSO‐*d*_6_) *δ*: 33.25 (q), 91.9 (d), 108.1 (s), 110.4 (d), 120.6 (s), 122.4 (d), 122.9 (d), 125.7 (d), 128.3 (2xd), 128.8 (2xd), 130.8 (d), 131.1 (d), 137.4 (s), 137.6 (s) 150.2 (s), 150.7 (s), 153.7 (s), 158.9 (s). *Anal.* Calculated for C_20_H_16_N_6_ (MW: 340.38): C, 70.57; H, 4.74; N, 24.69%. Found: C, 70.33; H, 4.60; N, 24.54%.

##### 7‐(5‐Bromo‐1‐methyl‐1H‐indol‐3‐yl)‐2‐phenylpyrazolo[1,5‐a][1,3,5]triazin‐4‐amine (3g)

Yield: 78%; Light Beige solid; m.p.: 291.1–294.5 °C. IR (cm^−1^): 3447 (NH_2_); ^1^H NMR (200 MHz, DMSO‐*d*_6_) *δ*: 3.86 (s, 3H, OCH_3_), 6.75 (s, 1H), 7.36 (dd, *J* = 8.7, 1.9 Hz, 1H), 7.53–7.47 (m, 4H), 8.12 (s, 1H), 8.40–8.37 (m, 2H), 8.57 (br s, 2H, NH_2_), 8.78 (d, *J* = 1.8 Hz, 1H). ^13^C NMR (50 MHz, DMSO‐*d*_6_) *δ*: 33.5 (q), 91.9 (d), 107.8 (s), 112.6 (d), 113.9 (s), 124.7 (d), 125.2 (d), 127.1 (s), 128.3 (2xd), 128.8 (2xd), 131.1 (d), 132.3 (d), 136.4 (s), 137.4 (s), 150.3 (s), 150.8 (s), 153.2 (s), 159.0 (s). *Anal.* Calculated for C_20_H_15_BrN_6_ (MW: 419.28): C, 57.29; H, 3.61; N, 20.04%. Found: C, 57.15; H, 3.66; N, 20.12%.

##### 7‐(5‐Chloro‐1‐methyl‐1H‐indol‐3‐yl)‐2‐phenylpyrazolo[1,5‐a][1,3,5]triazin‐4‐amine (3h)

Yield: 75%; white solid; m.p.: 286.3–287 °C. IR (cm^−1^): 3449 (NH_2_); ^1^H NMR (200 MHz, DMSO‐*d*_6_) *δ*: 3.87 (s, 3H, CH_3_), 6.75 (s, 1H), 7.25 (dd, *J* = 8.7, 2.1 Hz, 1H), 7.55–7.49 (m, 4H), 8.14 (s, 1H), 8.39–8.37 (m, 2H), 8.55 (br s, 2H, NH_2_), 8.67 (d, *J* = 2.0 Hz, 1H). ^13^C NMR (50 MHz, DMSO‐*d*_6_) *δ*: 33.5 (q), 91.8 (d), 107.9 (s), 112.1 (d), 121.9 (d), 122.5 (d), 125.8 (s), 126.4 (s), 128.3 (2xd), 128.8 (2xd), 131.1 (d), 132.5 (d), 136.1 (s), 137.4 (s), 150.3 (s), 150.8 (s), 153.2 (s), 159.0 (s). *Anal.* Calculated for C_20_H_15_ClN_6_ (MW: 374.83): C, 64.09; H, 4.03; N, 22.42%. Found: C, 64.25; H, 3.88; N, 22.33%.

##### 7‐(5‐Fluoro‐1‐methyl‐1H‐indol‐3‐yl)‐2‐phenyl‐pyrazolo[1,5‐a][1,3,5]triazin‐4‐ylamine (3i)

Yield: 60%; white solid; m.p.: 310.9–312.7 °C. IR (cm^−1^): 3445 (NH_2_); ^1^H NMR (200 MHz, DMSO‐*d*_6_) *δ*: 3.87 (s, 3H, CH_3_), 6.74 (s, 1H), 7.09 (td, *J* = 9.2, 2.6 Hz, 1H), 7.53–7.49 (m, 4H), 8.13 (s, 1H), 8.39–8.37 (m, 4H), 8.61–8.44 (m, 3H, NH_2_). ^13^C NMR (50 MHz, DMSO‐*d*_6_) *δ*: 33.6 (q), 91.6 (d), 107.8 (d, *J*_C4‐F_ = 24.3 Hz), 108.2 (d, *J*_C7a‐F_ = 4.4 Hz), 110.6 (d, *J*_C6‐F_ = 26.6 Hz), 111.6 (d, *J*_C7‐F_ = 10.1 Hz), 125.8 (d, *J*_C3a‐F_ =11.3 Hz), 128.3 (2xd), 128.8 (2xd), 131.1 (d), 132.6 (d), 134.4 (s), 137.4 (s), 150.3 (s), 150.7 (s), 157.4 (s), 159.0 (s), 158.2 (d, *J*_C5‐F_ =162.3 Hz)*. Anal.* Calculated for C_20_H_15_FN_6_ (MW: 358.37): C, 67.03; H, 4.22; N, 23.45%. Found: C, 66.88; H, 4.11; N, 23.33%.

##### 7‐(5‐Methoxy‐1‐methyl‐1H‐indol‐3‐yl)‐2‐phenylpyrazolo[1,5‐a][1,3,5]triazin‐4‐amine (3j)

Yield: 90%; white cream solid; m.p.: 271.2–275.5 °C. IR (cm^−1^): 3412 (NH_2_); ^1^H NMR (200 MHz, DMSO‐*d*_6_) *δ*: 3.84 (s, 3H, CH_3_), 3.89 (s, 3H, OCH_3_), 6.74 (s, 1H), 6.89 (dd, *J* = 8.8, 2.5 Hz, 1H), 7.40 (d, *J* = 8.9 Hz, 1H), 7.54–7.47 (m, 3H), 7.97 (d, *J* = 2.4 Hz, 1H), 7.99 (s, 1H), 8.40–8.19 (m, 4H, NH_2_). ^13^C NMR (50 MHz, DMSO‐*d*_6_) *δ*: 33.4 (q), 56.3 (q), 91.9 (d), 104.5 (d), 107.6 (s), 111.2 (d), 112.4 (d), 126.1 (s), 128.3 (2xd), 128.8 (2xd), 131.1 (d), 131.3 (d), 132.9 (s), 137.4 (s), 150.3 (s), 150.7 (s), 153.9 (s), 155.0 (s), 158.9 (s). *Anal.* Calculated for C_21_H_18_N_6_O (MW: 370.41): C, 68.09; H, 4.90; N, 22.69%. Found: C, 68.21; H, 4.78; N, 22.65%.

##### 7‐(1‐Methyl‐1H‐pyrrolo[2,3‐b]pyridin‐3‐yl)‐2‐phenylpyrazolo[1,5‐a][1,3,5]triazin‐4‐amine (4a)

Yield: 87%; white solid; m.p.: 309.3–311.5 °C. IR (cm^−1^): 3460 (NH_2_); ^1^H NMR (200 MHz, DMSO‐*d*_6_) *δ*: 3.91 (s, 3H, CH_3_), 6.80 (s, 1H), 7.23 (dd, *J* = 7.9, 4.6 Hz, 1H), 7.53–7.49 (m, 3H), 8.25 (s, 1H), 8.67–8.32 (m, 4H, NH_2_), 9.01 (dd, *J* = 7.9, 1.6 Hz, 1H). ^13^C NMR (50 MHz, DMSO‐*d*_6_) *δ*: 31.6 (q), 91.7 (d), 106.8 (s), 117.0 (d), 118.0 (s), 128.4 (2xd), 128.8 (2xd), 130.7 (d), 131.1 (d) 131.3 (d), 137.4 (s), 143.8 (d), 148.3 (s), 150.4 (s), 150.7 (s), 153.1 (s), 159.0 (s). *Anal.* Calculated for C_19_H_15_N_7_ (MW: 341.37): C, 66.85; H, 4.43; N, 28.72%. Found: C, 66.71; H, 4.32; N, 28.58%.

##### 7‐(5‐Bromo‐1‐methyl‐1H‐pyrrolo[2,3‐b]pyridin‐3‐yl)‐2‐phenyl‐pyrazolo[1,5‐a][1,3,5]triazin‐4‐ylamine (4b)

Yield: 89%; white solid; m.p.: 228.6‐ 236.2 °C. IR (cm^−1^): 3449 (NH_2_); ^1^H NMR (200 MHz, DMSO‐*d*_6_) *δ*: 3.49 (s, 3H, CH_3_), 6.79 (s, 1H), 7.53–7.48 (m, 3H), 8.33 (s, 1H), 8.40–8.35 (m, 2H), 8.42 (d, *J* = 2.2 Hz, 1H), 8.63 (br s, 2H, NH_2_), 9.21 (d, *J* = 2.2 Hz, 1H). ^13^C NMR (50 MHz, DMSO‐*d*_6_) *δ*: 31.8 (q), 91.6 (d), 106.5 (s), 112.9 (s), 119.4 (s), 128.4 (2xd), 128.8 (2xd), 131.1 (d), 132.4 (d), 132.9 (d), 137.4 (s), 144.0 (d), 146.7 (s), 150.4 (s), 150.8 (s), 152.5 (s), 159.1 (s). *Anal.* Calculated for C_19_H_14_BrN_7_ (MW: 420.27): C, 54.30; H, 3.36; N, 23.33%. Found: C, 54.16; H, 3.52; N, 23.19.

##### General Procedure for the N‐[7‐(1H‐indol‐3‐yl)pyrazolo[1,5‐a][1,3,5]triazin‐4‐yl]‐4‐nitrobenzamides (5a,b) and N‐[7‐(1H‐Indol‐3‐yl)‐2‐phenyl‐pyrazolo[1,5‐a][1,3,5]triazin‐4‐yl]‐4‐nitro‐benzamides (6a‐g)

To a solution of the proper 7‐(1*H*‐indol‐3‐yl)pyrazolo[1,5‐triazin‐4‐amines **1** (2 mmol) in anhydrous THF, sodium hydride 60% dispersion in mineral oil (6mmol) was added slowly at 0 °C. The reaction was stirred at 0 °C for 1 h. Subsequently, dropwise addition of a solution of 4‐nitrobenzoyl chloride (3 mmol) in anhydrous THF was carried out. The reaction was stirred at 0 °C for 30 min. Once complete, the reaction was dried under vacuum. The resulting crude material was purified in column chromatography using dichloromethane/methanol 96:4 as eluent.

##### N‐[7‐(1‐methyl‐1H‐indol‐3‐yl)‐pyrazolo[1,5‐a][1,3,5]triazin‐4‐yl]‐4‐nitro‐benzamide (5a)

Yield: 58%; red solid; m.p.: 306.3–307 °C. IR (cm^−1^): IR (cm^−1^): 3330 (NH), 1717 (CO), 1558 (NO); ^1^H NMR (200 MHz, DMSO‐*d*_6_) *δ*: 3.86 (s, 3H, CH_3_), 6.99 (s, 1H), 7.18 (t, *J* = 7.4 Hz, 1H), 7.25 (t, *J* = 7.4 Hz, 1H), 7.50 (d, *J* = 8.1 Hz, 1H), 8.08 (s, 1H), 8.15 (s), 8.18 (d, *J* = 7.6 Hz, 1H), 8.40 (d, *J* = 8.8 Hz, 2H), 8.44 (d, *J* = 8.6 Hz, 2H), 12.99 (s, 1H, NH). ^13^C NMR (50 MHz, DMSO‐*d*_6_) *δ*: 33.3 (q), 107.3 (s), 110.6 (d), 120.9 (d), 122.0 (d), 122.6 (d), 124.2 (2xd), 125.5 (d), 131.0 (2xd), 131.5 (d), 132.0 (d), 137.6 (s), 138.6 (s), 141.6 (s), 144.2 (s), 145.6 (s), 148.5 (s), 150.3 (s), 154.4 (s). *Anal.* Calculated for C_21_H_15_N_7_O_3_ (MW: 413.39): C, 61.01; H, 3.66; N, 23.72%. Found: C, 60.77; H, 3.69; N, 23.86%.

##### N‐[7‐(5‐fluoro‐1‐methyl‐1H‐indol‐3‐yl)‐pyrazolo[1,5‐a][1,3,5]triazin‐4‐yl]‐4‐nitro‐benzamide (5b)

Yield: 55%; yellow solid; m.p.: 278.4–281 °C. IR (cm^−1^): 3338 (NH), 1717 (CO), 1508 (NO); ^1^H NMR (200 MHz, DMSO‐*d*_6_) *δ*: 3.85 (s, 3H, CH_3_), 6.97 (s, 1H), 7.08 (td, *J* = 9.2, 2.6 Hz, 1H), 7.49–7.52 (m, 1H), 7.84 (d, *J* = 9.4 Hz, 1H), 8.15 (s, 1H), 8.34 (d, *J* = 2.0 Hz, 1H), 8.36 (d, *J* = 2.1 Hz, 1H), 8.39 (s, 1H), 8.42 (s, 1H), 12.94 (s, 1H, NH). ^13^C NMR (50 MHz, DMSO‐*d*_6_) *δ*: 33.6 (q), 106.6 (d, *J*_C4‐F_ = 24.2 Hz), 107.3 (s), 107.4 (s), 110.7 (d, *J*_C6‐F_ = 26.3 Hz), 111.9 (*J*_C7‐F_ = 9.4 Hz), 124.1 (2xd), 130.9 (2xd), 133.1 (2xd), 134.4 (s), 150.2 (2xs), 154.0 (2xs), 157.2 (s), 159.5 (s) *Anal.* Calculated for C_21_H_14_FN_7_O_3_ (MW: 431.38): C, 58.47; H, 3.27;; N, 22.73%. Found: C, 58.36; H, 3.22; N, 22.66%.

##### N‐[7‐(1H‐indol‐3‐yl)‐2‐phenyl‐pyrazolo[1,5‐a][1,3,5]triazin‐4‐yl]‐4‐nitro‐benzamide (6a)

Yield: 54%; orange solid; m.p.: 279.1–282.4 °C. IR (cm^−1^): 3365 (NH), 3260 (NH), 1717 (CO), 1541 (NO); ^1^H NMR (200 MHz, DMSO‐*d*_6_) *δ*: 7.15–7.11 (m, 2H), 7.22–7.16 (m, 1H), 7.47 (dd, *J* = 7.6, 5.3 Hz, 3H), 7.54 (t, *J* = 7.2 Hz, 1H), 8.13 (d, *J* = 1.5 Hz, 1H), 8.15 (s, 1H), 8.27 (s, 1H), 8.29 (s, 1H), 8.44–8.40 (m, 2H), 8.48 (d, *J* = 6.0 Hz, 1H), 11.63 (br s, 1H, NH), 11.97 (s, 1H, NH). ^13^C NMR (50 MHz, DMSO‐*d*_6_) *δ*: 108.4 (s), 117.8 (d), 112.2 (d), 120.7 (d), 122.3 (d), 122.5 (d), 124.2 (2xd), 125.2 (s), 128.0 (d), 128.3 (2xd), 129.0 (2xd), 130.5 (2xd), 131.9 (d), 137.2 (s), 146.1 (s), 150.1 (s), 150.8 (s), 151.7 (s), 153.1 (s), 154.0 (s), 154.6 (s), 155.5 (s). *Anal.* Calculated for C_26_H_17_N_7_O_3_ (MW: 475.46): C, 65.68; H, 3.60; N, 20.62%. Found: C, 65.44; H, 3.66; N, 20.48%.

##### N‐[7‐(5‐methoxy‐1H‐indol‐3‐yl)‐2‐phenyl‐pyrazolo[1,5‐a][1,3,5]triazin‐4‐yl]‐4‐nitro‐benzamide (6b)

Yield: 65%; yellow solid; m.p.: 259.8–263.6 °C. IR (cm^−1^): 3397 (NH), 3227 (NH), 1722 (CO), 1521 (NO); ^1^H NMR (200 MHz, DMSO‐*d*_6_) *δ*: 3.56 (s, 3H, OCH_3_), 6.79 (dd, *J* = 8.8, 2.5 Hz, 1H), 7.12 (s, 1H), 7.34 (d, *J* = 8.8 Hz, 1H), 7.60–7.48 (m, 3H), 7.85 (d, *J* = 2.4 Hz, 1H), 8.13 (d, *J* = 2.7 Hz, 1H), 8.28 (d, *J* = 1.3 Hz, 1H), 8.31 (d, *J* = 3.7 Hz, 1H), 8.33 (s, 1H), 8.39 (s, 1H), 8.41 (s. 1H), 11.51 (br s, 1H, NH), 12.09 (s, 1H, NH). ^13^C NMR (50 MHz, DMSO‐*d*_6_) *δ*: 55.3 (q), 93.5 (d), 103.6 (d), 108.2 (s), 113.0 (d), 124.1 (2xd), 125.7 (s), 128.4 (2xd), 129.1 (2xd), 130.6 (2xd), 131.9 (d), 132.1 (s), 139.8 (s), 146.4 (s), 150.3 (s), 150.7 (s), 154.8 (s), 155.7 (s), 157.6 (s), 158.3 (s), 162.0 (s). *Anal.* Calculated for C_27_H_19_N_7_O_4_ (MW: 505.48): C, 64.15; H, 3.79; N, 19.40%. Found: C, 64.31; H, 3.66; N, 19.31%.

##### N‐[7‐(1‐methyl‐1H‐indol‐3‐yl)‐2‐phenyl‐pyrazolo[1,5‐a][1,3,5]triazin‐4‐yl]‐4‐nitro‐benzamide (6c)

Yield: 68%; red solid; m.p.: 278.2–282.4 °C. IR (cm^−1^): 3200 (NH), 1734 (CO), 1559 (NO); ^1^H NMR (200 MHz, DMSO‐*d*_6_) *δ*: 3.88 (s, CH_3_), 7.06 (s, 1H), 7.17 (t, *J* = 7.4 Hz, 1H), 7.30–7.22 (m, 1H), 7.46 (t, *J* = 7.4 Hz, 2H), 7.57–7.50 (m, 2H), 8.11 (d, *J* = 1.4 Hz, 1H), 8.13 (s, 1H), 8.15 (s), 8.27 (d, *J* = 8.5 Hz, 1H), 8.45–8.40 (m, 1H), 8.51 (s, 1H), 11.98 (s, 1H, NH). ^13^C NMR (50 MHz, DMSO‐*d*_6_) *δ*: 33.3 (d), 107.5 (s), 110.6 (d), 120.9 (d), 122.6 (d), 123.5 (s), 124.2 (2xd), 125.6 (s), 128.3 (2xd), 129.0 (2xd), 130.5 (2xd), 131.8 (d), 131.9 (d), 134.8 (d), 136.7 (s), 137.7 (s), 138.7 (s), 140.5 (s), 150.1 (s), 150.4 (s), 154.5 (s), 155.1 (s). *Anal.* Calculated for C_27_H_19_N_7_O_3_ (MW: 489.48): C, 66.25; H, 3.91; N, 20.03%. Found: C, 66.12; H, 3.88; N, 19.88%.

##### N‐[7‐(5‐bromo‐1‐methyl‐1H‐indol‐3‐yl)‐2‐phenyl‐pyrazolo[1,5‐a][1,3,5]triazin‐4‐yl]‐4‐nitro‐benzamide (6d)

Yield: 85%; orange solid; m.p.: 338.3–340.3 °C. IR (cm^−1^): 3316 (NH), 1717 (CO), 1541 (NO); ^1^H NMR (200 MHz, DMSO) *δ*: 3.88 (s, 3H, CH_3_), 7.05 (s, 1H), 7.36 (dd, *J* = 8.7, 1.8 Hz, 1H), 7.59–7.44 (m, 4H), 8.16 (d, *J* = 7.5 Hz, 2H), 8.22 (s, 1H), 8.31 (d, *J* = 8.5 Hz, 2H), 8.41 (d, *J* = 8.7 Hz, 2H), 8.62 (s, 1H), 12.24 (s, 1H, NH). *Anal.* Calculated for C_27_H_18_BrN_7_O_3_ (MW: 568.38): C, 57.05; H, 3.19; N, 17.25%. Found: C, 57.09; H, 3.11N, 17.11%.

##### N‐[7‐(5‐chloro‐1‐methyl‐1H‐indol‐3‐yl)‐2‐phenyl‐pyrazolo[1,5‐a][1,3,5]triazin‐4‐yl]‐4‐nitro‐benzamide (6e)

Yield: 77%; orange solid; m.p.: 288.8–291.8 °C. IR (cm^−1^): 3397 (NH), 1724 (CO), 1516 (NO); ^1^H NMR (200 MHz, DMSO) *δ*: 3.89 (s, 3H, CH_3_), 7.05 (s, 1H), 7.25 (dd, *J* = 8.7, 2.1 Hz, 1H), 7.47 (t, *J* = 7.4 Hz, 2H), 7.59–7.51 (m, 2H), 8.15–8.10 (m, 2H), 8.23 (s, 1H), 8.30 (d, *J* = 8.6 Hz, 2H), 8.41 (d, *J* = 8.8 Hz, 2H), 8.50 (s, 1H), 12.23 (s, 1H, NH). *Anal.* Calculated for C_27_H_18_ClN_7_O_3_ (MW: 52.93): C, 61.90; H, 3.46;N, 18.71%. Found: C, 61.81; H, 3.34; N, 18.62%.

##### N‐[7‐(5‐Fluoro‐1‐methyl‐1H‐indol‐3‐yl)‐2‐phenyl‐pyrazolo[1,5‐a][1,3,5]triazin‐4‐yl]‐4‐nitro‐benzamide (6f)

Yield: 99%; yellow solid; m.p.: 288.8–291.8 °C. IR (cm^−1^): 3341 (NH), 1717 (CO), 1541 (NO); ^1^H NMR (200 MHz, DMSO) *δ*: 3.88 (s, 3H, CH_3_), 7.04 (s, 1H), 7.10 (td, *J* = 9.2, 2.6 Hz, 1H), 7.43 (t, *J* = 7.5 Hz, 2H), 7.55‐ 7.50 (m, 2H), 8.08–8.03 (m, 2H), 8.28–8.20 (m, 3H), 8.33 (s, 1H), 8.43–8.37 (m, 2H), 12.13 (s, 1H, NH). *Anal.* Calculated for C_27_H_18_FN_7_O_3_ (MW: 507.48): C, 63.90; H, 3.58; N, 19.32%. Found C, 63.81; H, 3.51; N, 19.38%.

##### N‐[7‐(5‐Methoxy‐1‐methyl‐1H‐indol‐3‐yl)‐2‐phenyl‐pyrazolo[1,5‐a][1,3,5]triazin‐4‐yl]‐4‐nitro‐benzamide (6g)

Yield: 89%; red solid; m.p.: 301.6–304.8 °C. IR (cm^−1^): 3376 (NH), 1734 (CO), 1542 (NO); ^1^H NMR (200 MHz, DMSO) *δ*: 3.57 (s, 3H, CH_3_), 3.85 (s, 3H, OCH_3_), 6.85 (dd, *J* = 8.8, 2.5 Hz, 1H), 7.06 (s, 1H), 7.41 (d, *J* = 8.9 Hz, 1H), 7.60–7.49 (m, 3H), 7.86 (d, *J* = 2.4 Hz, 1H), 8.11 (s, 1H), 8.32–8.26 (m, 3H), 8.33 (s, 1H), 8.42–8.38 (m, 2H), 12.09 (s, 1H, NH). *Anal.* Calculated for C_28_H_21_N_7_O_4_ (MW: 519.51): C, 64.73; H, 4.07; N, 18.87%. Found: C, 64.66; H, 4.01; N, 18.71%.

##### Biology: Cell Culture

Three lines of immortalized PDAC cells were selected as representative phenotypes: mesenchymal (PATU‐T), quasimesenchymal (PANC‐1), and an epithelial clone of SUIT 2 cells (SUIT 2.28). Cells were cultured in noncoated T‐75 and T‐25 flasks under adherent conditions, using 1× DMEM (Gibco, 41,966,029) for PATU‐T and RPMI (Gibco, 21,875,034) for SUIT 2.28 and PANC‐1, both supplemented with 10% NBCS and 1% P/S. Additional employed models were the PDAC3 cells, a primary cell culture, the PSCs, which were immortalized healthy pancreatic cells, and the PATU‐T‐GR, which was a gemcitabine resistant model of PATU‐T. PDAC3 and PSCs were cultivated using complete RPMI medium, while PATU‐T‐GR required complete DMEM medium. For seeding, cells were detached with Trypsin‐EDTA (0.05%) (GibcoTM, 25,300–096) and were consequently counted using the Automated Cell Counter (Luna‐II, Westburg). Cells were then plated on 96‐ or 6‐well plates depending on the planned experiment.

##### SRB Assay

The Sulforhodamine B (SRB) chemosensitivity assay was used to measure cytotoxicity and cell proliferation post drug treatment. At day 0, 3–6 × 10^3^ cells were seeded in a 96‐well plate, depending on the cell line. At day 1, cells were attached and ready to be treated. For the calculation of the IC_50_, drug dilutions for the compounds **1b, 1c, 1d, 1e, 1g, 1hr, 1i, 1j,** and LDC4297 (MedChemExpress, HY‐12,653) were prepared in dilution trays and eight different concentrations were included. These were added in triplicates to the cells, which were then incubated for h at 37 °C in 5% CO_2_. During day 1, the control plate was fixed with 50% Trichloroacetic acid (TCA), as intended to be the baseline plate used for the normalization of the data. 72 h post‐treatment, the cells were fixed with TCA, and placed at 4 °C for about 2 h. The plates were then washed with distillated water and left to dry under the hood with ventilation. Subsequently, plates were stained with 0.4% SRB for 15 min and washed with 1% acetic acid to remove excess stain. Once dried again, the protein‐bound SRB was dissolved in 10 mM TRIS, and the plates were placed on a shaker for 10 min per plate. Lastly, the absorbance was measured at 492 nm with a spectrophotometer (Synergy HT, BioTek). The absorbance data obtained were normalized to the day 1 control, and the percentage of cell growth in relation to the control was calculated using the following formula.

(1) $$\%\text{Cell growth}=\frac{\text{Mean OD treatment}-\text{Mean OD day}0}{\text{Mean OD control}-\text{Mean OD day}0}\times 100$$

The percentage of proliferation and their respective standard deviations were used to perform statistical analysis in GraphPad Prism software v 10 (GraphPad Software, San Diego, CA). Data were normalized to the day 0 control and to their own untreated 72 h control. These were transformed into logarithmic data, and log(inhibitor) versus response analysis was performed for obtaining the IC_50_ values per cell lines. Each experiment was conducted in triplicates.

##### Wound Healing Assay

The wound healing assay was used to test the migratory ability of the selected cells post‐treatment. Cells (5 × 10^4^ /well) were seeded on 96‐well. After 24 h, cells reached the optimal 100% confluency, and an artificial wound was created with a 96‐well Pintool Scratcher. The cells were immediately washed with complete medium, in order to remove cell debris after the scratch, and images of the wells were captured at time 0 h, right before the addition of the treatment. Each drug (**1b** and **1g**) was previously diluted in the respective medium at two different concentrations: 1 × IC_50_ and 4 × IC_50_ (according to the cell specific IC_50_ value) and were then added to the cells. Images were then taken at timepoints 4, 8, 20, and 24 h using the Leica microscope with a JAI TMC‐137 camera to assess wound closure. The images were analyzed using the Universal Grab 6.3 software (DCILabs), and the obtained data were used to determine the percentage of migration of the cells, using the following formula.

(2) $$\text{\% Migration}=\frac{\text{Mean width of the wound at}\;T=0-\text{Mean width of the wound at}\;T=X}{\text{Mean width of the wound at}\;T=0}\times 100$$

##### Apoptosis Assay with Annexin V

In order to investigate the apoptotic properties of the compounds **1b** and **1g**, the Annexin V apoptosis assay was performed. Annexins are calcium‐dependent phospholipid binding proteins, which are able to bind to phosphatidylserine (PS). The externalization of PS residues on the outer membrane of apoptotic cells can be detected by Annexin V.

For this assay, cells were seeded in a 96‐well plate at a concentration of 13.5 × 10^3^ and 4.5 × 10^3^ /well respectively for the 24 and 72 h experiments. The next day cells were treated with compounds **1b** and **1g** at 1 × IC_50_ and 4 × IC_50_ concentrations. The apoptosis assay was performed 24 and 72 h post‐treatment following the BD Pharmingen FITC Annexin V Apoptosis Detection Kit I (BD Biosciences, 556,547). The supernatant was aspirated, and cells were washed with phosphate‐buffered saline (PBS). Cells were consequently treated with binding buffer BBA + Annexin V (1:100) and were incubated in the dark at RT for 10 min. The supernatant was removed, and the cells were washed with BBA (1:10). Cells were then incubated with BBA + PI (1:100) and were immediately read with the spectrophotometer at ex. 485/ em. 535. Two different programs were used to read the Annexin V and the PI absorbance. Lastly, 100 µL of PBS were added to each well before being fixed with TCA. This was followed by the SRB assay to standardize fluorescence data according to cell count. Data were exported into GraphPad Prism 10 for statistical analysis.

##### RNA Isolation and RT‐PCR

In order to understand modulation of downstream genes of CDK7, gene expression analysis by qPCR was performed in all cell lines. Cells (5 × 10^5^/well) were seeded in 6‐well plates and treated with the compounds **1b** and **1g** at 1× and 4 × IC_50_ concentrations. Cell lysates were collected 24 h post‐treatment using 300 µL of TRIzol Reagent (Ambion, 15,596,018) per well. Total RNA was extracted by mixing 60 µL of chloroform to the samples, incubating for 3 min and centrifuging at 12,000 rcf for 15 min at 4 °C. The upper aqueous phase containing RNA was transferred to a new Eppendorf and mixed with 120 µL of cold isopropanol in order to allow the RNA to precipitate. Samples were incubated at RT for 10 min and were then centrifuged at 12,000 rcf for 10 min at 4 °C. The isopropanol was carefully removed, and the pellet was washed twice with 70% ETOH. Pellets were left to air dry and consequently resuspended in RNAse‐free water. Samples were left agitation on a thermomixer at 55 °C for 10 min at 600 rpm and were then quantified through NanoDrop technology (Thermo Scientific, NanoDrop One/One^c^). RNA samples were stored at −80 °C.

In order to perform cDNA synthesis, the First Strand cDNA synthesis kit (Thermo Scientific, K1612) was used. The previously isolated RNA was mixed with 1 µL of oligo dT18 primer into a pcr tube. Nuclease free water was added to reach a final volume of 11 µL. Samples were centrifuged at 1200 rcf for 5 min and were then placed on a thermomixer at 65 °C for a few minutes. A master mix was prepared, including 4 µL of 5× reaction buffer, 1 µL of ribolock RNAse inhibitor, 2 µL of dntp mix (10 mM), and 2 µL of M‐mulv reverse transcriptase for each sample. In total 9 µL of master mix were added to each vial. Samples were centrifuged again and were finally synthesized, following a cycle of 60 min at 37 °C and 5 min at 70 °C. cDNA was now finally synthesized and could be stored at −20 °C. RT‐PCRs reactions were performed using SYBR Green (Bio‐Rad, 1,725,271) (5 µL per sample), primer mix (0.3 µM) (2 µL per sample), and cDNA (10 ng µL^−^^1^) (3 µL per sample). Each sample had a final volume of 10 µL. RT‐PCR assays were carried out in a GeneAmp 5700 Sequence Detection System programmed to hold at 50 °C for 30 min, to hold at 95 °C for 10 min, and to complete 45 cycles of 95 °C for 15 min and 50 °C for 1 min. The genes that were investigated are shown in **Table** **5**, with their respective forward and reverse primer sequences.

**Table 5:** Genes of interest and their respective primer sequences. GusB (Glucuronidase beta, housekeeping); CDK7 (Cyclin‐dependent kinase 7); Bcl‐2 (B‐cell lymphoma 2, regulates cell deatch); Bax (Bcl‐2–associated X, apoptosis regulator); cMYC (Cellular myelocytomatosis, proto‐oncogene); CCND1 (Cyclin‐D1, cell cycle regulator).

Primers	Forward sequence	Reverse Sequence
GusB	CACCAGGATCCACCTCTGAT	TCCAAATGAGCTCTCCAACC
CDK7	ATGGCTCTGGACGTGAAGTCT	GCGACAATTTGGTTGGTGTTC
Bcl‐2	GATGTGATGCCTCTGCGAAG	CATGCTGATGTCTCTGGAATCT
Bax	GGTTGTCGCCCTTTTCTA	CGGAGGAAGTCCAATGTC
cMYC	GGAGGCTATTCTGCCCATTT	GTCGCAGTAGAAATACGGCT
CCND1	TCTACACCGACAACTCCATCCG	TCTGGCATTTTGGAGAGGAAGTG

##### Spheroids

The modulation of the growth of spheroids was utilized as a method to assess the effect of the novel compounds **1b** and **1g** in a 3D structure. The PATU‐T cell line was employed to make such a structure. Agarose 1.5% is a solid gel at RT; thus, in order to become a liquid solution, it was heated up into a microwave for about 30 s—1 min, depending on when it starts boiling. Once heated up, the bottle containing the agarose was wrapped in aluminum foil, and it was allowed to maintain the temperature and the liquid state of the agarose. Each well of a flat‐bottom 96‐well plate was coated with 70 µL of agarose. Plates were left to cool down and dried for 1 h at RT. Afterward, 7 × 10^3^, 1 × 10^4^, and 2 × 10^4^ cells/well (200 µL) were seeded in order to identify the optimal amount of cell for the spheroid formation. Finally, the plates were centrifuged at 1200 rpm for 6 min and placed in the incubator for 7 days at 37 °C, 5% CO_2_. After one week, spheroids were imaged at day 0 h with the inverted routine microscope Nikon Eclipse Ts2 and were consequently treated with compounds **1b** and **1g** at 4 × IC_50_ and 1 × IC_50_. In order to assess the effects of the treatment, images were then taken every 3 days, and medium along with treatment was refreshed at the same interval. At day 15, spheroids were destroyed with 50 µL of Trypsin. Cells were then collected, counted, and reseeded in 96‐well plates. After a further 7 days, a resazurin staining was conducted to evaluate cell viability, since the colorimetric signal was proportional to the number of living cells in the sample. 20 mg uL^−^^1^ of resazurin solution was added to each well and incubated at 37 °C 5% CO_2_ for 1 h. Absorbance was read at 570 nm.

##### Cell Cycle Analysis (FACS)

Modulation of the cell cycle was analyzed via flow cytometry. Cells (5 × 10^5^ cells mL^−^^1^) were seeded in 6‐well plates and were incubated at 37 °C overnight. Cells were treated with the compounds **1b** and **1g** at two different concentrations (1 × IC_50_ and 4 × IC_50_, according to each cell line) and were again incubated for 24 and 72 h. After treatment, cells were harvested with trypsin and were collected in specific fluorescence‐activated cell sorting (FACS) tubes. Cells were then washed with PBS and centrifuged at 1500 rpm for 5 min, in order to form a pellet. These pellets were fixed in 1 mL of ice‐cold 70% ethanol, added drop by drop during vortex, and placed in the refrigerator at 4 °C. After overnight incubation, the cells were washed twice with PBS, centrifuged (5 min at 1500 rpm), resuspended in 50 µL of RNase (100 µg mL^−^^1^), and incubated for 30 min at 37 °C. Finally, 200 µL of propidium iodide solution (PI, 50 µg mL^−^^1^) was added, and the cell distribution in the various phases of the cell cycle was analyzed via BD LSRFortessa Cell Analyzer. Data analysis was carried out with FACSdiva software (Becton Dickinson), while cell cycle distribution was determined using FCS express software.

##### Kinase Activity Profiling using the PamChip Platform

A Kinase activity profiling was conducted using a PamChip (Pamgene, s‐Hertogenbosch, The Netherlands) array containing 144 peptide substrates specific to tyrosine kinases to assess activity changes upon treatment with compounds 1b and 1g. Experiments were carried out in PDAC3 cells, using biological triplicates comprising three untreated controls and three samples treated for 2 h with either compound at their respective 1 × IC_50_ concentrations, following protocols adapted from previous studies.^[^ ^38^ ^]^ Protein concentrations were quantified using the Bio‐Rad protein assay, which is based on the Bradford method (Bio‐Rad, Hercules, CA). For chip loading, protein lysates were adjusted to a final concentration of 10 µg per array and combined with PamGene MasterMix. During the assay, samples were circulated through the array at 30 °C with one cycle per minute over 60 cycles. Fluorescence data acquisition was performed using a 12‐bit CCD camera, capturing real‐time fluorescence intensities. End‐point spot intensities were analyzed using the open‐source software ScanAnalyze, with background normalization applied. Statistical comparisons were performed using student's *t*‐test in R (version 3.6.1), followed by false discovery rate (FDR) correction. Peptides with an FDR below 0.01 were considered significantly differentially phosphorylated.

##### Enzyme‐Linked Immunosorbent Assay (ELISA)

To detect CDK7 and phospho‐CDK7 levels at Thr170 residue after treatment, an enzyme‐linked immunosorbent assay (Biomatik, Phospho‐CDK7 (Thr170) Colorimetric Cell‐Based ELISA Kit, Cat# EKA51274) was performed according to the manufacturer's protocols. Cells (2 × 10^5^) were seeded on the 96‐well plate and were evaluated after 24 h from the treatment with the selected compounds 1b and 1g at 1 × IC_50_ concentration. The absorbance was read at 450 nm using a microplate reader.

##### CDK7 Enzyme Inhibition Assay

The CDK7 enzyme inhibition assay was conducted using the ADP‐Glo Kinase Assay (VA7402, Promega Corporation, Madison, WI, USA) according to the manufacturer's instructions. In this assay, CDK7 catalyzes the conversion of ATP to ADP. The reaction was terminated by adding the ADP‐Glo reagent, which depletes residual ATP. Subsequently, the kinase detection reagent was introduced to convert ADP back into ATP, which was then measured via a luciferase reaction, producing luminescence. The assay was carried out at room temperature, using DMSO as the solvent for the tested compounds. Each compound was tested at eight different concentrations. LDC4297, a well‐established CDK7 inhibitor, was used as the reference compound for comparison. Luminescence was recorded using a BioTek plate reader (BioTek Instruments Inc.), as previously described.^[^ ^39^ ^]^ The percentage of enzyme inhibition was determined for each compound at different concentrations, and mean IC_50_ values of triplicate experiments were calculated using GraphPad Prism.

##### Statistical Analysis

Experiments were conducted in triplicates. The data was evaluated using the GraphPad Prism software v 10 (GraphPad Software, San Diego, CA). Data was expressed as mean values ± standard error of the mean (SEM) and analyzed by one way analysis of variance (ANOVA) test. *P* values < 0.05 were considered significant (*).

## Conflict of Interest

The authors declare no conflict of interest.

## Supporting information

Supplementary Material

## Data Availability

The data that support the findings of this study are available in the supplementary material of this article.
