# Supplementary material for: Novel Pyrazolo [1,5‐a]−1,3,5‐Triazine Derivatives as CDK7 Inhibitors: Synthesis and Biological Insights in Pancreatic Ductal Adenocarcinoma Models
Source: ChemMedChem. 2025 Aug 25;20(19):e202500448. doi: 10.1002/cmdc.202500448 (PMC12503906; doi:10.1002/cmdc.202500448)
Supplement: Supplementary file 1 — Supplementary Material [file CMDC-20-e202500448-s001.pdf]

Supporting Information

Novel pyrazolo [1,5-a]-1,3,5-triazine derivatives as CDK7 inhibitors: synthesis and biological insights in PDAC models

Daniela Carbone[a], Francesca Terrana[a], Ludovica Sciuto[b], Camilla Pecoraro[a], Geng Xu[b], Stella Cascioferro[a], Girolamo Cirrincione[a], Godefridus J. Peters[b], Elisa Giovannetti[b,c,*], Barbara Parrino[a,*] and Patrizia Diana[a]

[a] Department of Biological, Chemical, and Pharmaceutical Sciences and Technologies (STEBICEF), University of Palermo, Viale delle Scienze Ed.16, 90128 Palermo, Italy

[b] Department of Medical Oncology, Cancer Center Amsterdam, Amsterdam UMC, VU University, Amsterdam 1081 HV, The Netherlands

[c] Cancer Pharmacology Laboratory, Fondazione Pisana per la Scienza, Via Ferruccio Giovannini 13, 56017 Pisa, Italy

[*]Correspondence: barbara.parrino@unipa.it, elisa.giovannetti@gmail.com

Supplementary Table 1: P-values indicating statistically significant differences in migration of PATU-T cells treated vs. untreated over time (0h, 4h, 8h, 20h, 24h)
****p<0.0001. ***p<0.001. **p<0.01. *p<0.05. ns = non-significant.

COMPOUNDS	1b		1c		1d		1e		1g		1h		1i		1j	
Time (h)	$1xIC_{50}$	$4xIC_{50}$	$1xIC_{50}$	$4xIC_{50}$	$1xIC_{50}$	$4xIC_{50}$	$1xIC_{50}$	$4xIC_{50}$	$1xIC_{50}$	$4xIC_{50}$	$1xIC_{50}$	$4xIC_{50}$	$1xIC_{50}$	$4xIC_{50}$	$1xIC_{50}$	$4xIC_{50}$
4h	***	****	**	****	****	****	****	****	**	***	*	***	***	****	***	****
8h	****	****	****	****	****	****	****	****	****	***	***	***	****	****	****	****
20h	****	****	****	****	****	****	****	****	****	****	***	****	****	****	****	****
24h	****	****	****	****	****	****	****	****	****	****	***	****	****	****	****	****

Supplementary Table 2: P-values indicating statistically significant differences in migration of SUIT 2.28 cells treated vs. untreated over time (0h, 4h, 8h, 20h, 24h)
****p<0.0001. ***p<0.001. **p<0.01. *p<0.05. ns = non-significant.

COMPOUNDS	1b		1c		1d		1e		1g		1h		1i		1j	
Time (h)	$1xIC_{50}$	$4xIC_{50}$	$1xIC_{50}$	$4xIC_{50}$	$1xIC_{50}$	$4xIC_{50}$	$1xIC_{50}$	$4xIC_{50}$	$1xIC_{50}$	$4xIC_{50}$	$1xIC_{50}$	$4xIC_{50}$	$1xIC_{50}$	$4xIC_{50}$	$1xIC_{50}$	$4xIC_{50}$
4h	ns	ns	ns	ns	ns	ns	ns	ns	ns	ns	ns	ns	ns	ns	ns	ns
8h	ns	ns	ns	ns	ns	ns	ns	ns	ns	ns	ns	ns	ns	ns	ns	ns
20h	ns	*	ns	ns	ns	ns	ns	ns	***	***	*	**	ns	ns	*	*
24h	ns	***	*	**	ns	***	ns	*	***	****	**	****	ns	ns	*	****

Supplementary Table 3: P-values indicating statistically significant differences in migration of PATU-T GR cells treated vs. untreated over time (0h, 4h, 8h, 20h, 24h)
****p<0.0001. ***p<0.001. **p<0.01. *p<0.05. ns = non-significant.

COMPOUNDS	1b		1g	
Time (h)	$1xIC_{50}$	$4xIC_{50}$	$1xIC_{50}$	$4xIC_{50}$
4h	*	*	ns	*
8h	***	***	**	**
20h	****	***	**	****
24h	****	**	*	**

Supplementary Table 4: P-values indicating statistically significant differences in migration of PDAC3 cells treated vs. untreated over time (0h, 4h, 8h, 20h, 24h)
****p<0.0001. ***p<0.001. **p<0.01. *p<0.05. ns = non-significant.

COMPOUNDS	**1b**		**1g**	
Time (h)	1xIC_{50}	4xIC_{50}	1xIC_{50}	4xIC_{50}
4h	ns	ns	ns	ns
8h	ns	ns	ns	ns
20h	ns	ns	ns	ns
24h	**	***	**	**

Spectra of Compounds 1-6

1a ^{1}H NMR

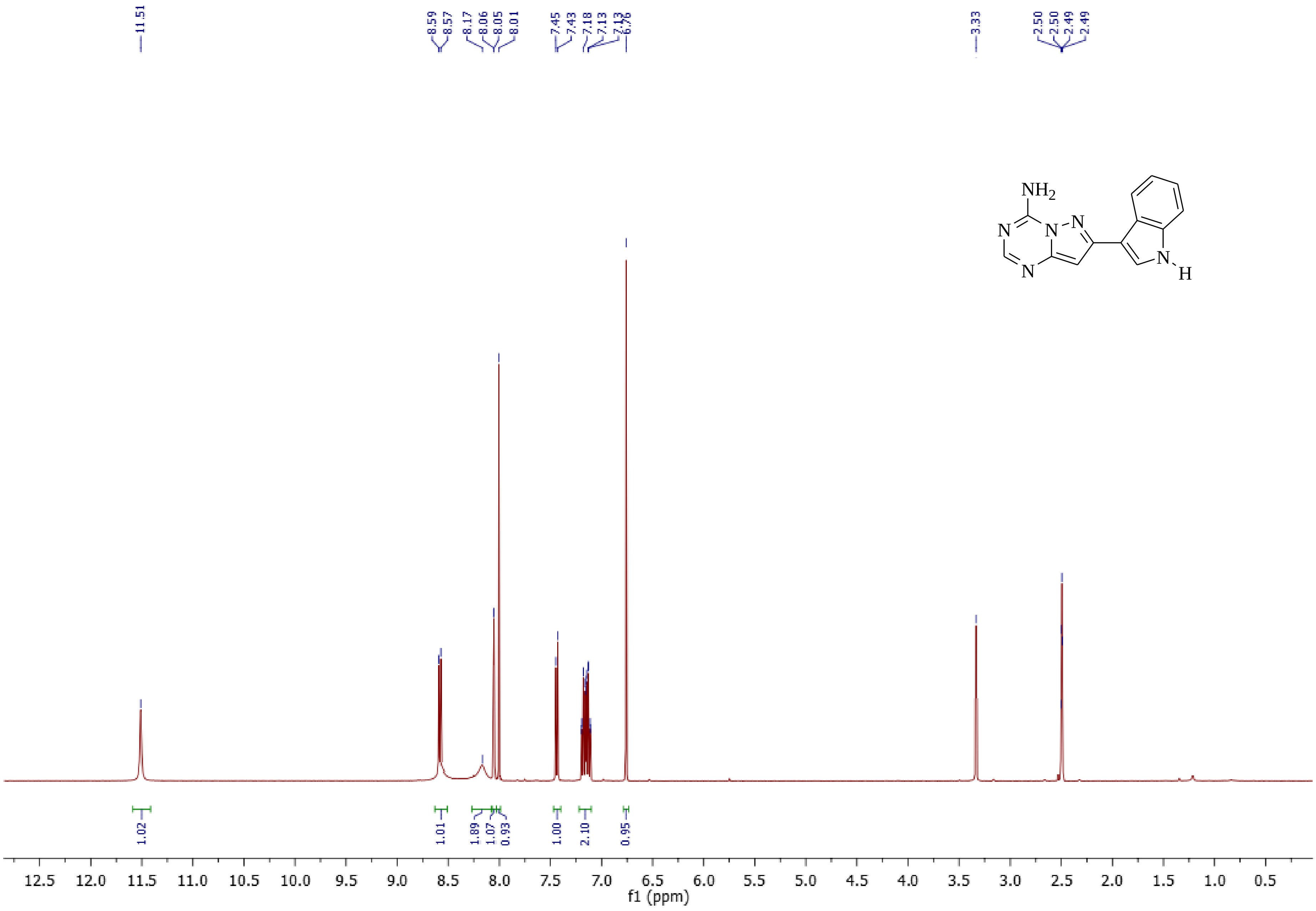
11.51
8.59
8.57
8.17
8.06
8.05
8.01
7.45
7.43
7.18
7.13
7.13
6.76
3.33
2.50
2.50
2.49
2.49
NH2
N
N
N
N
N
H
1.02
1.01
1.89
1.07
0.93
1.00
2.10
0.95
12.5 12.0 11.5 11.0 10.5 10.0 9.5 9.0 8.5 8.0 7.5 7.0 6.5 6.0 5.5 5.0 4.5 4.0 3.5 3.0 2.5 2.0 1.5 1.0 0.5
f1 (ppm)

1a ^{13}C NMR

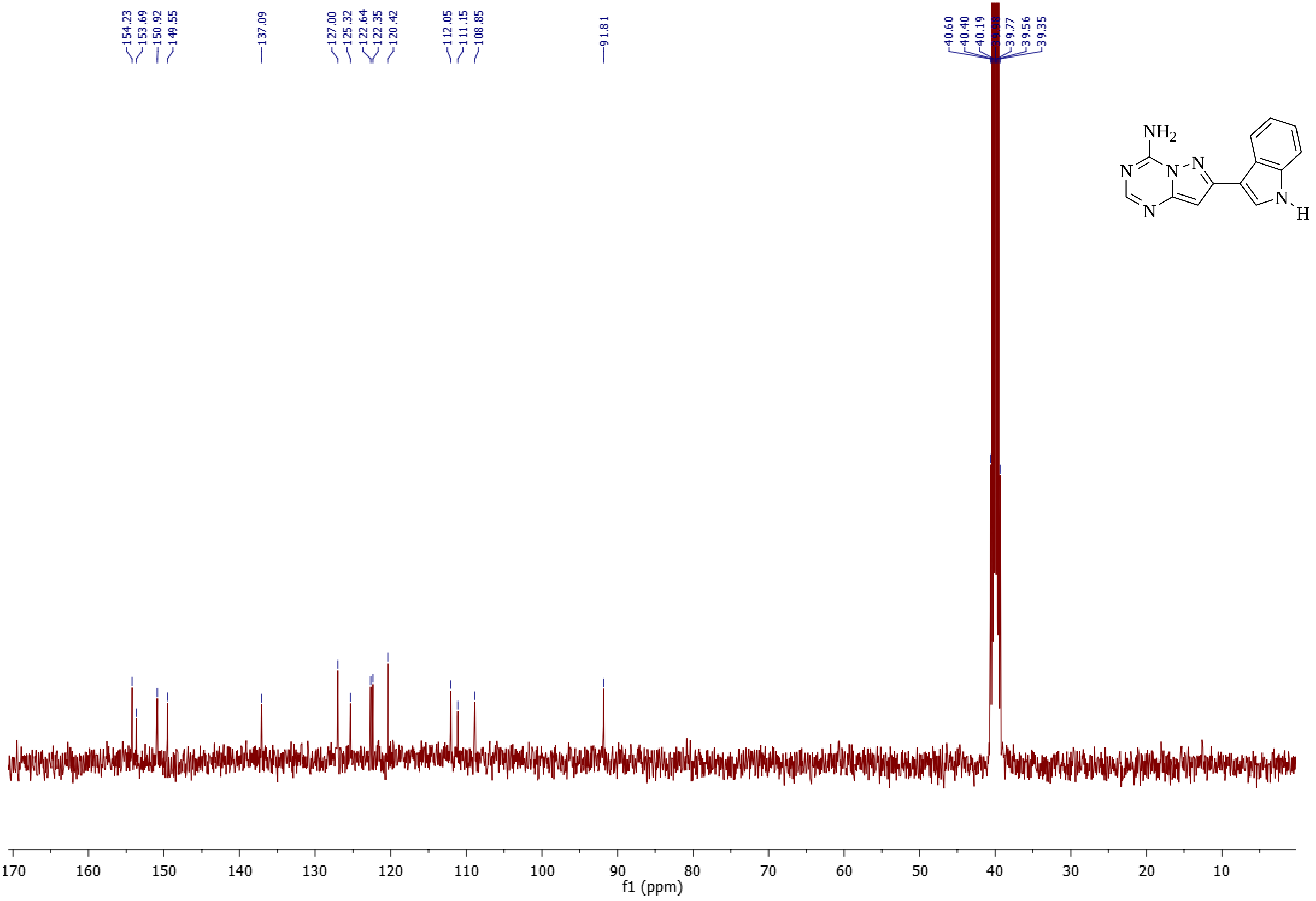

154.23
153.69
150.92
149.55
137.09
127.00
125.32
122.64
122.35
120.42
112.05
111.15
108.85
91.81
40.60
40.40
40.19
39.77
39.56
39.35
NH2
N
N
N
N
N
H
170 160 150 140 130 120 110 100 90 80 70 60 50 40 30 20 10
f1 (ppm)

1b ^{1}H NMR

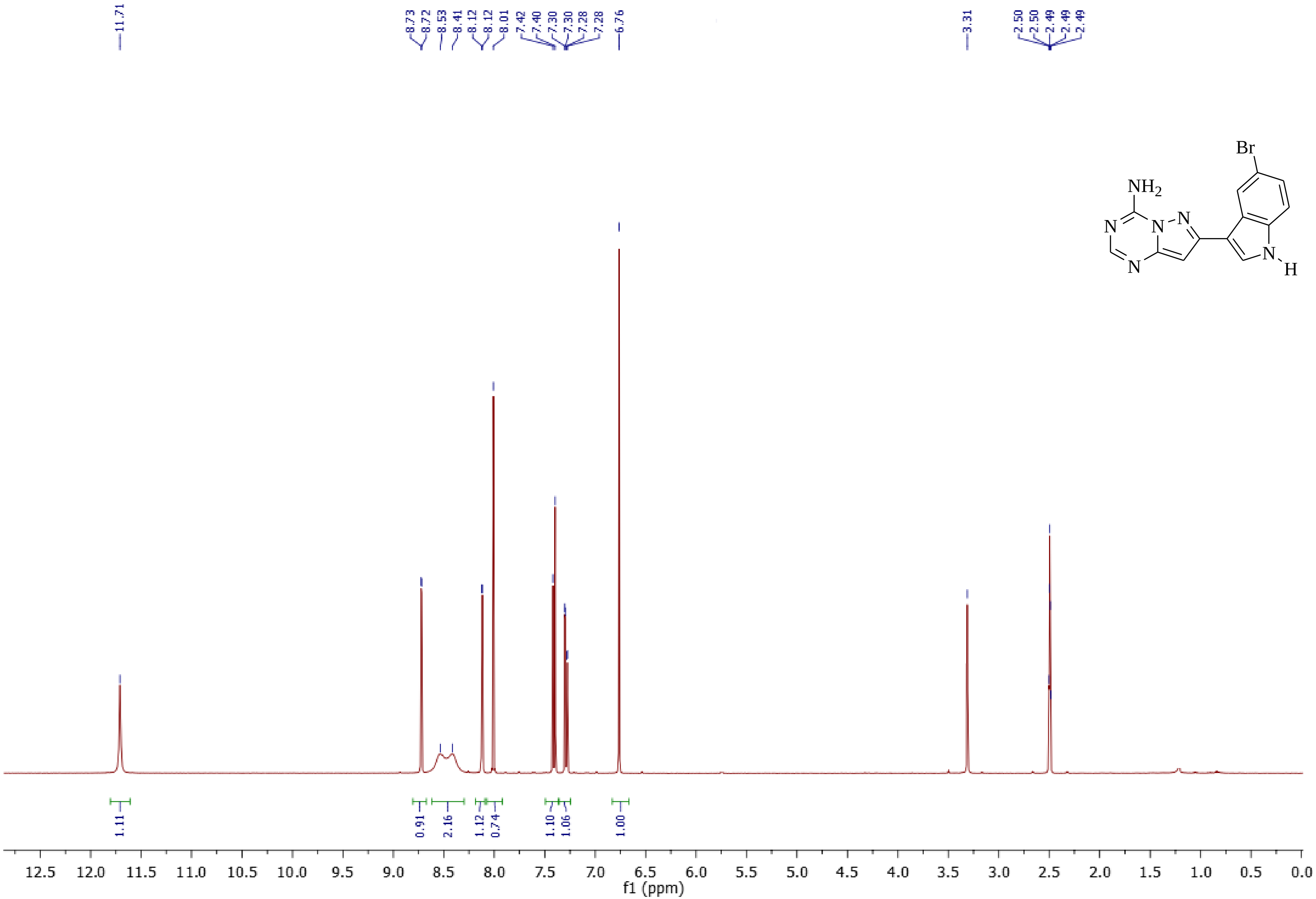
11.71
8.73
8.72
8.53
8.41
8.12
8.12
8.01
7.42
7.40
7.30
7.30
7.28
7.28
6.76
3.31
2.50
2.50
2.49
2.49
2.49
Br
NH2
N
N
N
N
N
N
H
1.11
0.91
2.16
1.12
0.74
1.10
1.06
1.00
12.5
12.0
11.5
11.0
10.5
10.0
9.5
9.0
8.5
8.0
7.5
7.0
6.5
6.0
5.5
5.0
4.5
4.0
3.5
3.0
2.5
2.0
1.5
1.0
0.5
0.0
f1 (ppm)

1b ^{13}C NMR

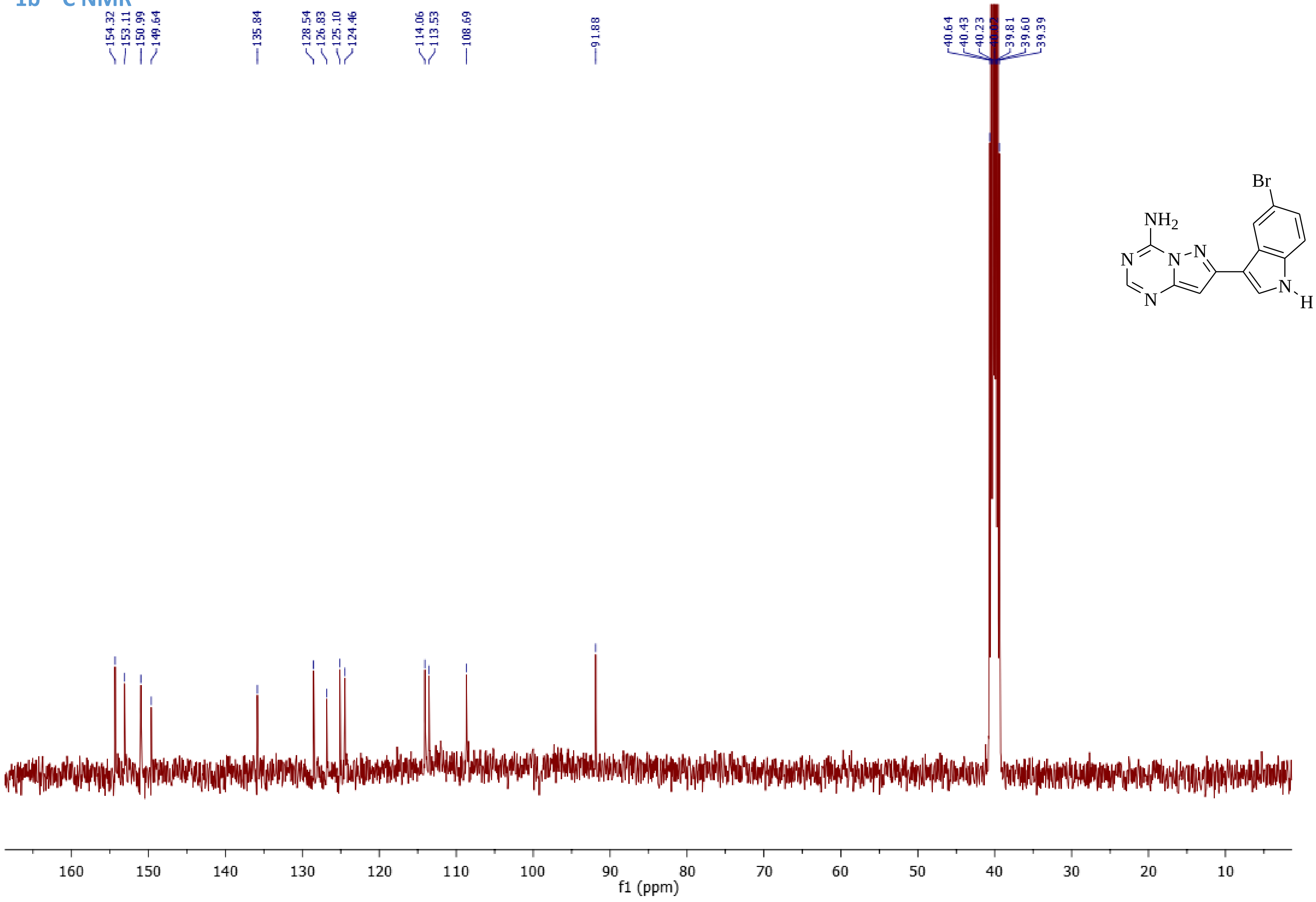

154.32
153.11
150.99
149.64
135.84
128.54
126.83
125.10
124.46
114.06
113.53
108.69
91.88
40.64
40.43
40.23
40.02
39.81
39.60
39.39
Br
NH2
N
N
N
N
N
N
H
160
150
140
130
120
110
100
90
80
70
60
50
40
30
20
10
f1 (ppm)

1b HRMS

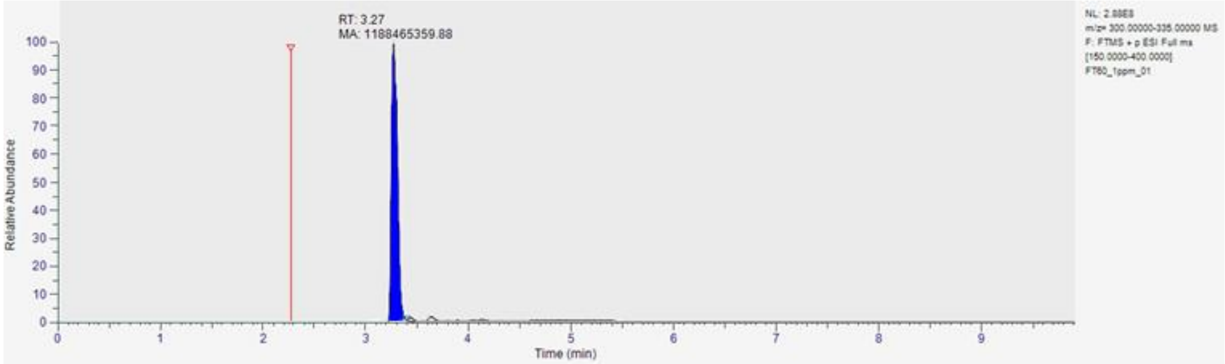
RT: 3.27
MA: 1188465359.88
NL: 2.88E8
m/z= 300.00000-335.00000 MS
F: FTMS + p ESI Full ms
[150.0000-400.0000]
FT60_1ppm_01
Relative Abundance
Time (min)

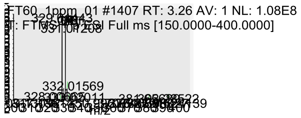
FT60_1ppm_01 #1407 RT: 3.26 AV: 1 NL: 1.08E8
332.01569

1c ^{1}H NMR

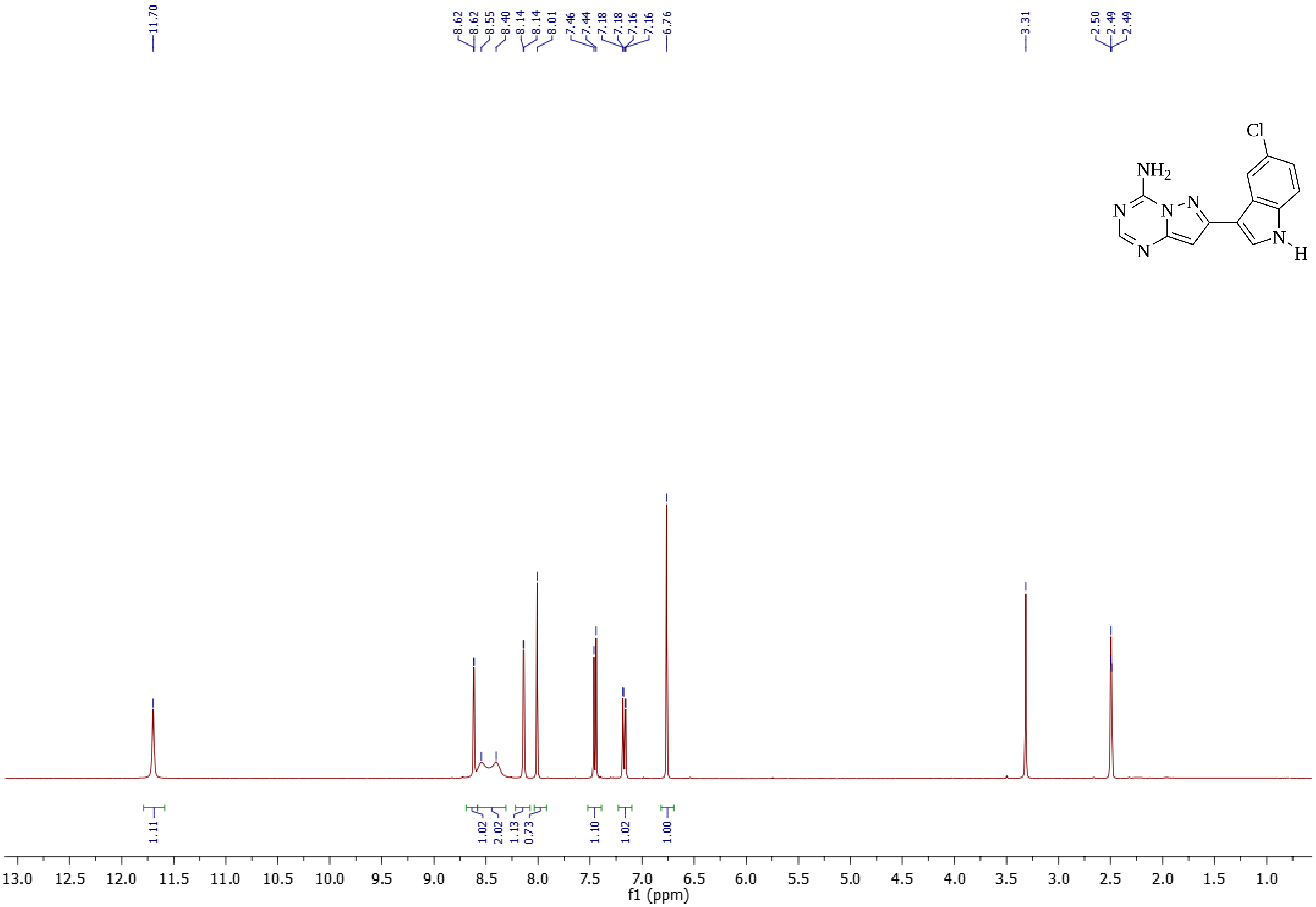

11.70
8.62
8.62
8.55
8.40
8.14
8.14
8.01
7.46
7.44
7.18
7.18
7.16
7.16
6.76
3.31
2.50
2.49
2.49
Cl
NH2
N
N
N
N
N
H
1.11
1.02
2.02
1.13
0.73
1.10
1.02
1.00
13.0 12.5 12.0 11.5 11.0 10.5 10.0 9.5 9.0 8.5 8.0 7.5 7.0 6.5 6.0 5.5 5.0 4.5 4.0 3.5 3.0 2.5 2.0 1.5 1.0
f1 (ppm)

1c ^{13}C NMR

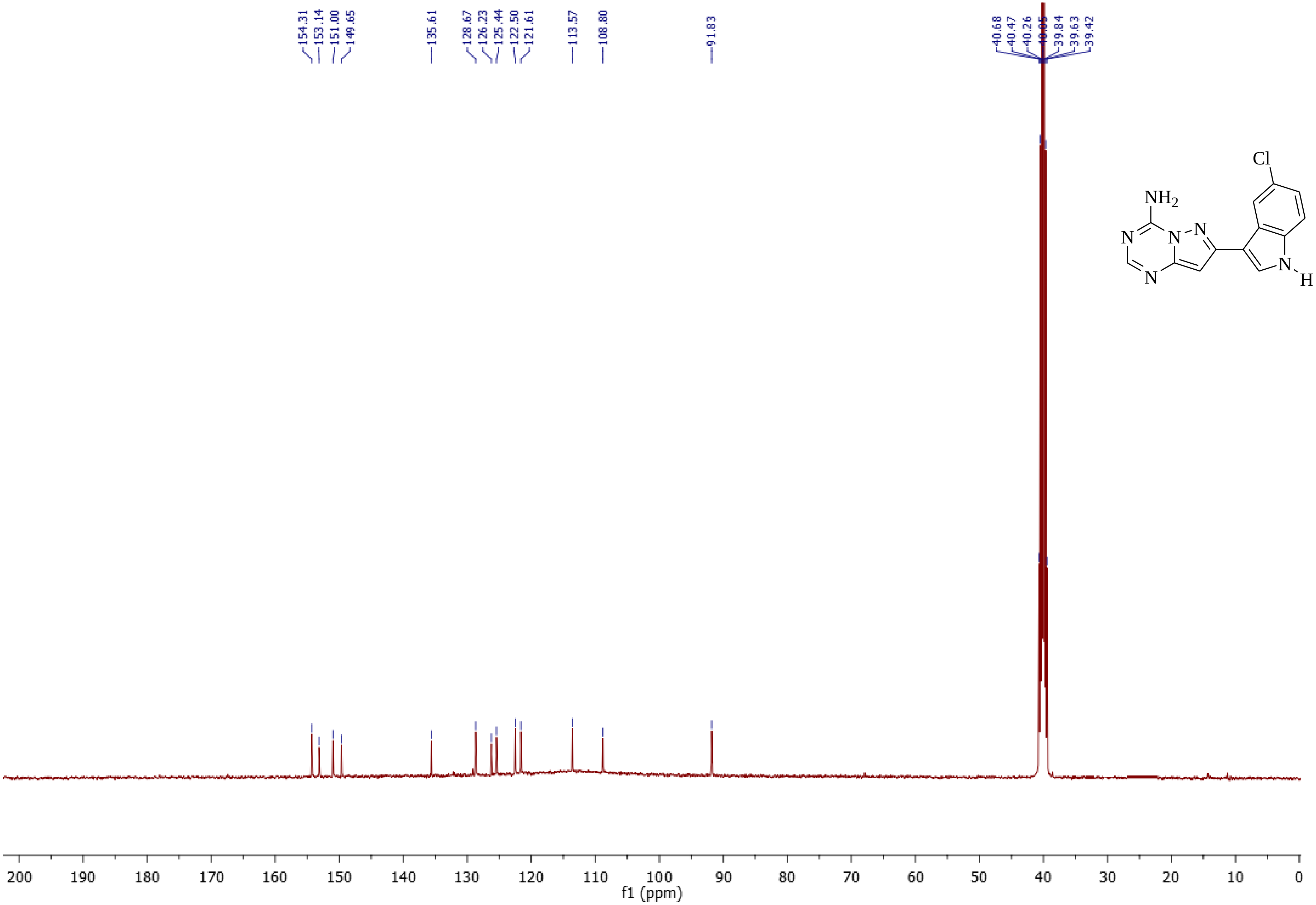
154.31
153.14
151.00
149.65
135.61
128.67
126.23
125.44
122.50
121.61
113.57
108.80
91.83
40.68
40.47
40.26
40.05
39.84
39.63
39.42
NH2
N
N
N
N
Cl
N
H
200 190 180 170 160 150 140 130 120 110 100 90 80 70 60 50 40 30 20 10 0
f1 (ppm)

1d ^{1}H NMR

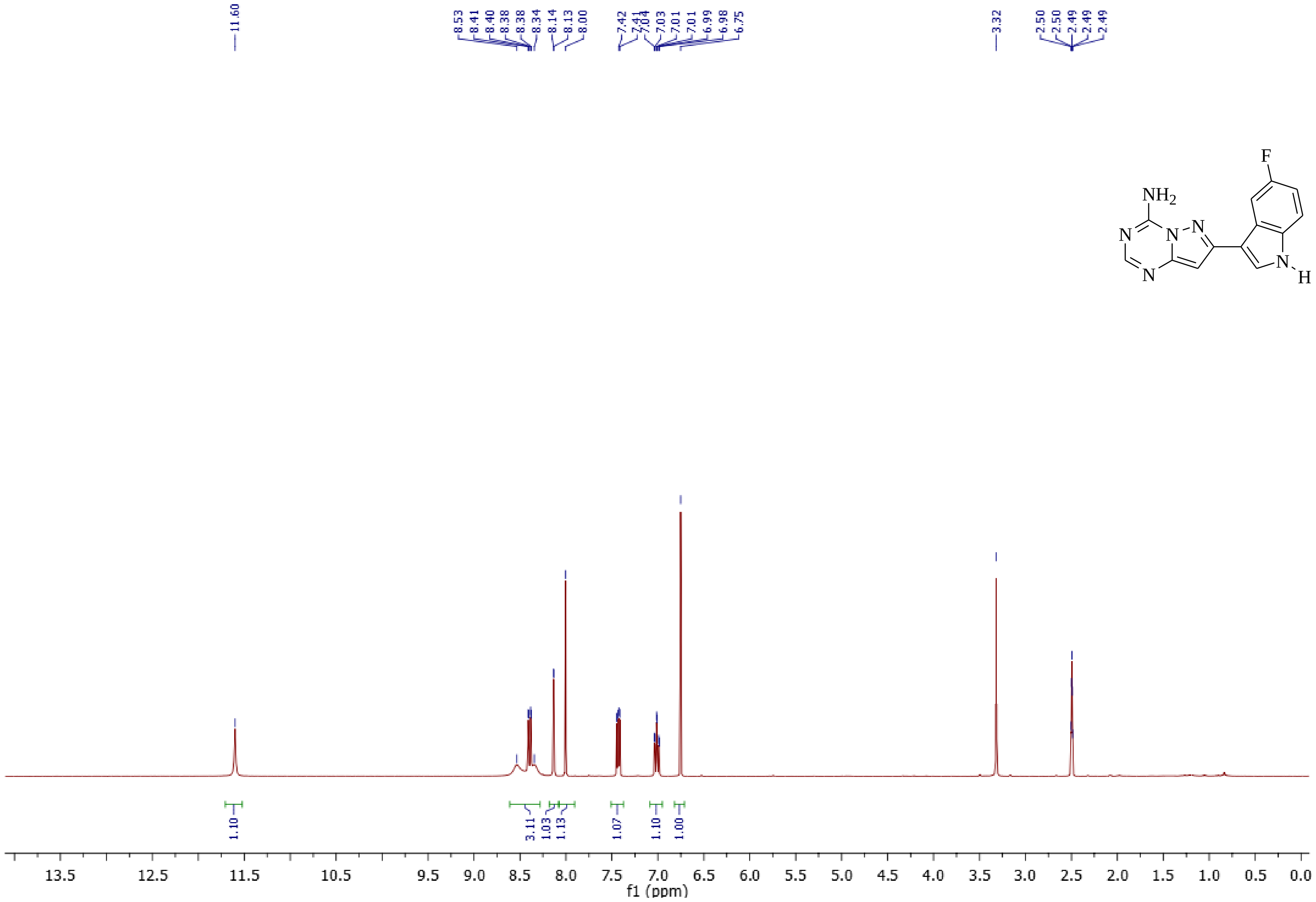
11.60
8.53
8.41
8.40
8.38
8.38
8.34
8.14
8.13
8.00
7.42
7.41
7.04
7.03
7.01
7.01
6.99
6.98
6.75
3.32
2.50
2.50
2.49
2.49
2.49
F
NH2
N
N
N
N
N
H
1.10
3.11
1.03
1.13
1.07
1.10
1.00
13.5
12.5
11.5
10.5
9.5
9.0
8.5
8.0
7.5
7.0
6.5
6.0
5.5
5.0
4.5
4.0
3.5
3.0
2.5
2.0
1.5
1.0
0.5
0.0
f1 (ppm)

1d ^{13}C NMR

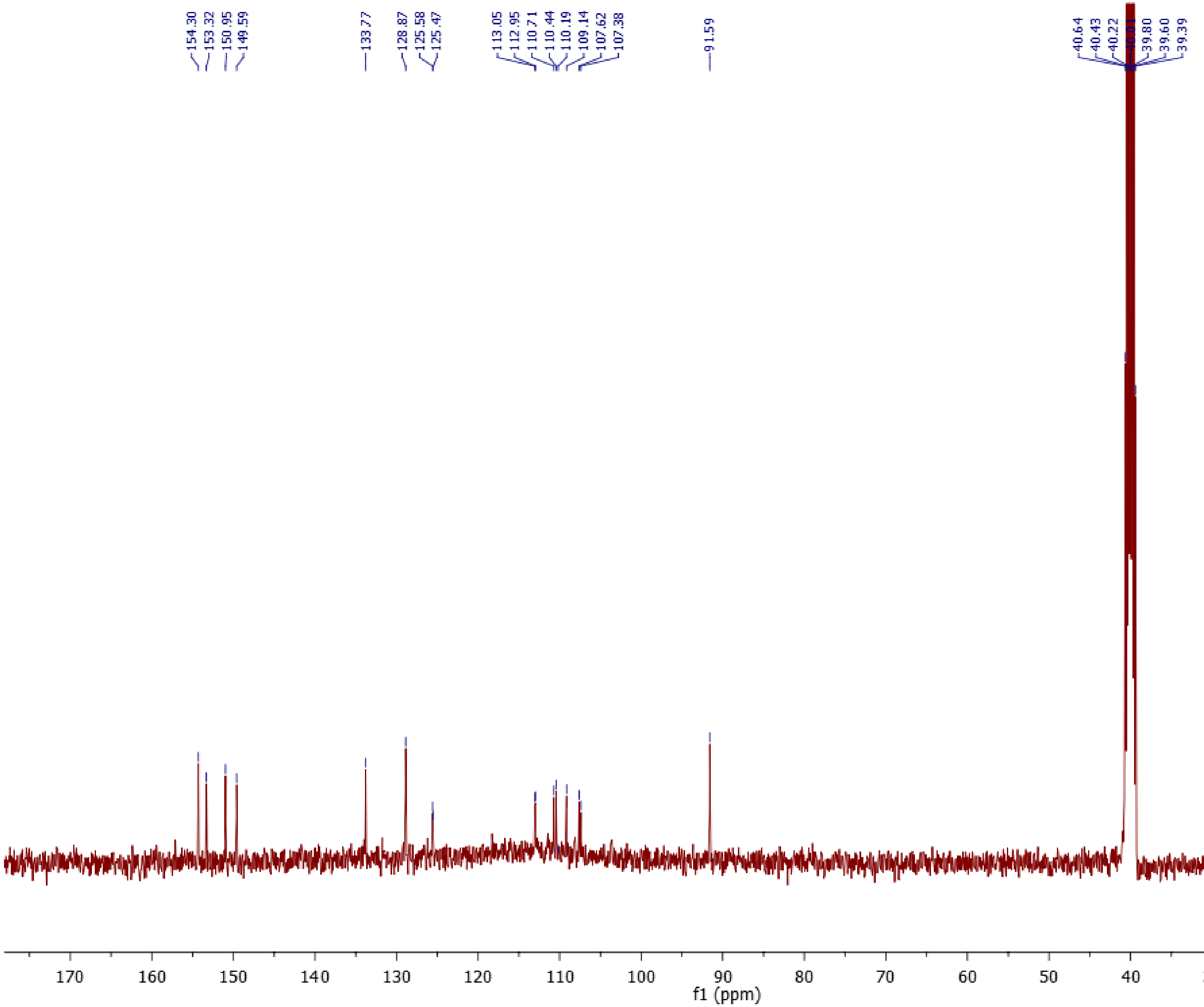
154.30
153.32
150.95
149.59
133.77
128.87
125.58
125.47
113.05
112.95
110.71
110.44
110.19
109.14
107.62
107.38
91.59
40.64
40.43
40.22
40.01
39.80
39.60
39.39
170
160
150
140
130
120
110
100
90
80
70
60
50
40
30
20
10
f1 (ppm)

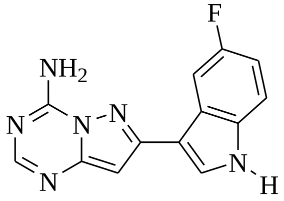
F
NH2
N
N
N
N
N
H

1e ^{1}H NMR

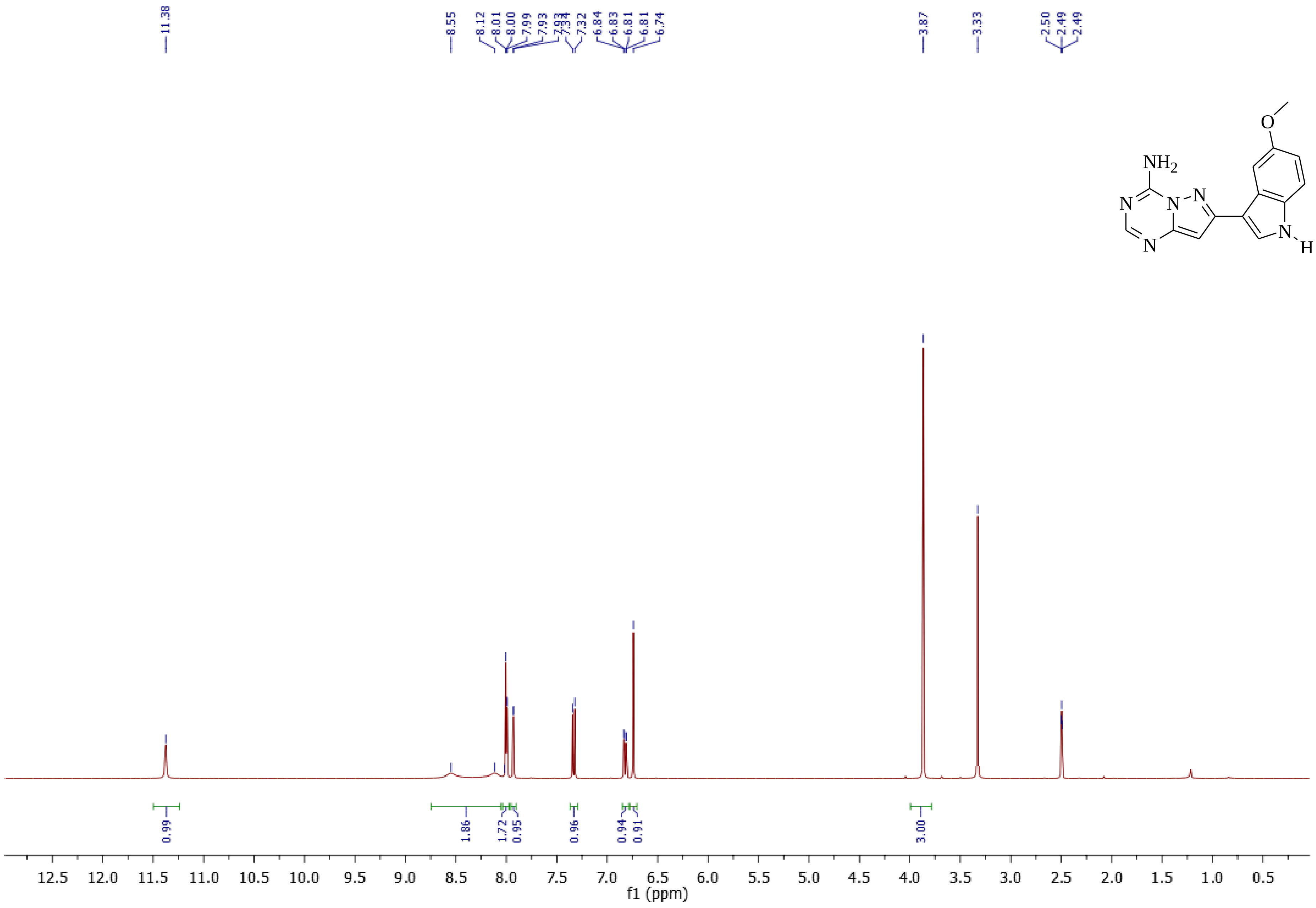
11.38
8.55
8.12
8.01
8.00
7.99
7.93
7.34
7.32
6.84
6.83
6.81
6.81
6.74
3.87
3.33
2.50
2.49
2.49
NH2
N
N
N
N
N
O
N
H
0.99
1.86
1.72
0.95
0.96
0.94
0.91
3.00
12.5 12.0 11.5 11.0 10.5 10.0 9.5 9.0 8.5 8.0 7.5 7.0 6.5 6.0 5.5 5.0 4.5 4.0 3.5 3.0 2.5 2.0 1.5 1.0 0.5
f1 (ppm)

1e ^{13}C NMR

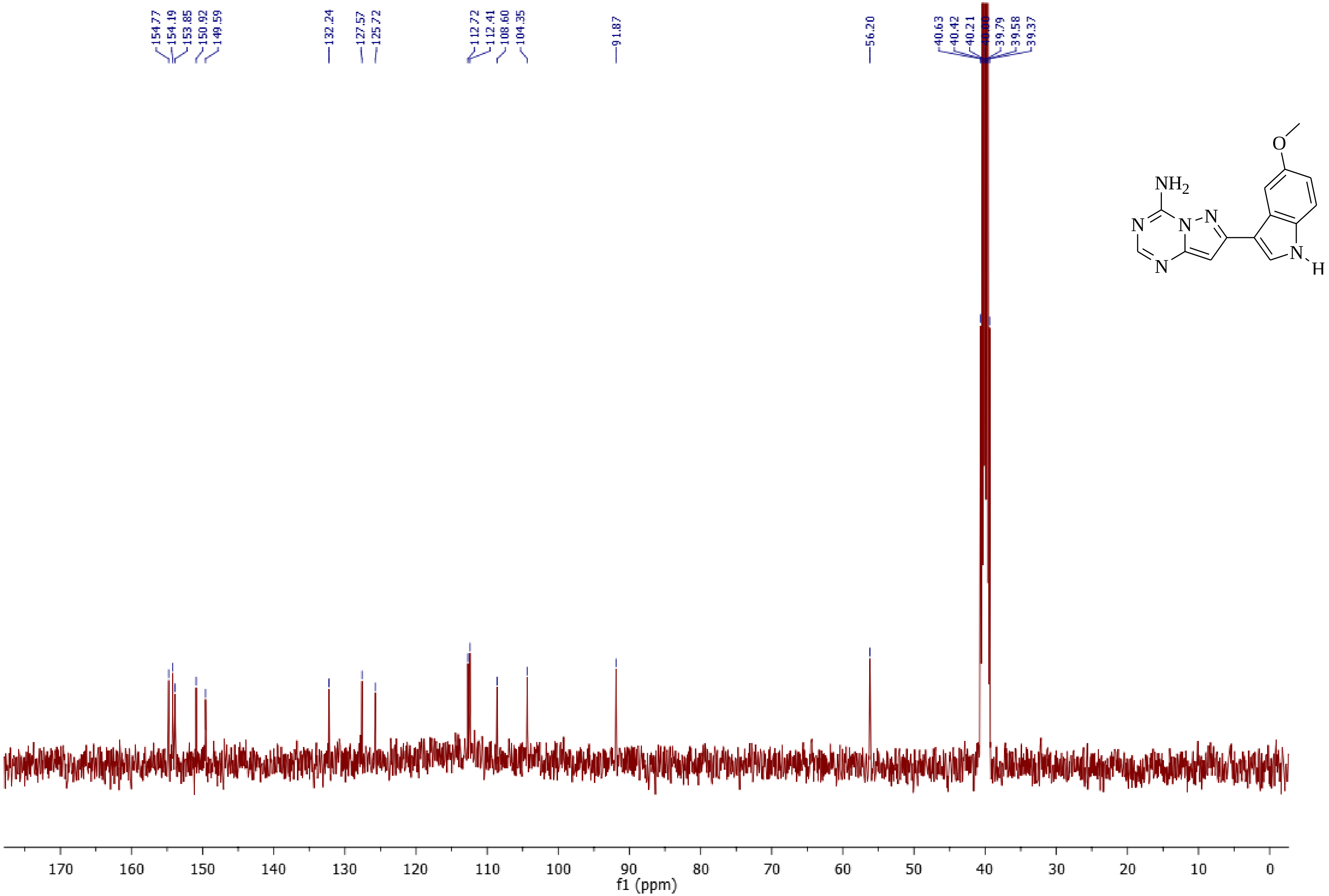
154.77
154.19
153.85
150.92
149.59
132.24
127.57
125.72
112.72
112.41
108.60
104.35
91.87
56.20
40.63
40.42
40.21
40.00
39.79
39.58
39.37
O
NH2
N
N
N
N
N
H
170 160 150 140 130 120 110 100 90 80 70 60 50 40 30 20 10 0
f1 (ppm)

1f ¹H NMR

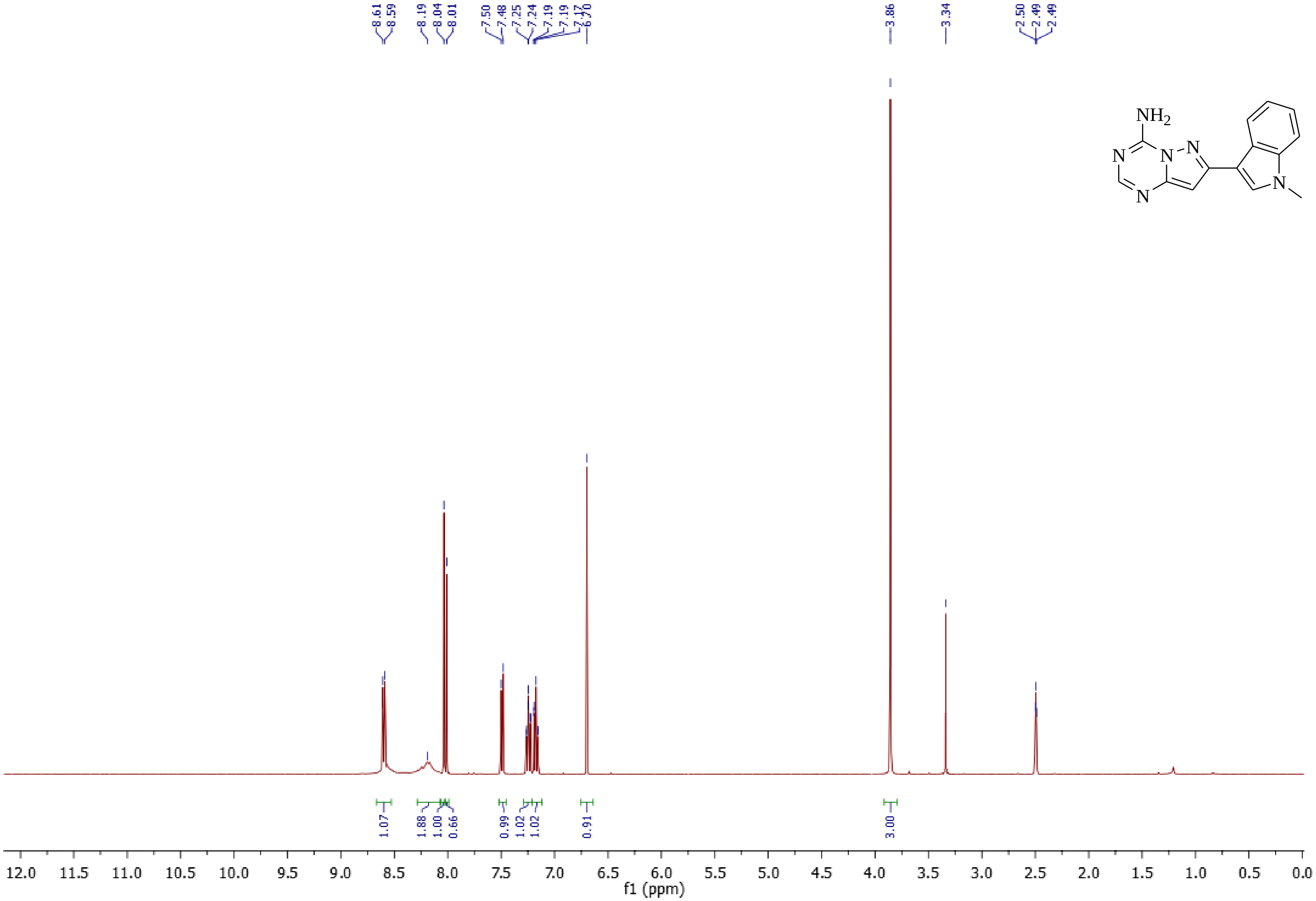
8.61
8.59
8.19
8.04
8.01
7.50
7.48
7.25
7.24
7.19
7.19
7.17
6.70
3.86
3.34
2.50
2.49
2.49
NH2
N
N
N
N
N
1.07
1.88
1.00
0.66
0.99
1.02
1.02
0.91
3.00
12.0
11.5
11.0
10.5
10.0
9.5
9.0
8.5
8.0
7.5
7.0
6.5
6.0
5.5
5.0
4.5
4.0
3.5
3.0
2.5
2.0
1.5
1.0
0.5
0.0
f1 (ppm)

2f ^{13}C NMR

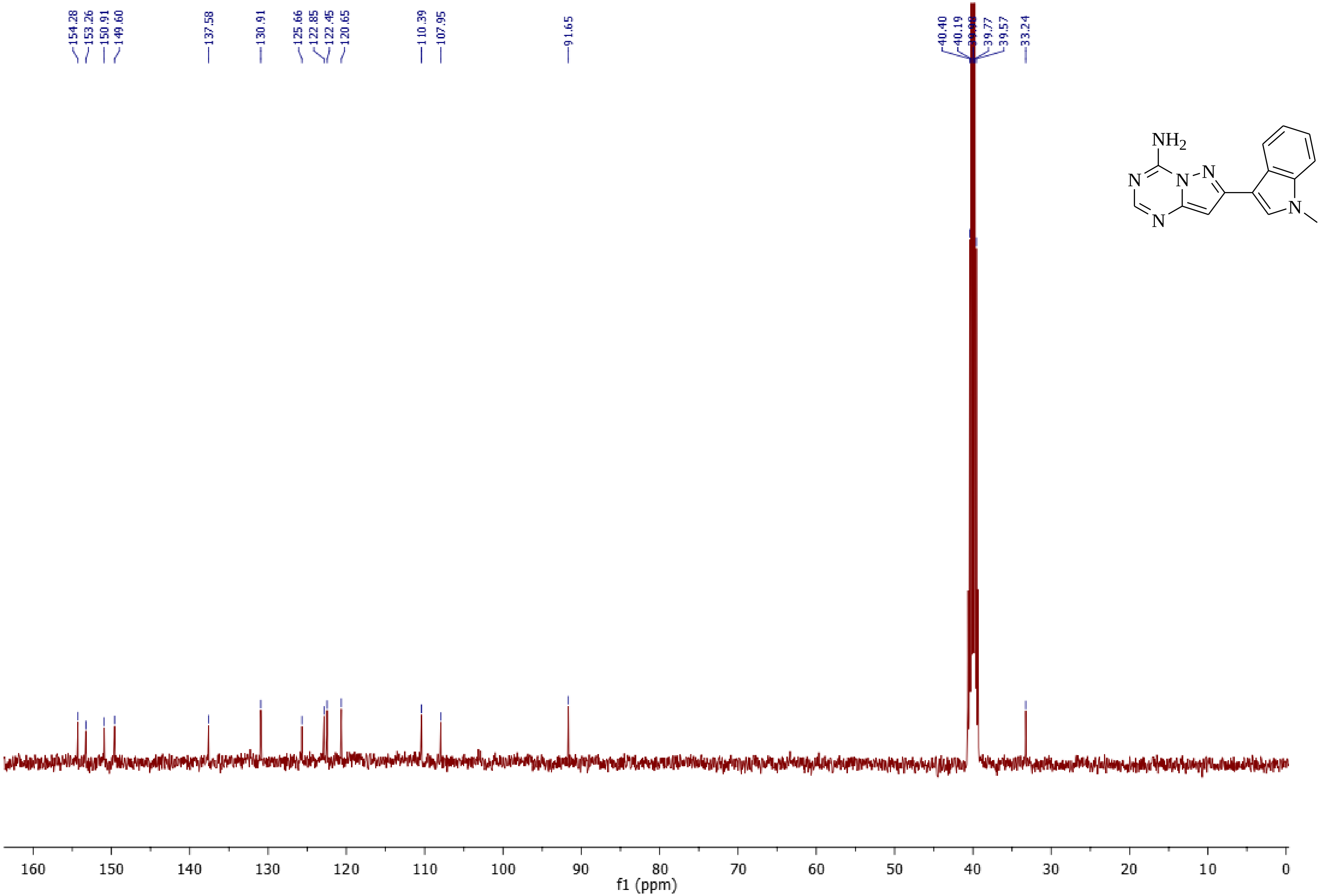

154.28
153.26
150.91
149.60
137.58
130.91
125.66
122.85
122.45
120.65
110.39
107.95
91.65
40.40
40.19
39.77
39.57
33.24
NH2
N
N
N
N
N
160
150
140
130
120
110
100
90
80
70
60
50
40
30
20
10
0
f1 (ppm)

1g ^{1}H NMR

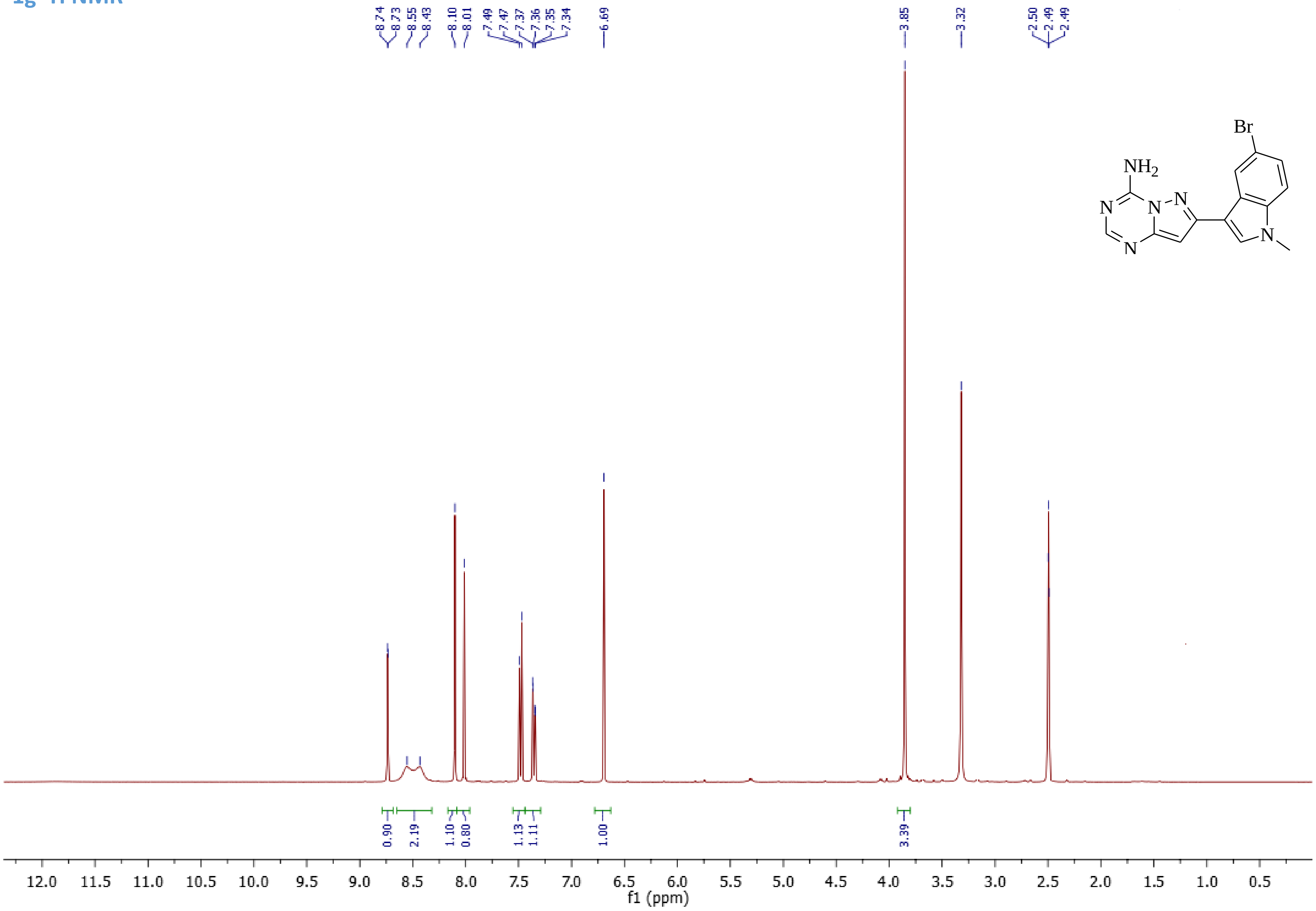
8.74
8.73
8.55
8.43
8.10
8.01
7.49
7.47
7.37
7.36
7.35
7.34
6.69
3.85
3.32
2.50
2.49
2.49
Br
NH2
N
N
N
N
N
0.90
2.19
1.10
0.80
1.13
1.11
1.00
3.39
12.0
11.5
11.0
10.5
10.0
9.5
9.0
8.5
8.0
7.5
7.0
6.5
6.0
5.5
5.0
4.5
4.0
3.5
3.0
2.5
2.0
1.5
1.0
0.5
f1 (ppm)

1g ^{13}C NMR

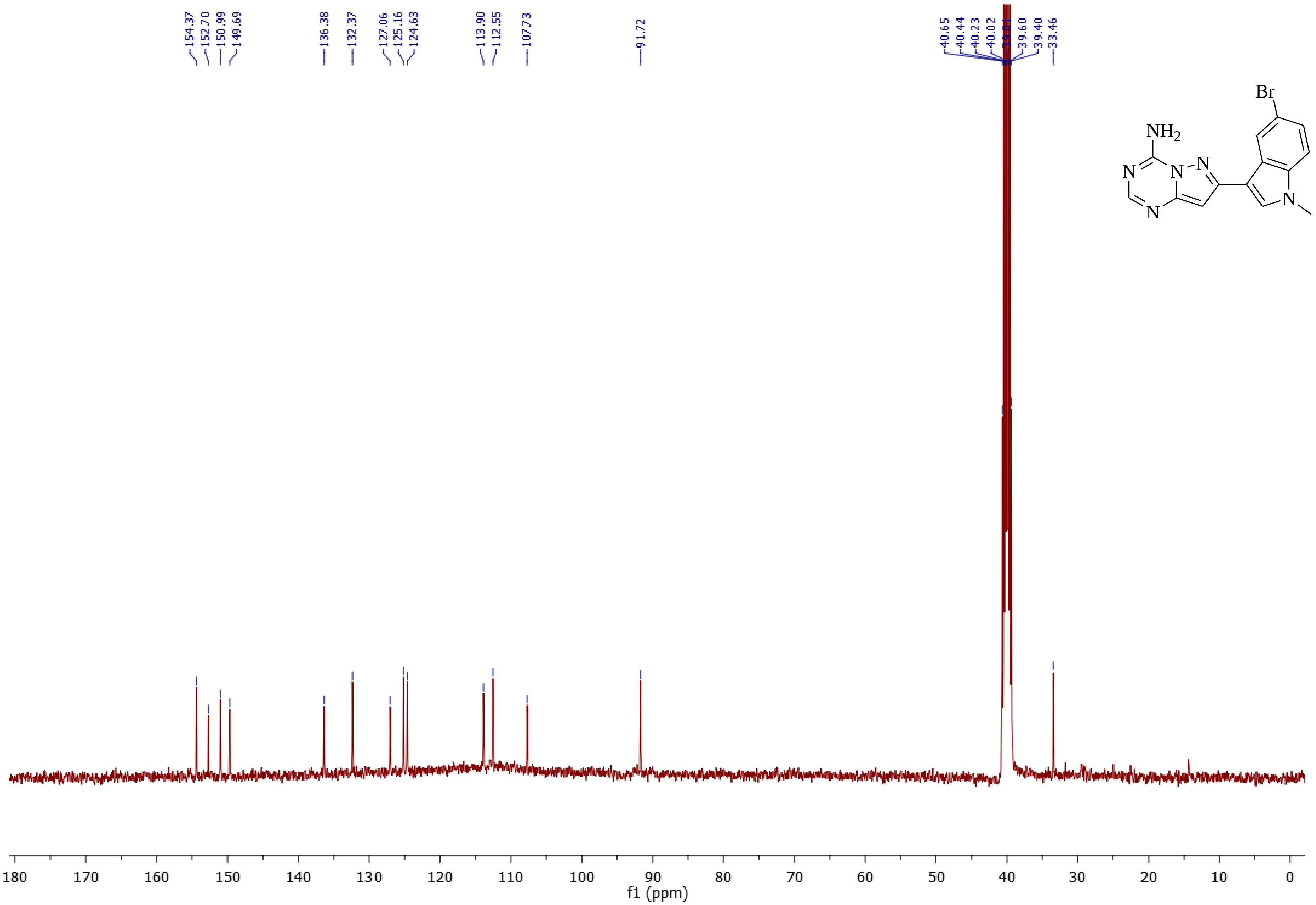
154.37
152.70
150.99
149.69
136.38
132.37
127.06
125.16
124.63
113.90
112.55
107.73
91.72
40.65
40.44
40.23
40.02
39.81
39.60
39.40
33.46
Br
NH2
N
N
N
N
N
N
f1 (ppm)
180
170
160
150
140
130
120
110
100
90
80
70
60
50
40
30
20
10
0

1g HRMS

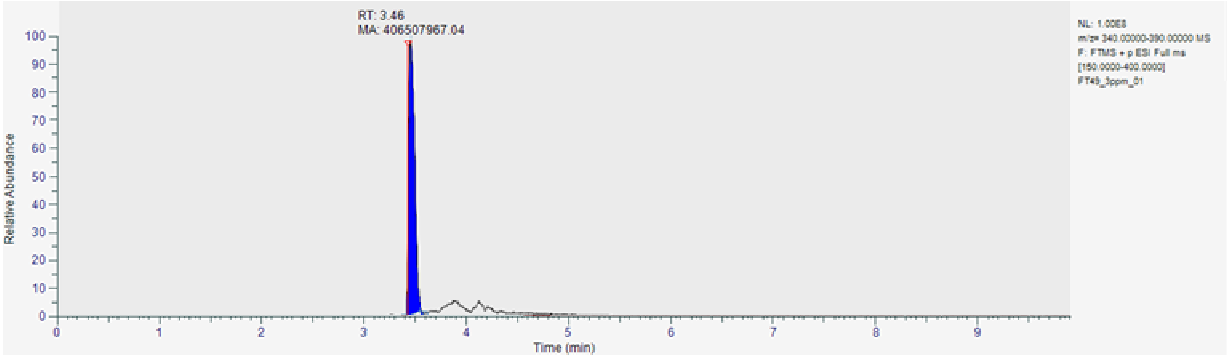
RT: 3.46
MA: 406507967.04
NL: 1.00E8
m/z= 340.00000-390.00000 MS
F: FTMS + p ESI Full ms
[150.0000-400.0000]
FT49_3ppm_01
Relative Abundance
Time (min)

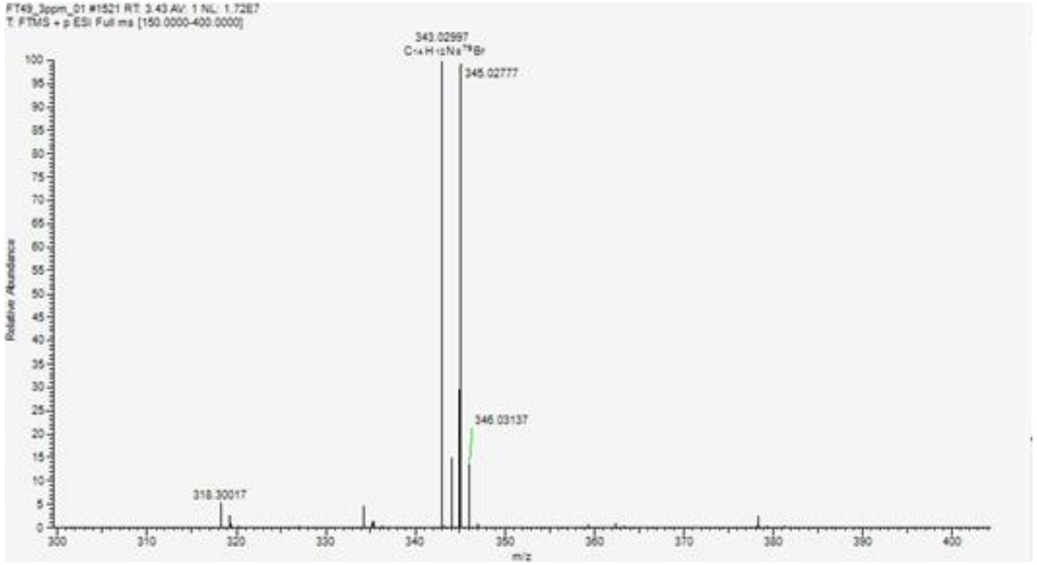
FT49_3ppm_01 #1521 RT: 3.43 AV: 1 NL: 1.72E7
T: FTMS + p ESI Full ms [150.0000-400.0000]
343.02997
C14 H12 N8 79Br
345.02777
346.03137
318.30017
Relative Abundance
m/z

1h ¹H NMR

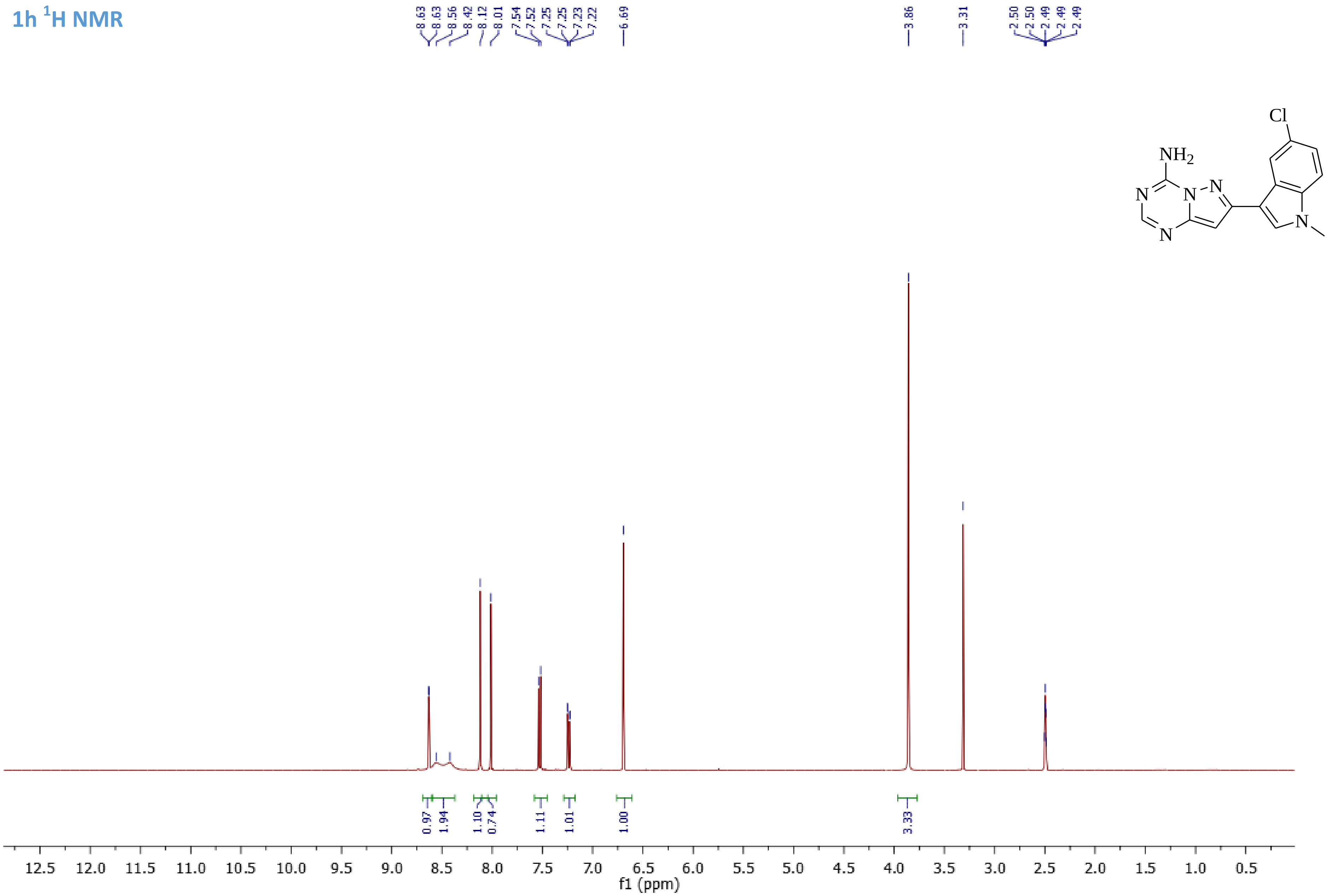
8.63
8.63
8.56
8.42
8.12
8.01
7.54
7.52
7.25
7.25
7.23
7.22
6.69
3.86
3.31
2.50
2.50
2.49
2.49
2.49
Cl
NH2
N
N
N
N
N
N
0.97
1.94
1.10
0.74
1.11
1.01
1.00
3.33
12.5
12.0
11.5
11.0
10.5
10.0
9.5
9.0
8.5
8.0
7.5
7.0
6.5
6.0
5.5
5.0
4.5
4.0
3.5
3.0
2.5
2.0
1.5
1.0
0.5
f1 (ppm)

1h ^{13}C NMR

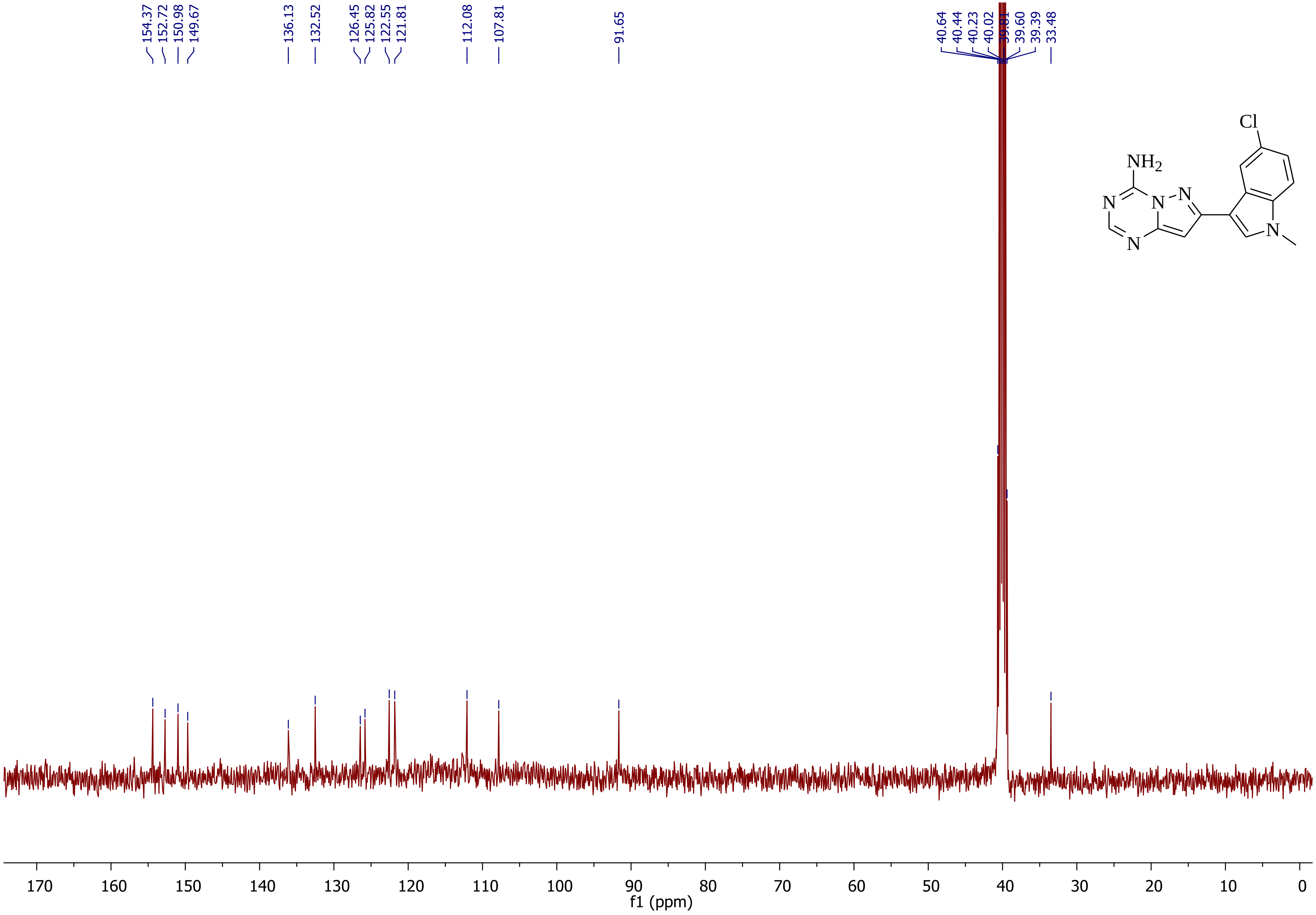
154.37
152.72
150.98
149.67
136.13
132.52
126.45
125.82
122.55
121.81
112.08
107.81
91.65
40.64
40.44
40.23
40.02
39.81
39.60
39.39
33.48
170
160
150
140
130
120
110
100
90
80
70
60
50
40
30
20
10
0
f1 (ppm)
Cl
NH2
N
N
N
N
N

1i ^{1}H NMR

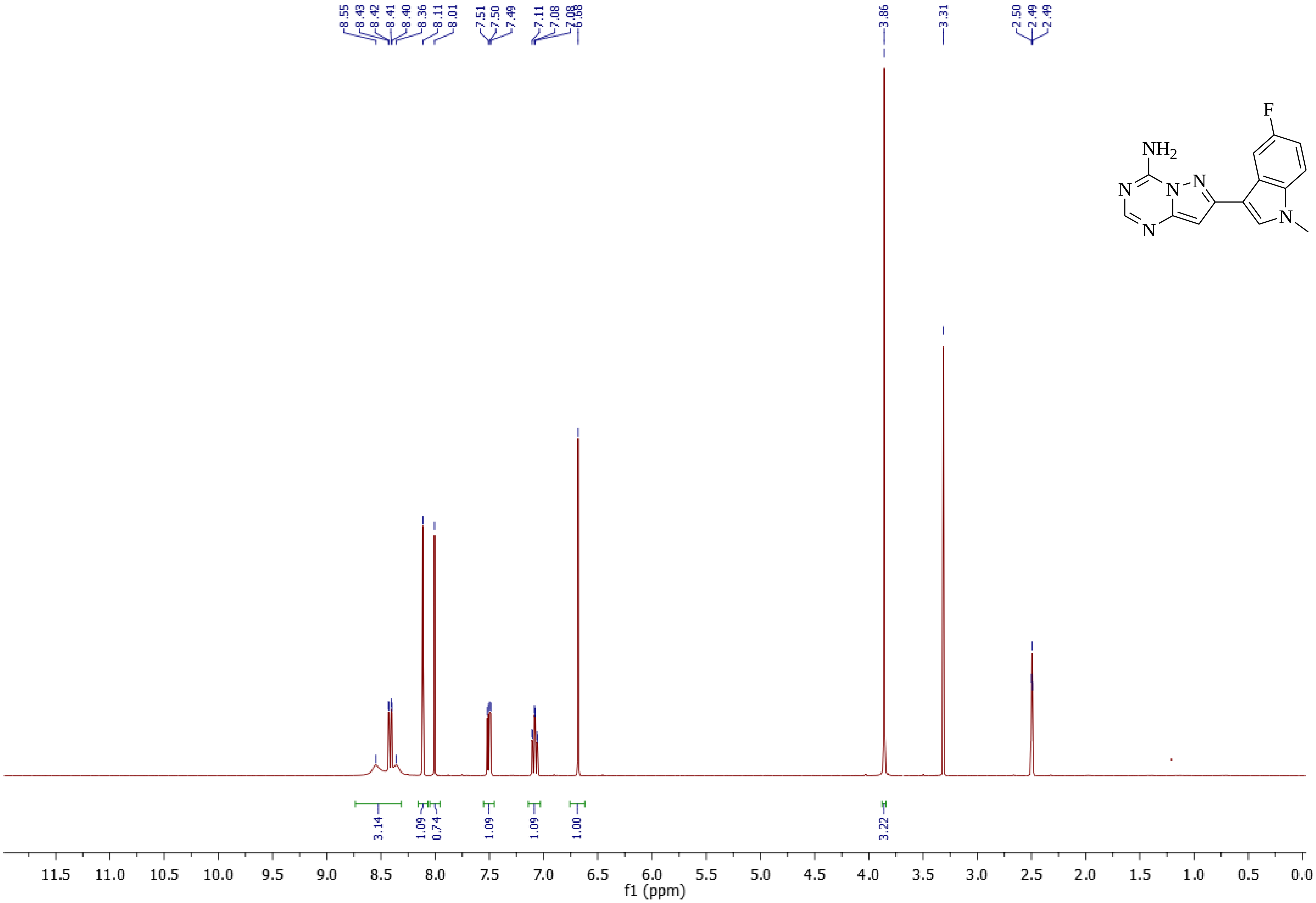
8.55 8.43 8.42 8.41 8.40 8.36 8.11 8.01 7.51 7.50 7.49 7.11 7.08 7.08 6.68
3.86
3.31
2.50 2.49 2.49
3.14 1.09 0.74 1.09 1.09 1.00 3.22
f1 (ppm)
F
NH2
N
N
N
N
N

1i ^{13}C NMR

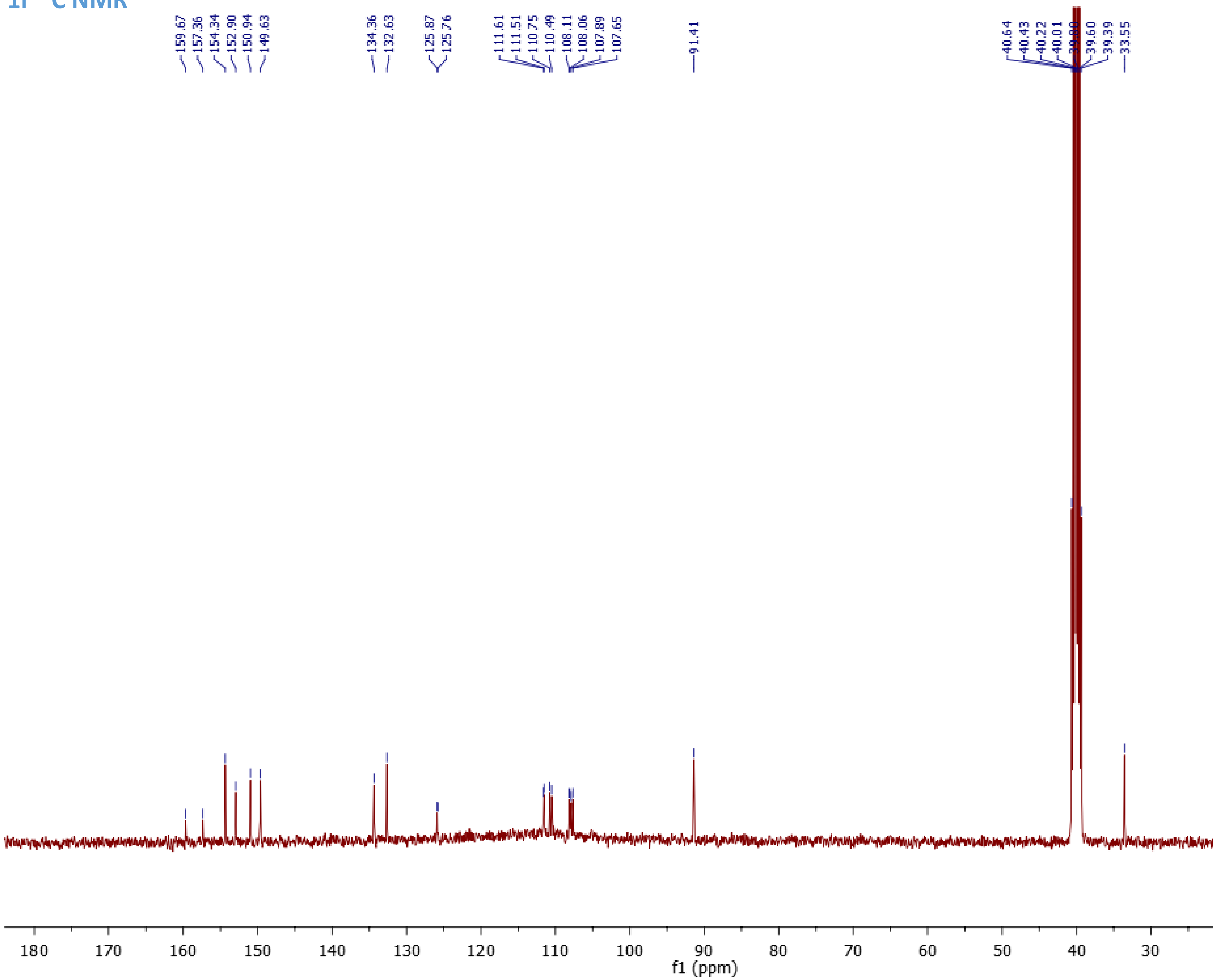
159.67
157.36
154.34
152.90
150.94
149.63
134.36
132.63
125.87
125.76
111.61
111.51
110.75
110.49
108.11
108.06
107.89
107.65
91.41
40.64
40.43
40.22
40.01
39.60
39.39
33.55
180
170
160
150
140
130
120
110
100
90
80
70
60
50
40
30
20
10
0
f1 (ppm)

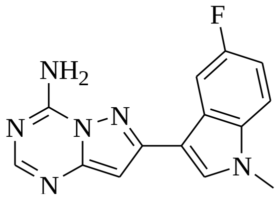
F
NH2
N
N
N
N
N

1j ^{1}H NMR

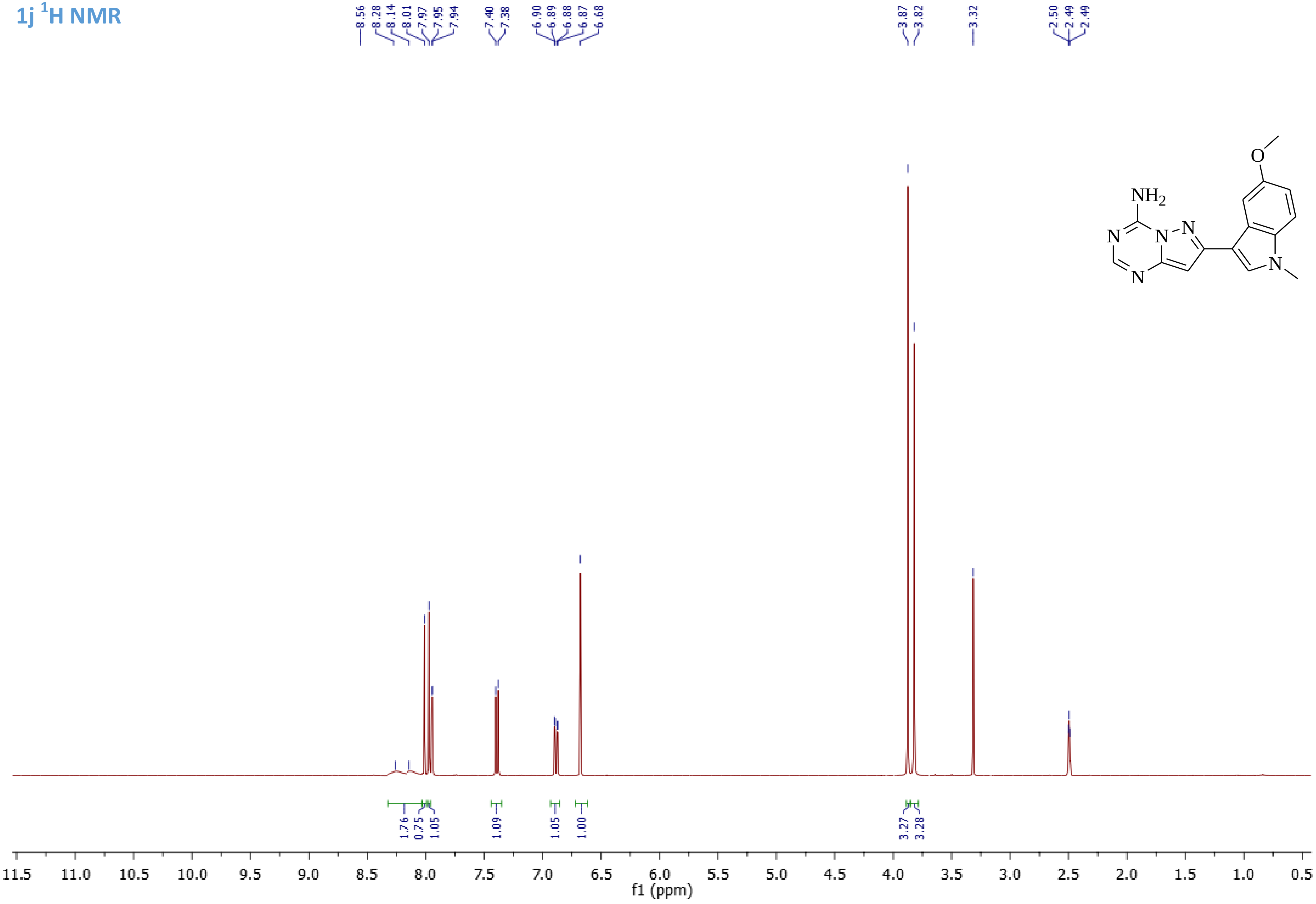
8.56
8.28
8.14
8.01
7.97
7.95
7.94
7.40
7.38
6.90
6.89
6.88
6.87
6.68
3.87
3.82
3.32
2.50
2.49
2.49
1.76
0.75
1.05
1.09
1.05
1.00
3.27
3.28
NH2
N
N
N
N
N
O
11.5
11.0
10.5
10.0
9.5
9.0
8.5
8.0
7.5
7.0
6.5
6.0
5.5
5.0
4.5
4.0
3.5
3.0
2.5
2.0
1.5
1.0
0.5
f1 (ppm)

1j ^{13}C NMR

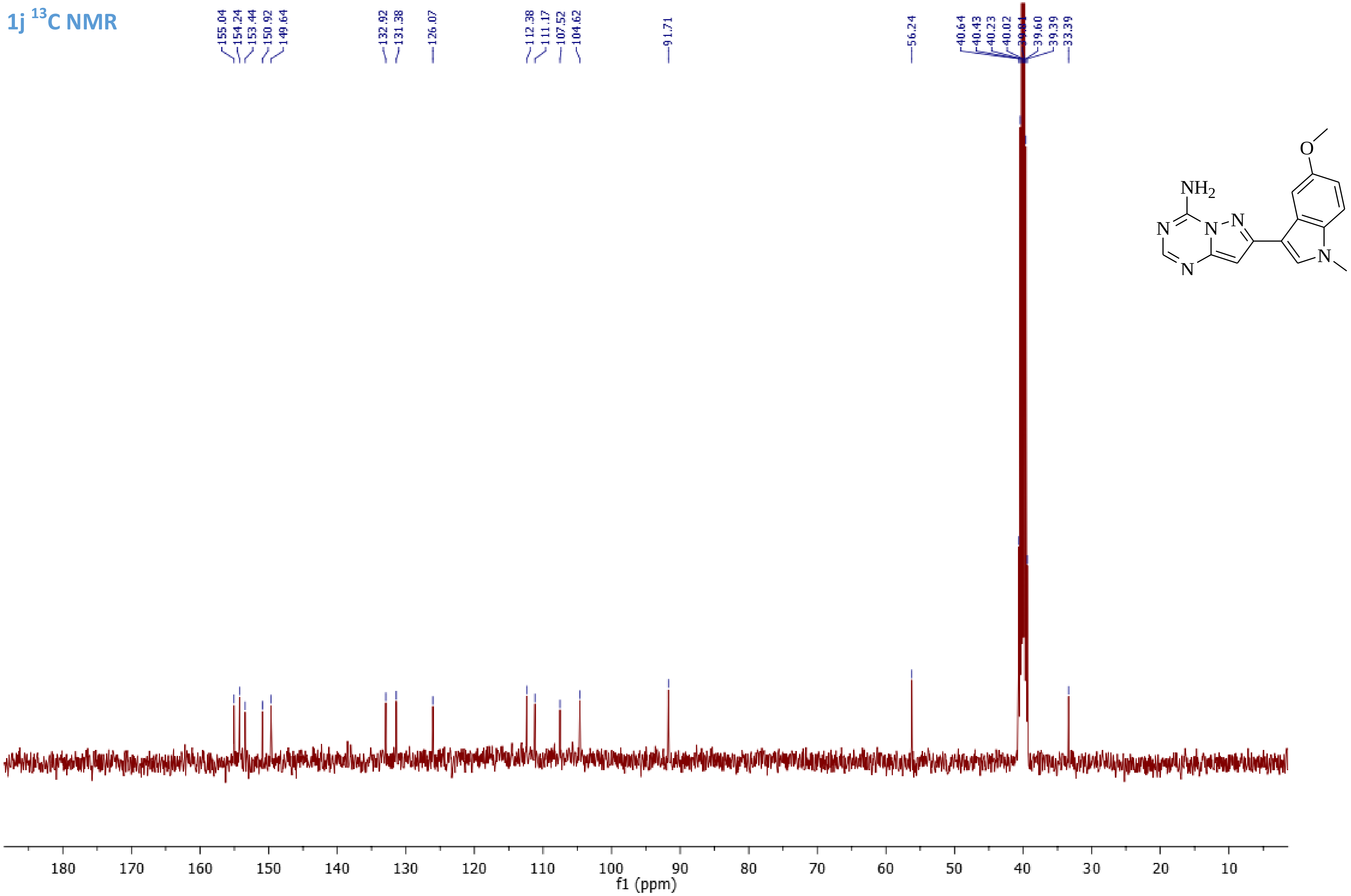
155.04
154.24
153.44
150.92
149.64
132.92
131.38
126.07
112.38
111.17
107.52
104.62
91.71
56.24
40.64
40.43
40.23
40.02
39.60
39.39
33.39
180 170 160 150 140 130 120 110 100 90 80 70 60 50 40 30 20 10
f1 (ppm)
O
NH2
N
N
N
N
N

2a ¹H NMR

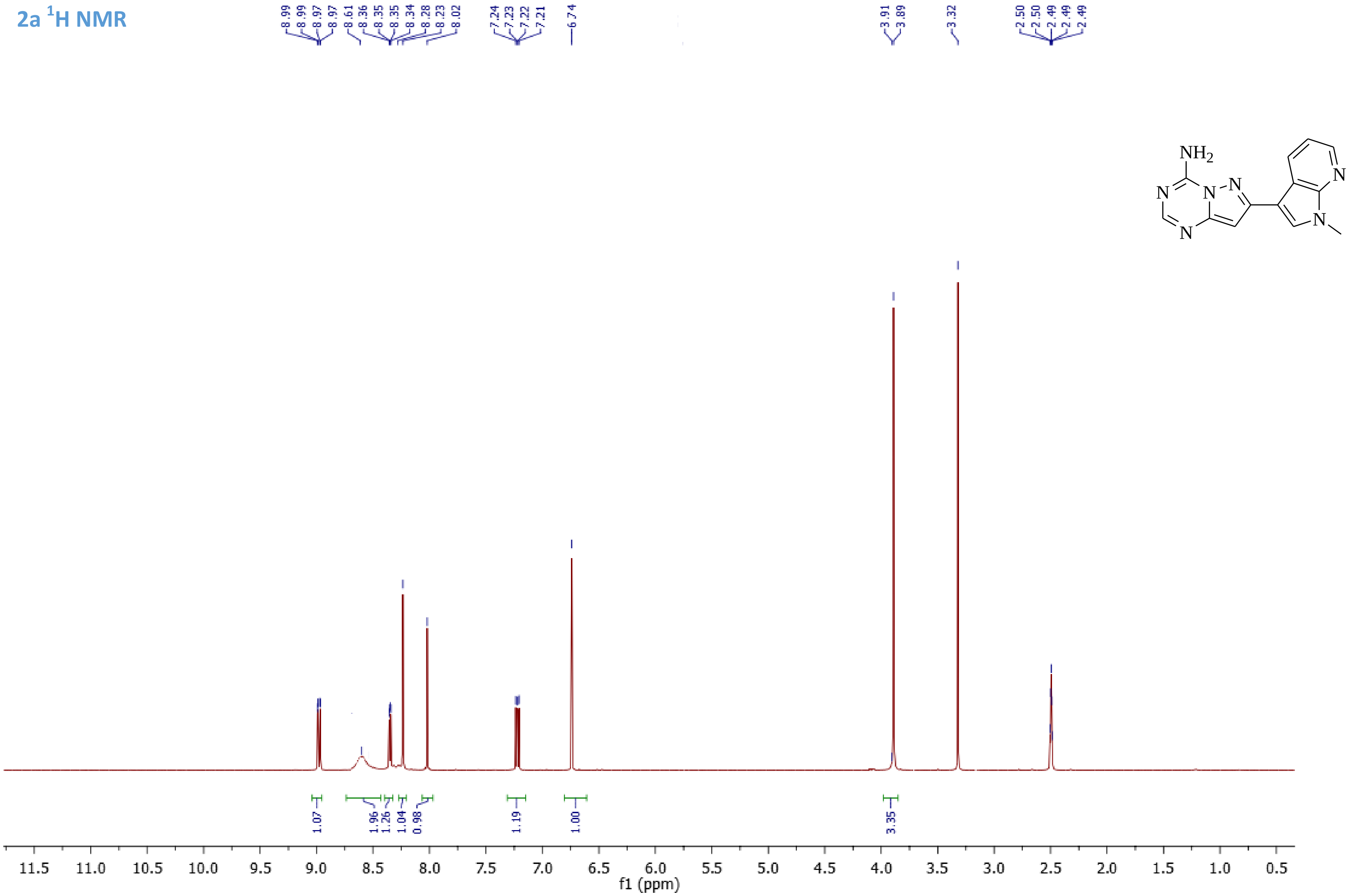

8.99
8.99
8.97
8.97
8.61
8.36
8.35
8.35
8.34
8.28
8.23
8.02
7.24
7.23
7.22
7.21
6.74
3.91
3.89
3.32
2.50
2.50
2.49
2.49
2.49
NH2
N
N
N
N
N
N
1.07
1.96
1.26
1.04
0.98
1.19
1.00
3.35
11.5
11.0
10.5
10.0
9.5
9.0
8.5
8.0
7.5
7.0
6.5
6.0
5.5
5.0
4.5
4.0
3.5
3.0
2.5
2.0
1.5
1.0
0.5
f1 (ppm)

2a ^{13}C NMR

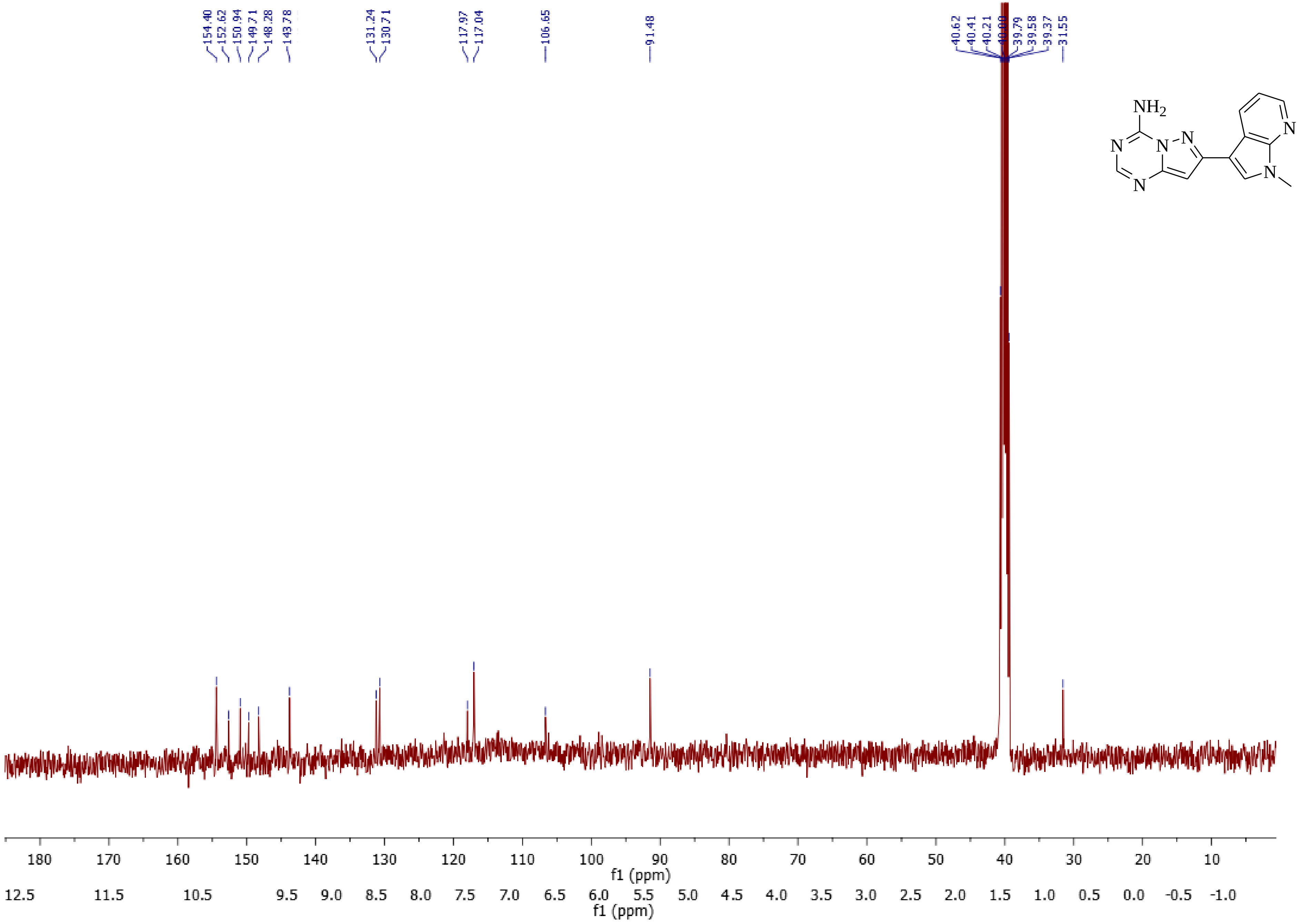
154.40
152.62
150.94
149.71
148.28
143.78
131.24
130.71
117.97
117.04
106.65
91.48
40.62
40.41
40.21
40.00
39.79
39.58
39.37
31.55
NH_2
N
N
N
N
N
N
180 170 160 150 140 130 120 110 100 90 80 70 60 50 40 30 20 10
f1 (ppm)
12.5 11.5 10.5 9.5 9.0 8.5 8.0 7.5 7.0 6.5 6.0 5.5 5.0 4.5 4.0 3.5 3.0 2.5 2.0 1.5 1.0 0.5 0.0 -0.5 -1.0
f1 (ppm)

2b ^{1}H NMR

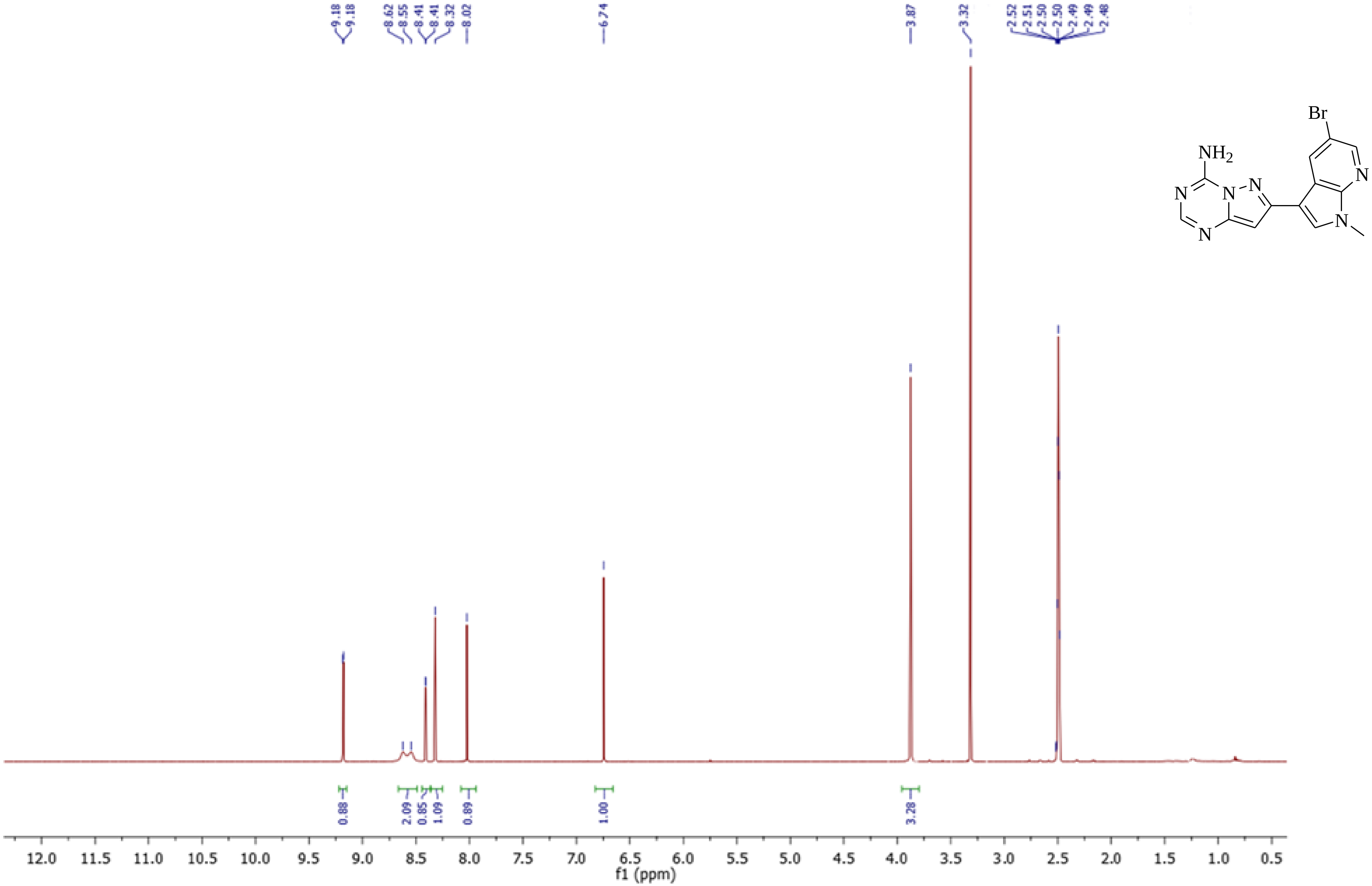
9.18
9.18
8.62
8.55
8.41
8.41
8.32
8.02
6.74
3.87
3.32
2.52
2.51
2.50
2.50
2.49
2.49
2.48
0.88
2.09
0.85
1.09
0.89
1.00
3.28
12.0 11.5 11.0 10.5 10.0 9.5 9.0 8.5 8.0 7.5 7.0 6.5 6.0 5.5 5.0 4.5 4.0 3.5 3.0 2.5 2.0 1.5 1.0 0.5
f1 (ppm)
Br
NH2
N
N
N
N
N
N
N

2b ^{13}C NMR

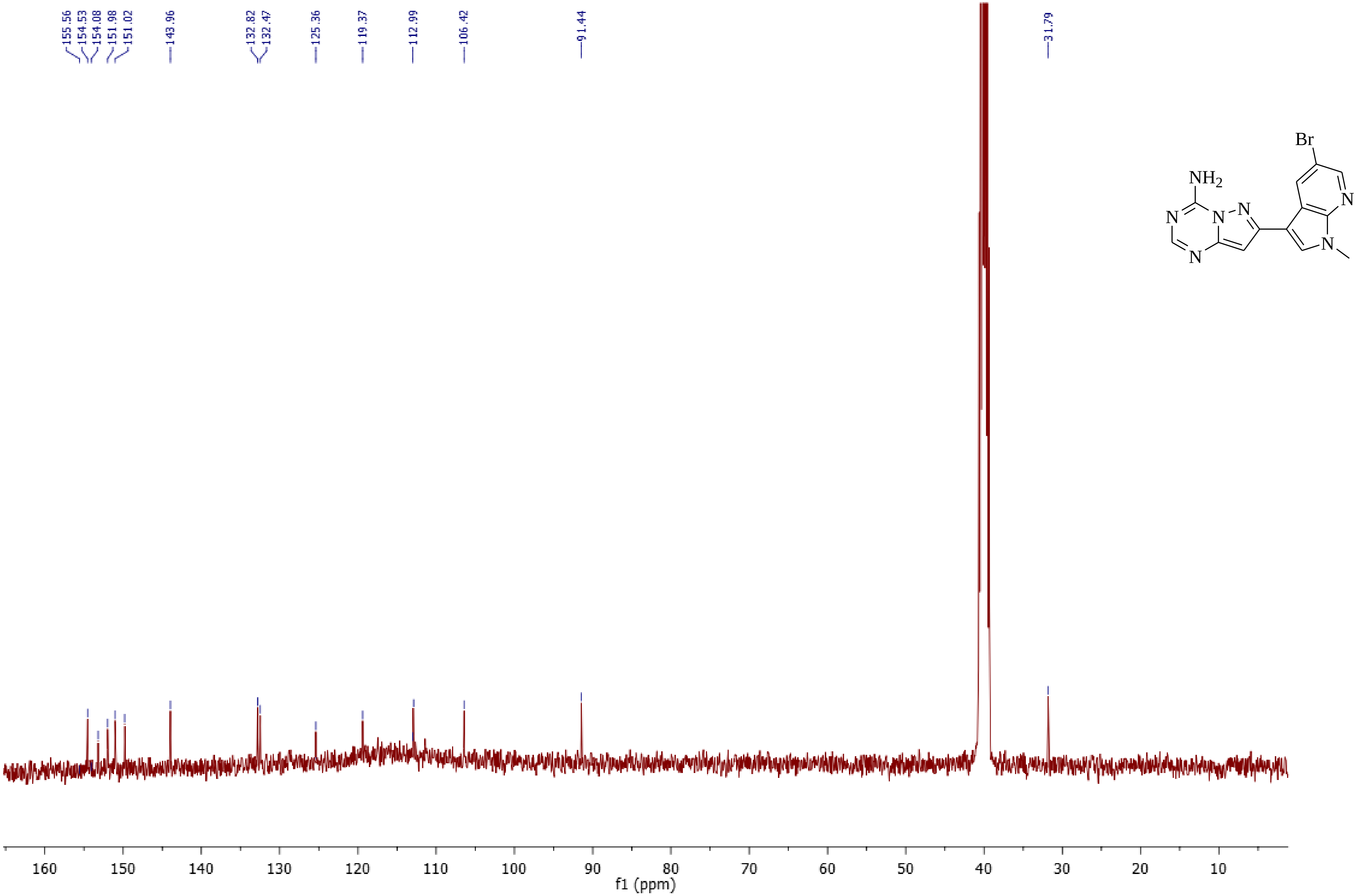
155.56
154.53
154.08
151.98
151.02
143.96
132.82
132.47
125.36
119.37
112.99
106.42
91.44
31.79
160
150
140
130
120
110
100
90
80
70
60
50
40
30
20
10
f1 (ppm)
Br
NH2
N
N
N
N
N
N
N

3a ^{1}H NMR

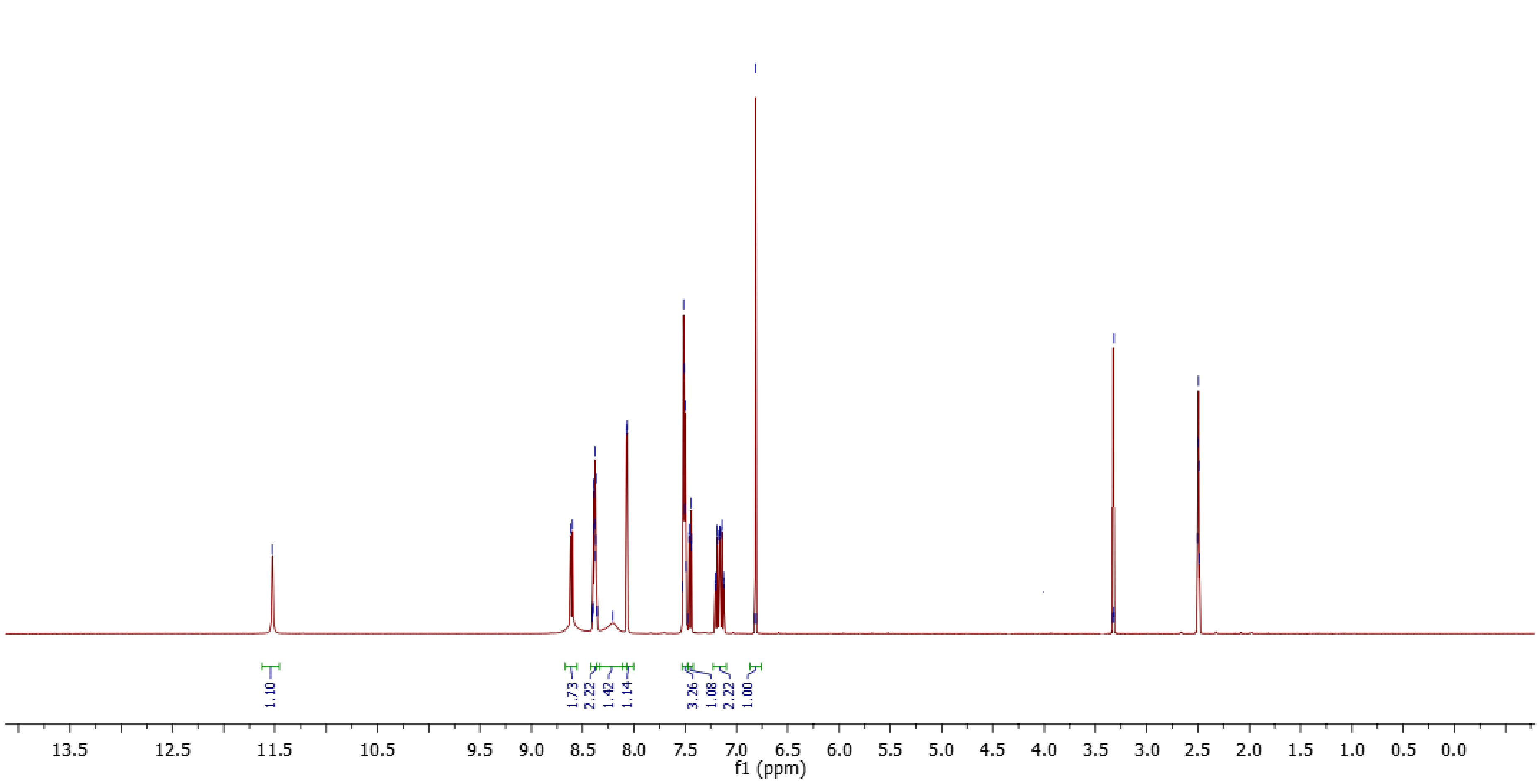
11.53
8.62 8.60 8.41 8.40 8.40 8.39 8.39 8.39 8.38 8.37 8.37 8.37 8.36 8.36 8.21 8.07 8.07
7.52 7.51 7.50 7.50 7.46 7.44 7.19 7.16 7.14 6.81
3.33 3.33 3.32 3.32
2.50 2.50 2.49 2.49
1.10 1.73 2.22 1.42 1.14 3.26 1.08 2.22 1.00
13.5 12.5 11.5 10.5 9.5 9.0 8.5 8.0 7.5 7.0 6.5 6.0 5.5 5.0 4.5 4.0 3.5 3.0 2.5 2.0 1.5 1.0 0.5 0.0
f1 (ppm)

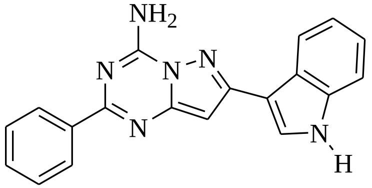
NH2
N
N
N
N
N
H

3a ^{13}C NMR

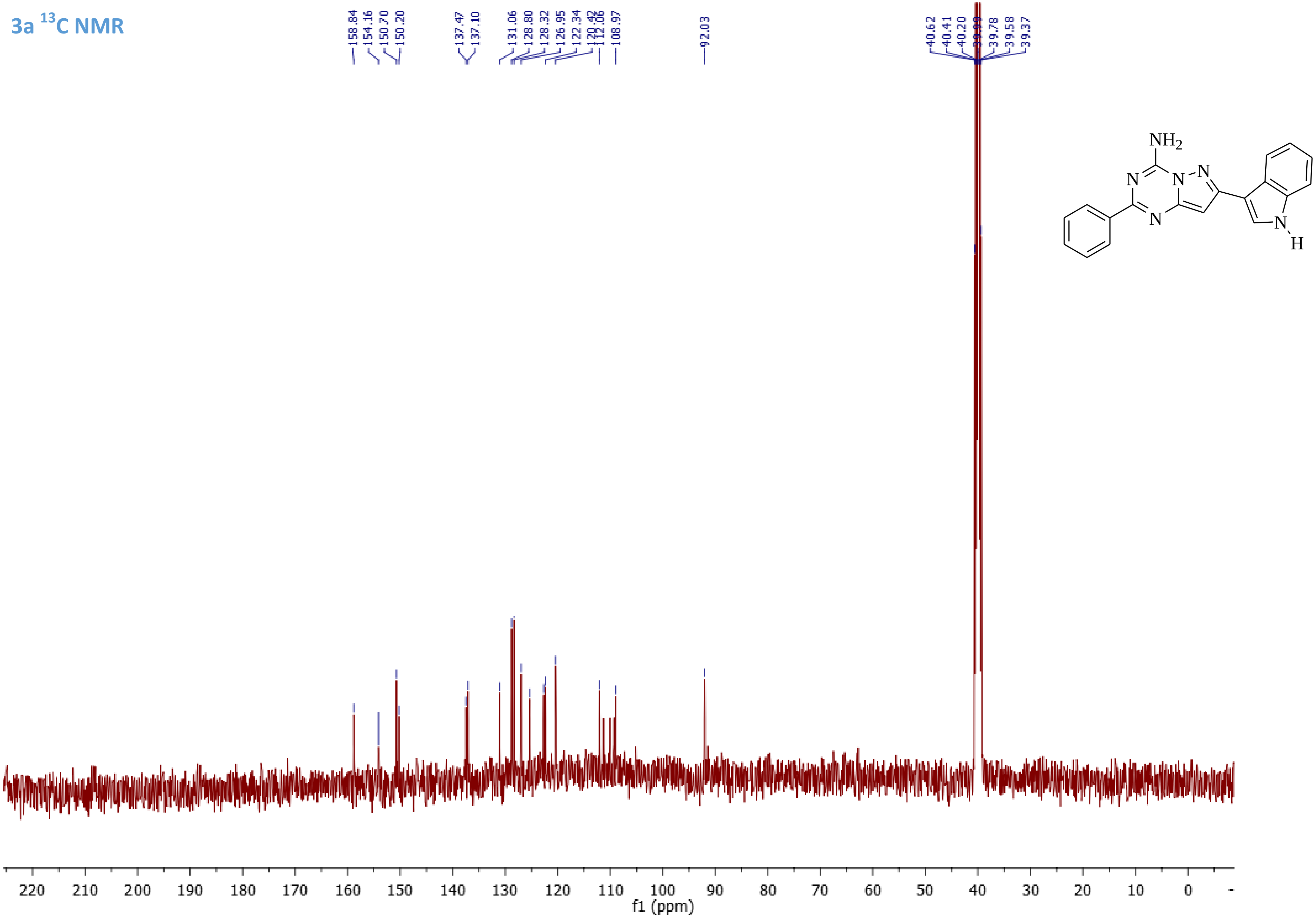
158.84
154.16
150.70
150.20
137.47
137.10
131.06
128.80
128.32
126.95
122.34
120.42
112.06
108.97
92.03
40.62
40.41
40.20
39.78
39.58
39.37
NH2
N
N
N
N
N
H
220 210 200 190 180 170 160 150 140 130 120 110 100 90 80 70 60 50 40 30 20 10 0
f1 (ppm)

3b ^{1}H NMR

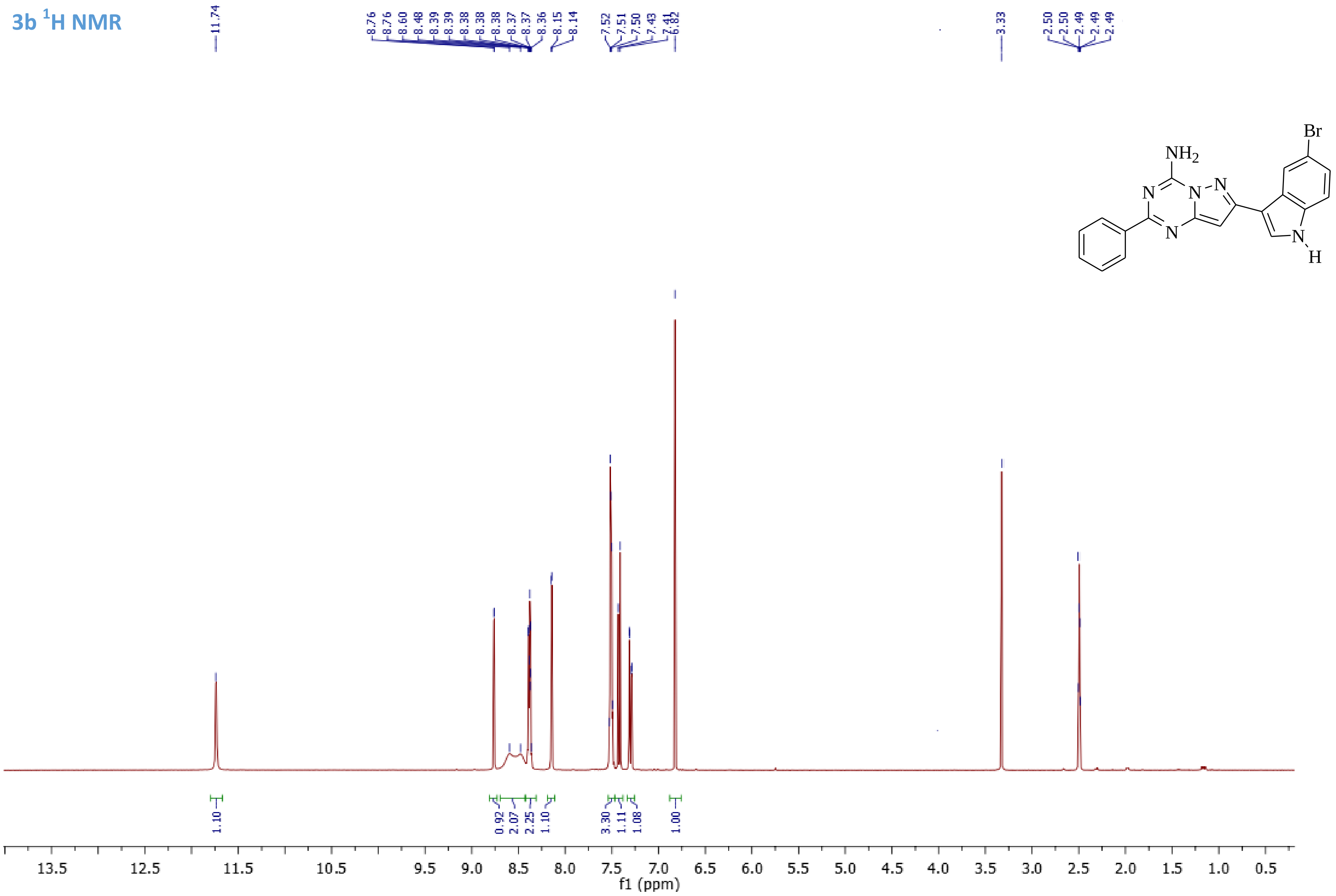
11.74
8.76
8.76
8.60
8.48
8.39
8.39
8.38
8.38
8.38
8.37
8.37
8.36
8.15
8.14
7.52
7.51
7.50
7.43
7.41
6.82
3.33
2.50
2.50
2.49
2.49
2.49
NH2
Br
N
N
N
N
N
H
1.10
0.92
2.07
2.25
1.10
3.30
1.11
1.08
1.00
13.5
12.5
11.5
10.5
9.5
9.0
8.5
8.0
7.5
7.0
6.5
6.0
5.5
5.0
4.5
4.0
3.5
3.0
2.5
2.0
1.5
1.0
0.5
f1 (ppm)

3b ^{13}C NMR

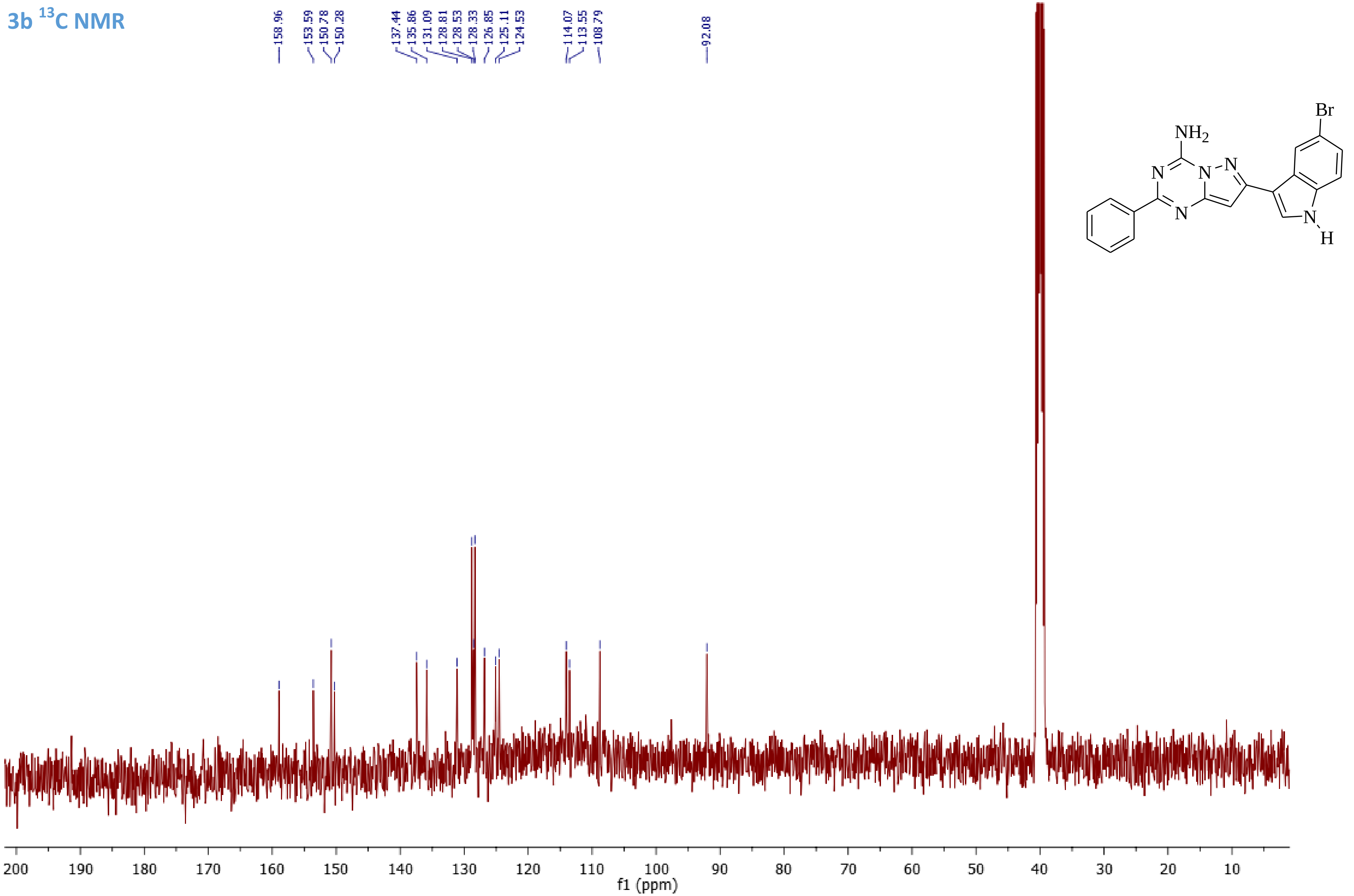
158.96
153.59
150.78
150.28
137.44
135.86
131.09
128.81
128.53
128.33
126.85
125.11
124.53
114.07
113.55
108.79
92.08
NH2
N
N
N
N
N
H
Br
200
190
180
170
160
150
140
130
120
110
100
90
80
70
60
50
40
30
20
10
f1 (ppm)

3c ¹H NMR

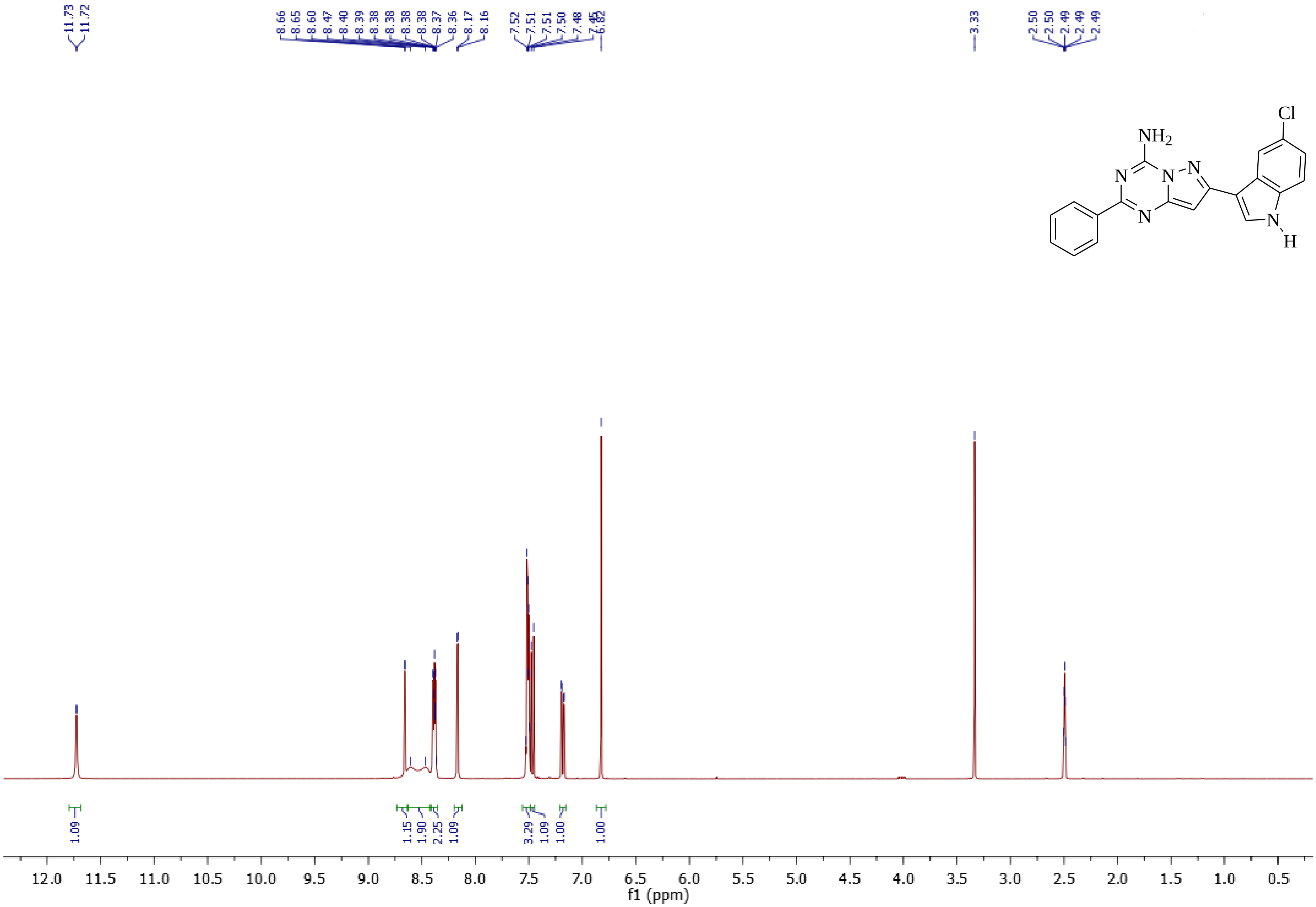
11.73
11.72
8.66
8.65
8.60
8.47
8.40
8.39
8.38
8.38
8.38
8.38
8.37
8.36
8.17
8.16
7.52
7.51
7.51
7.50
7.48
7.45
6.82
3.33
2.50
2.50
2.49
2.49
2.49
Cl
NH2
N
N
N
N
N
N
H
1.09
1.15
1.90
2.25
1.09
3.29
1.09
1.00
1.00
12.0
11.5
11.0
10.5
10.0
9.5
9.0
8.5
8.0
7.5
7.0
6.5
6.0
5.5
5.0
4.5
4.0
3.5
3.0
2.5
2.0
1.5
1.0
0.5
f1 (ppm)

3c 13C NMR

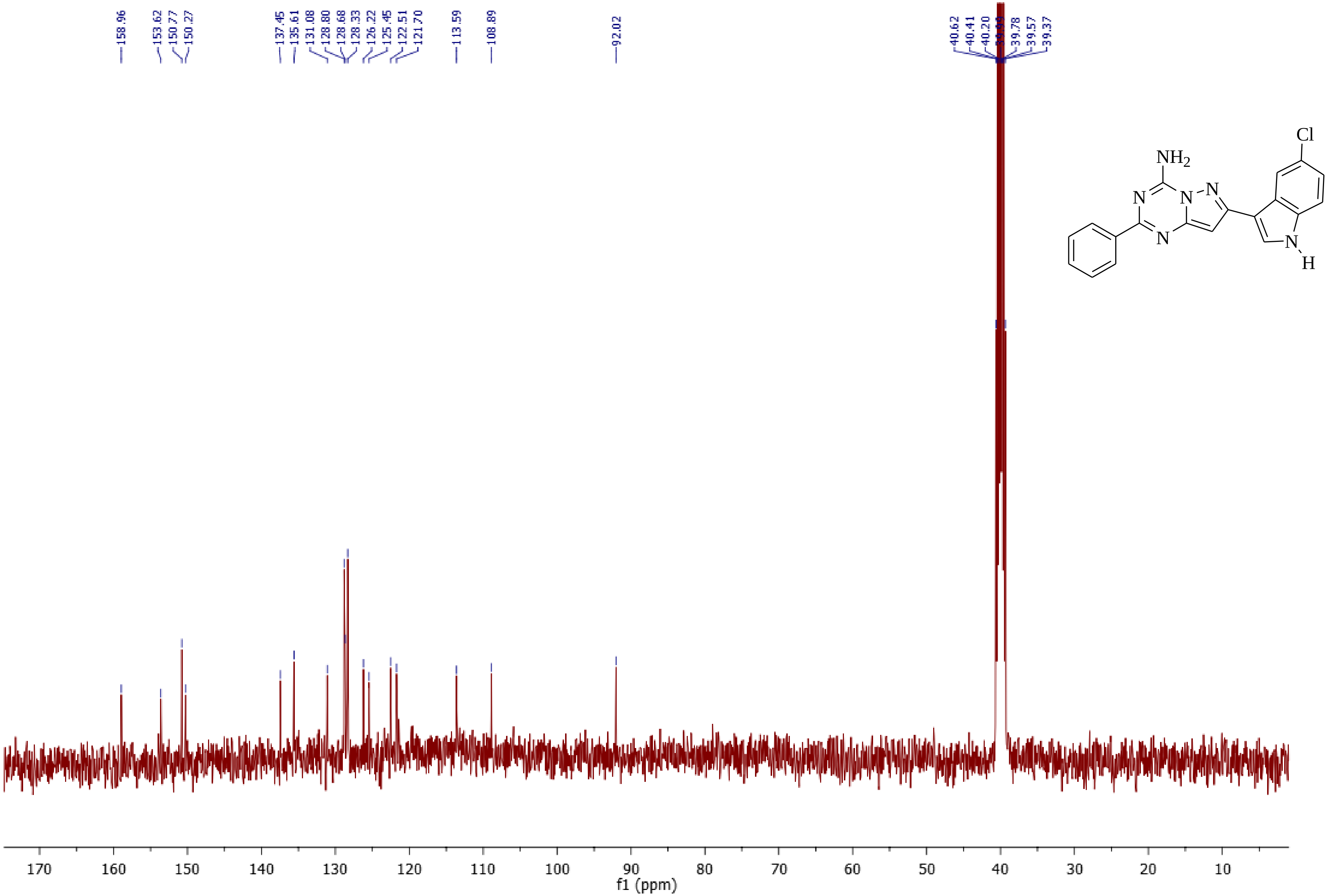
158.96
153.62
150.77
150.27
137.45
135.61
131.08
128.80
128.68
128.33
126.22
125.45
122.51
121.70
113.59
108.89
92.02
40.62
40.41
40.20
39.78
39.57
39.37
NH2
N
N
N
N
N
Cl
N
H
170
160
150
140
130
120
110
100
90
80
70
60
50
40
30
20
10
f1 (ppm)

3d ^{1}H NMR

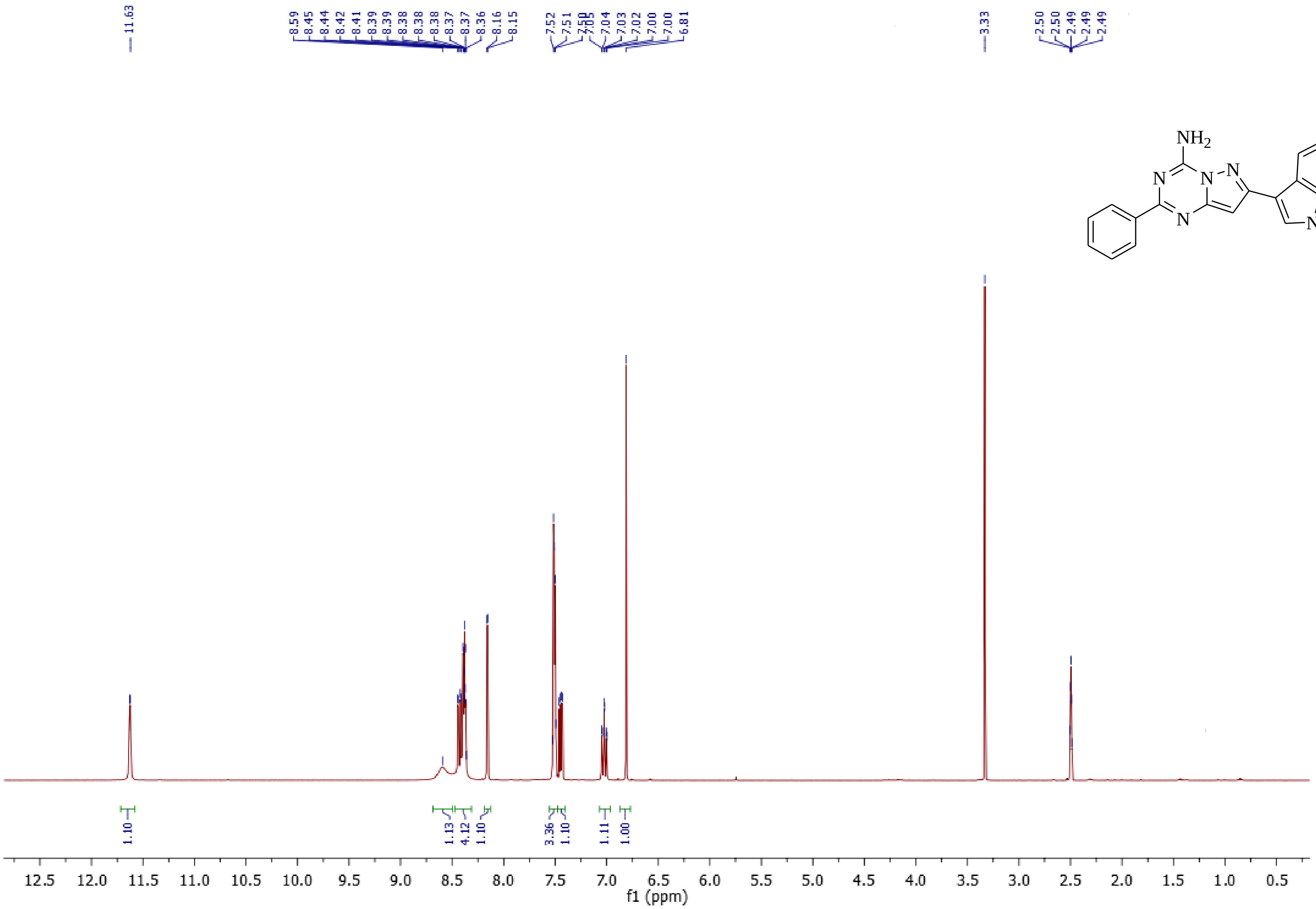
11.63
8.59
8.45
8.44
8.42
8.41
8.39
8.39
8.38
8.38
8.38
8.37
8.37
8.36
8.16
8.15
7.52
7.51
7.50
7.05
7.04
7.03
7.02
7.00
7.00
6.81
3.33
2.50
2.50
2.49
2.49
2.49
NH2
N
N
N
N
F
N
H
1.10
1.13
4.12
1.10
3.36
1.10
1.11
1.00
12.5
12.0
11.5
11.0
10.5
10.0
9.5
9.0
8.5
8.0
7.5
7.0
6.5
6.0
5.5
5.0
4.5
4.0
3.5
3.0
2.5
2.0
1.5
1.0
0.5
f1 (ppm)

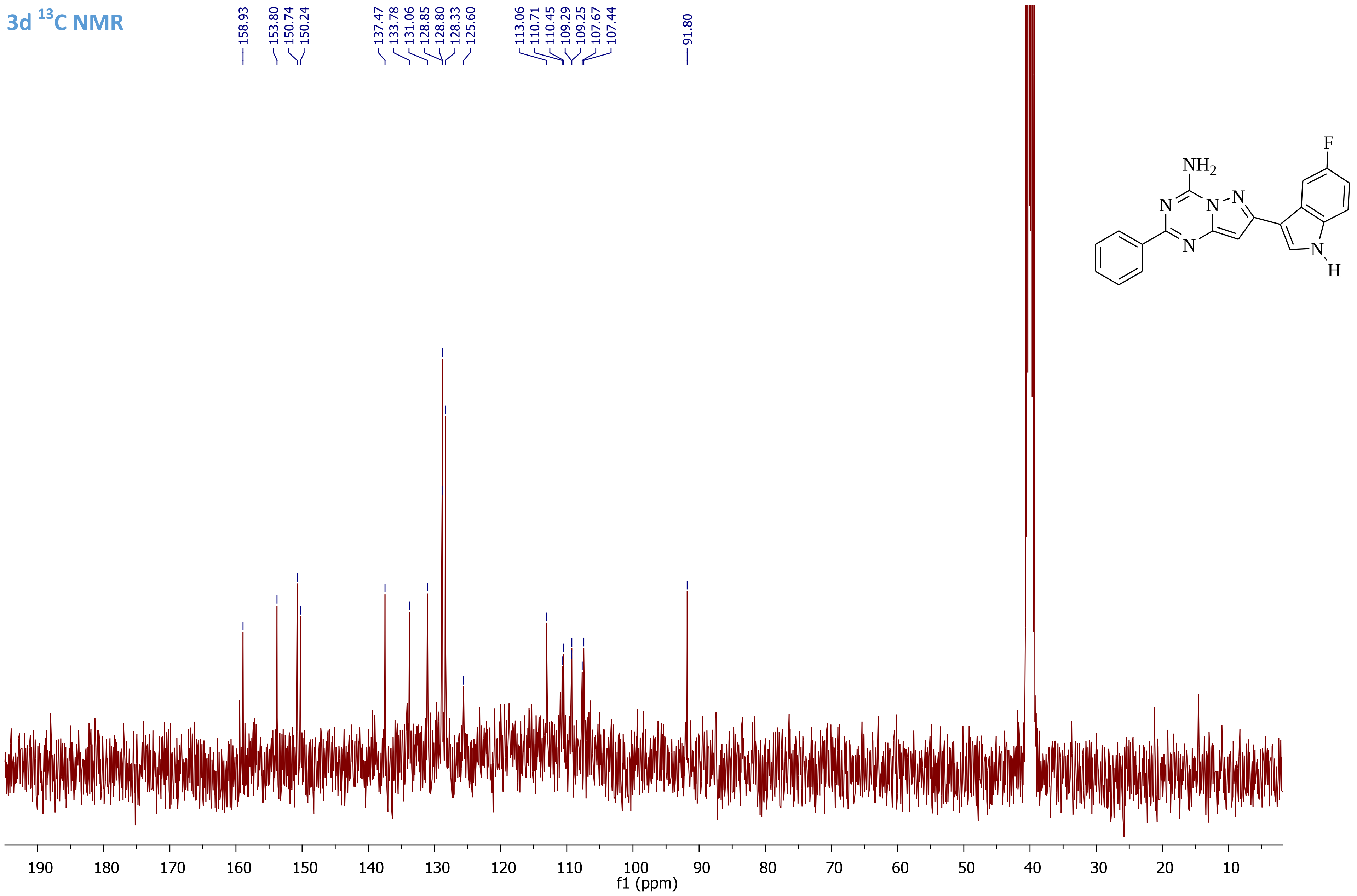
3d 13C NMR
158.93
153.80
150.74
150.24
137.47
133.78
131.06
128.85
128.80
128.33
125.60
113.06
110.71
110.45
109.29
109.25
107.67
107.44
91.80
NH2
N
N
N
N
N
F
N
H
190
180
170
160
150
140
130
120
110
100
90
80
70
60
50
40
30
20
10
f1 (ppm)

3e ^{1}H NMR

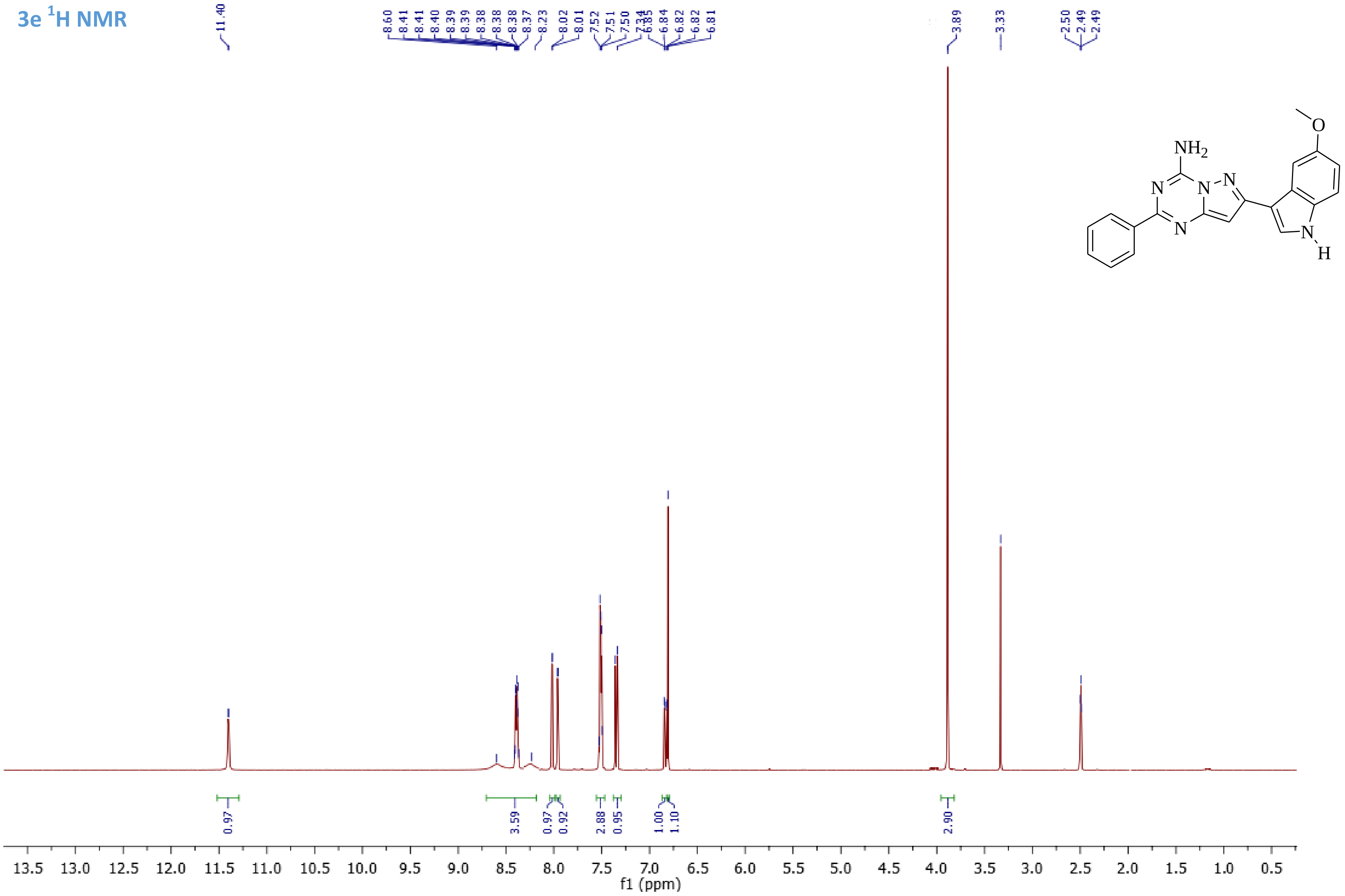
11.40
8.60 8.41 8.41 8.40 8.39 8.39 8.38 8.38 8.38 8.37 8.23 8.02 8.01 7.52 7.51 7.50 7.34 6.85 6.84 6.82 6.82 6.81
3.89
3.33
2.50 2.49 2.49
0.97
3.59
0.97 0.92
2.88 0.95
1.00 1.10
2.90
13.5 13.0 12.5 12.0 11.5 11.0 10.5 10.0 9.5 9.0 8.5 8.0 7.5 7.0 6.5 6.0 5.5 5.0 4.5 4.0 3.5 3.0 2.5 2.0 1.5 1.0 0.5
f1 (ppm)
NH2
N
N
N
N
N
O
H

3e ^{13}C NMR

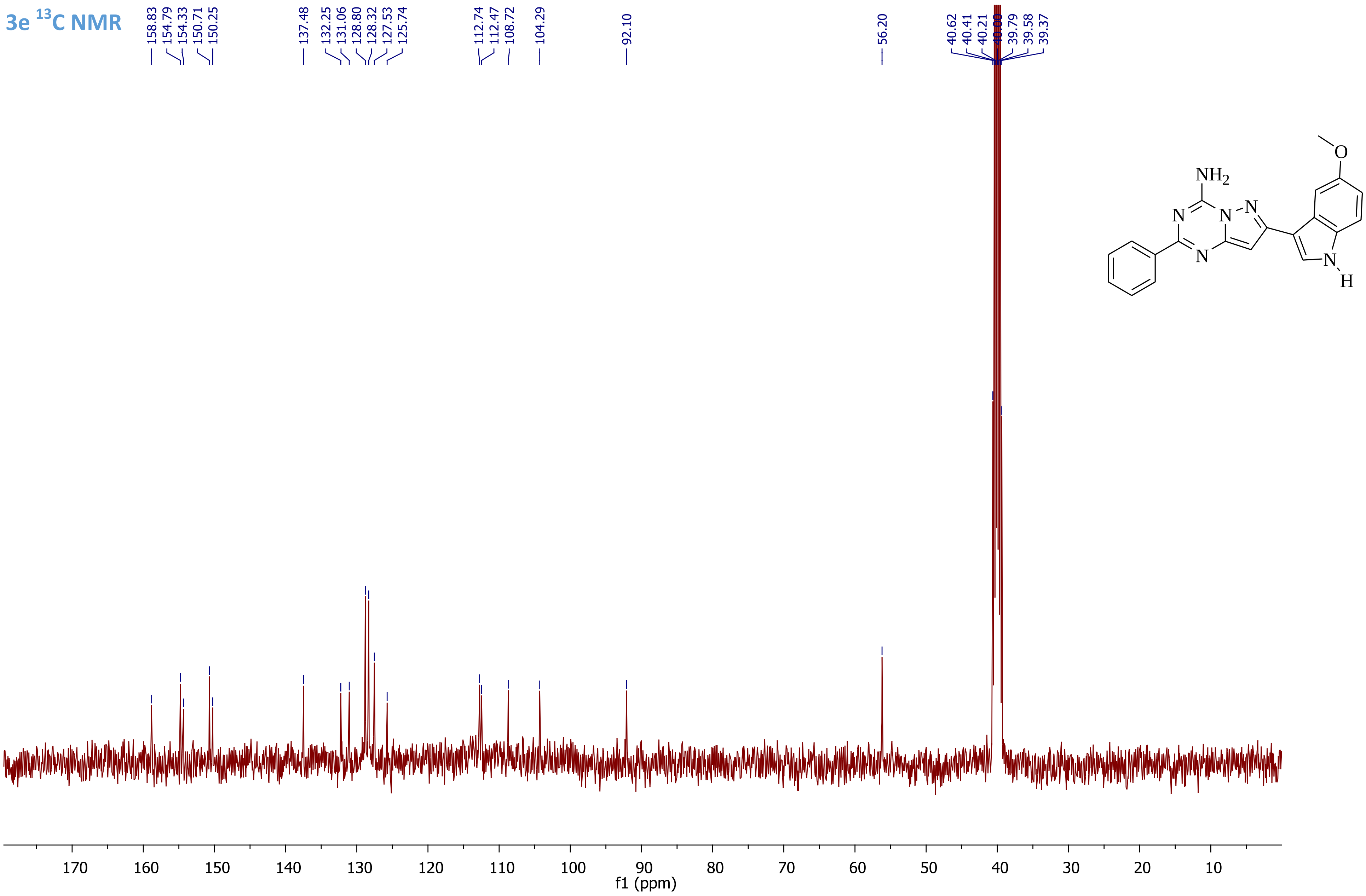
158.83
154.79
154.33
150.71
150.25
137.48
132.25
131.06
128.80
128.32
127.53
125.74
112.74
112.47
108.72
104.29
92.10
56.20
40.62
40.41
40.21
40.00
39.79
39.58
39.37
170
160
150
140
130
120
110
100
90
80
70
60
50
40
30
20
10
f1 (ppm)
NH2
N
N
N
N
N
O
N
H

3f ^{1}H NMR

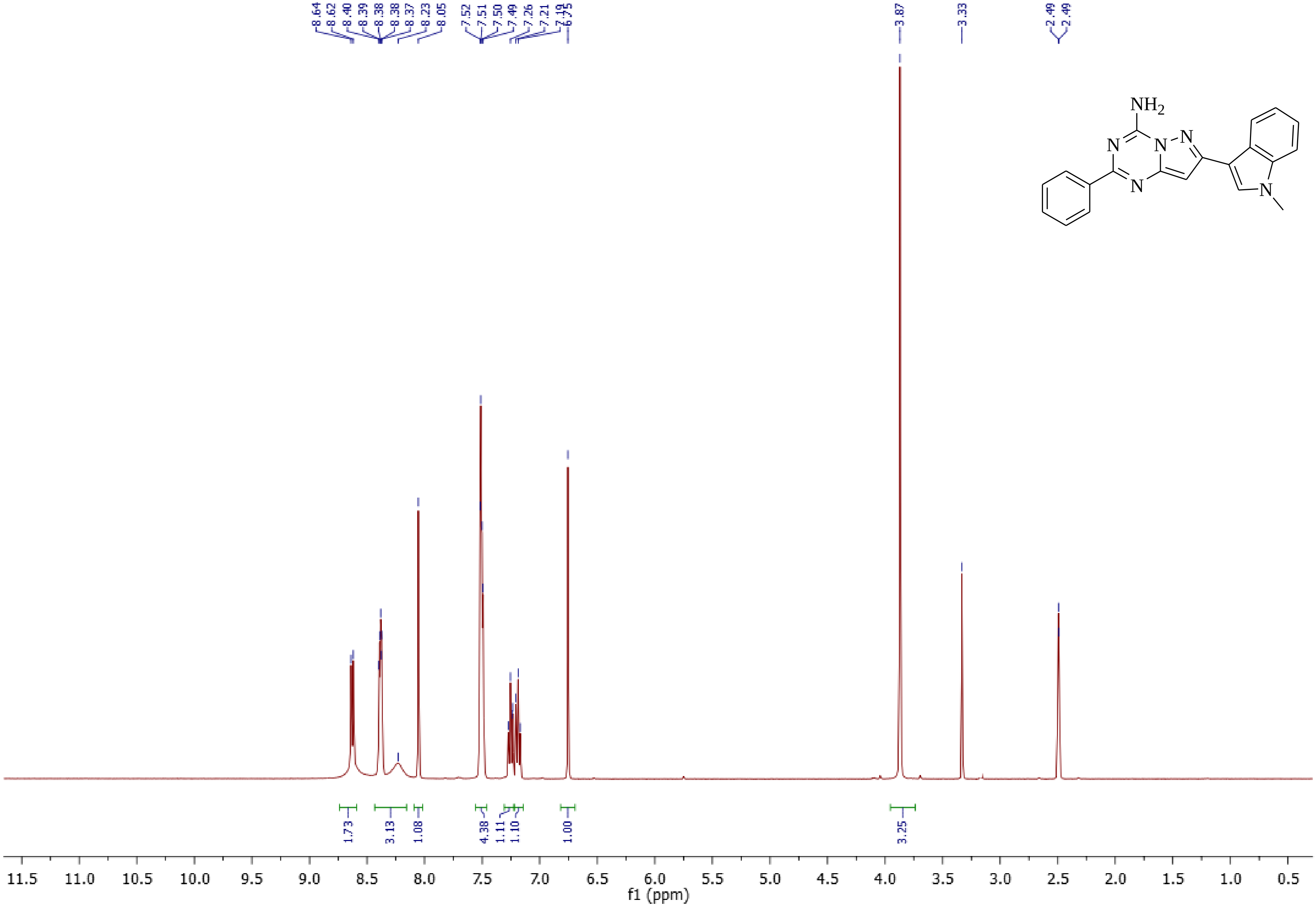

8.64 8.62 8.40 8.39 8.38 8.38 8.37 8.23 8.05 7.52 7.51 7.50 7.49 7.26 7.21 7.19 6.75
3.87
3.33
2.49 2.49
NH2
N
N
N
N
N
1.73 3.13 1.08 4.38 1.11 1.10 1.00 3.25
11.5 11.0 10.5 10.0 9.5 9.0 8.5 8.0 7.5 7.0 6.5 6.0 5.5 5.0 4.5 4.0 3.5 3.0 2.5 2.0 1.5 1.0 0.5
f1 (ppm)

3f ^{13}C NMR

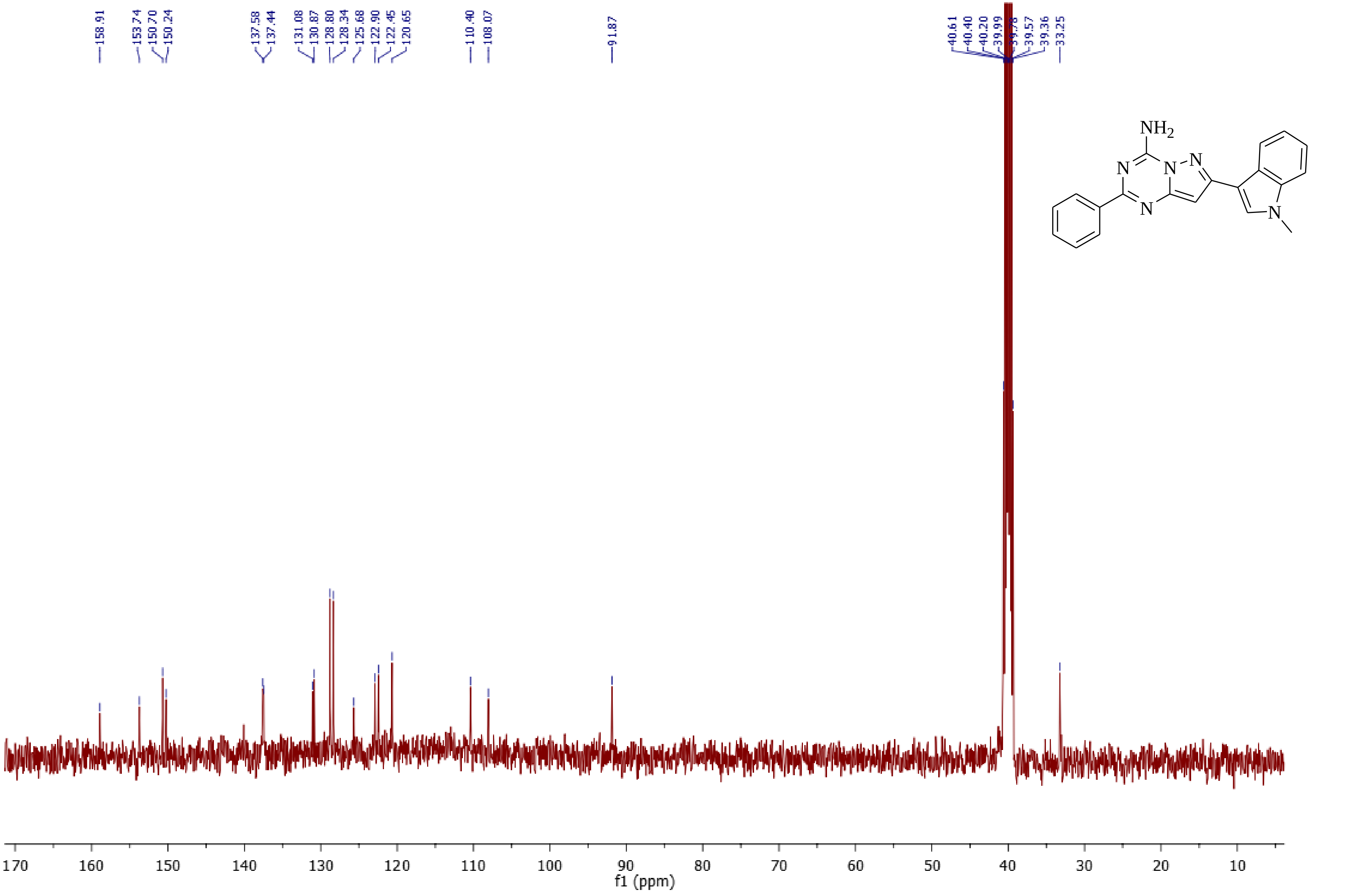
158.91
153.74
150.70
150.24
137.58
137.44
131.08
130.87
128.80
128.34
125.68
122.90
122.45
120.65
110.40
108.07
91.87
40.61
40.40
40.20
39.99
39.78
39.57
39.36
33.25
NH2
N
N
N
N
N
170 160 150 140 130 120 110 100 90 80 70 60 50 40 30 20 10
f1 (ppm)

3g ^{1}H NMR

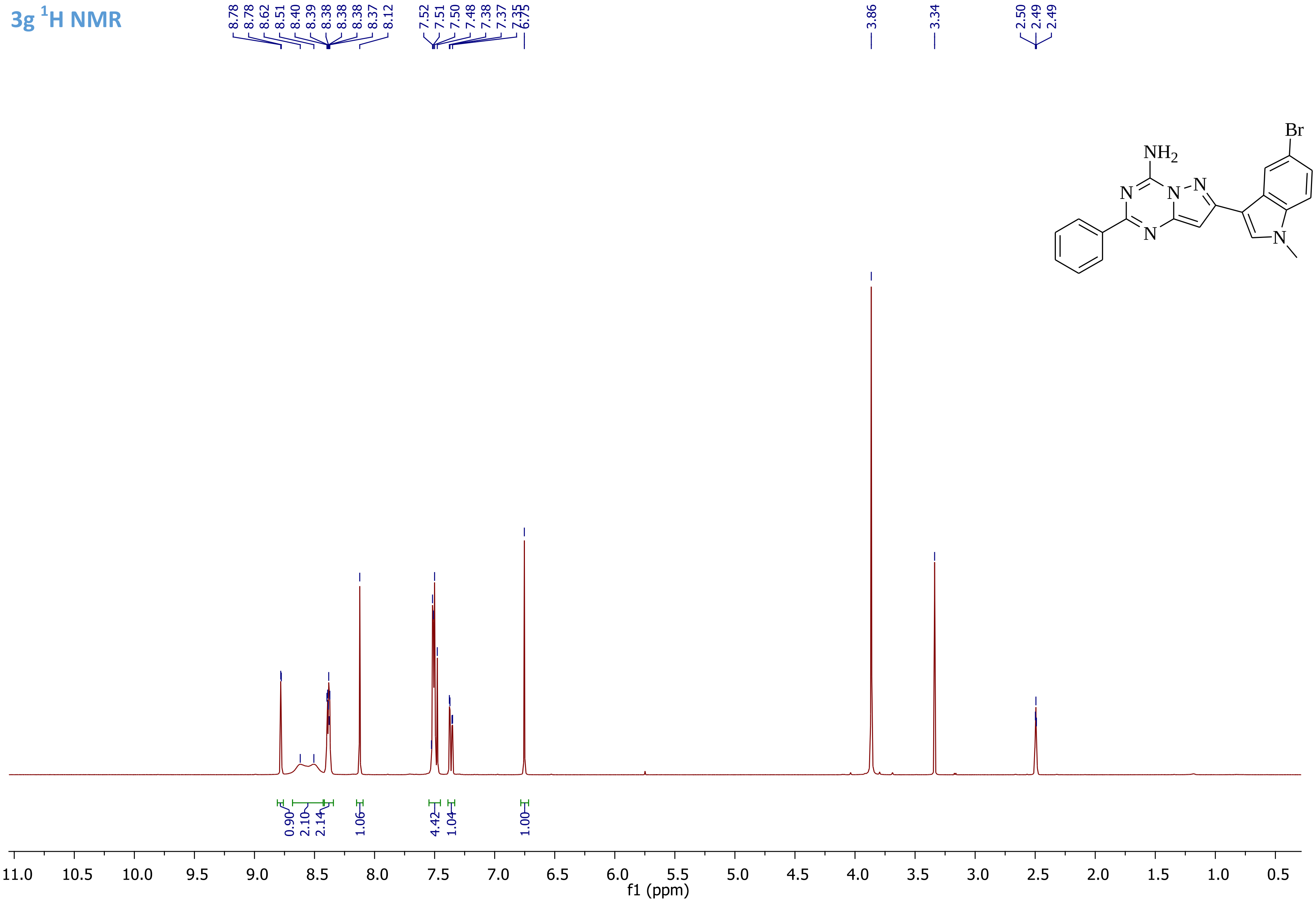
8.78 8.78 8.62 8.51 8.40 8.39 8.38 8.38 8.38 8.37 8.12
7.52 7.51 7.50 7.48 7.38 7.37 7.35 6.75
3.86
3.34
2.50 2.49 2.49
NH2
N
N
N
N
N
Br
N
0.90 2.10 2.14 1.06 4.42 1.04 1.00
11.0 10.5 10.0 9.5 9.0 8.5 8.0 7.5 7.0 6.5 6.0 5.5 5.0 4.5 4.0 3.5 3.0 2.5 2.0 1.5 1.0 0.5
f1 (ppm)

3g ^{13}C NMR

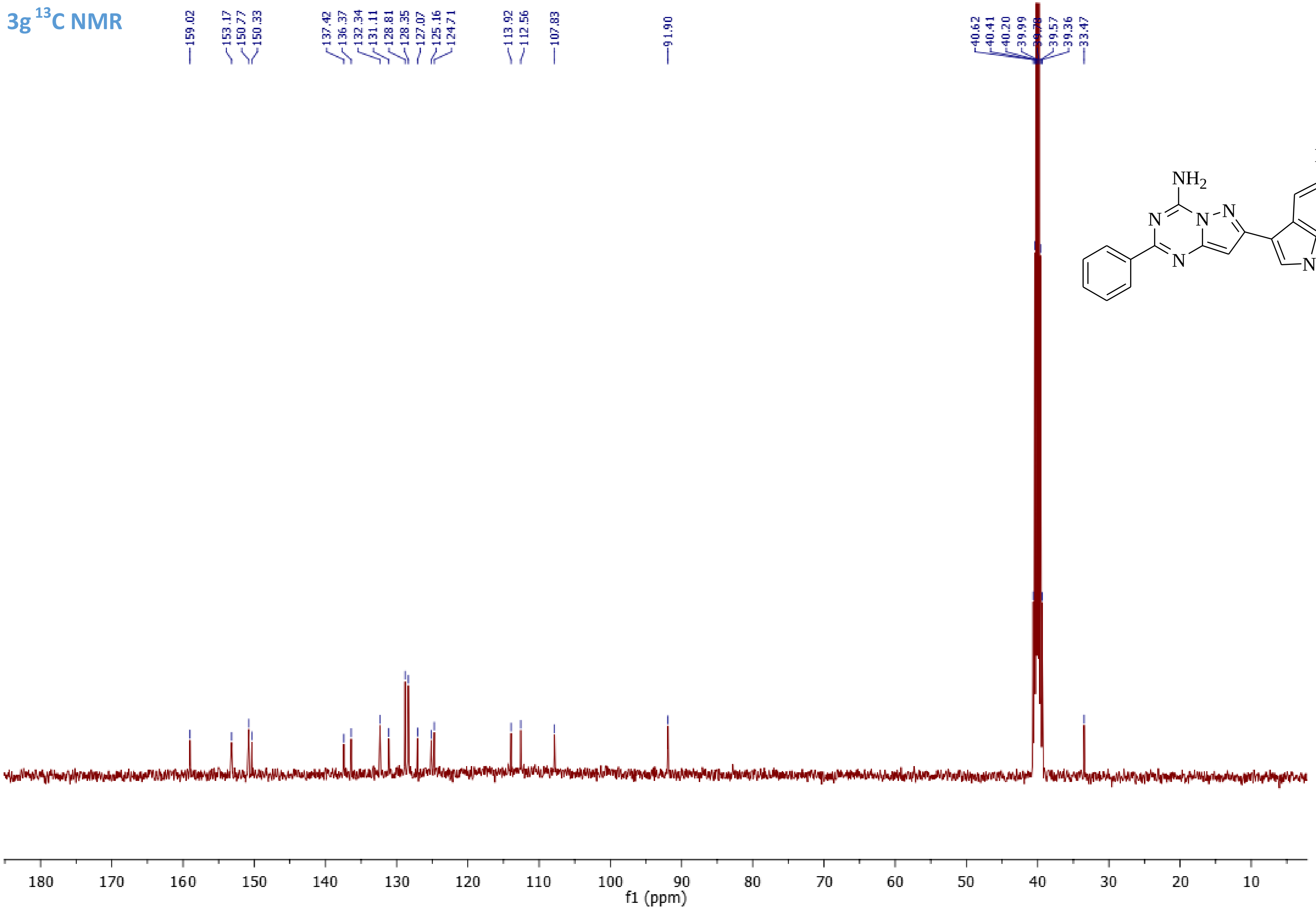
159.02
153.17
150.77
150.33
137.42
136.37
132.34
131.11
128.81
128.35
127.07
125.16
124.71
113.92
112.56
107.83
91.90
40.62
40.41
40.20
39.99
39.78
39.57
39.36
33.47
NH2
N
N
N
N
N
Br
180
170
160
150
140
130
120
110
100
90
80
70
60
50
40
30
20
10
f1 (ppm)

3h ^{1}H NMR

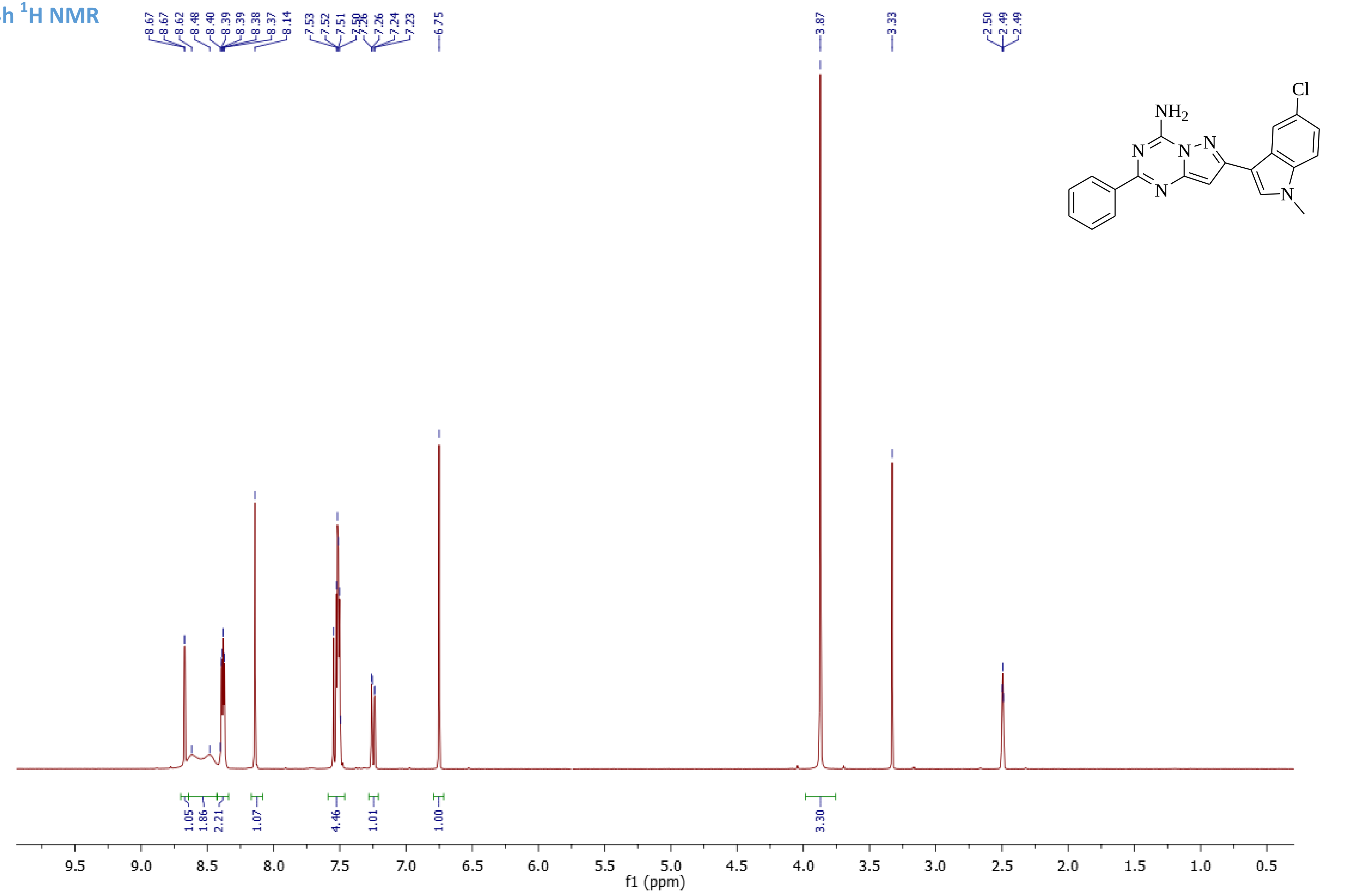
8.67
8.67
8.62
8.48
8.40
8.39
8.39
8.38
8.37
8.14
7.53
7.52
7.51
7.50
7.26
7.24
7.23
6.75
3.87
3.33
2.50
2.49
2.49
1.05
1.86
2.21
1.07
4.46
1.01
1.00
3.30
9.5
9.0
8.5
8.0
7.5
7.0
6.5
6.0
5.5
5.0
4.5
4.0
3.5
3.0
2.5
2.0
1.5
1.0
0.5
f1 (ppm)
Cl
NH2
N
N
N
N
N
N

3h ^{13}C NMR

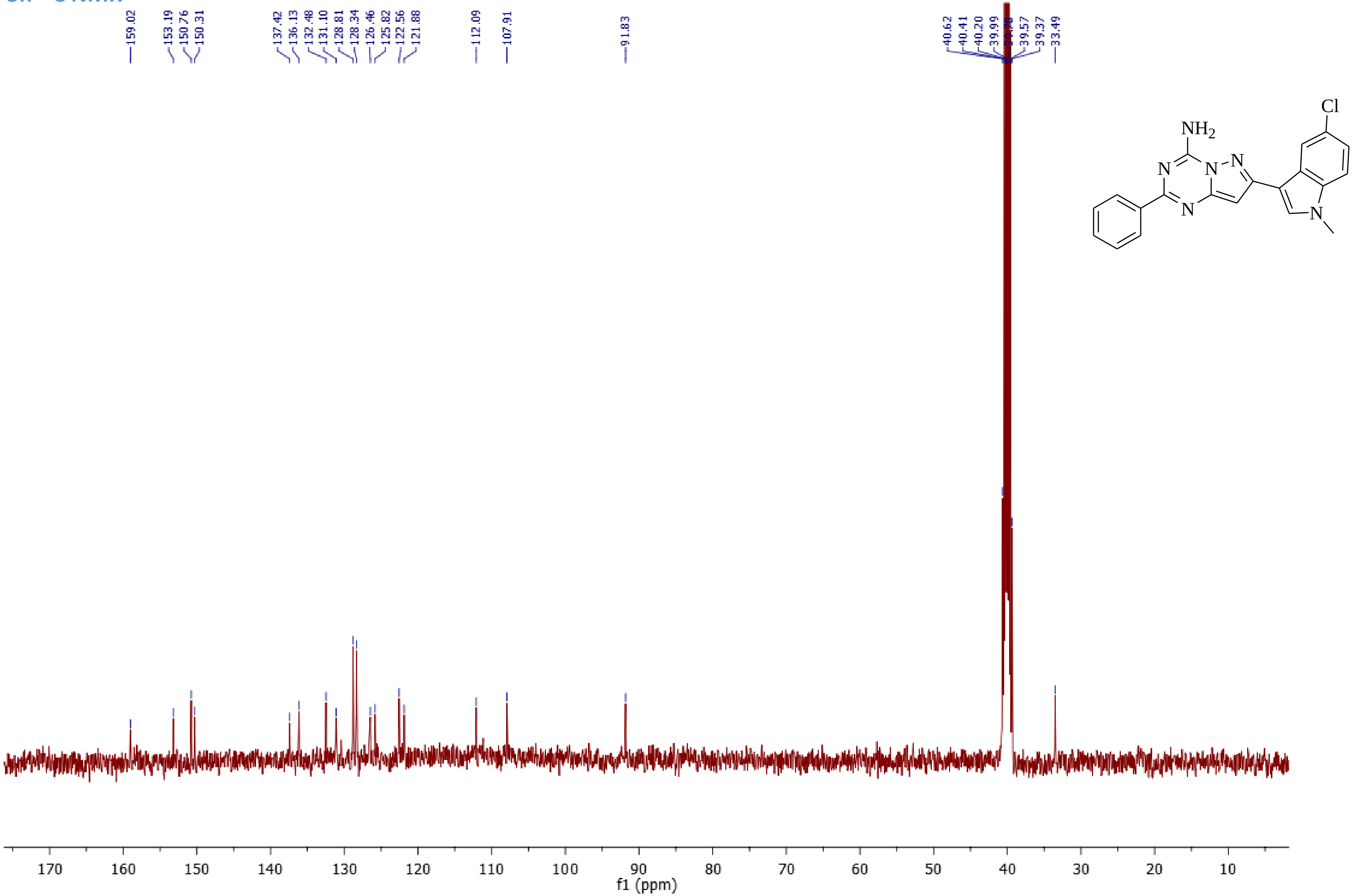
159.02
153.19
150.76
150.31
137.42
136.13
132.48
131.10
128.81
128.34
126.46
125.82
122.56
121.88
112.09
107.91
91.83
40.62
40.41
40.20
39.99
39.57
39.37
33.49
170
160
150
140
130
120
110
100
90
80
70
60
50
40
30
20
10
f1 (ppm)
Cl
NH2
N
N
N
N
N

3i ^{1}H NMR

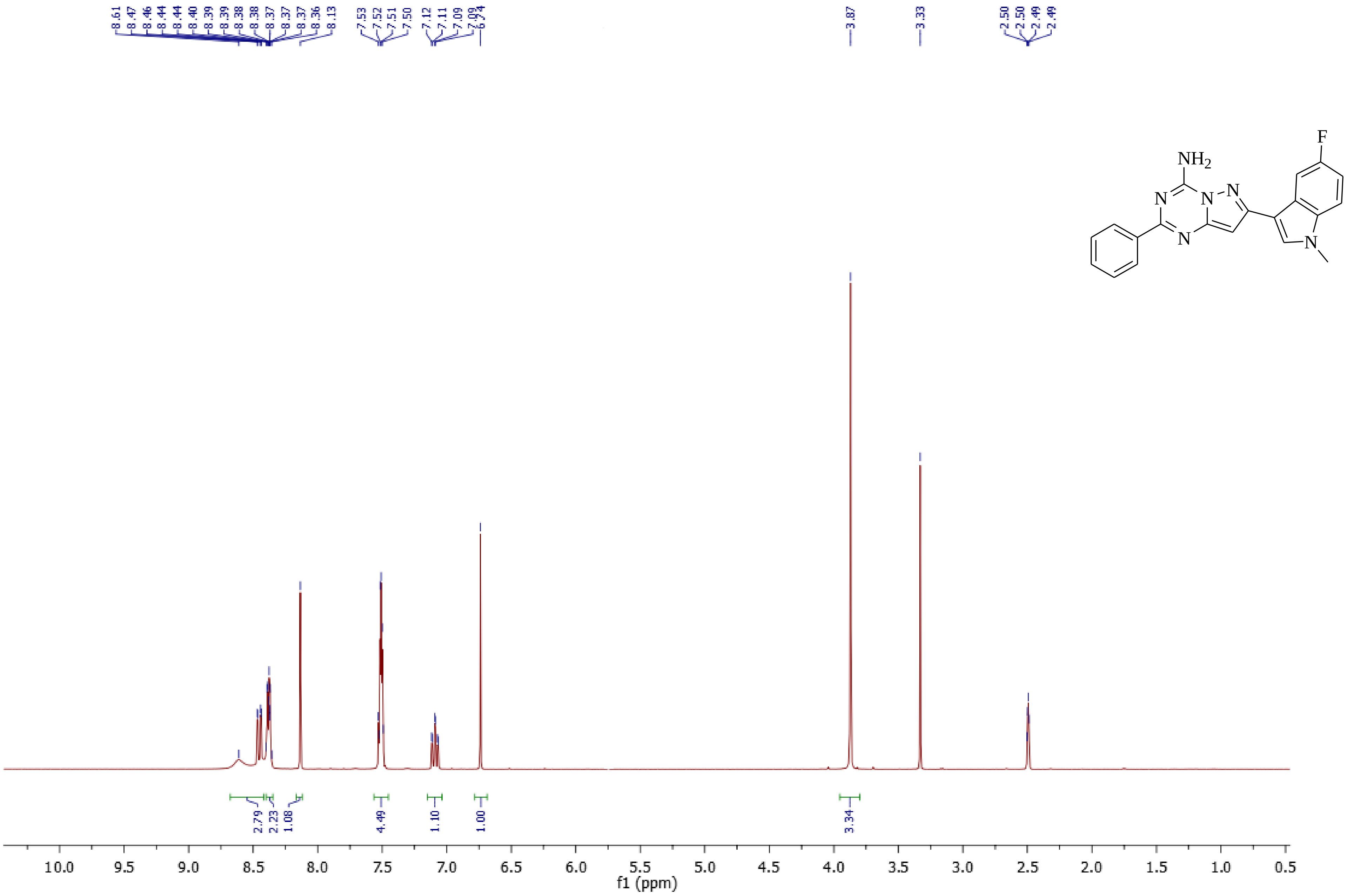
8.61
8.47
8.46
8.44
8.44
8.40
8.39
8.39
8.38
8.38
8.37
8.37
8.37
8.36
8.13
7.53
7.52
7.51
7.50
7.12
7.11
7.09
7.09
6.74
3.87
3.33
2.50
2.50
2.49
2.49
F
NH_2
N
N
N
N
N
N
2.79
2.23
1.08
4.49
1.10
1.00
3.34
10.0
9.5
9.0
8.5
8.0
7.5
7.0
6.5
6.0
5.5
5.0
4.5
4.0
3.5
3.0
2.5
2.0
1.5
1.0
0.5
f1 (ppm)

3i ^{13}C NMR

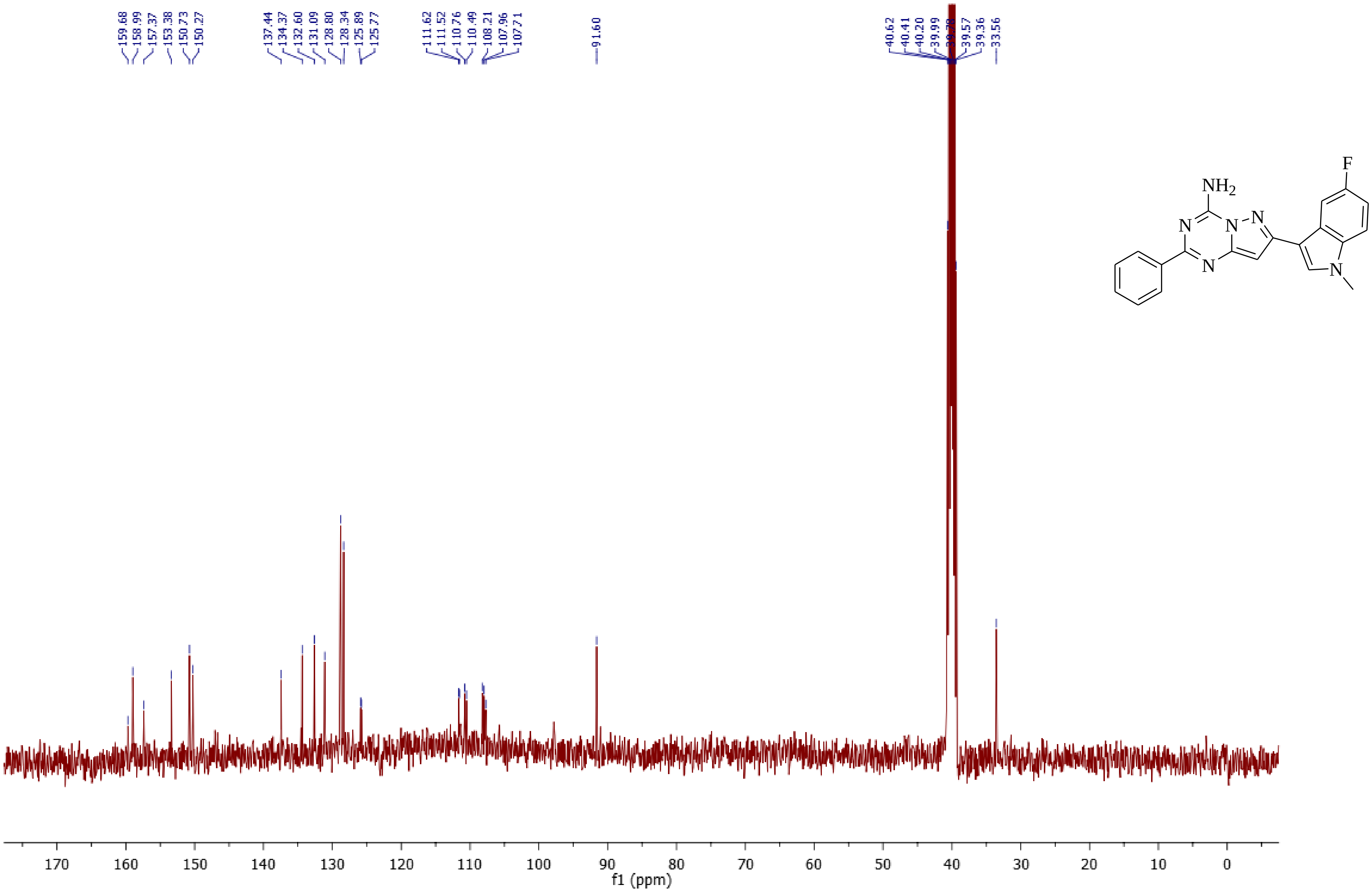
159.68
158.99
157.37
153.38
150.73
150.27
137.44
134.37
132.60
131.09
128.80
128.34
125.89
125.77
111.62
111.52
110.76
110.49
108.21
107.96
107.71
91.60
40.62
40.41
40.20
39.99
39.57
39.36
33.56
NH2
F
N
N
N
N
N
N
170
160
150
140
130
120
110
100
90
80
70
60
50
40
30
20
10
0
f1 (ppm)

3j ^{1}H NMR

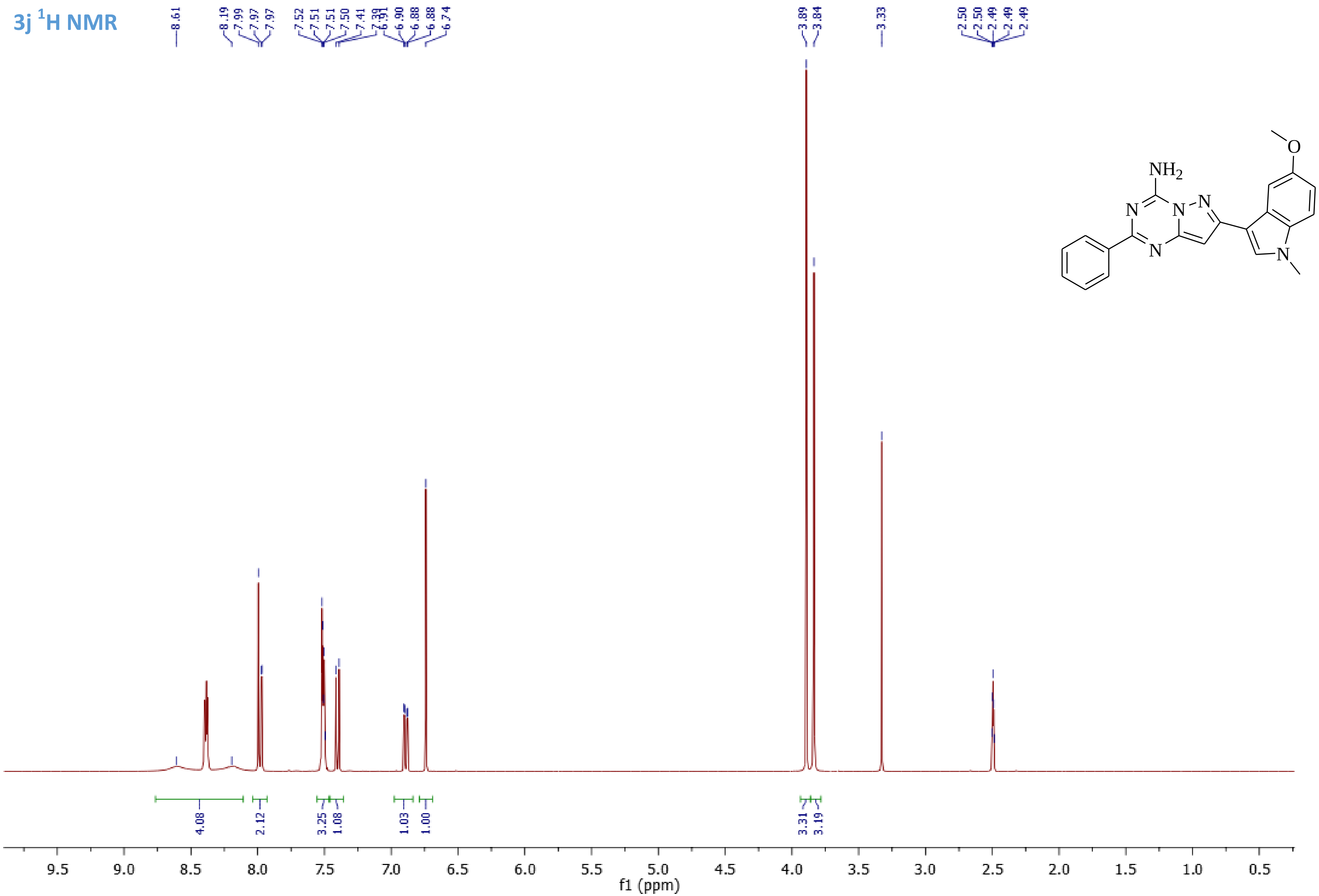
8.61
8.19
7.99
7.97
7.97
7.52
7.51
7.51
7.50
7.41
7.39
6.91
6.90
6.88
6.88
6.74
3.89
3.84
3.33
2.50
2.50
2.49
2.49
2.49
4.08
2.12
3.25
1.08
1.03
1.00
3.31
3.19
9.5
9.0
8.5
8.0
7.5
7.0
6.5
6.0
5.5
5.0
4.5
4.0
3.5
3.0
2.5
2.0
1.5
1.0
0.5
f1 (ppm)
NH2
N
N
N
N
N
O

3j ^{13}C NMR

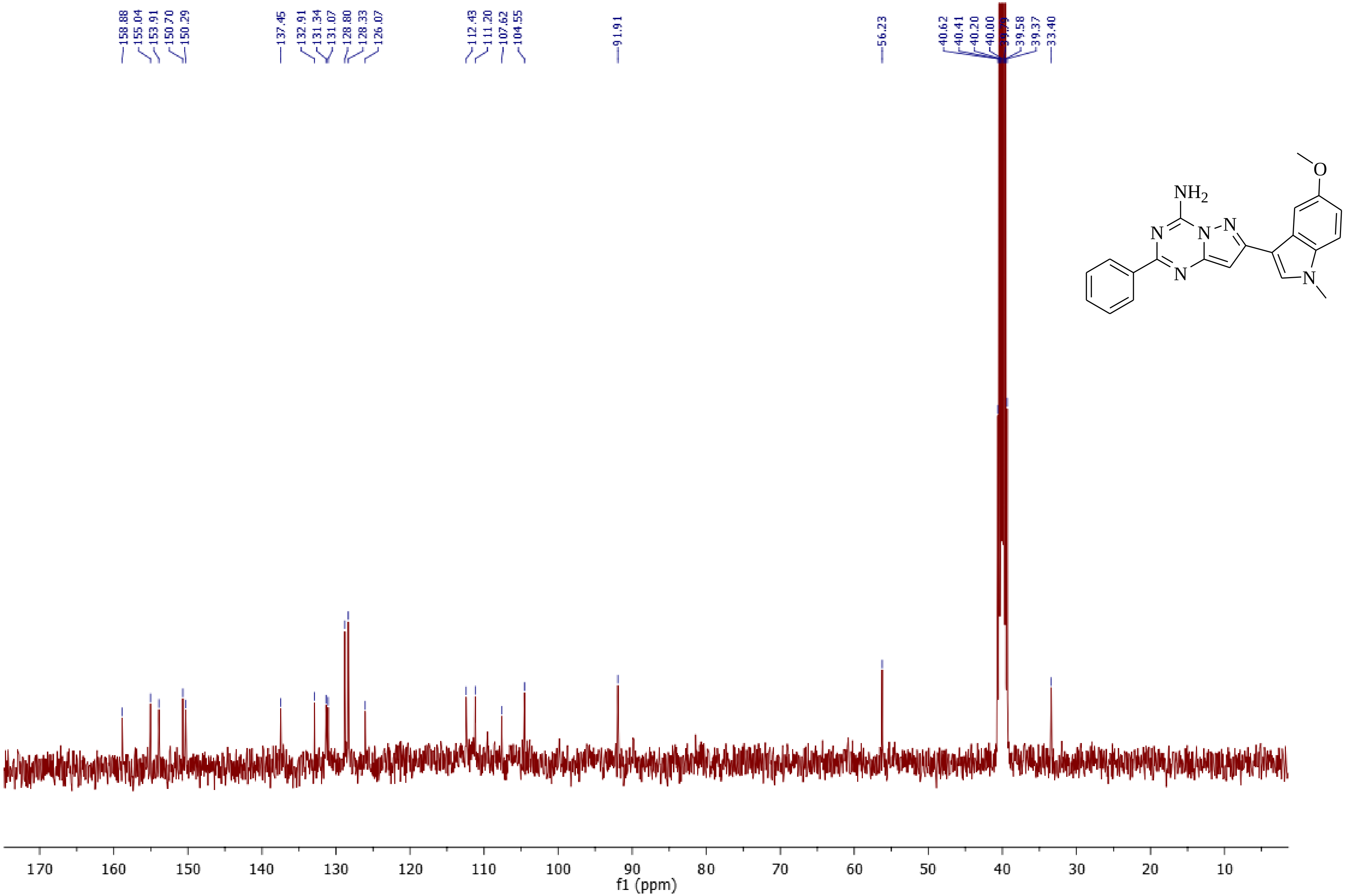
158.88
155.04
153.91
150.70
150.29
137.45
132.91
131.34
131.07
128.80
128.33
126.07
112.43
111.20
107.62
104.55
91.91
56.23
40.62
40.41
40.20
40.00
39.79
39.58
39.37
33.40
NH2
N
N
N
N
N
O
N
170
160
150
140
130
120
110
100
90
80
70
60
50
40
30
20
10
f1 (ppm)

4a ^{1}H NMR

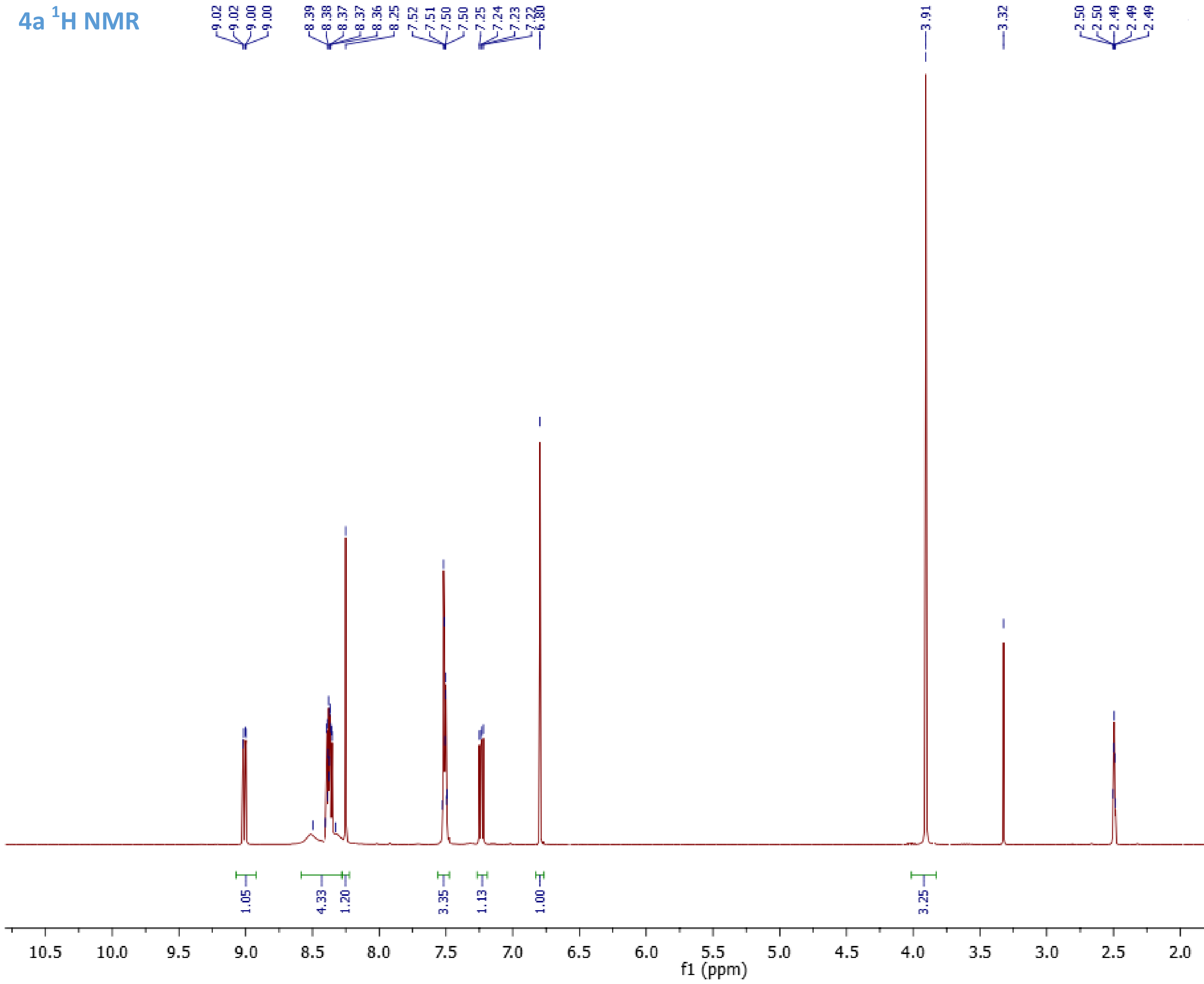
9.02
9.02
9.00
9.00
8.39
8.38
8.37
8.37
8.36
8.25
7.52
7.51
7.50
7.50
7.25
7.24
7.23
7.22
6.80
3.91
3.32
2.50
2.50
2.49
2.49
2.49
1.05
4.33
1.20
3.35
1.13
1.00
3.25
10.5
10.0
9.5
9.0
8.5
8.0
7.5
7.0
6.5
6.0
5.5
5.0
4.5
4.0
3.5
3.0
2.5
2.0
1.5
1.0
0.5
f1 (ppm)

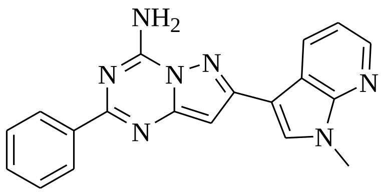
NH2
N
N
N
N
N
N

4a ^{13}C NMR

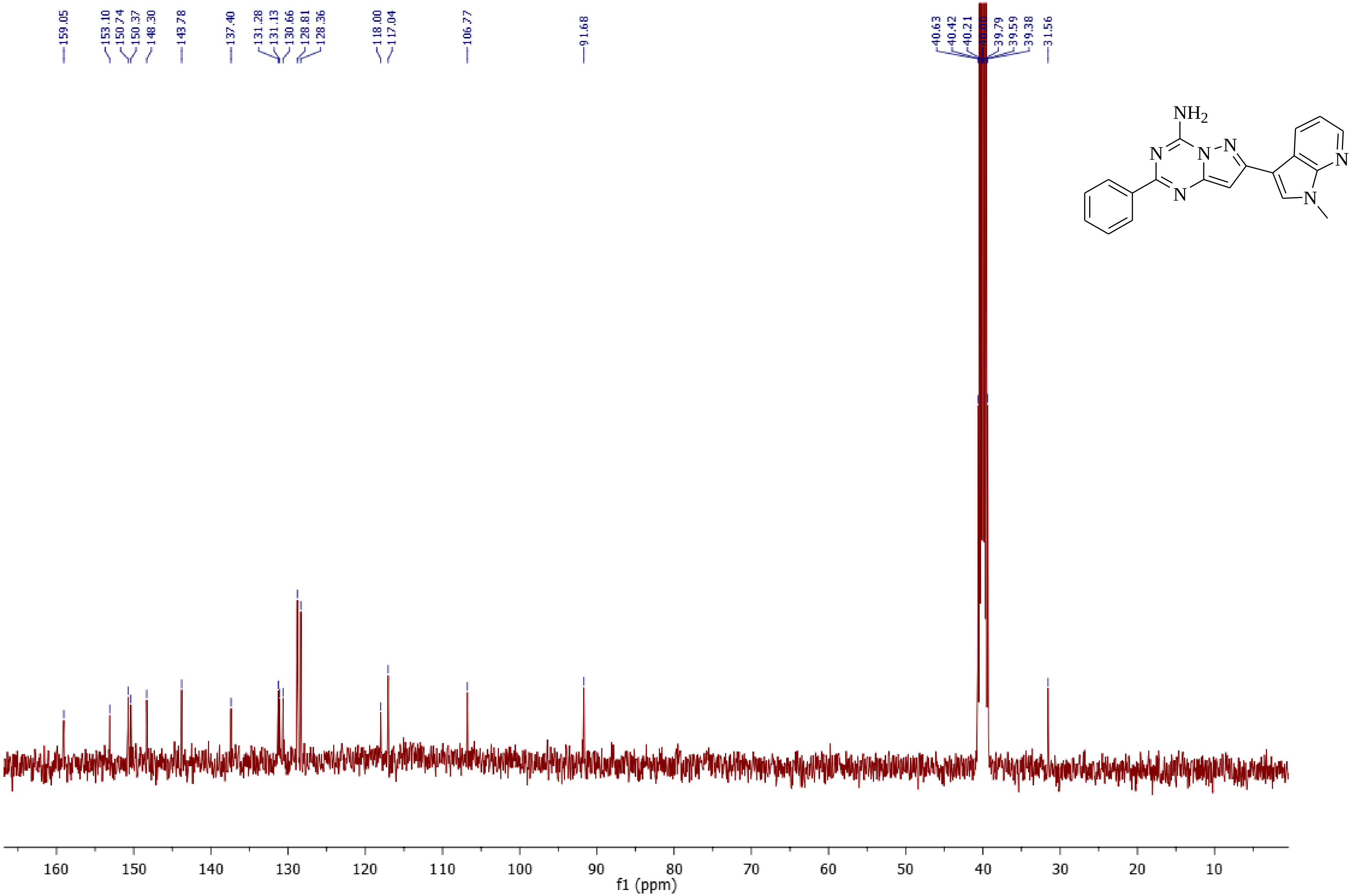
159.05
153.10
150.74
150.37
148.30
143.78
137.40
131.28
131.13
130.66
128.81
128.36
118.00
117.04
106.77
91.68
40.63
40.42
40.21
39.79
39.59
39.38
31.56
NH2
N
N
N
N
N
N
160
150
140
130
120
110
100
90
80
70
60
50
40
30
20
10
f1 (ppm)

4b ^{1}H NMR

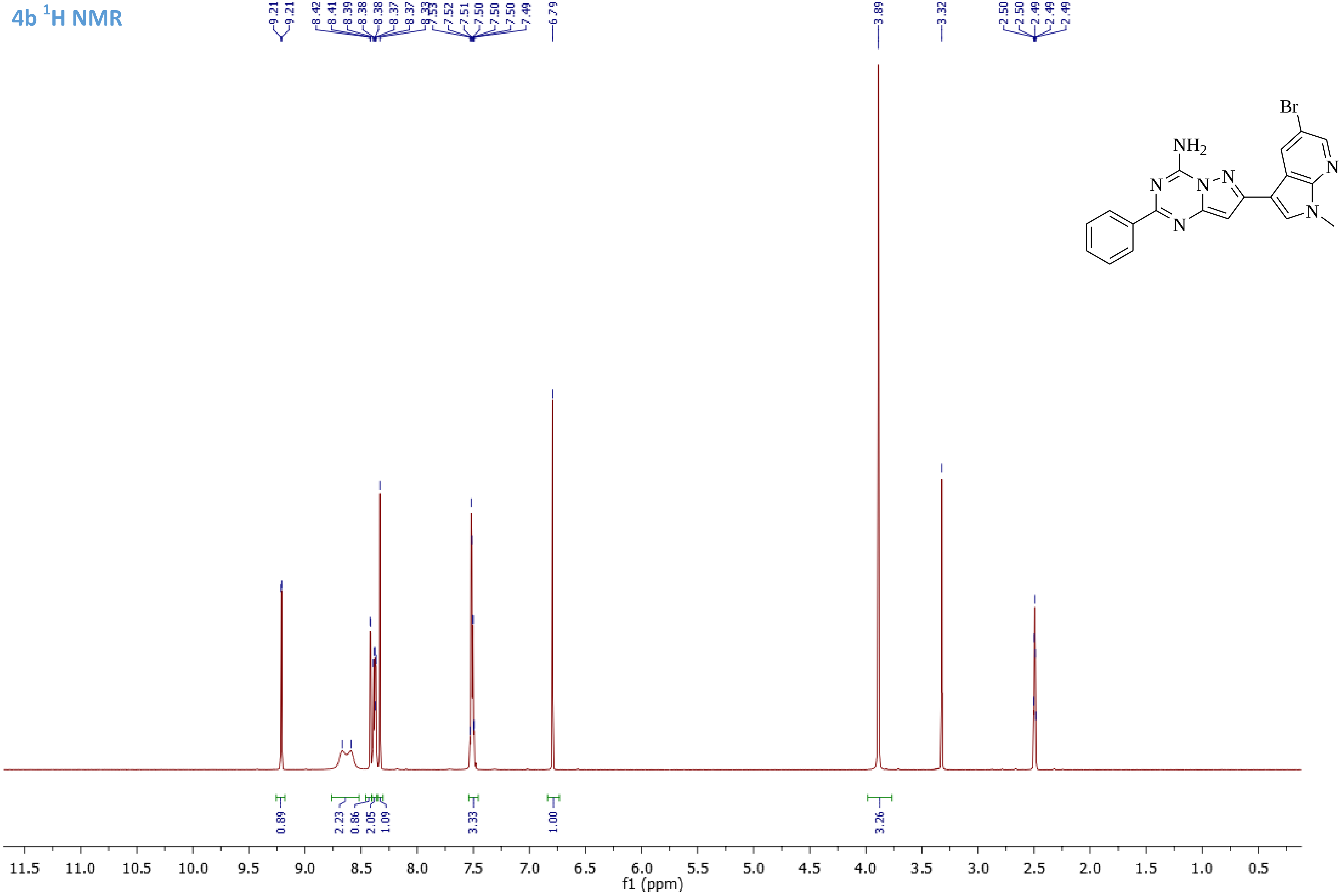
9.21
9.21
8.42
8.41
8.39
8.38
8.38
8.37
8.37
8.33
7.53
7.52
7.51
7.50
7.50
7.50
7.49
6.79
3.89
3.32
2.50
2.50
2.49
2.49
2.49
Br
NH2
N
N
N
N
N
N
N
0.89
2.23
0.86
2.05
1.09
3.33
1.00
3.26
11.5 11.0 10.5 10.0 9.5 9.0 8.5 8.0 7.5 7.0 6.5 6.0 5.5 5.0 4.5 4.0 3.5 3.0 2.5 2.0 1.5 1.0 0.5
f1 (ppm)

4b 13C NMR

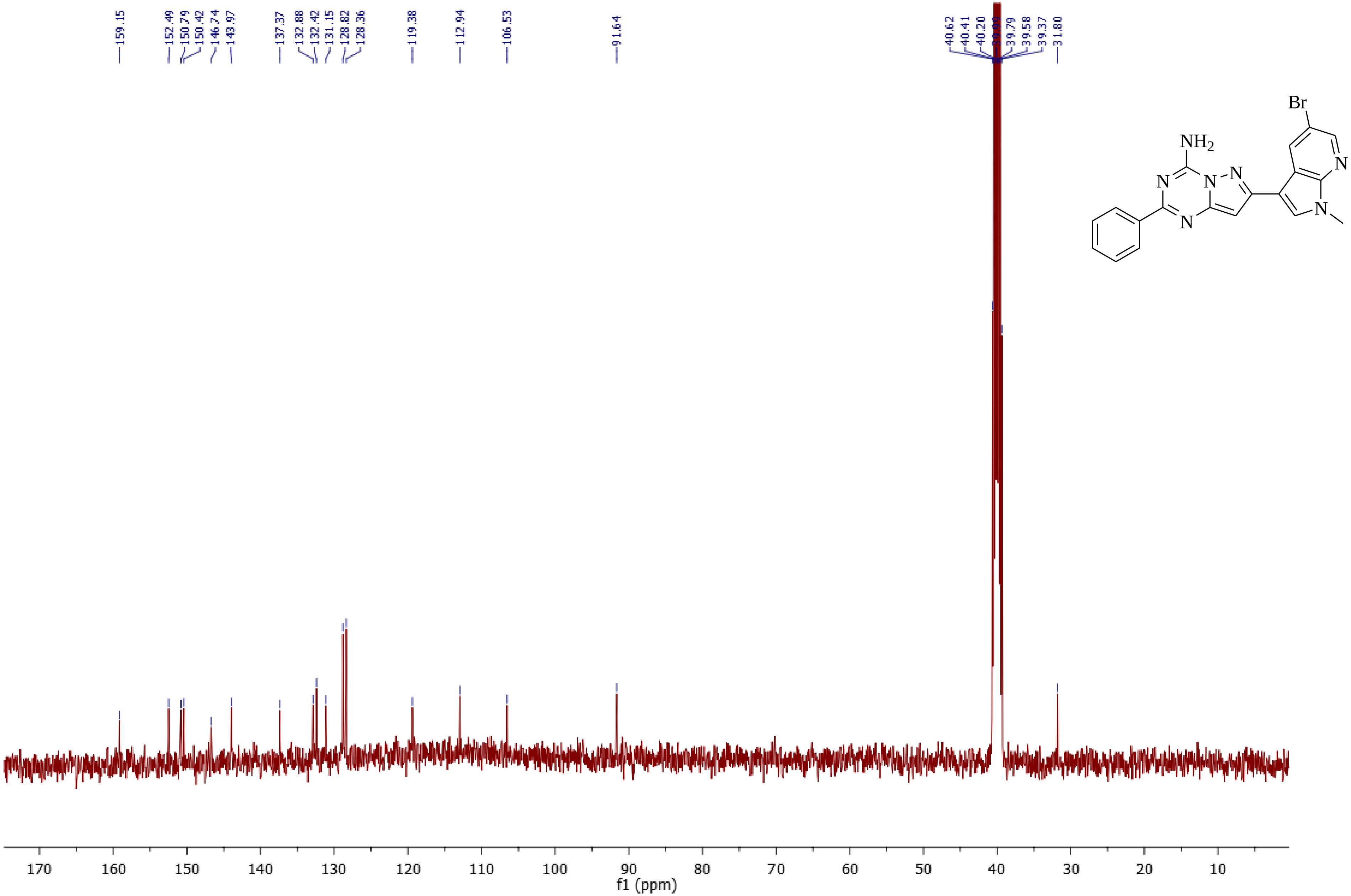
159.15
152.49
150.79
150.42
146.74
143.97
137.37
132.88
132.42
131.15
128.82
128.36
119.38
112.94
106.53
91.64
40.62
40.41
40.20
39.79
39.58
39.37
31.80
Br
NH2
N
N
N
N
N
N
N
170
160
150
140
130
120
110
100
90
80
70
60
50
40
30
20
10
f1 (ppm)

5a ^{1}H NMR

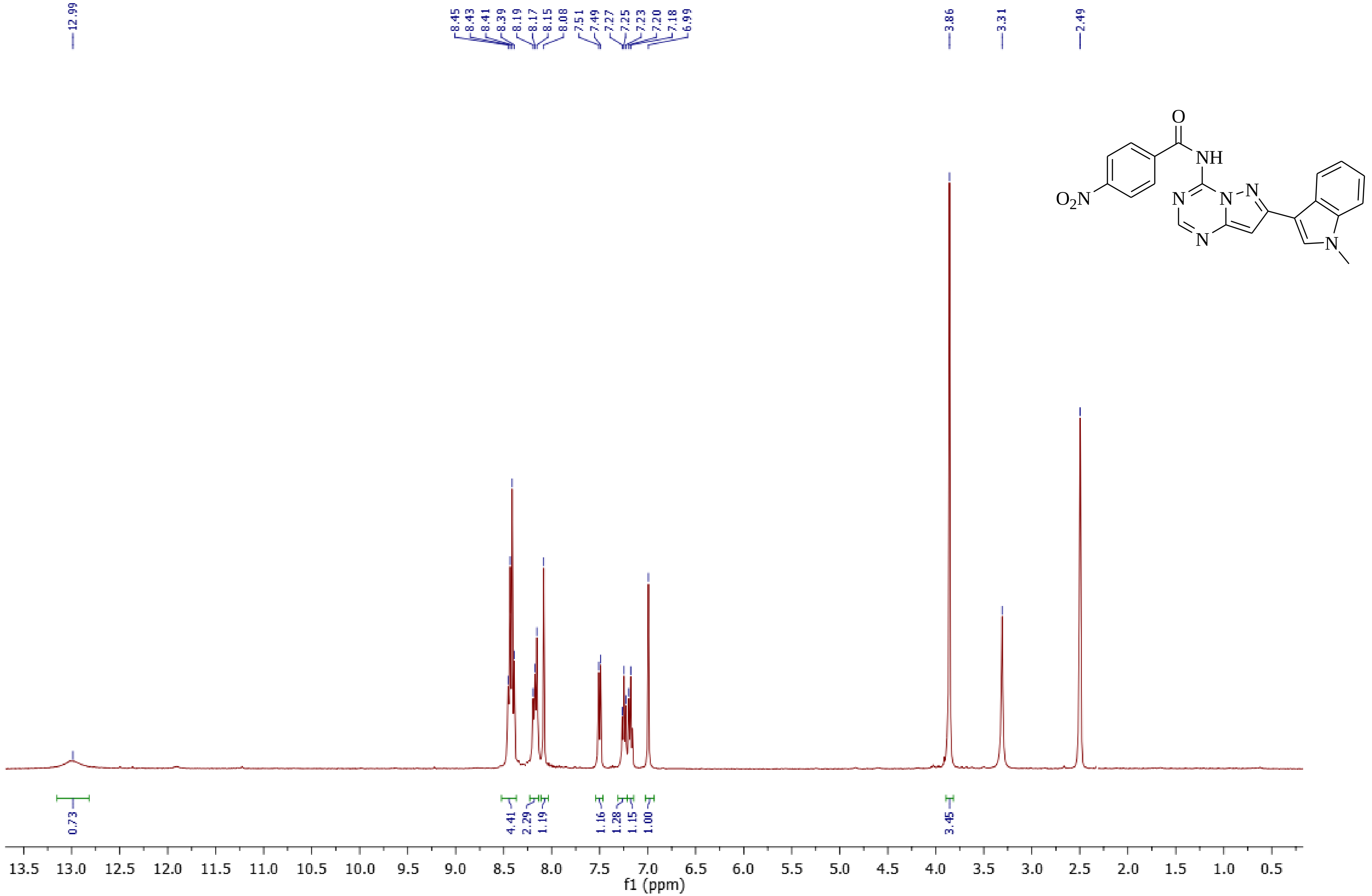
12.99
8.45
8.43
8.41
8.39
8.19
8.17
8.15
8.08
7.51
7.49
7.27
7.25
7.23
7.20
7.18
6.99
3.86
3.31
2.49
O
NH
O_2N
N
N
N
N
N
0.73
4.41
2.29
1.19
1.16
1.28
1.15
1.00
3.45
13.5 13.0 12.5 12.0 11.5 11.0 10.5 10.0 9.5 9.0 8.5 8.0 7.5 7.0 6.5 6.0 5.5 5.0 4.5 4.0 3.5 3.0 2.5 2.0 1.5 1.0 0.5
f1 (ppm)

5a ^{13}C NMR

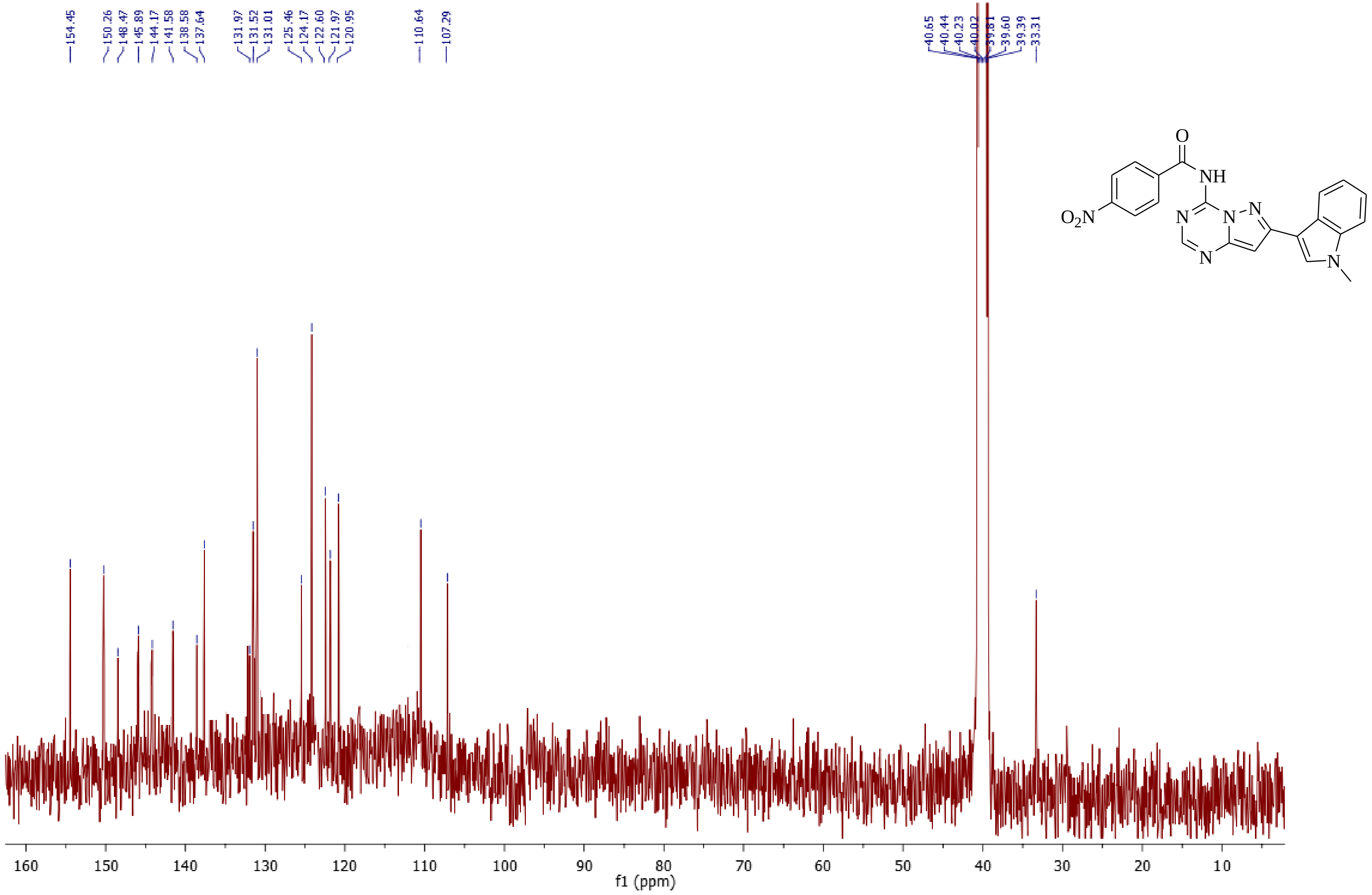
154.45
150.26
148.47
145.89
144.17
141.58
138.58
137.64
131.97
131.52
131.01
125.46
124.17
122.60
121.97
120.95
110.64
107.29
40.65
40.44
40.23
40.02
39.81
39.60
39.39
33.31
f1 (ppm)
O
NH
O_2N
N
N
N
N
N
N

5b ^{1}H NMR

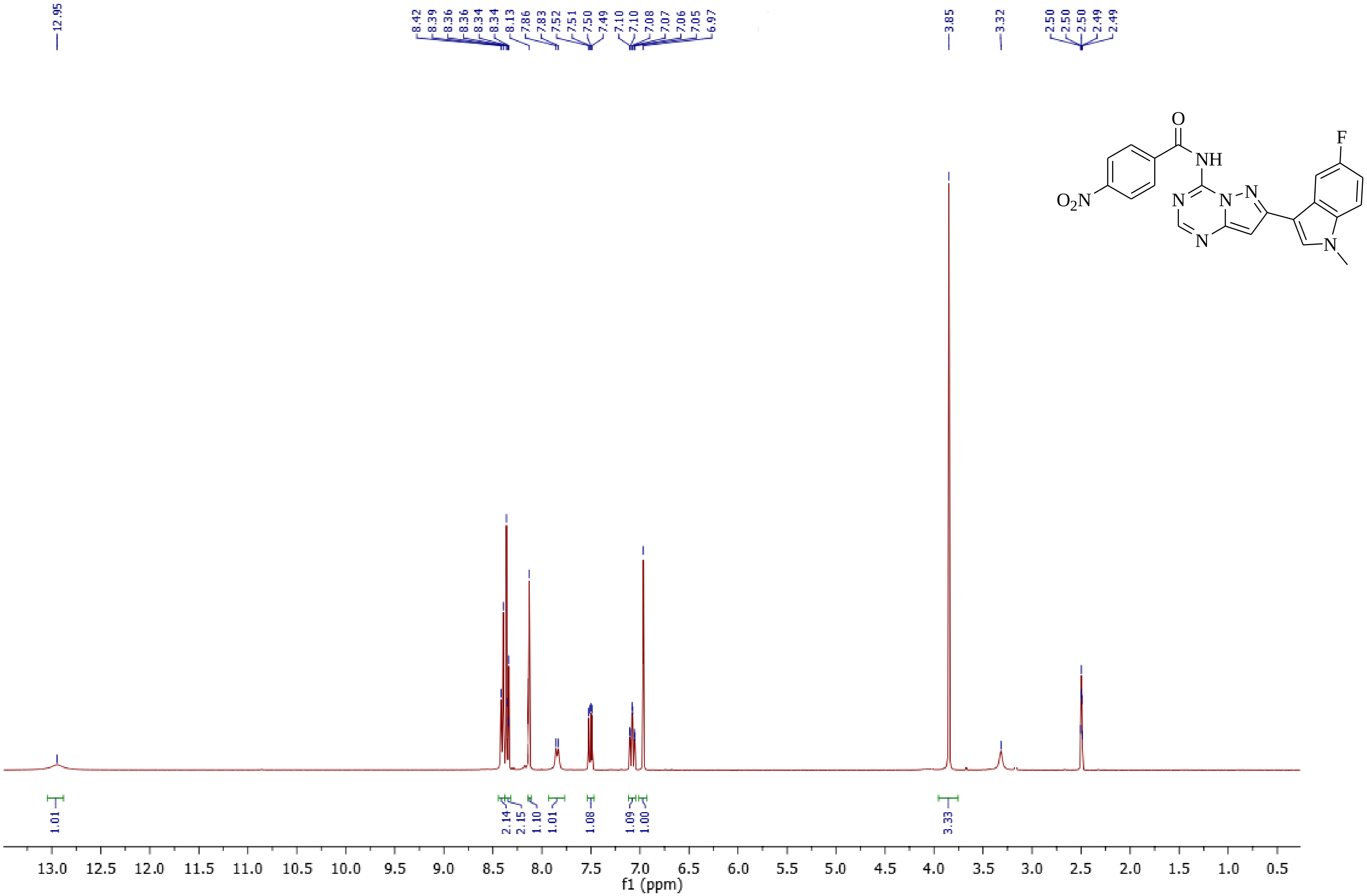
12.95
8.42
8.39
8.36
8.36
8.34
8.34
8.13
7.86
7.83
7.52
7.51
7.50
7.49
7.10
7.10
7.08
7.07
7.06
7.05
6.97
3.85
3.32
2.50
2.50
2.50
2.49
2.49
1.01
2.14
2.15
1.10
1.01
1.08
1.09
1.00
3.33
f1 (ppm)
O
NH
F
O_2N
N
N
N
N
N
N

5b ^{13}C NMR

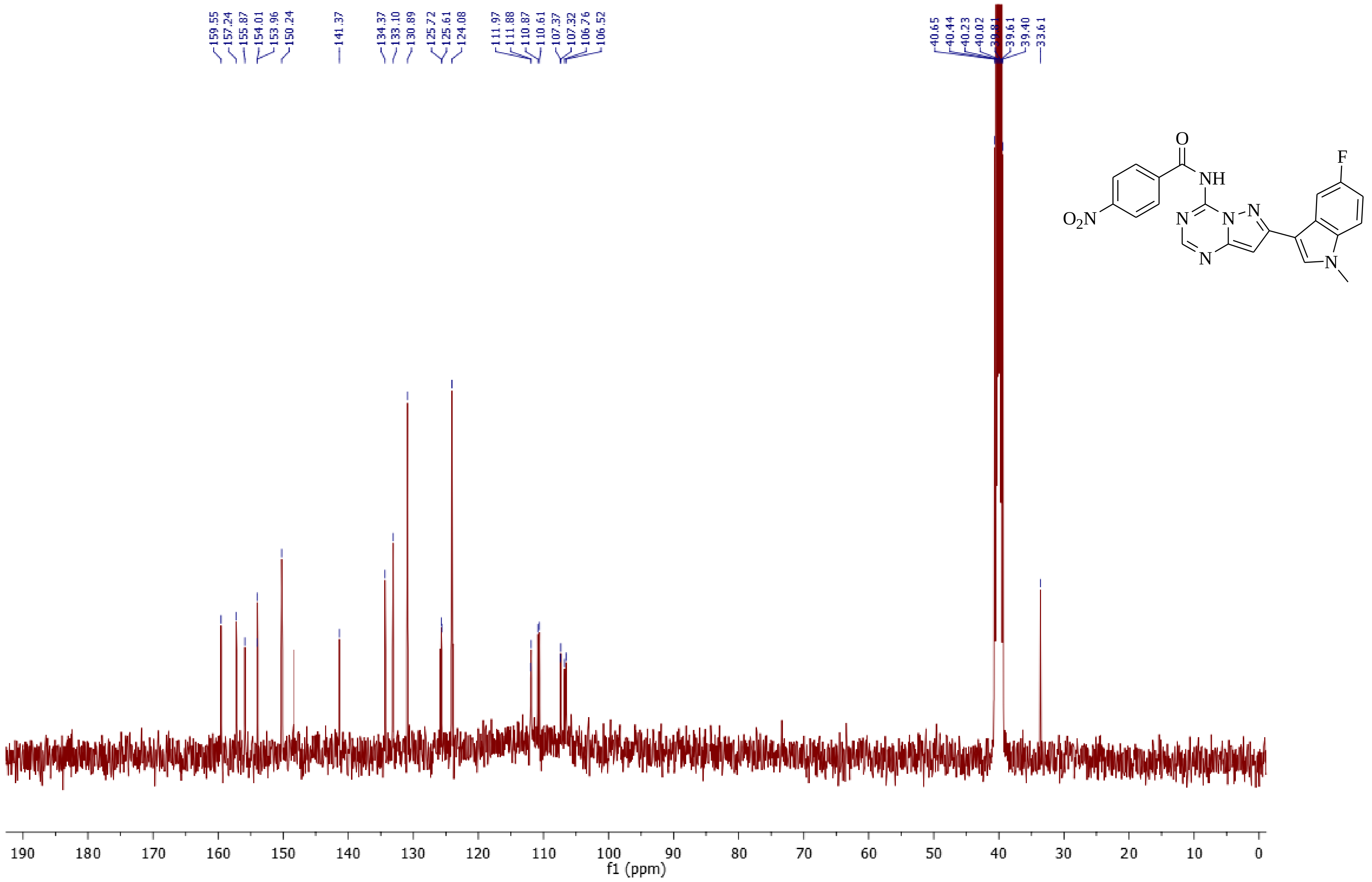
159.55
157.24
155.87
154.01
153.96
150.24
141.37
134.37
133.10
130.89
125.72
125.61
124.08
111.97
111.88
110.87
110.61
107.37
107.32
106.76
106.52
40.65
40.44
40.23
40.02
39.81
39.61
39.40
33.61
O
NH
F
O_2N
N
N
N
N
N
190 180 170 160 150 140 130 120 110 100 90 80 70 60 50 40 30 20 10 0
f1 (ppm)

6a ^{1}H NMR

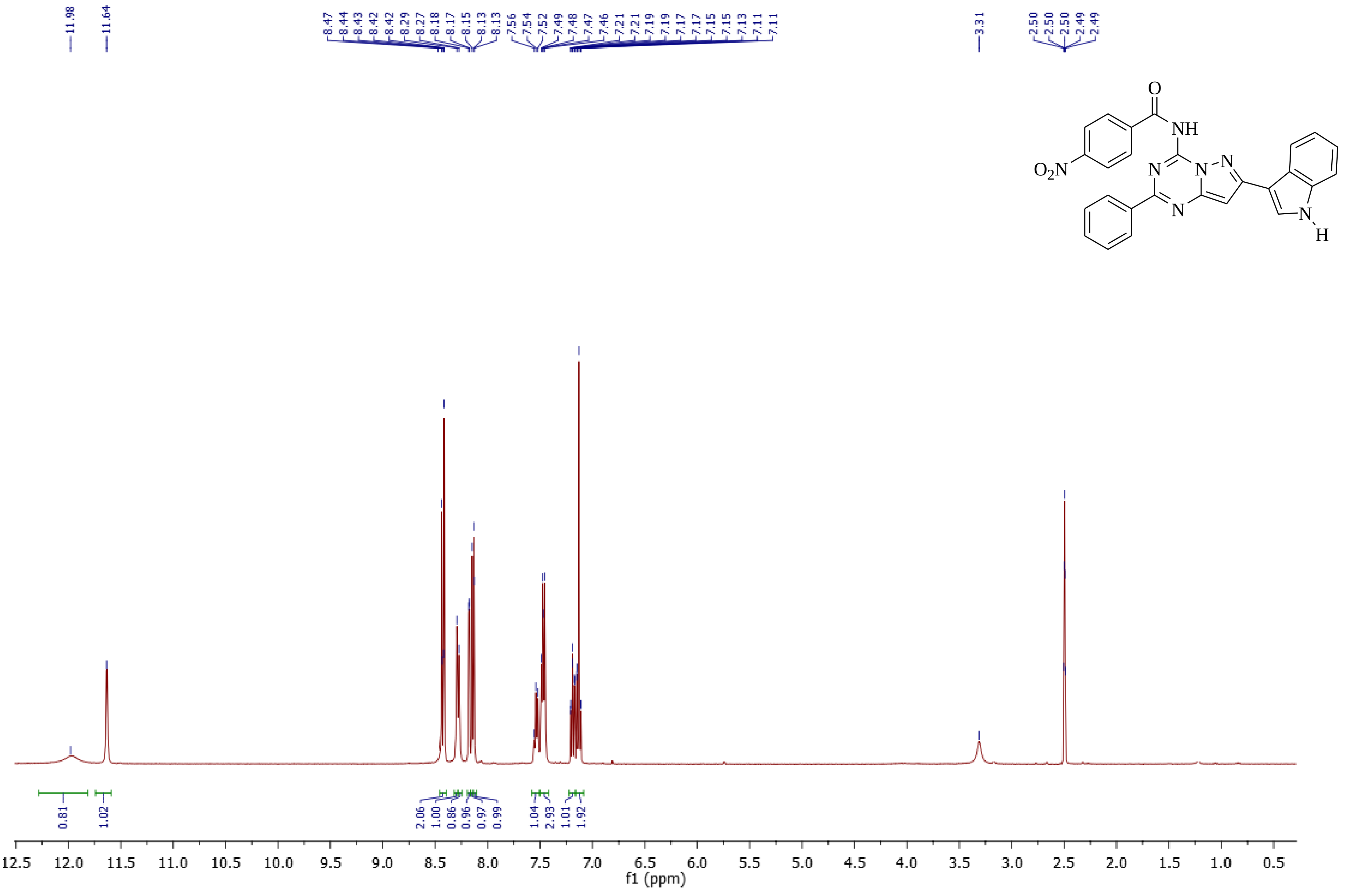
11.98
11.64
8.47
8.44
8.43
8.42
8.42
8.29
8.27
8.18
8.17
8.15
8.13
8.13
7.56
7.54
7.52
7.49
7.48
7.47
7.46
7.21
7.21
7.19
7.19
7.17
7.17
7.15
7.15
7.13
7.11
7.11
3.31
2.50
2.50
2.50
2.49
2.49
O
NH
O2N
N
N
N
N
N
H
0.81
1.02
2.06
1.00
0.86
0.96
0.97
0.99
1.04
2.93
1.01
1.92
12.5
12.0
11.5
11.0
10.5
10.0
9.5
9.0
8.5
8.0
7.5
7.0
6.5
6.0
5.5
5.0
4.5
4.0
3.5
3.0
2.5
2.0
1.5
1.0
0.5
f1 (ppm)

6a ^{13}C NMR

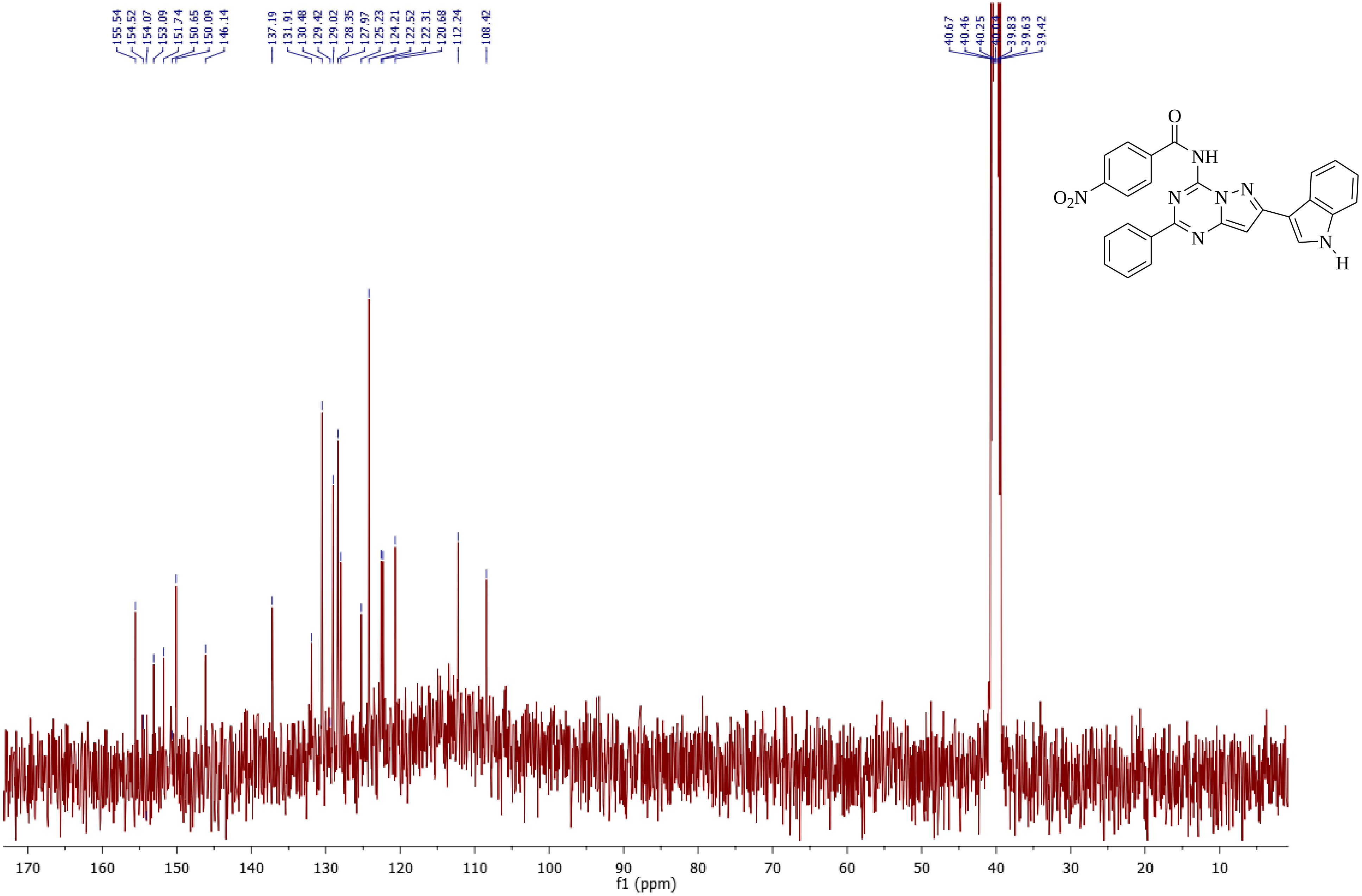
155.54
154.52
154.07
153.09
151.74
150.65
150.09
146.14
137.19
131.91
130.48
129.42
129.02
128.35
127.97
125.23
124.21
122.52
122.31
120.68
112.24
108.42
40.67
40.46
40.25
39.83
39.63
39.42
O
NH
O_2N
N
N
N
N
N
H
170
160
150
140
130
120
110
100
90
80
70
60
50
40
30
20
10
f1 (ppm)

6b ^{1}H NMR

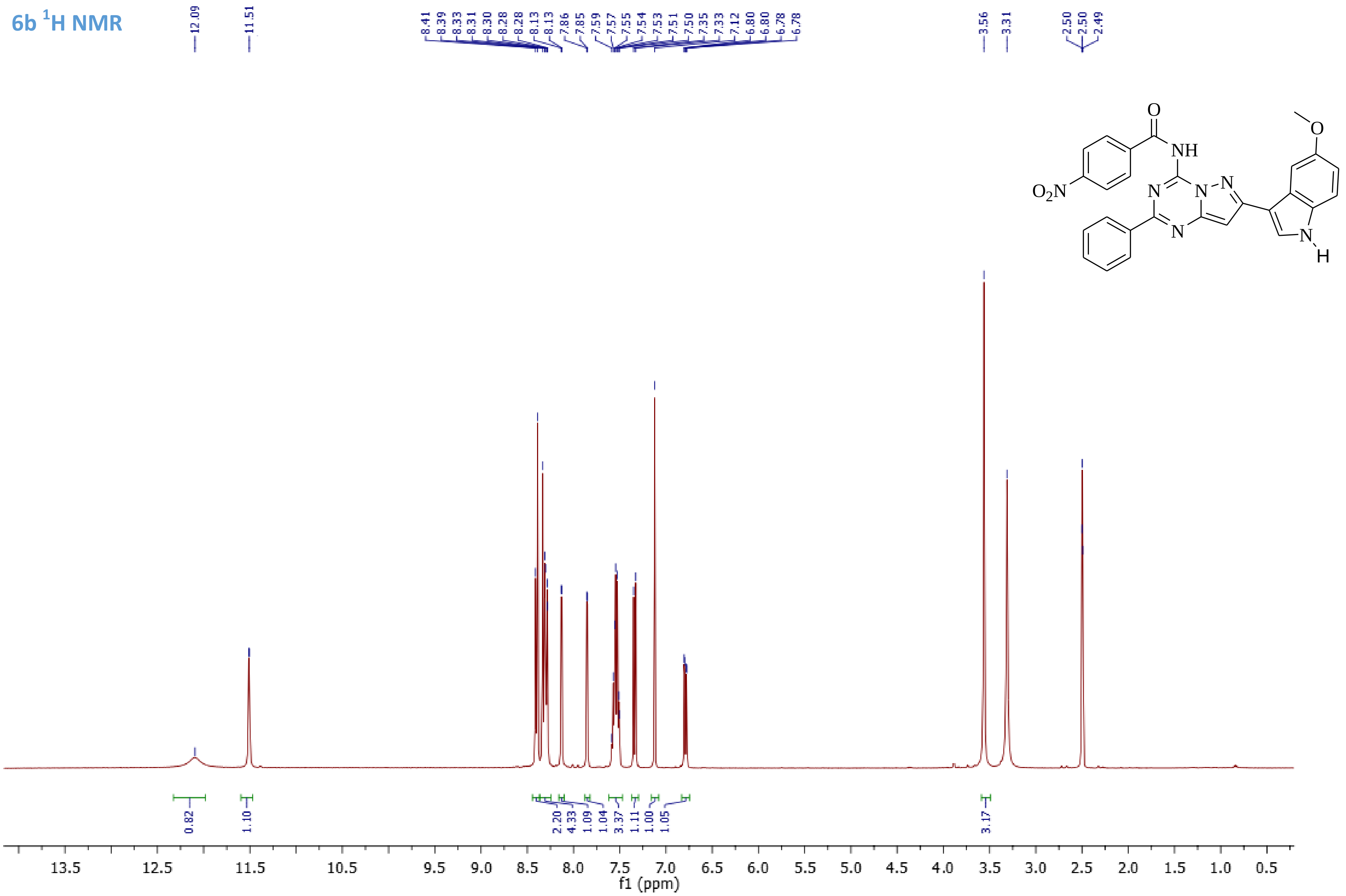
12.09
11.51
8.41
8.39
8.33
8.31
8.30
8.28
8.28
8.13
8.13
7.86
7.85
7.59
7.57
7.55
7.54
7.53
7.51
7.50
7.35
7.33
7.12
6.80
6.80
6.78
6.78
3.56
3.31
2.50
2.50
2.49
O
NH
O_2N
N
N
N
N
N
O
H
0.82
1.10
2.20
4.33
1.09
1.04
3.37
1.11
1.00
1.05
3.17
13.5
12.5
11.5
10.5
9.5
9.0
8.5
8.0
7.5
7.0
6.5
6.0
5.5
5.0
4.5
4.0
3.5
3.0
2.5
2.0
1.5
1.0
0.5
f1 (ppm)

6b ^{13}C NMR

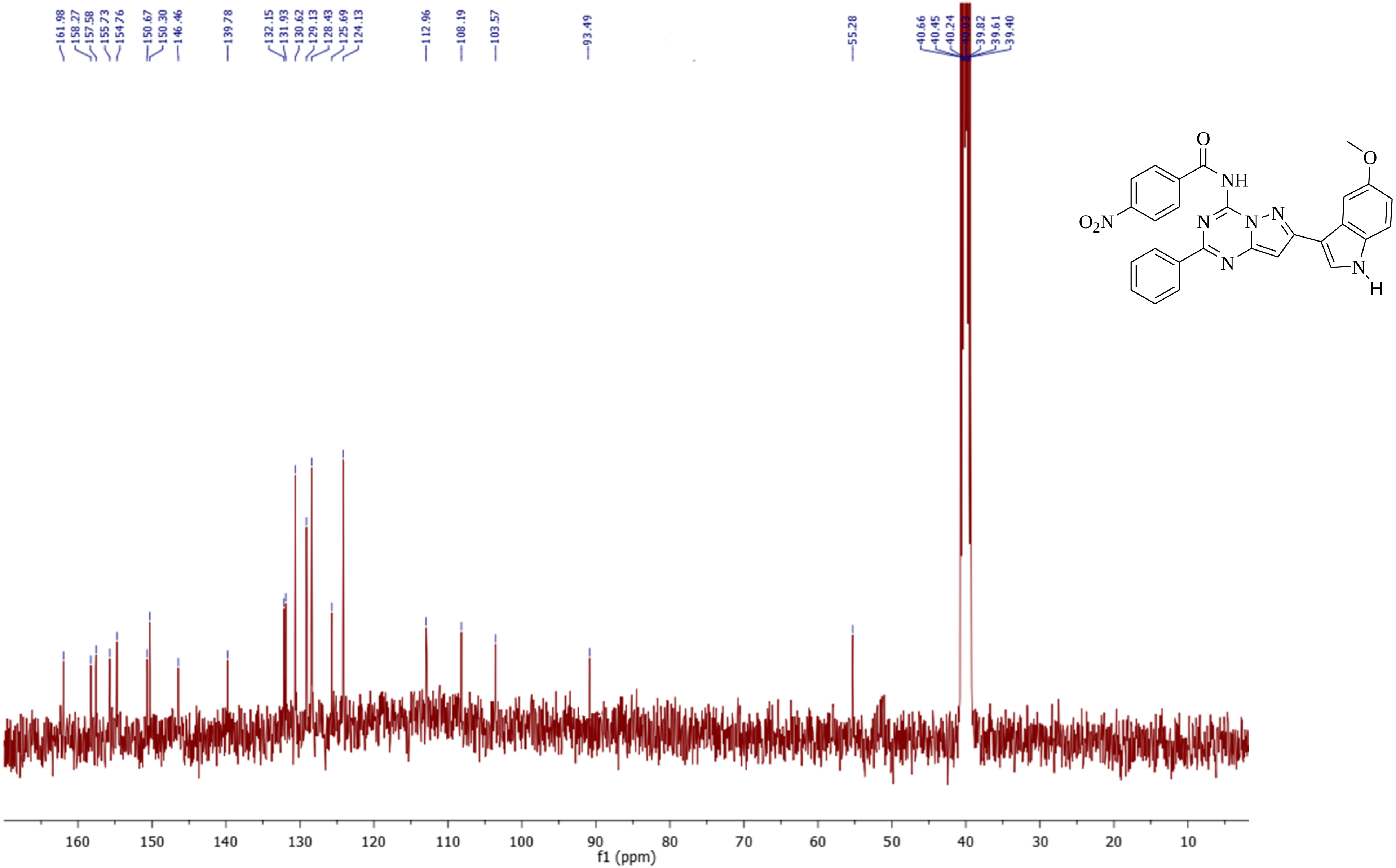
161.98
158.27
157.58
155.73
154.76
150.67
150.30
146.46
139.78
132.15
131.93
130.62
129.13
128.43
125.69
124.13
112.96
108.19
103.57
93.49
55.28
40.66
40.45
40.24
39.82
39.61
39.40
160
150
140
130
120
110
100
90
80
70
60
50
40
30
20
10
f1 (ppm)
O
NH
O2N
N
N
N
N
N
O
N
H

6c ^{1}H NMR

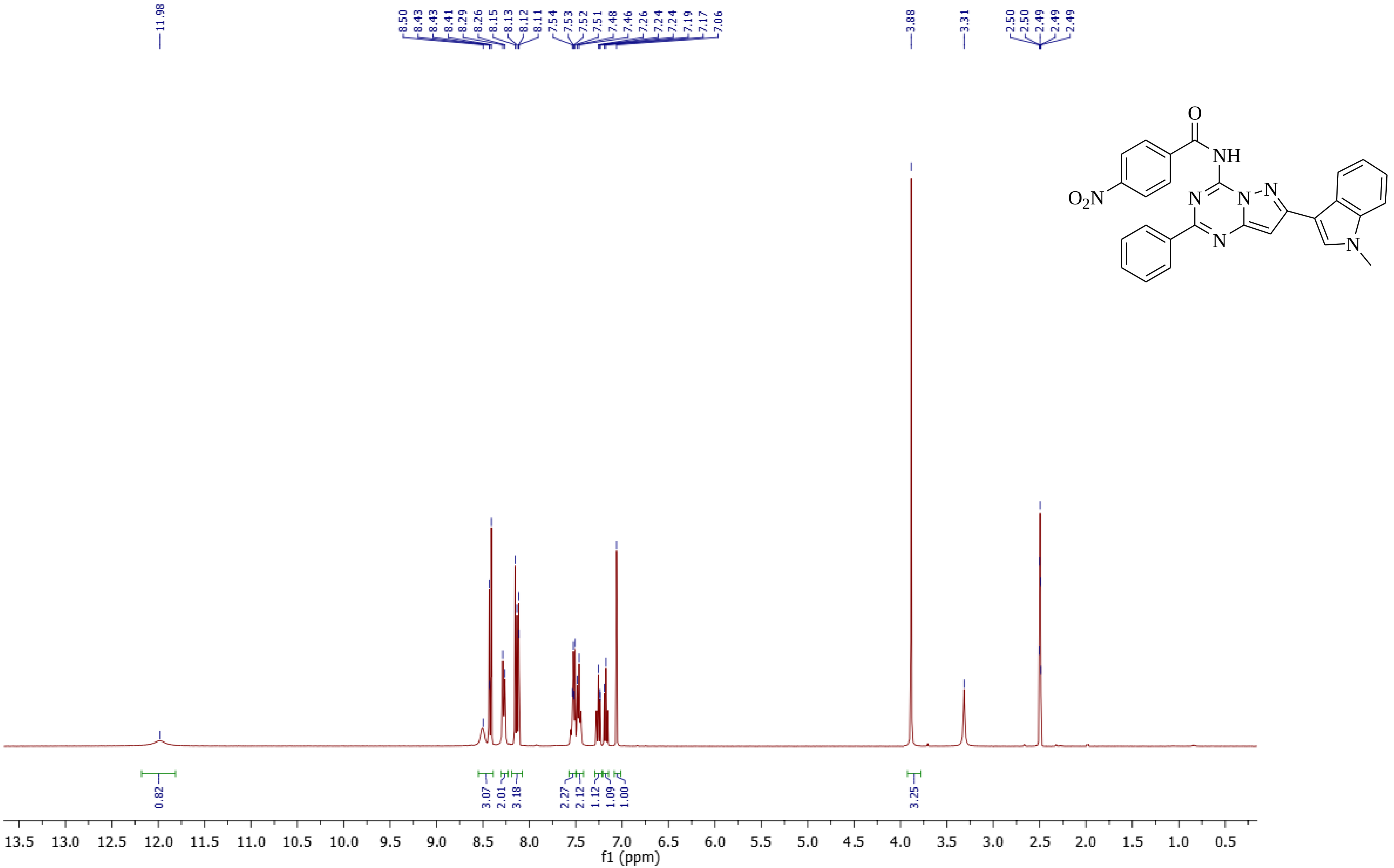
11.98
8.50
8.43
8.43
8.41
8.29
8.26
8.15
8.13
8.12
8.11
7.54
7.53
7.52
7.51
7.48
7.46
7.26
7.24
7.24
7.19
7.17
7.06
3.88
3.31
2.50
2.50
2.49
2.49
2.49
O
NH
O_2N
N
N
N
N
N
0.82
3.07
2.01
3.18
2.27
2.12
1.12
1.09
1.00
3.25
13.5 13.0 12.5 12.0 11.5 11.0 10.5 10.0 9.5 9.0 8.5 8.0 7.5 7.0 6.5 6.0 5.5 5.0 4.5 4.0 3.5 3.0 2.5 2.0 1.5 1.0 0.5
f1 (ppm)

6c ^{13}C NMR

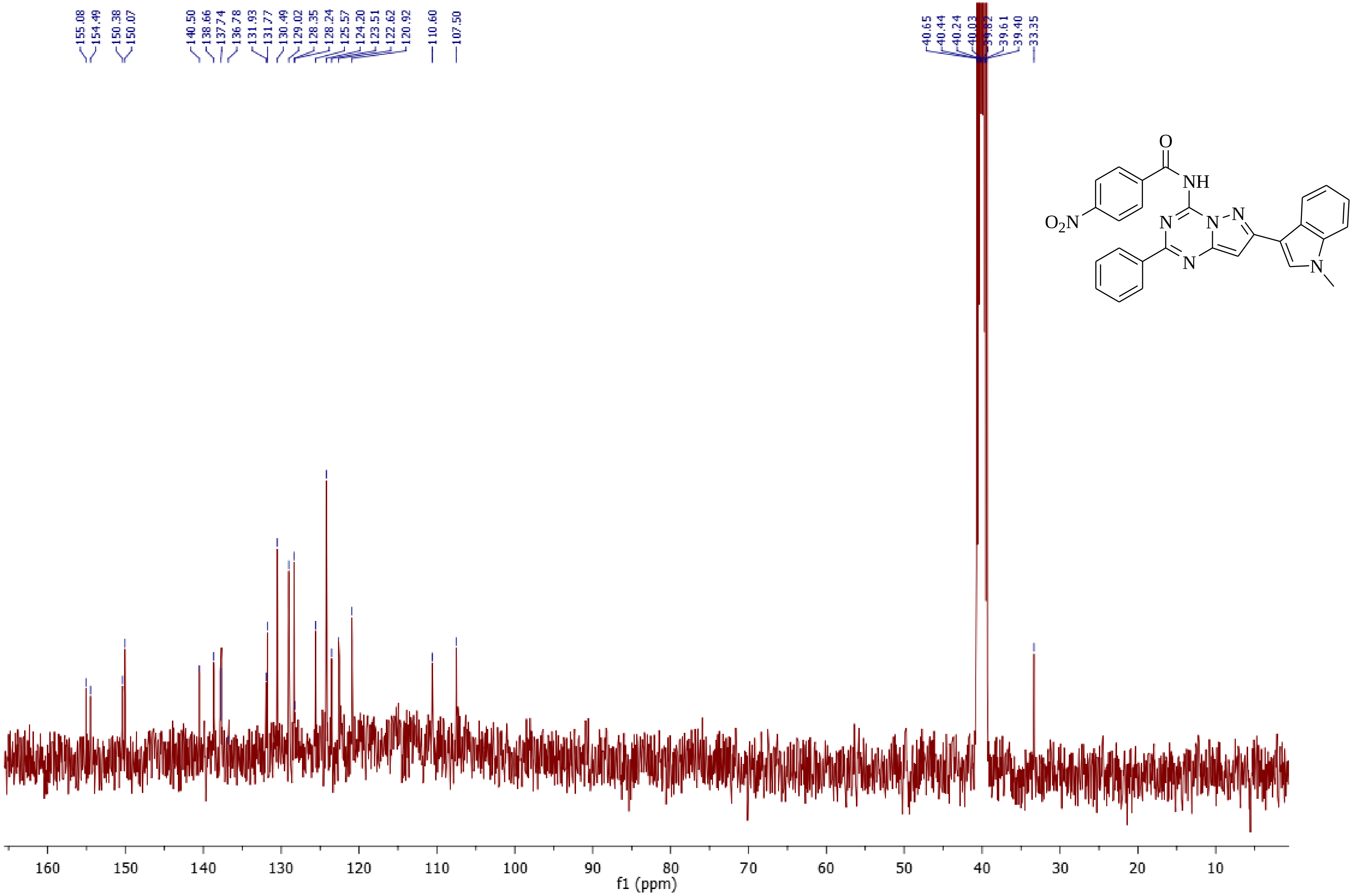

155.08
154.49
150.38
150.07
140.50
138.66
137.74
136.78
131.93
131.77
130.49
129.02
128.35
128.24
125.57
124.20
123.51
122.62
120.92
110.60
107.50
40.65
40.44
40.24
40.03
39.61
39.40
33.35
O
NH
O_2N
N
N
N
N
N
160 150 140 130 120 110 100 90 80 70 60 50 40 30 20 10
f1 (ppm)

6d ^{1}H NMR

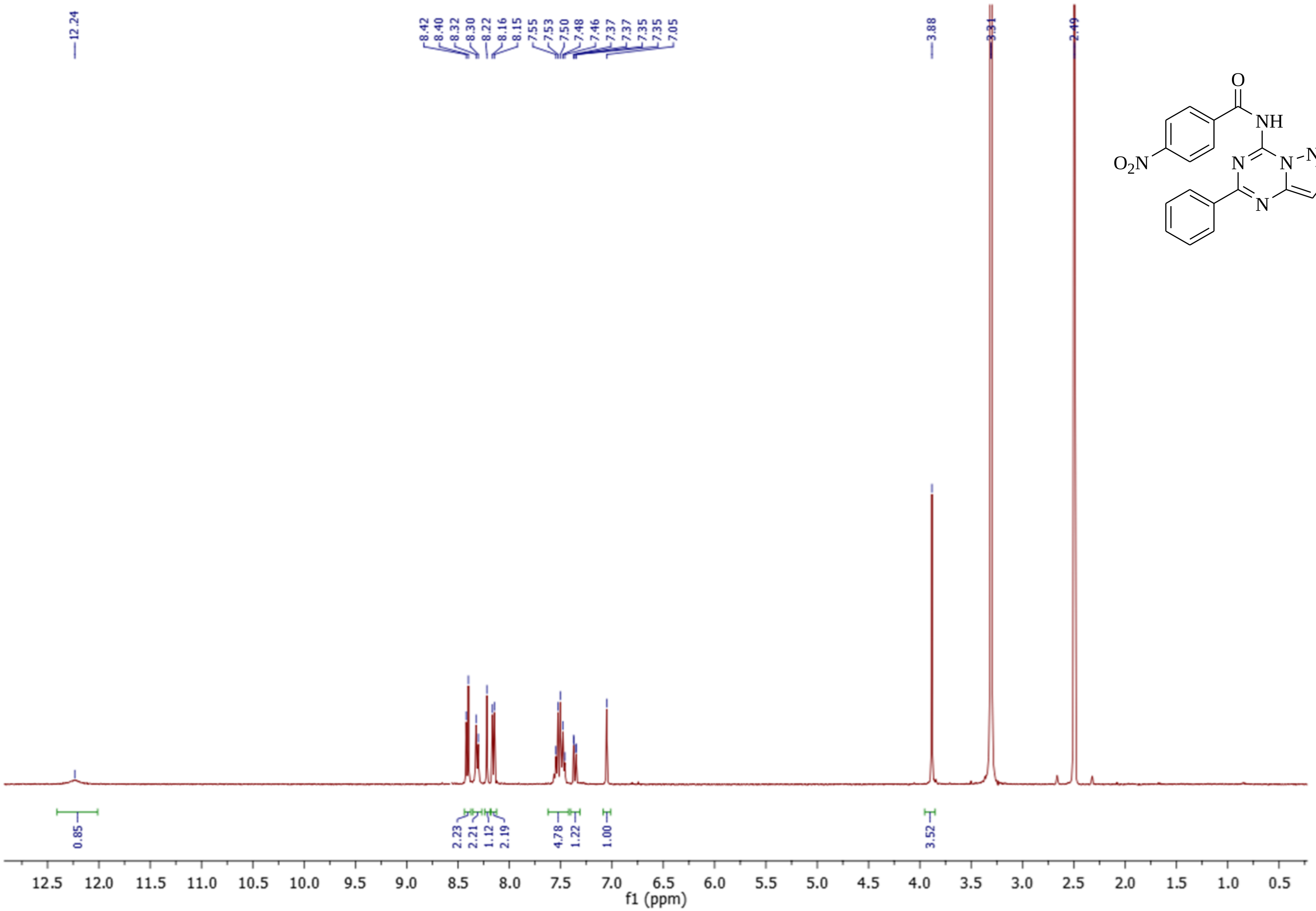
12.24
8.42
8.40
8.32
8.30
8.22
8.16
8.15
7.55
7.53
7.50
7.48
7.46
7.37
7.37
7.35
7.35
7.05
3.88
3.31
2.49
0.85
2.23
2.21
1.12
2.19
4.78
1.22
1.00
3.52
f1 (ppm)
O
NH
Br
O_2N
N
N
N
N
N
N

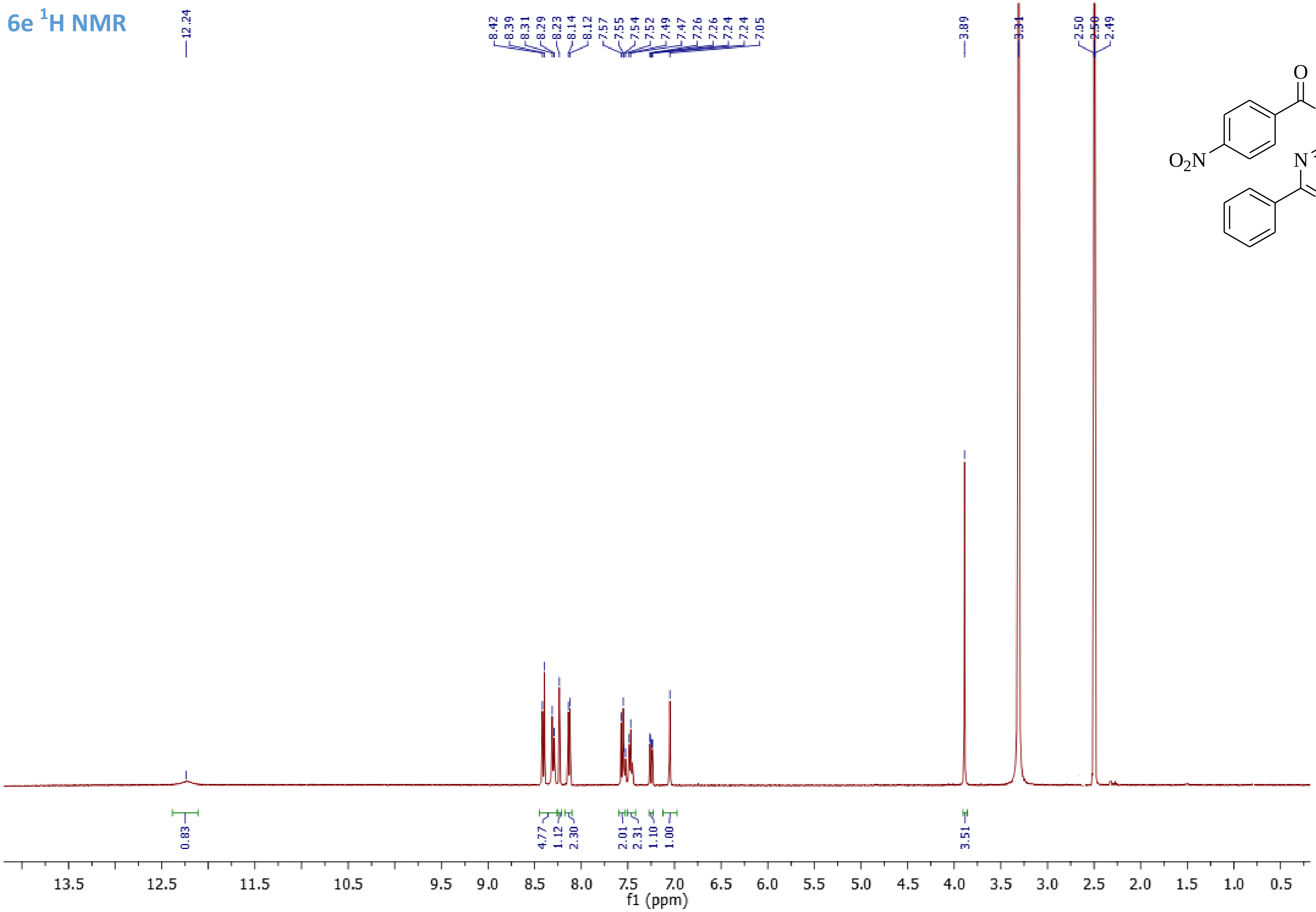
6e 1H NMR
12.24
8.42
8.39
8.31
8.29
8.23
8.14
8.12
7.57
7.55
7.54
7.52
7.49
7.47
7.26
7.26
7.24
7.24
7.05
3.89
3.31
2.50
2.50
2.49
0.83
4.77
1.12
2.30
2.01
2.31
1.10
1.00
3.51
13.5
12.5
11.5
10.5
9.5
9.0
8.5
8.0
7.5
7.0
6.5
6.0
5.5
5.0
4.5
4.0
3.5
3.0
2.5
2.0
1.5
1.0
0.5
f1 (ppm)

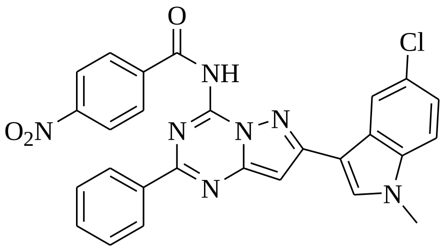
O
NH
Cl
O_2N
N
N
N
N
N
N

6f ^{1}H NMR

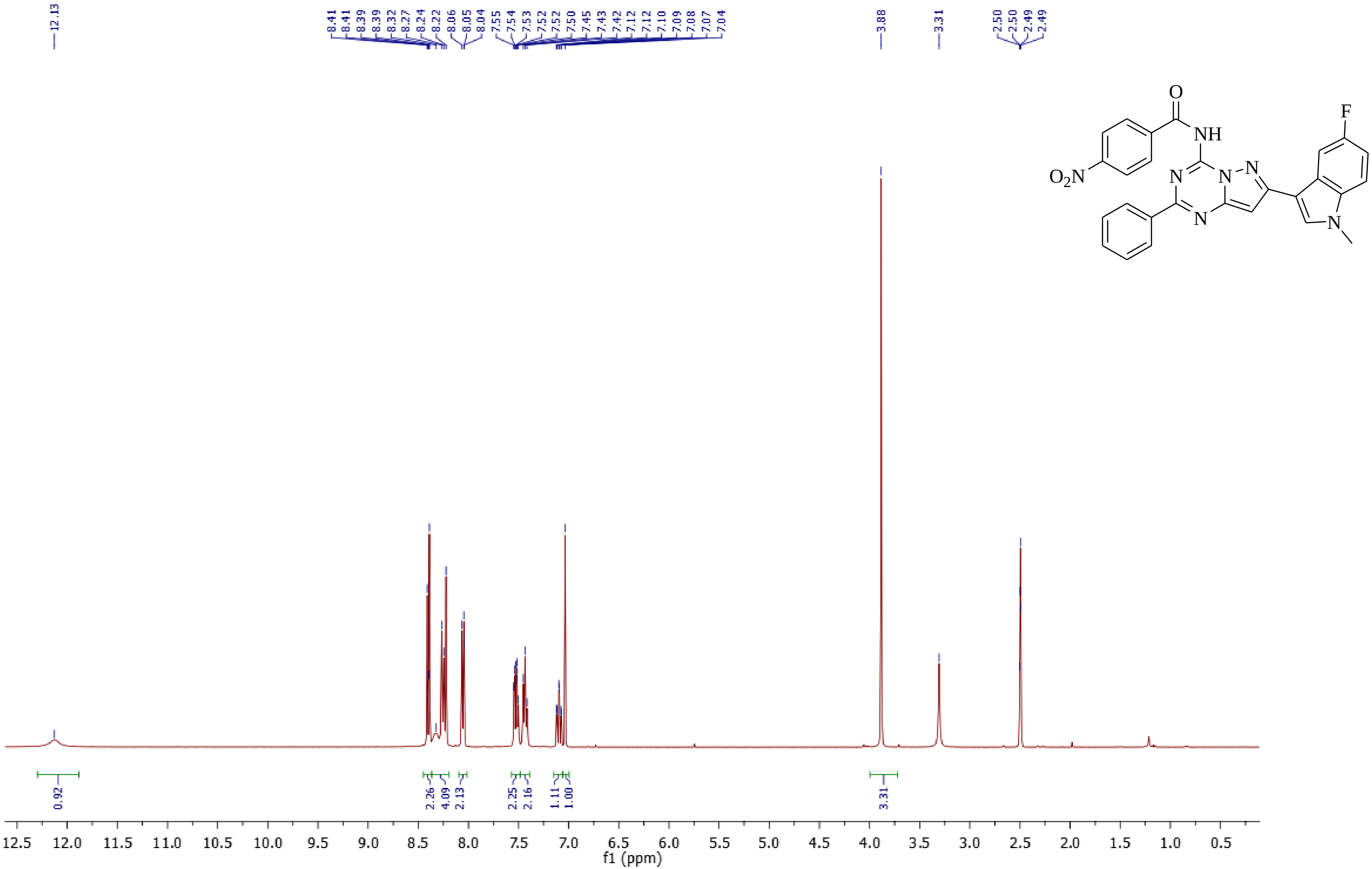
12.13
8.41
8.41
8.39
8.39
8.32
8.27
8.24
8.22
8.06
8.05
8.04
7.55
7.54
7.53
7.52
7.52
7.50
7.45
7.43
7.42
7.12
7.12
7.10
7.09
7.08
7.07
7.04
3.88
3.31
2.50
2.50
2.49
2.49
0.92
2.26
4.09
2.13
2.25
2.16
1.11
1.00
3.31
12.5 12.0 11.5 11.0 10.5 10.0 9.5 9.0 8.5 8.0 7.5 7.0 6.5 6.0 5.5 5.0 4.5 4.0 3.5 3.0 2.5 2.0 1.5 1.0 0.5
f1 (ppm)
O
NH
F
O2N
N
N
N
N
N

6g ¹H NMR

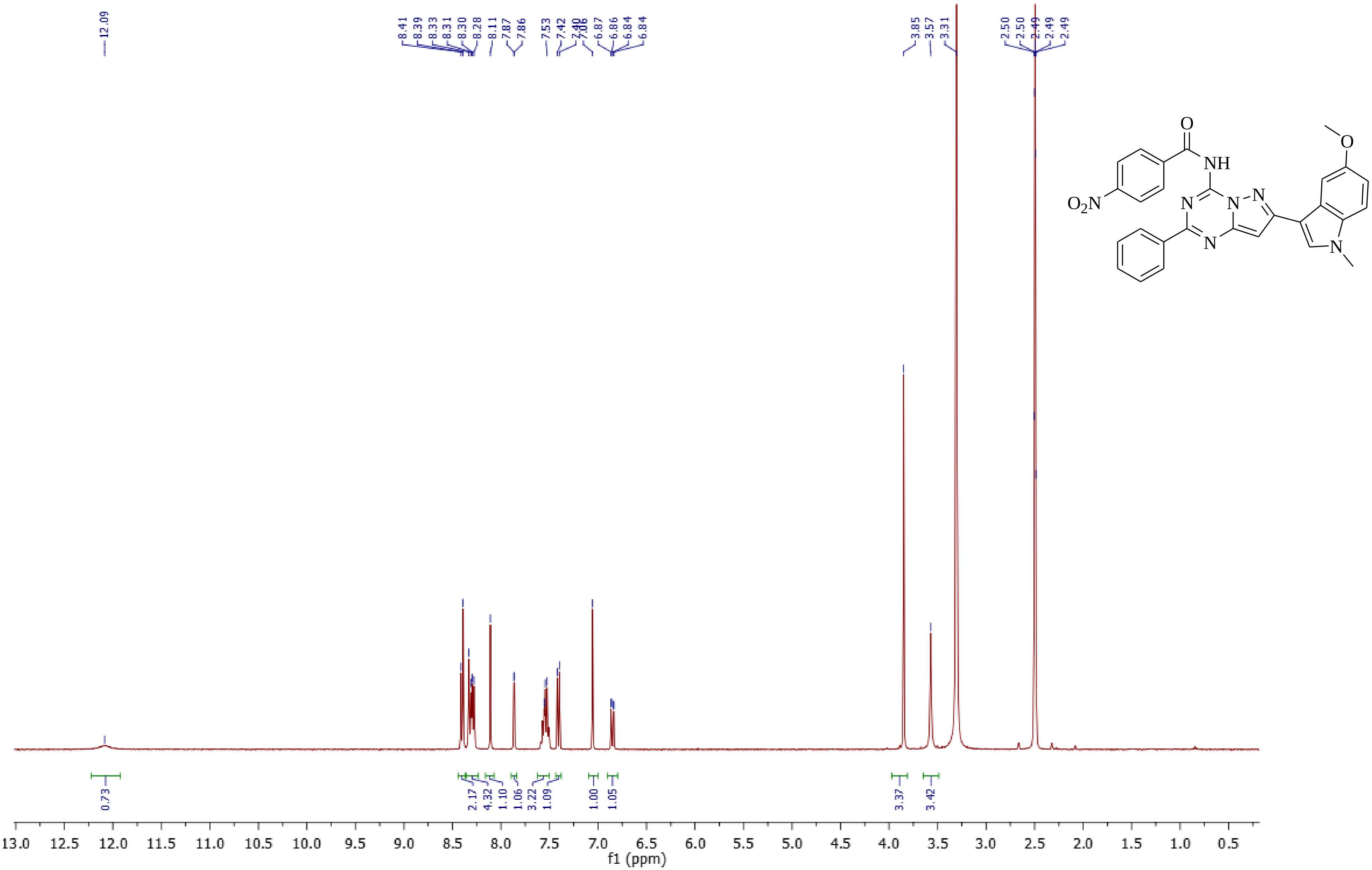

Inhibition of cell growth curves with IC_{50} values indicating concentrations that inhibit 50% of cell growth

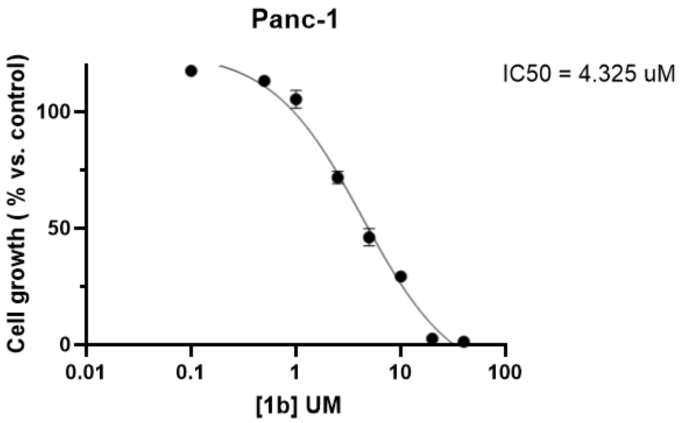

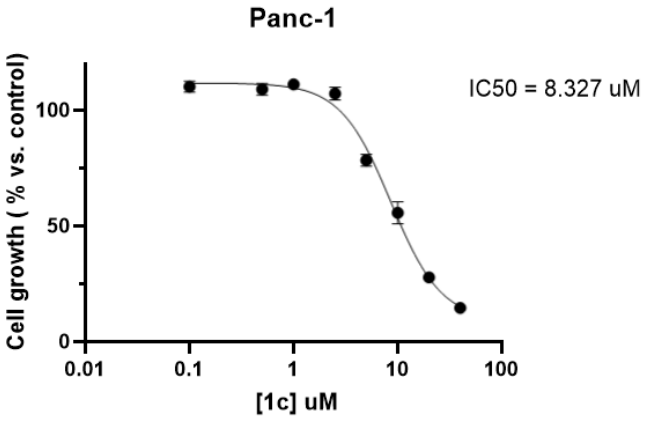

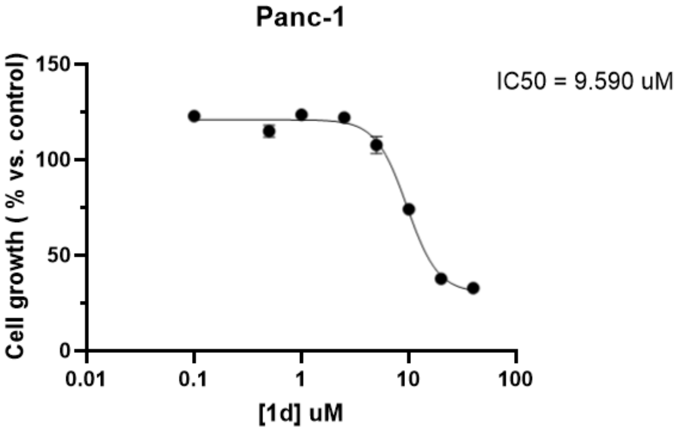

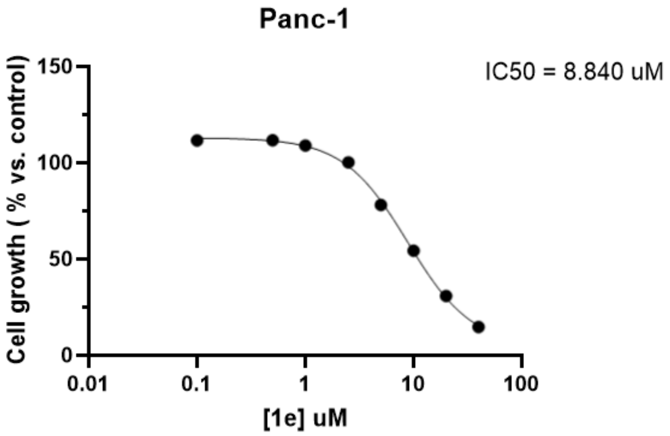
Panc-1
IC50 = 8.840 uM
Cell growth (% vs. control)
150
100
50
0
0.01
0.1
1
10
100
[1e] uM

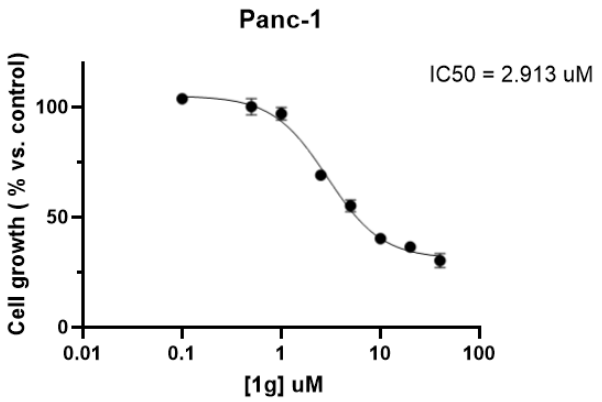
Panc-1
IC50 = 2.913 uM
Cell growth (% vs. control)
100
50
0
0.01
0.1
1
10
100
[1g] uM

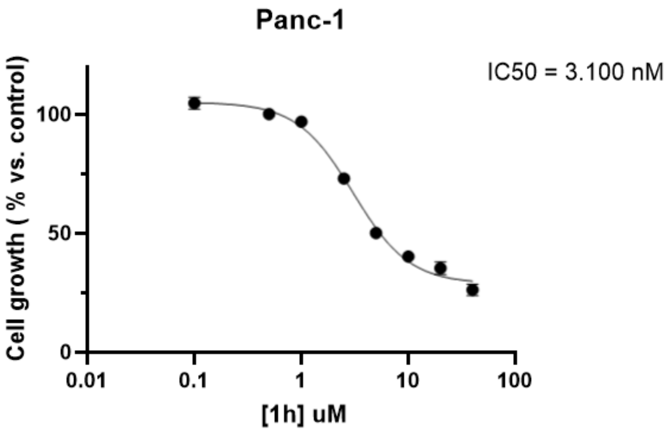
Panc-1
IC50 = 3.100 nM
Cell growth (% vs. control)
100
50
0
0.01
0.1
1
10
100
[1h] uM

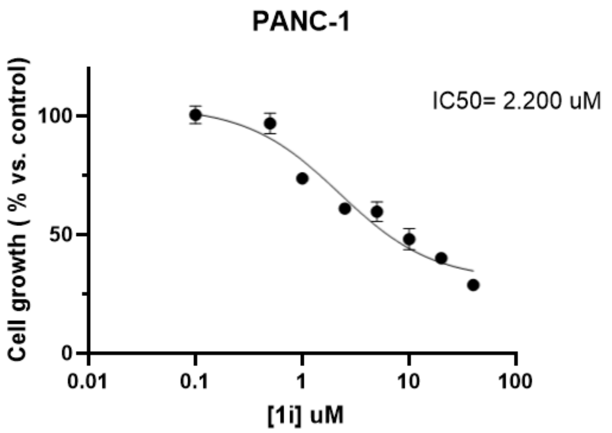
PANC-1
IC50= 2.200 uM
Cell growth (% vs. control)
100
50
0
0.01
0.1
1
10
100
[1i] uM

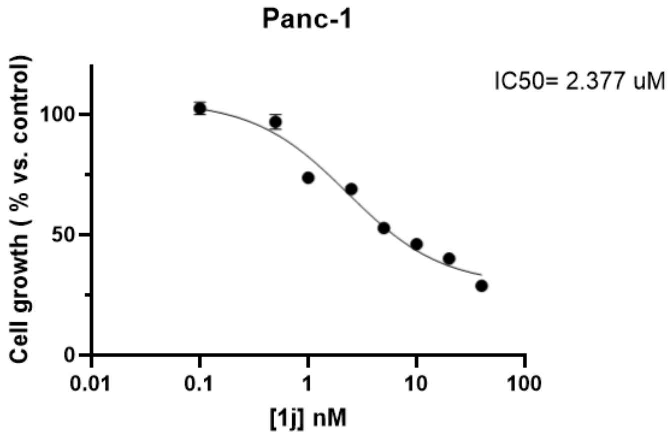
Panc-1
IC50= 2.377 uM
Cell growth (% vs. control)
100
50
0
0.01
0.1
1
10
100
[1j] nM

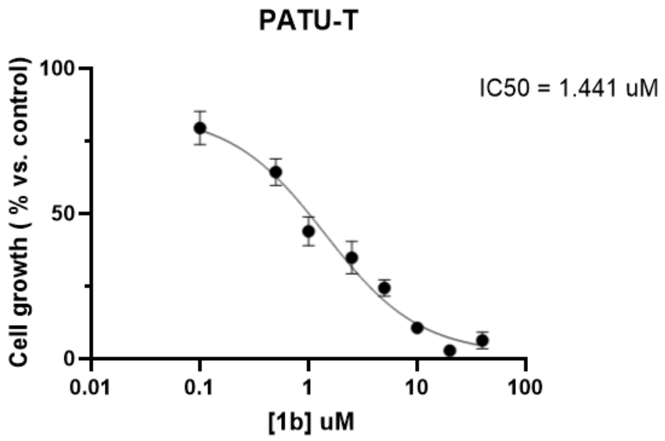
PATU-T
IC50 = 1.441 uM
Cell growth (% vs. control)
100
50
0
0.01
0.1
1
10
100
[1b] uM

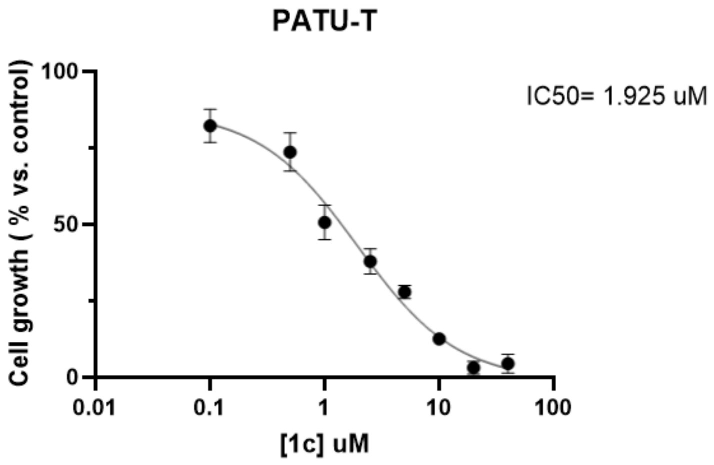
PATU-T
IC50= 1.925 uM
Cell growth (% vs. control)
100
50
0
0.01
0.1
1
10
100
[1c] uM

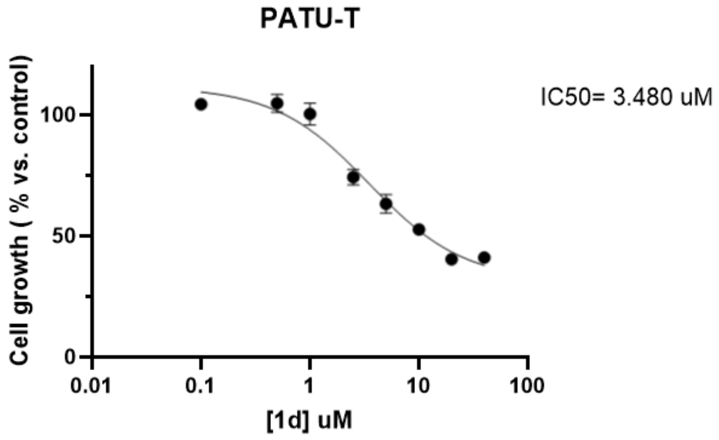
PATU-T
IC50= 3.480 uM
Cell growth (% vs. control)
100
50
0
0.01
0.1
1
10
100
[1d] uM

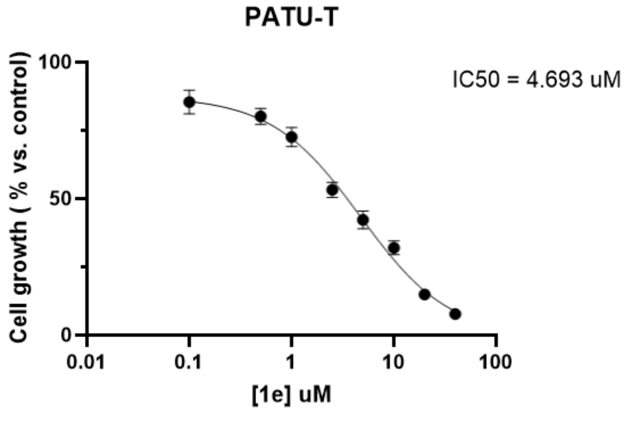
PATU-T
IC50 = 4.693 uM
Cell growth (% vs. control)
100
50
0
0.01
0.1
1
10
100
[1e] uM

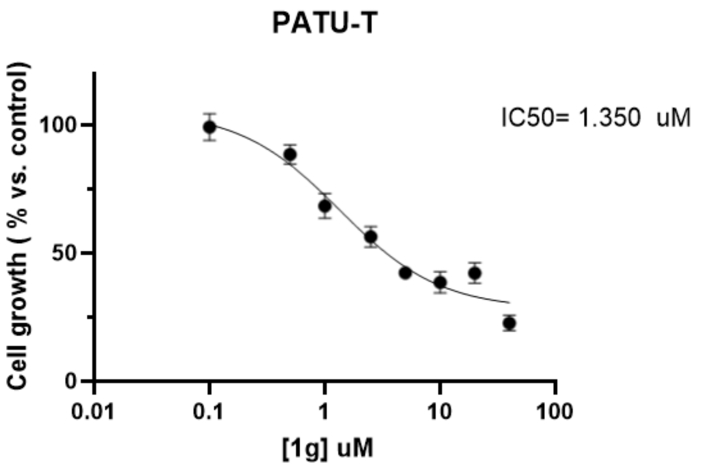
PATU-T
IC50= 1.350 uM
Cell growth (% vs. control)
100
50
0
0.01
0.1
1
10
100
[1g] uM

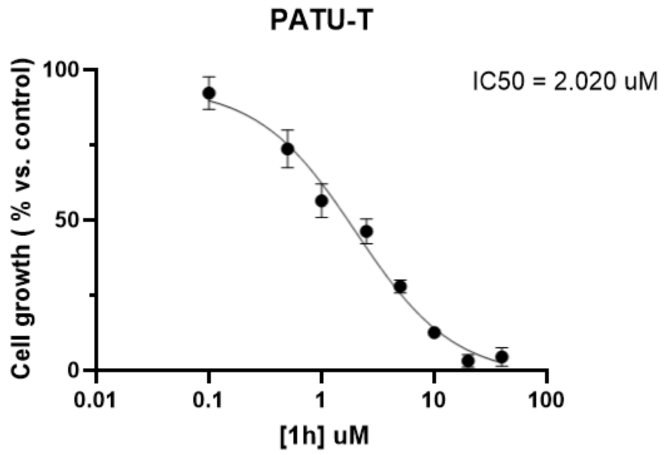
PATU-T
IC50 = 2.020 uM
Cell growth (% vs. control)
100
50
0
0.01
0.1
1
10
100
[1h] uM

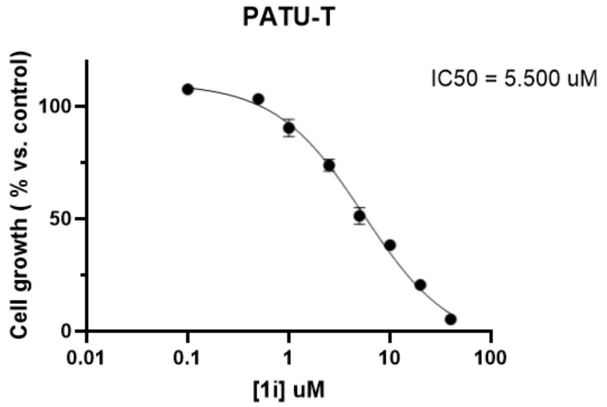
PATU-T
IC50 = 5.500 uM
Cell growth (% vs. control)
100
50
0
0.01
0.1
1
10
100
[1i] uM

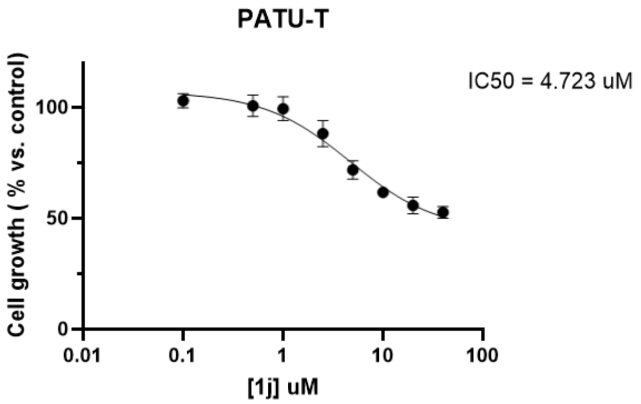
PATU-T
IC50 = 4.723 uM
Cell growth (% vs. control)
100
50
0
0.01
0.1
1
10
100
[1j] uM

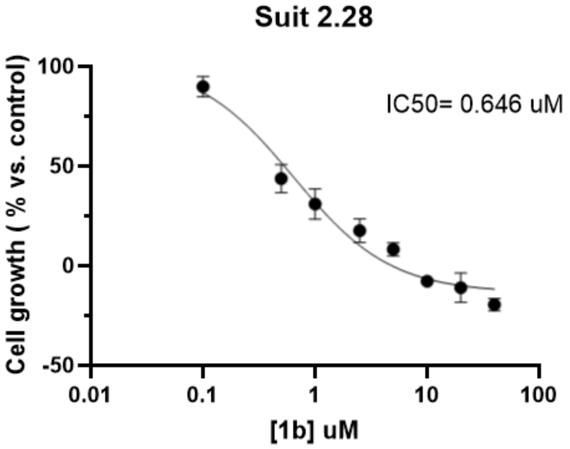
Suit 2.28
IC50= 0.646 uM
Cell growth (% vs. control)
100
50
0
-50
0.01
0.1
1
10
100
[1b] uM

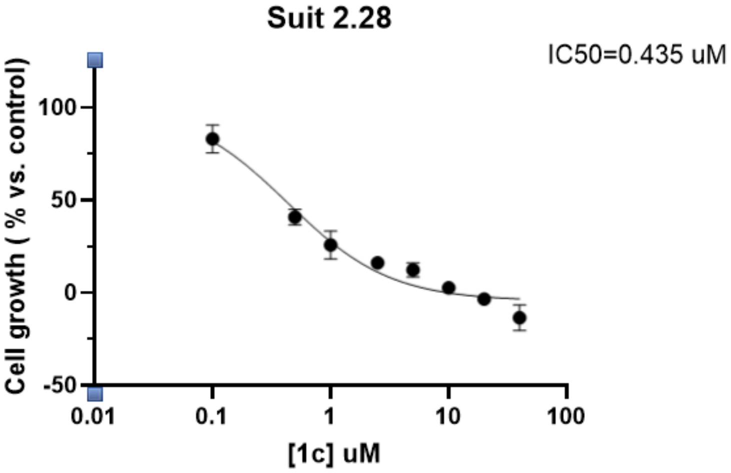
Suit 2.28
IC50=0.435 uM
Cell growth (% vs. control)
100
50
0
-50
0.01
0.1
1
10
100
[1c] uM

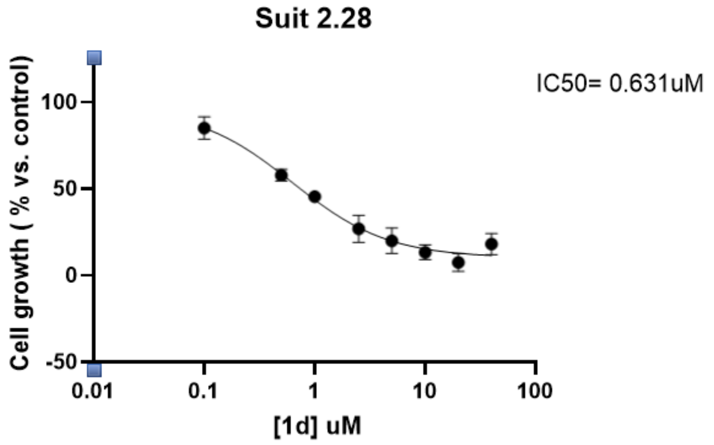
Suit 2.28
IC50= 0.631uM
Cell growth (% vs. control)
100
50
0
-50
0.01
0.1
1
10
100
[1d] uM

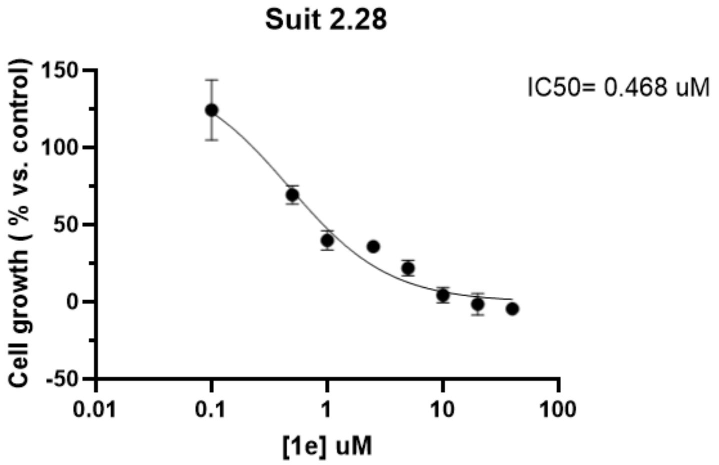
Suit 2.28
IC50= 0.468 uM
Cell growth (% vs. control)
150
100
50
0
-50
0.01
0.1
1
10
100
[1e] uM

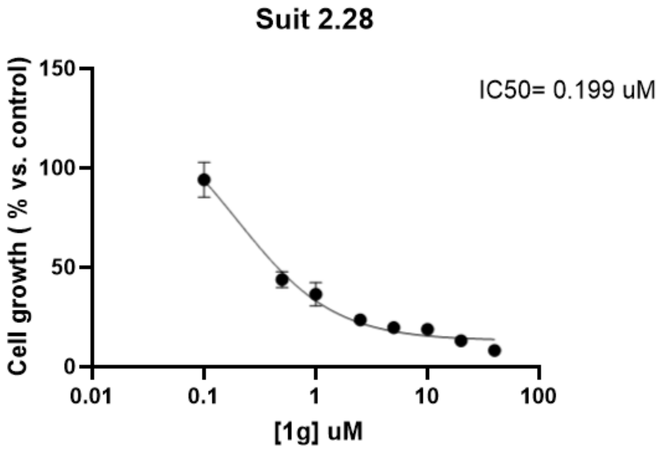
Suit 2.28
IC50= 0.199 uM
Cell growth (% vs. control)
150
100
50
0
0.01
0.1
1
10
100
[1g] uM

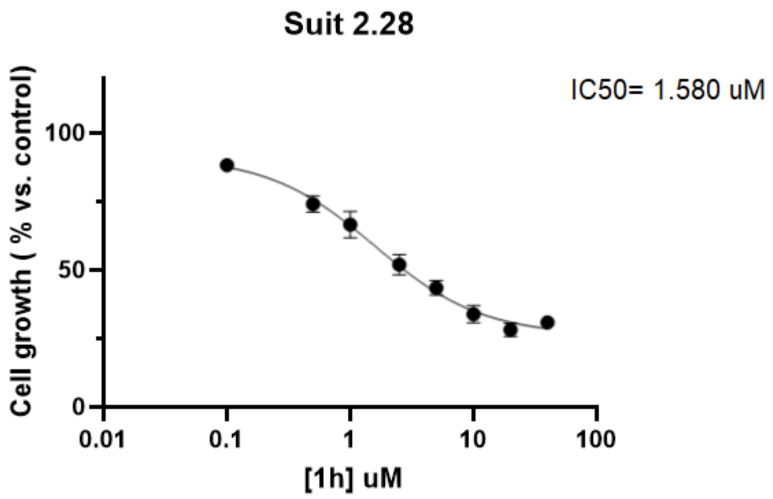
Suit 2.28
IC50= 1.580 uM
Cell growth (% vs. control)
100
50
0
0.01
0.1
1
10
100
[1h] uM

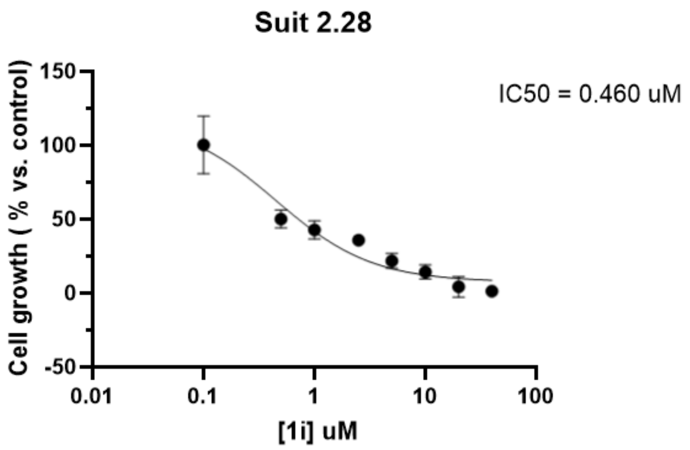
Suit 2.28
IC50 = 0.460 uM
Cell growth (% vs. control)
150
100
50
0
-50
0.01
0.1
1
10
100
[1i] uM

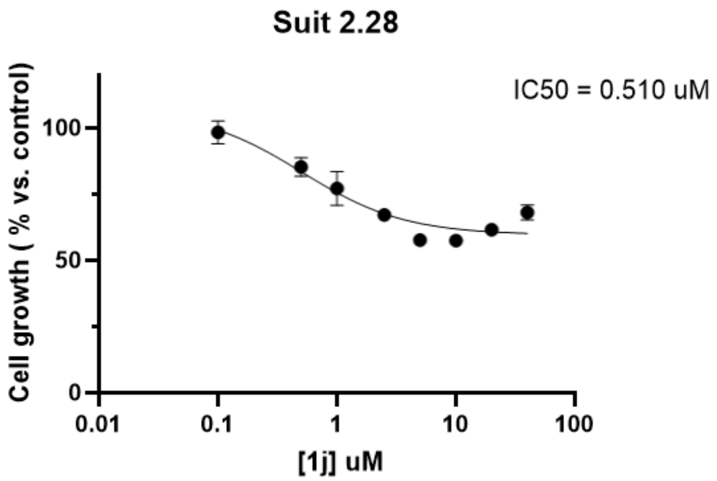
Suit 2.28
IC50 = 0.510 uM
Cell growth (% vs. control)
100
50
0
0.01
0.1
1
10
100
[1j] uM

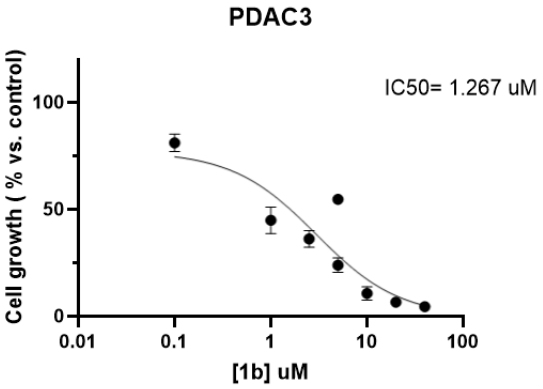
PDAC3
IC50= 1.267 uM
Cell growth (% vs. control)
100
50
0
0.01
0.1
1
10
100
[1b] uM

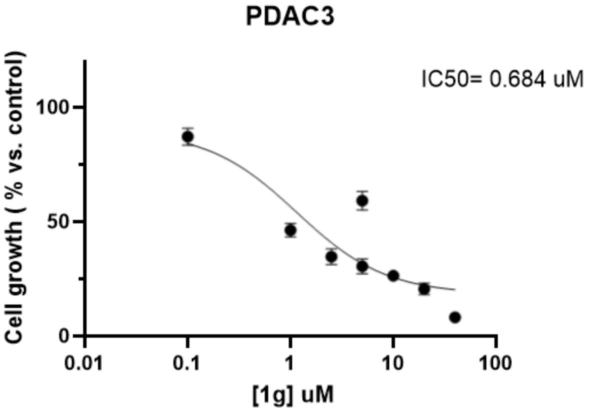
PDAC3
IC50= 0.684 uM
Cell growth (% vs. control)
100
50
0
0.01
0.1
1
10
100
[1g] uM

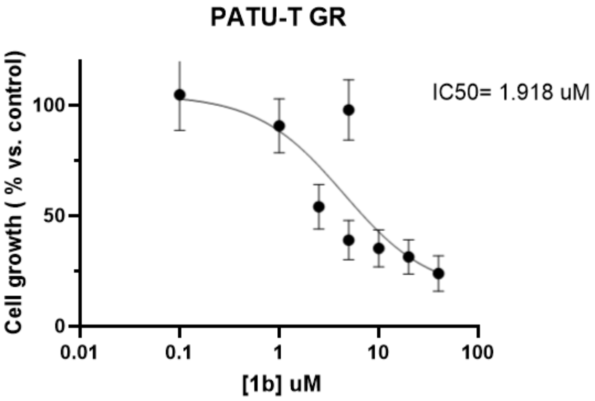
PATU-T GR
IC50= 1.918 uM
Cell growth (% vs. control)
100
50
0
0.01
0.1
1
10
100
[1b] uM

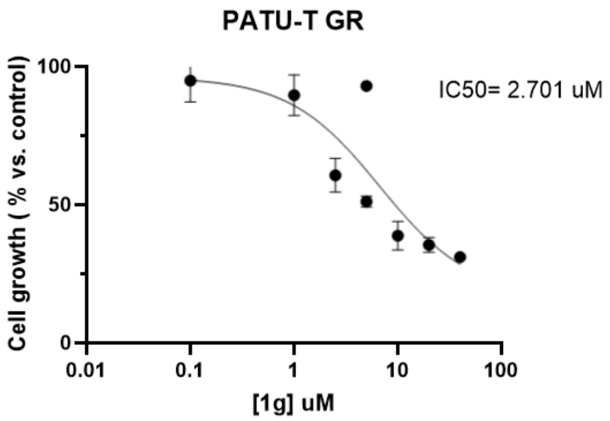
PATU-T GR
IC50= 2.701 uM
Cell growth (% vs. control)
100
50
0
0.01
0.1
1
10
100
[1g] uM

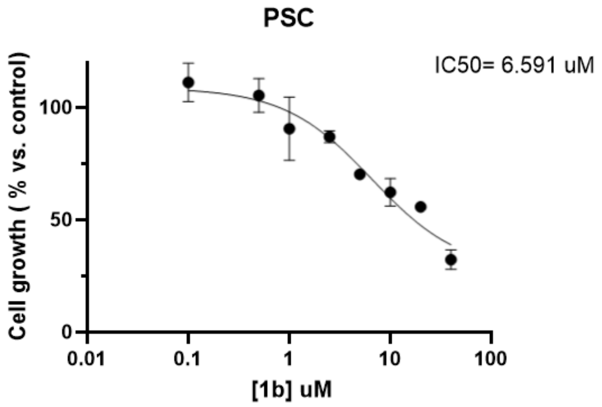
PSC
IC50= 6.591 uM
Cell growth (% vs. control)
100
50
0
0.01
0.1
1
10
100
[1b] uM

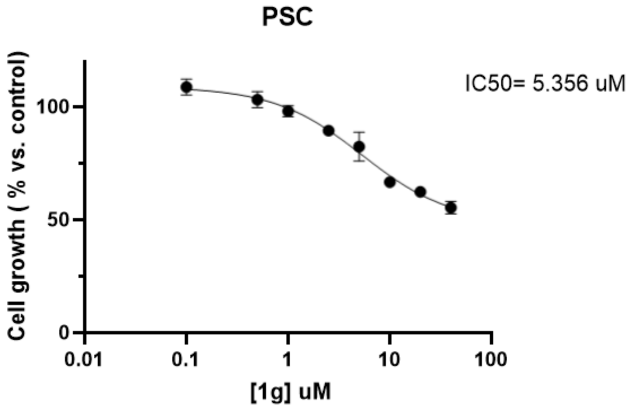
PSC
IC50= 5.356 uM
Cell growth (% vs. control)
100
50
0
0.01
0.1
1
10
100
[1g] uM
